# DNA-Microarray-based Genotyping of *Clostridium difficile*

**DOI:** 10.1186/s12866-015-0489-2

**Published:** 2015-08-05

**Authors:** Darius Gawlik, Peter Slickers, Ines Engelmann, Elke Müller, Christian Lück, Anette Friedrichs, Ralf Ehricht, Stefan Monecke

**Affiliations:** Institute for Medical Microbiology and Hygiene, Technische Universität Dresden, Dresden, Germany; Hamm-Lippstedt University, Hamm, Germany; Alere Technologies GmbH, Jena, Germany; Department of Internal Medicine I, University Hospital Schleswig-Holstein, Campus Kiel, Kiel, Germany; Infectognostics Research Campus, Jena, Germany

**Keywords:** *Clostridium difficile*, DNA-microarray, Molecular typing, Surveillance

## Abstract

**Background:**

*Clostridium difficile* can cause antibiotic-associated diarrhea and a possibility of outbreaks in hospital settings warrants molecular typing. A microarray was designed that included toxin genes (*tcdA/B, cdtA/B*), genes related to antimicrobial resistance, the* slpA *gene and additional variable genes.

**Results:**

DNA of six reference strains and 234 clinical isolates from South-Western and Eastern Germany was subjected to linear amplification and labeling with dUTP-linked biotin. Amplicons were hybridized to microarrays providing information on the presence of target genes and on their alleles. Tested isolates were assigned to 37 distinct profiles that clustered mainly according to MLST-defined clades. Three additional profiles were predicted from published genome sequences, although they were not found experimentally.

**Conclusions:**

The microarray based assay allows rapid and high-throughput genotyping of clinical *C. difficile* isolates including toxin gene detection and strain assignment. Overall hybridization profiles correlated with MLST-derived clades.

**Electronic supplementary material:**

The online version of this article (doi:10.1186/s12866-015-0489-2) contains supplementary material, which is available to authorized users.

## Background

*Clostridium difficile *is a component of the human colonic flora. If the physiological bacterial flora in the colon is altered or damaged by antibiotics, especially by clindamycin, fluoroquinolones, cephalosporins, or amoxicillin/clavulanic acid [1, 2], *C. difficile* is able to multiply and to cause damage due to its production of several toxins. Resulting conditions are antibiotic-associated diarrhea and pseudomembranous colitis (for a recent review, see [1]). Severe cases might progress to toxic megacolon and end fatally [3].

Important virulence factors are secreted toxins TcdA and TcdB, encoded by genes *tcdA* and *tcdB* [4] that form a pathogenicity locus together with regulatory genes (*tcdC *and *tcdD*) and a gene (tcdE) encoding a holin-like pore-forming protein [5]. TcdA and TcdB irreversibly modify GTPases from the Ras superfamily resulting in disruption of vital signaling pathways of the cell and in cell death [4]. Besides, some *C. difficile* strains harbor a binary toxin encoded by *cdtA *and *cdtB*. The binary toxin appears to modify actin via its ADP-ribosyltransferase activity. Its clinical significance is not yet fully elucidated [4, 6, 7]

The therapy of *C. difficile* infection includes rehydration, discontinuation of antibiotics triggering the condition, oral administration of vancomycin or metronidazole as well as surgical intervention in severe cases [1]. Relapses are common, either due to surviving spores, or to re-infection. A possible role of probiotics is still investigated as well as the concept of transplanting feces in order to restore the physiological flora [8, 9]. With increasing numbers of patients who receive long-term, broad-spectrum antibiotic therapies, *C. difficile* became an increasingly important problem in healthcare. Case numbers as well as fatality rates are increasing; with the latter being attributed to the emergence of more virulent strains [10].

Transmissions of *C. difficile* and even outbreaks within hospital settings are common, given that spores are able to survive in a clinical environment and are resistant to alcoholic disinfectants [1]. Hospitalizations, or residence in nursing homes, are significant risk factors for acquisition of *C. difficile*, and 50 % of patients who stayed in hospital for more than one month acquired *C. difficile* [11]. Transmissions within healthcare setting justify infection control measures, in analogy to,* e.g.*, methicillin-resistant *S. aureus*. Besides barrier nursing, isolation, disinfection, etc., this also should include molecular typing in order to trace chains of infections. A variety of methods that included multilocus sequence typing (MLST), sequencing of the *slpA* gene, multilocus variable-number tandem-repeat analysis and ribotyping has been described previously [12–17] and genome sequencing might become an option in the future.

Microarray-based rapid typing proved to be a convenient tool for MRSA genotyping [18] allowing both, virulence and resistance gene detection and molecular typing within one experiment. Therefore, a microarray-based assay was designed to prove this concept for *C. difficile.*

## Results

### Profile- and MLST based clade assignment

Data for a subset of most relevant target genes are presented in Table 1; full data are provided in the Additional file 1.

**Table 1:** Detected hybridization pattern types and their association with ribo- and sequence types as well as toxin gene alleles and resistance markers

Hybridi-sation profile	Fully sequenced reference strains	Additional genome sequences, that were analyzed in silico only	Tested isolates	Clade	Associated sequence types	*slpA *allele	Associated ribotypes	*tcdA*	*tcdB*	*cdtA/B*	*bcrA*	*lmrB*	*vatA*	*cat*	*erm* (B)	*tet* (M)	*vncS/vexP*1
HP-01	BI-9 (FN668944)	QCD-63q42* (ABHD)	71	I	ST-03	*slpA*_BI9_	RT-001, RT-015, RT-072	*tcdA*_R20291_	*tcdB*_630_	*cdtA*_630_+ *cdtB*_630_	*bcrA*_630_	*lmrB*_630_	*vatA*_630_	-	(var)	-	pos
HP-02	-	ATCC43255* (ABKJ)	5	I	ST45, ST-46*	*slpA*_BI9_	RT-001, RT-013, RT-087	*tcdA*_R20291_	*tcdB*_630_	*cdtA*_630_+ *cdtB*_630_	*bcrA*_630_	*lmrB*_630_	*vatA*_630_	-	-	-	-
HP-03	-	-	4	I	ST-58	*slpA*_6407_	RT-011, RT-049, RT-056	*tcdA*_R20291_	*tcdB*_630_	*cdtA*_630_+ *cdtB*_630_	*bcrA*_630_	*lmrB*_630_	*vatA*_630_	-	(var)	(var)	-
HP-04	-	-	5	I	ST-04	*slpA*_630_	RT-137, RT-150	*tcdA*_R20291_	*tcdB*_630_	(*cdtA*_630_+ *cdtB*_630_ )	*bcrA*_630_	*lmrB*_630_	*vatA*_630_				-
HP-05	-	-	2	I	N/A	*slpA*_DJNS0578_	RT-163	*tcdA*_R20291_	*tcdB*_630_	*cdtA*_630_+ *cdtB*_630_	*bcrA*_630_	*lmrB*_630_	*vatA*_630_	-	-	-	-
HP-06	-	-	1	I	N/A	*slpA *negative	Unidentified pattern	*tcdA*_R20291_	*tcdB*_630_	*cdtA*_630_+ *cdtB*_630_	*bcrA*_630_	*lmrB*_630_	*vatA*_630_	-	-	-	-
HP-07	-	-	1	I	N/A	*slpA*_R12884_ trunc.	RT-054	*tcdA*_R20291_	*tcdB*_630_	*cdtA*_630_+ *cdtB*_630_	*bcrA*_630_	*lmrB*_630_	*vatA*_630_	-	-	-	-
HP-08	-	-	10	I	ST-55	*slpA*_R13711_	RT-057, RT-070, RT-094	*tcdA*_R20291_	*tcdB*_630_	*cdtA*_630_+ *cdtB*_630_	*bcrA*_630_	*lmrB*_630_	*vatA*_630_	-			-
HP-09	-	-	17	I	ST-08	*slpA*_R13541_	RT-002, RT-159	*tcdA*_R20291_	*tcdB*_630_	*cdtA*_630_+ *cdtB*_630_	*bcrA*_630_	*lmrB*_630_	*vatA*_630_	-	-	(var)	-
HP-10	-		7	I	N/A	*slpA*_23m63_	N/A	*tcdA*_R20291_	*tcdB*_630_	*cdtA*_630_+ *cdtB*_630_	*bcrA*_630_	*lmrB*_630_	*vatA*_630_	-	-	-	-
HP-11		CD37*, (AHJJ)	2	I	ST-03	*slpA*_23m63_	RT-009	-	-	-	*bcrA*_630_	*lmrB*_630_	*vatA*_630_			(var)	-
HP-12	-	-	2	I	N/A	*slpA*_JND08162_	RT-103	*tcdA*_R20291_	*tcdB*_630_	*cdtA*_630_+ *cdtB*_630_	*bcrA*_630_	*lmrB*_630_	*vatA*_630_	-	-	-	-
HP-13	-	70-100-2010*, (AGAC)	24	I	ST42*	*slpA*_R12885_	RT-014, RT-049	*tcdA*_R20291_	*tcdB*_630_	*cdtA*_630_+ *cdtB*_630_	*bcrA*_630_	*lmrB*_630_	*v* *atA*_630_	-	-	-	-
HP-14	-	-	1	I	N/A	*slpA*_Kohn_	RT-015	*tcdA*_R20291_	*tcdB*_630_	*cdtA*_630_+ *cdtB*_630_	*bcrA*_630_	*lmrB*_630_	*vatA*_630_	-	-	-	-
HP-15	-	-	1	I	N/A	*slpA*_Kohn_	N/A	-	-	-	*bcr* *A*_630_	*lmrB*_630_	*vatA*_630_	-	-	-	-
HP-16	-	-	2	I	N/A	*slpA*_79685_	RT-029	*tcdA*_R20291_	*tcdB*_630_	*cdtA*_630_+ *cdtB*_630_	*bcrA*_630_	*lmrB*_630_	*vatA*_630_	-	-	-	-
HP-17	-	-	2	I	N/A	*slpA*_JND09041_	RT-064	*tcdA*_R20291_	*tcdB*_630_	*cdtA*_630_+ *cdtB*_630_	*bcrA*_630_	*lmrB*_630_	*vatA*_630_	-	-	(var)	-
HP-18	-	-	7	I	ST-17	*slpA*_MRY060211_	RT-005	*tcdA*_R20291_	*tcdB*_630_	*cdtA*_630_+ *cdtB*_630_	*bcrA*_630_	*lmrB*_630_	*vatA*_630_	-	-	(var)	-
HP-19	-	-	4	I	N/A	*slpA*_J9952_ trunc.	RT-013, RT-087	*tcdA*_R20291_	*tcd* *B*_630_	-	*bcrA*_630_	*lmrB*_630_	*vatA*_630_	-	-	-	-
HP-20	-	-	9	I	ST-08	*slpA*_R12884_	RT-005, RT-045, RT-054	*tcdA*_R20291_	*tcdB*_630_	*cdtA*_630_+ *cdtB*_630_	*bcrA*_630_	*lmrB*_630_	*vatA*_630_	-	-	-	-
HP-21	-	-	2	I	N/A	*slpA*_R13711_	RT-031	-	-	-	*bcrA*_630_	*lmrB*_630_	*vatA*_630_	-			-
HP-22	-	-	1	I	N/A	*slpA*_R13711_	N/A	*tcdA*_R20291_	*tcdB*_630_	*cdtA*_630_+ *cdtB*_630_	*bcrA*_630_	*lmrB*_630_	*vatA*_630_	-	pos	pos	pos
HP-23	-	-	1	I	ST-54	*slpA*_R13711_	RT-012	*tcdA*_R20291_	*tcdB*_630_	*cdtA*_630_+ *cdtB*_630_	*bcrA*_630_	*lmrB*_630_	*vatA*_630_	-	pos	pos	pos
HP-24	Strain 630 (AM180355)	Strain 6534* (ADEJ)	18	I	ST-54	*slpA*_630_	RT-012	*tcdA*_R20291_	*tcdB*_630_	*c* *dtA*_630_+ *cdtB*_630_	*bcrA*_630_	*lmrB*_630_	*vatA*_630_	(var)	(var)	(var)	pos
HP-25	-	-	7	I	ST-35	*slpA*_JND08037_	RT-046	*tcdA*_R20291_	*tcdB*_630_	-	*b* *crA*_630_	*lmrB*_630_	*vatA*_630_	pos	pos	pos	pos
HP-26	-	-	2	I	N/A	*slpA*_1446_	RT-039	-	-	-	*bcrA*_630_	*lmrB*_630_	*vatA*_630_	-	pos	pos	pos
HP-27	-	-	2	I	N/A	*slpA*_R13700_	RT-010	-	-	-	*bcrA*_630_	*lmrB*_630_	*vatA*_630_	-	pos	-	-
HP-28	-	Strain 6407* (ADEH)	-	I	ST-58 or related (2 loci incomplete)*	*slpA*_6407_*	N/A	*tcdA*_CF5_*	*tcdB*_630_*	-*	*bcrA*_630*_	*lmr* *B*_630_*	*vatA*_630_*	-*	-*	-*	-*
HP-29	-	-	2	I	N/A	*slpA*_6407_	RT-071	-	-	-	*bcrA*_630_	*lmrB*_630_	*vatA*_630_	-	-	-	-
HP-30	-	-	5	I	ST-09	*slpA* negative (probe-1164 sometimes ambiguous)	RT-029, RT-081, RT-094	*tcdA*_R20291_	*tcdB*_630_	-	*bcrA*_630_	*lmrB*_630_	*vatA*_630_	-	(var)	-	-
HP-31	CD196 (FN538970)	BI1*, (FN668941), CIP107932*, (ABKK), QCD-76w55*, (ABHE), QCD-97b34*, (ABHF)	2	II	ST-01*	*slpA*_R20291_	RT-027	*tcdA*_R20291_	*tcdB*_R20291_	*cdtA*_R20291_+ *cdtB*_R20291_	*bcrA*_630_	*lmrB*_630_	*vatA*_630_	-	-	-	-
HP-32	R20291 (FN545816)	Strain 2,007,855* (FN665654), QCD-32 g58*, (AAML), QCD-37x79*, (ABHG), QCD-66c26*, (ABFD)	-	II	ST-01*	*slp* *A*_R20291_*	RT-027	*tcdA*_R20291_*	*tcdB*_R20291_*	*cdtA*_R20291_+ *cdtB*_R20291_	*bcrA*_630_*	*lmrB*_630_*	*vatA*_630_*	-*	(var)*	-	pos*
HP-33	-	-	2	III	N/A	*slpA*_R12884_	RT-023	*tcdA*_R20291_	*tcdB*_630_	*c* *dtA*_Clade III_+ cdtB_R20291_	-	*lmrB*_630_	*vatA*_630_	-	(var)	-	-
HP-34	-	-	1	III	N/A	*slpA*_R12884_ trunc.	N/A	*tcdA*_R20291_	*tcdB*_630_	*cdtA*_Clade III_+ *cdtB*_R20291_	-	*lmrB*_630_	*vatA*_630_	-	-	-	-
HP-35	CF5, (FN665652)	002-P50-2011* (AGAA), 050-P50-2011* (AGAB), M68*, (FN668375)	1	IV	ST-37*, ST-86*	*slpA*_CF5_	RT-017	*tcdA*_CF5_	*tcdB*_CF5_	-	*bcrA*_CF5_	*lmrB*_630_	*vatA*_630_	-	(var)	(var)	pos
HP-36	-	-	1	IV	N/A	*slpA*_79685_	RT-017	*tcdA*_CF5_	*tcdB*_CF5_	-	*bcrA*_CF5_	*lmrB*_630_	*vatA*_630_	-	-	-	-
HP-37	Strain M120, (FN665653)	NAP07*, (ADVM), NAP08*, (ADNX)	8	V	ST11	*slpA*_R13540_	RT-078	*tcdA*_R20291_	*tcdB*_630_	*cdtA*_R20291_+ *c* *dtB*_M120_	*bcrA*_NAP07_	*lmrB*_NAP07_	*vatA*_NAP07_	-	(var)	(var)	-
HP-38	-	Strain 6466 *, (ADDE)	-	V	ST-11*, (1 mismatch)	*slpA*_R13540_*	N/A	*tcdA*_R20291_*	*tcdB*_630_*	*cdtA*_R20291_+ *cdtB*_M120_*	*bcrA*_NAP07_*	*lmrB*_NAP07_*	*vatA*_NAP07_*	-*	-*	pos*	pos*
HP-39	-	QCD-23 m63*, (ABKL)	2	V	ST-11*, (1 mismatch)	*slpA*_23m63_	N/A	*tcdA*_R20291_	*tcdB*_630_	*cdtA*_R20291_+ *cdtB*_M120_	*bcrA*_NAP07_	*lmrB*_NAP07_	*vatA*_NAP07_	-	-	-	-
HP-40	-	Strain 6503*, (ADEI)	-	“VI”	ST127 dlv*	*slpA*_6503_*	N/A	-*	-*	-*	*bcrA*_CF5_*	*lmrB*_630_*	*vatA*_630_*	-*	-*	-*	-*

Full hybridization profiles are provided as Additional file 2

Asterisk indicates *in silico* analysis only

Isolates were clustered into hybridization profiles (HP) or strains based on overall hybridization profiles with emphasis to *tcdA/B* and *slpA* alleles. Isolates or strains were regarded as one HP in case of at least 88 % identity of positive/ambiguous/negative classifications for all probe positions covered, plus presence of identical *tcdA/B* and *slpA* alleles. Possibly mobile resistance markers were counted for the score, but they were, contrarily to *tcdA/B *and *slpA,* not considered for the definition of hybridization profiles or strains. It still needs to be clarified whether these genes could be used as subtyping markers for isolates within one HP (*i.e*., for outbreak investigations).

**Fig. 1:**
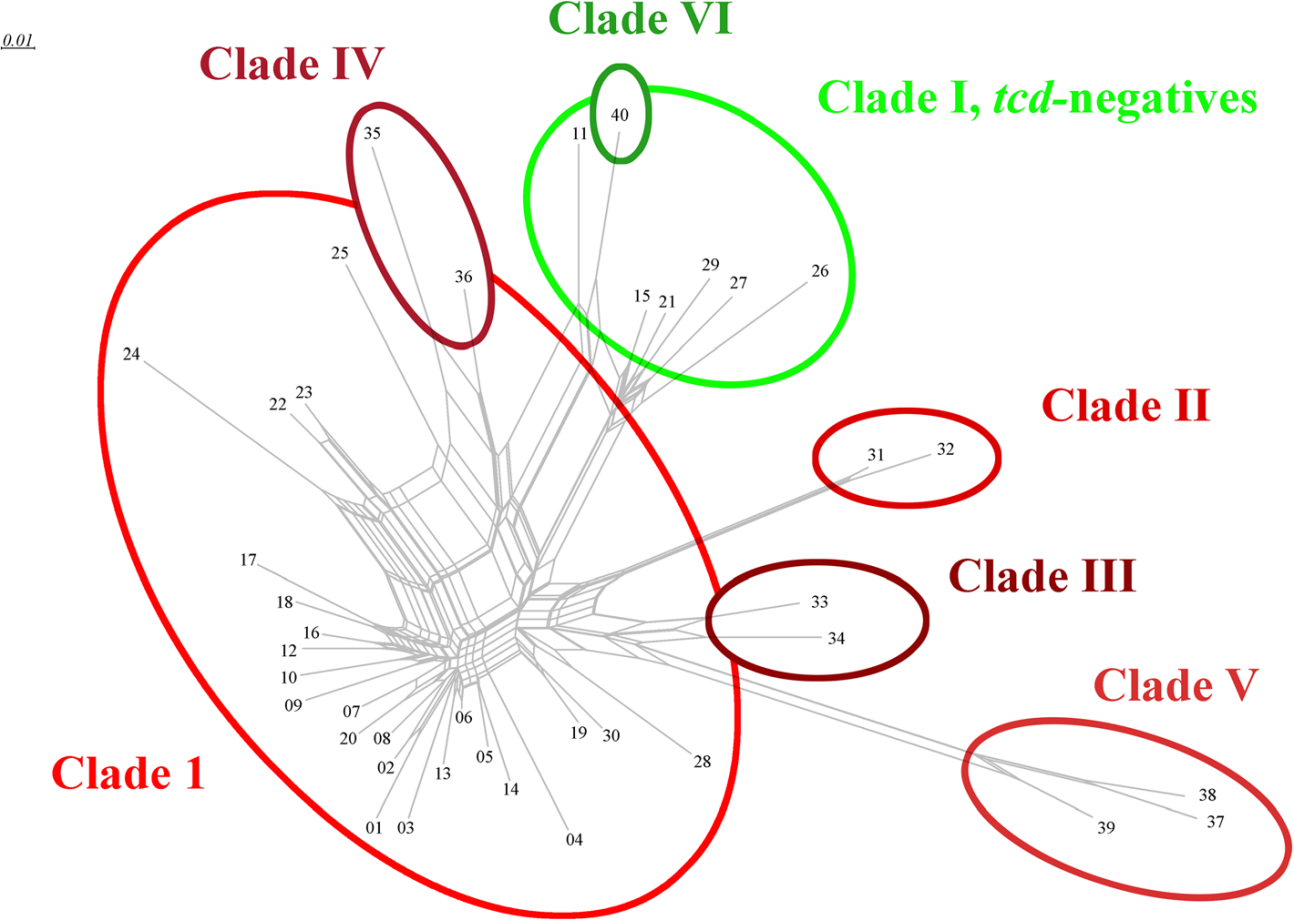
SplitsTree graph based on hybridization profiles, showing the clustering of profiles into different clades as defined by MLST. For the issue of the *tcd*-negatives, see Discussion.

Applying this approach, tested isolates and reference strains clustered into 37 distinct hybridization profiles (HPs; Table 1 and Fig. 1). Three additional profiles were predicted from published genome sequences, although they were not found experimentally. If several isolates with identical hybridization profiles were subjected to MLST, they yielded identical or related sequence types. Occasionally, several ribotypes (RTs) were observed within one cluster and some ribotypes were present in different, although similar or related, clusters.

In *C. difficile*, MLST-derived sequence types (STs) cluster into five major clades [19]. Hybridization profiles also can be clustered into these clades when analyzing their similarities (see Fig. 1).

Clade I encompasses a variety of sequence types including ST-03, ST-45, ST-54 and others [19]. It was found to correspond to the largest and most diverse cluster of hybridization profiles (HP) comprising HP-1 to 30.

Clade II comprised ST-01/RT-027 strains [19]. It matched hybridization profiles 31 and 32. Beside reference strains, only two isolates were assigned to this Clade indicating that the emergence and spread of ST-01/RT-027 strains [20, 21] did not yet engulf the Dresden region at the time when the samples were taken.

Clade III includes ST-05/RT-023 strains [19] corresponding HP-33 and −34. Clade IV consists of ST-37/RT-017 or HP-35 and -36 strains while a Clade V include ST-11/RT-078 corresponding to HP-37 to HP-39. ST-127-like STs might form an additional clade according to eBurst analysis (with ST254 as predicted founder), putatively named “Clade VI” herein. It included the genome sequence of Strain 6503 (GenBank prefix ADEI) which translated into a 40th hybridization profile. It was not identified experimentally.

In the visualization using SplitsTree (see Methods as well as Fig. 1), the *tcdA/B *negative isolates appear to form a separate clade. This, however, can be regarded as an artifact related to the relatively high number of probes recognizing the *tcd *locus (see Discussion).

### Alleles of *slpA*

The gene *slpA* encodes the surface layer protein. Fifty four probes were designed to distinguish *slpA* alleles that are currently represented in GenBank, with one or two probes recognizing one allele. Table 2 shows the predicted patterns and the respective GenBank entries as well as the corresponding ribotyping and/or MLST data for isolates identified within this study. The analysis predicted twenty-eight patterns; twenty-one were found. Additionally, two patterns were observed which probably represent truncated variants of known alleles.

**Table 2:** Alleles of *splA*, corresponding probes, GenBank entries and typing data

*slpA *allele	Reference sequence	Other GenBank entries	Hybridization pattern	Associated ribotypes	Associated clades	Associated sequence types	Associated hybridization profiles
*slpA*_1446_	DQ117219.1		probe-1186 + probe-1201	RT-039	I		HP-22
*slpA*_23m63_	ABKL02000030.1	AB489091.1 (partial), AB236726.1, AB621540.1, AB629936.1, AB675076.1, AF458883.1, AF458884.1, AF458885.1, AHJJ01000092.1, GU230470.1, GU230471.1, DQ117238.1 (partial)	probe-1169 + probe-1170	RT-009	I; V	ST-03, ST11slv*	HP-10, HP-11, HP-39
*slpA*_630_	AM180355.1	ADEJ01000377.1, AF448123.1, AF448124.1, AJ291709.1, DQ060634.1, DQ060635.1, DQ060636.1, DQ060637.1	probe-1166 + probe-1198	RT-012, RT-137, RT-150	I	ST-04, ST-54	HP-04, HP-24
*slpA*_6407_	AB236728.2	ADEH01003569.1, GU230473.1	probe-1167 + probe-1168	RT-011, RT-049, RT-056, RT-071	I	ST-58	HP-03, HP-28, HP-29
*slpA*_6503_	ADEI01000069.1	-	probe-1164 + probe-1197	-	“VI”	ST-127dlv	HP-40
*slpA*_6503_ trunc.	-	-	probe-1164	RT-029, RT-081, RT-094	I	ST-09	(HP-30)
*slpA*_79685_	AF448371.1/AB236727.1	AB239685.1; AB239686.1; AB261625.1; DQ117228.1; DQ117239.1AF448372.1, AF448373.1, AY004256.1	(probe-1163) + probe-1188	RT-017, RT-029	I; IV	-	HP-16, HP-36
*slpA*_ATCC43593_	AF458879.1	AF448122.1, AF448121.1	probe-1176 + probe-1236	-	-	-	-
*slpA*_CF5_	FN665652.1	AB236153.1, AB236154.1, AB236155.1, AB236156.1, AB236157.1, AB602320.1, AB704917.1, AB704920.1, AB704921.1, AB704922.1, AF448125.1, AF448126.1, AF448127.1, AGAA01000010.1, AGAB01000015.1, AJ300677.1, DQ060640.1, FN668375.1	probe-1234 + probe-1249	RT-017	IV	ST-37, ST-86	HP-35
*slpA*_DJNS05008_	AB259786.1	-	probe-1174 + probe-1182	-	I	-	-
*slpA*_DJNS0578_	AB258983.1	-	probe-1199 + probe-1200	RT-163	I	-	HP-05
*slpA*_HR02_	AB236725.1	-	probe-1171 + probe-1237	-	-	-	-
*slpA*_J9952_	AB232929.1	-	probe-1175 + probe-1195	-	-	-	-
*slpA*_J9952_ trunc.	-	-	probe-1175	RT-013, RT-087	I	-	HP-19
*slpA*_JND08037_	AB465011.2	AB259787.1	probe-1173 + probe-1243	RT-046	I	ST-35	HP-25
*slpA*_JND08162_	AB533281.1	AB258978.1, AB258979.1, AB258980.1	probe-1193 + probe-1202	RT-103	I	-	HP-12
*s* *lpA*_JND08232_	AB621541.1	-	probe-1184 + probe-1211	-	-	-	-
*slpA*_JND09041_	AB602321.1	-	probe-1205 + probe-1206	RT-064	I	-	HP-17
*slpA*_Kohn_	AF448119.1	-	probe-1158 + probe-1183	RT-015	I	-	HP-14, HP-15
*slpA*_MRY060211_	AB256018.1	AB180242.1, AB181350.1, AB181351.1, AB453824.1, AB510162.1, GU230474.1, GU230475.1	probe-1178 + probe-1204	RT-005	I	ST-17	HP-18
*slpA*_OG45_	AB231584.1	-	probe-1208	-	-	-	-
*slpA*_R12884_	DQ060_630_.1	AF458877.1, AF458878.1, AF478570.1, DQ060631.1, AB259785.1 (partial), AB518670.1 (partial), DQ060632.1	probe-1156 + probe-1203	RT-005, RT-023, RT-045, RT-054	I; III	ST-08	HP-20, HP-33
*slpA*_R12884_ trunc.	-	-	probe-1156	RT-054	I; III	-	HP-07, HP-34
*slpA*_R12885_	DQ060638.1	AB231583.2, AB257281.1, AB257282.1, AB534595.1, AB534596.1, AB534597.1, AB704918.1, AB704919.1, AF448365.1, AF448366.1, AF448367.1, AGAC01000036.1, DQ060639.1, DQ117221.1, DQ117224.1, FM160740.1, GU230469.1	probe-1209 + probe-1210	RT-014, RT-049	I	ST-42	HP-13
*slpA*_R13540_	DQ060643.1	AB470267.1, ADDE01000013.1, ADNX01000091.1, ADVM01000007.1, AF448120.1, FN665653.1	probe-1177+ probe-1233	RT-078	V	ST-11 slv	HP-37, HP-38
*slpA*_R13541_	DQ060628.1	DQ060629.1 AB240196.1; AB257283.1; AB257284.1	(probe-1155) + probe-1191	RT-002, RT-159	I	ST-08	HP-09
*slpA*_R13700_	DQ060633.1	AF458880.1, AF458881.1, AF458882.1, AF478571.1	probe-1154 + probe-1192	RT-010	I		HP-27
*slpA*_R13711_	DQ060641.1	AB258981.1, AB258982.1, AB518669.1, AF448368.1, AF448369.1, AF448370.1, DQ060642.1	probe-1232 + probe-1239	RT-012, RT-031, RT-057, RT-070, RT-094	I	ST-54, ST-55	HP-08, HP-21, HP-22, HP-23
*slpA*_BI9_	DQ060627.1/ FN668944.1	AB249984.1, AB249985.1, AB257287.1, AB302932.1, ABHD02000026.1, ABKJ02000019.1, AF448128.1, AF448129.1, AJ300676.1, DQ060625.1, DQ060626.1, DQ117225.1, DQ117231.1, FN668944.1	probe-1151 + probe-1190	RT-001, RT-013, RT-015, RT-072, RT-087	I	ST-03, ST-45, ST-46	HP-01, HP-02
*slpA*_R20291_	FM160739.1	ABKK02000030.1, AAML04000014.1, AB249986.1, AB257285.1, AB257286.1, AB269264.1, AB461839.1, AB461840.1, ABFD02000011.1, ABHE02000032.1, ABHF02000035.1, ABHG02000023.1, FN538970.1, FN545816.1, FN665654.1, FN668941.1	probe-1150 + probe-1153	RT-027	II	ST-01	HP-31, HP-32
*slpA*_Y5_	AB538230.1	GU230472.1, AB269265.1	probe-1180 + probe-1196	-	-	-	-
*splA* negative	-	-	none	RT-081	I	-	HP-06, (HP-30)

Five isolates (2.1 %) yielded no positive* slpA* signals. Based on their overall hybridization profiles they clustered into two distinct Clade I strains (HP-06,–30). However in HP-30, ambiguous signals for one probe were observed which might indicate the presence of a truncated variant or divergent allele.

There was no direct correlation of *slpA* alleles, ribotyping and MLST, with isolates of some ribotypes or STs yielding different *slpA* alleles.

### Alleles of *tcdA/tcdB*

Four probes allowed distinguishing two *tcdA* alleles. Both alleles, *tcdA*_R20291_ and *tcdA*_CF5_, were found in this study; with the former one being more common and being detected in more diverse lineages. Table 3 shows corresponding GenBank entries, HPs, RTs, MLST types and *slpA* types. Nineteen isolates were *tcdA*-negative.

**Table 3:** Alleles of *tcdA*, corresponding probes, GenBank entries and typing data

*tcdA *allele	Reference sequence	Other GenBank entries	Hybridization pattern	Associated* slpA* alleles	Associated ribotypes	Associated clades	Associated sequence types	Associated hybridization profiles
*tcdA*_R20291_	FN545816.1	AAML04000007.1, ABFD02000006.1, ABHD02000008.1, ABHE02000016.1, ABHF02000018.1, ABHG02000011.1, ABKJ02000013.1, ABKK02000013.1, ABKL02000008.1, ADDE01000337.1, ADNX01000011.1, ADVM01000023.1, AGAC01000012.1, AM180355.1, AY238985.1, FN538970.1, FN665653.1, FN665654.1, FN668941.1, FN668944.1, M30307.1, X51797.1, X92982.1	probe-1132+ probe-1134+ probe-1135+ probe-1247	*slpA*_23m63_,* slpA*_79685_, *slpA*_DJNS0578_, *slpA*_JND08037_, *slpA*_JND08162_, *slpA*_JND09041_, *slpA*_MRY060211_, *slpA*_R12885_, *slpA*_R13540_, *slpA*_R13711_, *slpA*_630_, *s* *lpA*_6407_, *slpA*_6503_ trunc., *slpA*_BI9_, *slpA*_J9952_ trunc., *slpA*_Kohn_, *slpA*_R12884_ trunc., *slpA*_R12884_, *slpA*_R13541_, *slpA*_R20291_, *slpA*-negatives	RT-001, RT-002, RT-005, RT-009, RT-011, RT-012, RT-013, RT-014, RT-015, RT-023, RT-027, RT-029, RT-031, RT-045, RT-046, RT-049, RT-054, RT-056, RT-057, RT-064, RT-070, RT-071, RT-072, RT-078, RT-081, RT-087, RT-094, RT-103, RT-137, RT-150, RT-159, RT-163	I, II, III, V	ST-01, ST-03, ST-04, ST-08, ST-09, ST-11, ST-17, ST-35, ST-42, ST-45, ST-46, ST-54, ST-55, ST-58	HP-01, HP-02, HP-03, HP-04, HP-05, HP-06, HP-07, HP-08, HP-09, HP-10, HP-12, HP-13, HP-14, HP-16, HP-17, HP-18, HP-19, HP-20, HP-22, HP-23, HP-24, HP-25, HP-30, HP-31, HP-32, HP-33, HP-34, HP-37, HP-38, HP-39
*tcdA*_CF5_	FN665652.1	AB012304.1, AF217291.1, AGAA01000013.1, AGAB01000024.1, FN668375.1, Y12616.1	probe-1132+ probe-1135+ probe-1247	*slpA*_6407,_ *slpA*_CF5_, *slpA*_79685_	RT-17	I, IV	ST-37, ST-86	HP-28, HP-35, HP-36
*tcdA *negative	-	-	none	*slpA*_1446_, *slpA*_23m63_, *slpA*_R13711_,* slpA*_6407_, *s* *lpA*_6503_, *slpA*_Kohn_, *slpA*_R13700_	RT-009, RT-010, RT-011, RT-012, RT-015, RT-031, RT-039, RT-049, RT-056, RT-057, RT-070, RT-071, RT-094	I, ”VI”	ST-03, ST-54, ST-55, ST-58, ST-127dlv	HP-11, HP-15, HP-21, HP-26, HP-27, HP-29, HP-40

For *tcdB*, seven alleles were distinguished using nine probes (Table 4), but only three, *tcdB*_R20291_, *tcdB*_630_ and* tcdB*_CF5_, were experimentally identified. Allele *tcdB*_630_ was the most common and widespread one. Nineteen isolates were negative for *tcdB*; its absence correlated with the absence of *tcdA*.

**Table 4:** Alleles of* tcdB*, corresponding probes, GenBank entries and typing data

*tcdB *allele	Reference sequence	Other GenBank entries	Hybridization pattern	Associated *slpA* alleles	Associated ribotypes	Associated clades	Associated sequence types	Associated hybridization profiles
*tcdB*_630_	AM180355.1	ABHD02000008.1, ABKJ02000013.1, ABKL02000008.1, ADEJ01000447.1, ADNX01000011.1, ADVM01000023.1, AGAC01000012.1, AM180355.1, FN665653.1, FN668944.1, HM062501.1, HM062503.1, HM062505.1, HM062506.1, HM062507.1, HM062508.1, X53138.1, X92982	probe1119+ probe1122+ probe1129	*slpA*_23m63_, *slpA*_79685_, *slpA*_DJNS0578_, *slpA*_JND08037_, *slpA*_JND08162_, *slpA*_JND09041_, *slpA*_MRY060211_, *slpA*_R12885_, *slpA*_R13540_, *slpA*_R13711_, *slpA*_630,_ *slpA*_6407_, *slpA*_6503_ trunc., *slpA*_BI9_, *slpA*_J9952_ trunc., *slpA*_Kohn_, *slpA*_R12884_ trunc., *slpA*_R12884_, *slpA*_R13541_, *slpA*-negatives	RT-001, RT-002, RT-005, RT-009, RT-011, RT-012, RT-013, RT-014, RT-015, RT-023, RT-029, RT-031, RT-045, RT-046, RT-049, RT-054, RT-056, RT-057, RT-064, RT-070, RT-071, RT-072, RT-078, RT-081, RT-087, RT-094, RT-103, RT-137, RT-150, RT-159, RT-163	I, III, V	ST-03, ST-04, ST-08, ST-09, ST-11, ST-17, ST-35, ST-42, ST-45, ST-46, ST-54, ST-55, ST-58	HP-01, HP-02, HP-03, HP-04, HP-05, HP-06, HP-07, HP-08, HP-09, HP-10, HP-12, HP-13, HP-14, HP-16, HP-17, HP-18, HP-19, HP-20, HP-22, HP-23, HP-24, HP-25, HP-30, HP-33, HP-34, HP-37, HP-38, HP-39
*tcdB*_R20291_	FN545816.1	AAML04000007.1, ABFD02000006.1, ABHE02000016.1, ABHF02000018.1, ABHG02000011.1, ABKK02000013.1, FN538970.1, FN545816.1, FN665654.1, FN668941.1, HM062498.1, HM062509.1, HM062510.1	probe1119+ probe1121+ (probe1126) + probe1130	*slpA*_R20291_	RT-027	II	ST-01	HP-31, HP-32
*tcdB*_CF5_	FN665652.1	AF217292.1, AGAA01000013.1, AGAB01000024.1, FN668375.1, HM062499.1, Z23277.1	probe1118+ probe1122+ probe1127+ probe1129	*slpA*_CF5_,* slpA*_79685_	RT-017	IV	ST-37, ST-86	HP-35, HP-36
*tcdB*_51680_	HM062504.1	-	probe1118+ probe1121+ probe1126+ probe1127+ probe1130	-	-	-	-	-
*tcdB*_8864_	AJ011301.1	HM062500.1	probe1118+ probe1121+ probe1124+ probe1127+ probe1130	-	-	-	-	-
*tcdB*_R9385/R10870_	HM062497.1 HM062502.1	-	probe1118+ probe1121+ (probe1126) + probe1130	-	-	-	-	-
*tcdB*_SE844_	HM062511.1	-	probe1119+ probe1121+ probe1129	-	-	-	-	-
*tcdB* negative	-	-	none	*slpA*_1446_, *slpA*_23m63_, *slpA*_R13711_, *slpA*_6407_, *slpA*_6503_,*slpA*_Kohn_, *slpA*_R13700_	RT-009, RT-010, RT-011, RT-012, RT-015, RT-031, RT-039, RT-049, RT-056, RT-057, RT-070, RT-071, RT-094	I, ”VI”	ST-03, ST-54, ST-55, ST-58, ST-127dlv	HP-11, HP-15, HP-21, HP-26, HP-27, HP-29, HP-40

Note, ADDE01000319.1, ADDE01000337.1, ADEH01001038.1, ADEH01001419.1, ADEH01001594.1, AJ002558.1, AJ294944.1, AY238986.1, AY238987.1, DQ683724.1, X60984.1 were excluded from analysis because these were partial sequences only that did not cover all probe binding sites

Co-localized genes *tcdC* and *tcdE* were interrogated with one probe each. They were absent from all *tcdA/B*-negative strains, but frequently they yielded also in other isolates negative or ambiguous results. This might be attributed to sub-optimal binding conditions for these individual probes, un-appreciated sequence variation or to a technical problem during probe synthesis, and should in future be overcome by re-design.

### Binary toxin

Two alleles of the A component (*cdtA*_R20291_ and *cdtA*_630_) of the Binary Toxin were theoretically predicted from published sequences as well as experimentally identified with four different oligonucleotide probes. Isolates of RT-023/MLST Clade III yielded an additional pattern for which no matching GenBank entry was identified. It is putatively named “*cdtA*_Clade III_” in Tables 1 and 5. For the B component (*cdtB*), three alleles (*cdtB*_M120_, *cdtB*_R20291_ and *cdtB*_630_) were distinguishable with six probes.

**Table 5:** Alleles of the Binary Toxin, corresponding probes, GenBank entries and typing data

*cdtA/B *alleles	Reference sequences	Other GenBank entries	Hybridization pattern for *cdtA*	Hybridization pattern for *cdtB*	Associated *slpA *alleles	Associated ribotypes	Associated clades	Associated sequence types	Associated hybridization profiles
*cdtA*_630_ +*cdtB*_630_	AM180355.1	ABHD02000025.1, ABKJ02000018.1, ADEJ01000391.1, AGAC01000133.1, AM180355.1, AY341253.1	probe-1023 + probe-1026	(probe-1038) + probe-1039	*slpA*_23m63_, *slpA*_79685_, *slpA*_DJNS0578_, *slpA*_JND08162_, *slpA*_JND09041_, *slpA*_MRY060211_, *slpA*_R12885_, *slpA*_R13711_, *slpA*_630_, *slpA*_6407_, *slpA*_BI9_, *slpA*_Kohn_, *slpA*_R12884_, *slpA*_R12884_ trunc., *slpA*_R13541_, *slpA*-negatives	RT-001, RT-002, RT-005, RT-009, RT-011, RT-012, RT-013, RT-014, RT-015, RT-029, RT-031, RT-045, RT-049, RT-054, RT-056, RT-057, RT-064, RT-070, RT-071, RT-072, RT-087, RT-094, RT-103, RT-137, RT-150, RT-159, RT-163	I	ST-03, ST-04, ST-08, ST-17, ST-42, ST-45, ST-46, ST-54, ST-55, ST-58	HP-01, HP-02, HP-03, HP-04, HP-05, HP-06, HP-07, HP-08, HP-09, HP-10, HP-12, HP-13, HP-14, HP-22, HP-23, HP-24
*cdtA*_R20291_ + *cdtB*_R20291_	FN545816.1	AAML04000014.1, ABFD02000010.1, ABHE02000029.1, ABHF02000033.1, ABHG02000020.1, ABKK02000028.1, AF271719.1, EF581852.1, FN538970.1, FN665654.1, FN668941.1, HQ639670.1, HQ639671.1, HQ639672.1, HQ639673.1, HQ639675.1, HQ639676.1, HQ639677.1, HQ639678.1, L76081.2	probe-1026 + probe-1027 + probe-1029	probe-1031 + probe-1038 + (probe-1039) + probe-1040	*slpA*_R20291_	RT-027	II	ST-01	HP-31, HP-32
*cdtA*_Clade III_+ *cdtB*_R20291_	-	-	probe-1027 + probe-1029	(probe-1031) + probe-1038 + probe-1039 + (probe-1040)	*slpA*_R12884_, *slpA*_R12884_ trunc.,	RT-023	III	N/A	HP-33, HP-34
*cdtA*_R20291_ + *cdtB*_M120_	FN665653.1	ABKL02000028.1, ADDE01000043.1, ADNX01000028.1, ADVM01000026.1, HQ639674.1, HQ639679.1	probe-1026 + probe-1027 + probe-1029	probe-1030 + probe-1038 + (probe-1039) + probe-1041	*slpA*_23m63_, *slpA*_R13540_	RT-078	V	ST-11	HP-37, HP-38, HP-39
*cdtA/B *negative	-	-	-	-	*slpA*_1446_, *slpA*_R13711_, *slpA*_6407_, *slpA*_79685_, *slpA*_CF5_, *slpA*_JND08037_, *slpA*_6503_, *slpA*_6503_ trunc., *slpA*_J9952_ trunc., *slpA*_Kohn_, *slpA*_R13700_, *slpA*-negatives	RT-010, RT-011, RT-012, RT-013, RT-015, RT-017, RT-029, RT-039, RT-046, RT-049, RT-056, RT-071, RT-081, RT-087, RT-094	I, IV, ”VI”	ST-09, ST-35, ST-37, ST-54, ST-58, ST-86, ST-127dlv	HP-04, HP-11, HP-15, HP-19, HP-21, HP-25, HP-26, HP-27, HP-28, HP-29, HP-30, HP-35, HP-36

The variant *cdtA*_630_ + *cdtB*_630_ was the most ubiquitous one in accordance to the predominance of Clade 1, although some isolates completely lacked *cdtA/B*. In Clade 1 isolates, ambiguous signals were frequently detected apparently due to a poor performance of two probes (as discussed above for *tcdC* and *tcdE*). Clade II strains harbored a distinct variant, *cdtA*_R20291_ + *cdtB*_R20291._ Isolates of RT-023 or MLST Clade III yielded “*cdtA*_Clade III_” while *cdtB* signals in these isolates were indistinguishable from the *cdtB*_R20291_ allele. Clade V isolates carried *cdtA*_R20291_ and a characteristic *cdtB* allele, *cdtB*_M120._ Finally, no *cdtA/B* was detected in Clade IV isolates, and a “Clade VI” genome sequence (Strain 6503, ADEI) did also not include these genes.

### Ubiquitous resistance markers

The gene *bcrA*, encoding the bacitracin ATP binding cassette transporter BcrA, was present in all *C. difficile* isolates but four. Three probes could be used to identify three different alleles.

Allele *bcrA*_630_ (GenBank AM180355.1; 767,494 to 768,420;probe 1072) was present in all Clade I and Clade II isolates. Clade V isolates carried allele* bcrA*_NAP07_ (GenBank ADVM01000079.1; 10,507 to 11,100;probes 1071 and 1073). Clade IV and VI harbor *bcrA*_CF5_ (GenBank FN665652.1; 715,979 to 716,905)which also yielded a signal with probe 1071 while the binding site of 1073 was more similar to the equivalent site in *bcrA*_630_ (differing in one base from *bcrA*_630_ but in five from *bcrA*_NAP07_). Three tested Clade III isolates appeared *bcrA*-negative. Since no published genome sequence was available for that clade, it is not clear whether this lineage lacks the gene entirely, or harbors an unknown allele.

The gene *lmrB*, associated with lincomycin/clindamycin resistance was detected in all tested isolates, and in all published genome sequences analyzed. Two probes were used to identify two different alleles. Allele *lmrB*_630_ (GenBank AM180355.1; 2,893,512 to 2,894,912), was detected in the vast majority of isolates. In isolates associated with Clade V, another allele,* lmrB*_NAP07_ (GenBank ADVM01000028.1; 28,036 to 29,436) was found.

Likewise, *vatA* (synonym *sat*) encoding a virginiamycin/streptogramin A acetyltransferase was found ubiquitously, in tested isolates as well as in analyzed genome sequences. Two alleles were differentiated using two probes, *vatA*_NAP07_ (GenBank ADVM01000028.1; 23 to 655) in Clade V isolates and *vatA*_630_ (AM180355.1; 2,576,453 to 2,577,085) in all others.

### Variable/mobile resistance markers

The presence of *cat* (chloramphenicol acetyl transferase), *erm*(B) (RNA methyl-transferase, conferring resistance to macrolides and clindamycin) and *tet*(M), encoding tetracycline resistance, was variable. The gene *cat *was found in 18 isolates (*i.e*., in 7.5 % of tested strains and isolates). The gene *erm*(B) was detected in two reference strains, BI-9 and 630, as well as in 78 isolates (30 %). *tet*(M) was present in two reference strains, M120 and 630, and in 33 isolates (14.6 %). Carriage rates within *C. difficile *strains were ranging widely, with isolates of certain hybridization profiles (e.g., HP-25 to -27) being virtually always positive for *erm*(B) and/or *tet*(M).

For *tet*(M), five probes reacted in different combinations (Additional file 2). An assignment to alleles was not performed because of several possible sources for error. These might include i) a simultaneous presence of different plasmids in one strain, ii) the existence of chimeric forms (for instance, 5′-and 3′-ends in AJ973139.1, AJ973141.1 and FN665653.1 are identical to ADNX01000070.1 while the middle parts are identical to AM180355.1) and iii) possible irregular patterns for low-copy number plasmids with an effective target concentration around the detection limit of the linear amplification procedure.

### Other markers

Two genes,* vncS/vexP*1 encoding a histidine kinase and a permease were found to always occur together. Some similar strains (*e.g*., HP-31 and-32, or HP-35 and -36) could be distinguished by their presence or absence.

Several other markers contributed to specific profile showing different alleles that were uniform within a HP but could vary within a clade (Additional file 2). These included genes encoding septum formation initiation protein (*divC*), flagellin subunit C (*fliC*), cell wall proteins 66 and 84.

## Discussion

A rapid, reproducible and convenient method for molecular typing of *C. difficile* was developed. It based on a linear multiplex amplification followed by array hybridization. Target genes were resistance genes localized in published *C. difficile* genome sequences and toxin genes with their different alleles. In addition to these markers, other genes were selected based on the variability of their presence (*e.g*., *vncS/vexP*1) or their sequence (*divC, fliC, bcrA, lmrB, vatA*, genes encoding cell wall proteins 66 and 84). Alone these genes would not be suitable typing markers but taken together, they can be used to generate stable profiles or fingerprints that allow assignment to clusters or clades as defined by other methods.

Genes that show clade-specific allelic variations also include the toxin genes. Therefore, a topic for a future study could be a possible correlation of toxin alleles and/or of clonal complex affiliations to clinical severity. In order to check whether a possible higher virulence is caused by the actual toxin alleles, or by some other factor linked to phylogenetic background, a high number of isolates from defined conditions need to be typed and their toxin alleles need to be determined. The proposed system might be a suitable platform for such a task.

It can be assumed that ribotyping, *slpA* typing, MLST and array hybridization yield comparable phylogenetic information,* i.e.*, strains that are recognized as similar/related by one method will also appear as similar/related by the other methods. However, there is no complete correlation. One ribotype might be associated with two similar array profiles or related MLST types and* vice versa*. Single and multilocus typing schemes by design tend to emphasize subtle differences. Isolates that are identical belong by definition to the same ST, but single locus variants, and even those that differ in a single base exchange are defined to belong to another ST. STs are numbered chronologically (*i.e*., by date of submission to the database curator) so that their numbers yield no phylogenetic information. Thus, STs with very different numbers might be still very similar. In order to cluster related STs, clonal complexes (as in, *e.g*., *Staphylococcus aureus*, [22]) or Clades [19] were introduced giving a more structured overview on the phylogeny of the target species. In *C. difficile* there are five major clades, at least one minor clade and several “singletons”, *i.e*., STs that have no known links to others [19].

When converting HPs to a SplitsTree graph, its topology is strikingly similar to a SplitsTree graph of MLST sequences as presented by Dingle et al. [19]. The only significant difference is that all* tcdA/B* negatives are categorized as one “branch”. This is an artifact caused by the high number of probes associated with this locus (n = 15, out of which nine to ten normally are positive). The loss of this locus would thus significantly impact the overall hybridization profile overriding other features affecting a smaller number of probes. Negative results of other markers, such as for* slpA,* would not have this effect because of the smaller number of probes involved.

With regard to practicalities, a major advantage for the array-based approach is that isolate typing as well as toxin gene detection and allele identification can be performed within one experiment by a single amplification reaction starting from clonal colony material. The amplification follows linear kinetics, utilizing one primer per target. This has the advantage of facilitating unlimited “multiplexing”, *i.e*., the simultaneous detection of multiple targets, and of being resistant to contaminations by amplicons from previous experiments. The disadvantage is a reduced sensitivity compared to standard, exponential PCR. However, since the assay was designed to characterize cultured and cloned bacterial cultures (as opposed to native patient samples) this is not of relevance; and sequencing-based typing methods would also lead to nonsensical results when applied to polyclonal samples. In practical terms, protocol and time requirements, including hands-on-time, of the linear amplification are the same as for normal PCR. The subsequent hybridization procedure can be performed within half a day being more rapid than ribotyping. The assay as well as analysis and interpretation can largely be automatized. The set of probes can, possibly combined with MLST markers and *splA *sequences, also be mapped to “conventional” or “next generation” sequence data in order to rapidly obtain clinically relevant typing information out of an abundance of data and to create a database that encompasses both, in silico and in vitro typing data.

## Conclusions

The microarray based assay allows rapid and high-throughput genotyping of clinical *C. difficile *isolates including toxin gene detection and strain assignment. Overall hybridization profiles correlated with MLST-derived clades, and target genes that showed clade-specific allelic variations also included the toxin genes.

## Methods

### Strains and isolates

Completely sequenced strains 630 (GenBank AM180355), BI9 (FN668944), CF5 (FN665652), M120 (FN665653), CD196 (FN538970) and R20291 (FN545816) were used for protocol development and validation. Besides that, 234 clinical isolates were tested. 147 isolates were collected 2007–2009 at the Institute for Medical Microbiology and Hygiene Dresden, Germany (IMMHD; serving the Dresden University Medical Center and a 1000 beds rehabilitation center nearby). Additionally, 80 isolates were contributed by the Institute for Medical Microbiology, University Medical Center Freiburg, Germany and seven by the Friedrich Loeffler Institute Jena, Germany.

### Ethics statement

Isolates were obtained as part of routine diagnostics and were analyzed retrospectively and anonymously. No patient data were used. Ethical approval and informed consent were thus not required.

### Culture and DNA preparation

Isolates were kept frozen at–80 °C using cryobank tubes (Microbank, Pro-Lab Diagnostics, Richmond Hill, Canada). Prior to use they were inoculated on pre-reduced Schaedler haemin-cysteine blood agar and incubated at 37 °C for 48 hours. Then, harvested culture material was transferred into 200 μl Lysis buffer/enzyme mix (A1 + A2; from Alere StaphyType Kit, Alere Technologies, Jena, Germany). After 60 min incubation at 37 °C and 550 rpm, 200 μl AL buffer and 25 μl Proteinase K (from the QIAamp DNA Mini Kit Qiagen, Hilden, Germany) were added and another incubation step of 60 min, at 56 °C and 550 rpm followed. After addition of ethanol, DNA was purified using spin columns (QIAamp DNA Mini Kit Qiagen). Finally, DNA was eluted in 50 μl water and heated for 10 minutes at 85 °C in order to evaporate trace contaminants of ethanol. The DNA concentration was determined spectrophotometrically at 260 nm. If necessary, DNA was concentrated to 150 ng/μl by evaporation.

### Array design

The array was designed to include toxin genes (*tcdA/B, cdtA/B*), genes related to antimicrobial resistance (*cat*, *erm*(B), *tet*(M)), known typing markers (*slpA*) as well as genes for which the analysis of published genome sequences showed either a variable occurrence, or the occurrence of distinct alleles. A complete list of targets and primer/probe sequences is provided in Additional file 1. First, all GenBank entries for any given target were retrieved. One entry was selected as reference, and its coding sequence was excised. All resulting BLAST hits were downloaded and re-annotated into a local database excising and aligning all valid open reading frames. Sequences were classified into paralogues and allelic variants based on similarity. Consensus regions from the alignments were chosen for the probe and primer design. Probe sequences were selected for specificity and for similar GC content, length, and melting temperature. Resulting probe sequences were re-blasted against all available sequences to check for false negativity or cross-reactivity.

One hundred thirty-five probes were spotted in triplicate on arrays that were mounted into ArrayStrips (http://alere-technologies.com/en/products/lab-solutions/platform-components/arraystrip-as.html). The length of the probes ranged from 24 to 34 bases (mean length, 27 bases; median length, 28 bases). There were 140 primers. Their lengths ranged from 18 to 25 bases (mean and median lengths, 20 and 21 bases, respectively).

### Protocol optimization

For validation of the array and for the optimization of the protocol, completely sequenced strains (see above) were used. Hybridization profiles were predicted by comparing the probe sequences with their known genome sequences. Real hybridization experiments were performed stepwise modifying hybridization and washing temperatures until the experiments yielded results that were in accordance to the theoretical predictions (Additional file 2). The resulting protocol is described below.

### Linear DNA amplification and labeling

DNA labeling was performed during the linear amplification step by incorporating dUTP-linked biotin. The master mix consisted of B1 Buffer (3.9 μl/sample; as all buffers and reagents used herein, unless stated otherwise, taken from Alere HybPLUS kit, Alere Technologies), B2 Buffer (0.1 μl/sample) and a primer mix (0.135 μmol/L of each primer and a total of 1.0 μl/sample). Then, 5 μl of the DNA preparation was added. The amplification was carried out using a Mastercycler (Eppendorf GmbH, Hamburg, Germany) with 5 min of initial denaturation at 96 °C, followed by 55 cycles (60 sec at 96 °C, 20 sec at 50 °C and 40 sec at 72 °C).

### Hybridization and detection

Prior to use, each array was subsequently incubated with 200 μl double-distilled water and 200 μl C1 washing buffer (both steps at 50 °C, 5 min and 550 rpm on a BioShake iQ thermomixer; Quantifoil Instruments, Jena, Germany). Then, the biotin-labeled amplicons and 90 μl C1 buffer were pipetted onto the array and hybridized for 60 min at 50 °C and 550 rpm. After removal of the liquid, two washing steps were performed using 200 μl buffer C2 for 10 min at 45 °C and 550 rpm. Horseradish-streptavidin conjugate C3 was diluted 1:100 in C4 buffer; 100 μl was added to the array and incubated for 10 min at 30 °C and 550 rpm. After removal, 200 μl C5 Buffer was added and incubated (5 min, 30 °C, 550 rpm). Finally 100 μl precipitating dye (D1) was pipetted to the array and incubated for 10 min at room temperature. After removal of liquids, the array was photographed and automatically analyzed using a ArrayMate reading device (Alere Technologies). Normalized intensities of the spots were calculated based on their average intensities and local background [18, 23]. For each probe, three spots were spotted and for all further analyses the median of the spot signals was used.

Breakpoint determination relied on the signal intensities for the ubiquitous, species-specific markers (*bacA*1, *bcrA, lmrB, hly*3, *ydiC, spaE*) and the biotin staining control. Because there are several probes for mutually exclusive alleles of some of these markers (*bcrA, lmrB, spaE*), only those probes that gained raw values above 0.2 were considered. The median of signals of these species markers and the biotin control was calculated. Each individual probe on the array that yielded a signal of more than 2/3 of the median was considered positive; and signals between 1/3 and 2/3 of this median were regarded ambiguous. If the median of the species markers and the biotin marker was below 0.6 the entire experiment was regarded invalid. If four or less of these markers gained raw values above 0.2, the entire experiment was regarded invalid, too.

Full hybridization profiles are provided in Additional file 2.

### SplitsTree

In order to visualize similarities, array hybridization profiles (as in Additional file 2) were converted into ‘sequences’ in which each probe position could have a value of ‘positive’, ‘negative’, ‘ambiguous’ or ‘variable’. These ‘sequences’ were used to construct a tree using SplitsTree vers. 4.12.6 [24] on default settings (characters transformation, uncorrected P; distance transformation, Neighbor-Net; and variance, ordinary least squares).

### Additional typing methods

For representative isolates, Multi Locus Sequence Typing (MLST) was performed with a 3130 Genetic Analyzer (Applied Biosystems, Foster City, USA). Primer sequences and reaction conditions were previously described by Griffith *et al*. [14]. Data analysis was performed using the database accessible under http://pubmlst.org/cdifficile/. Ribotyping of representative isolates was performed as previously described [13].

Additional typing data are also provided in Additional file 2.

## Additional files

Additional file 1:
**Probe and primer sequences.** (PDF 42 kb) Additional file 2:
**Full datasets for all tested strains and isolates.** (PDF 437 kb)
