# Supplementary material for: DNA-Microarray-based Genotyping of Clostridium difficile
Source: BMC Microbiol. 2015 Aug 5;15:158. doi: 10.1186/s12866-015-0489-2 (PMC4526300; doi:10.1186/s12866-015-0489-2)
Supplement: Additional file 1: — Probe and primer sequences. (PDF 42 kb) [file 12866_2015_489_MOESM1_ESM.pdf]

PROBES

No	Name	Sequence	Length	GC %	Tm °C
1	hp1012|cwp66	TGGAGTATGGTTACAATATCAAATCAATC	29	31	55.5
2	hp1013|bacA1	AGGTACATCTAGGTCTGGTTCTACTATA	28	39	56.9
3	hp1014|catD	CAACGGTATGGAAACAATCATAGAATG	27	37	56.4
4	hp1016|cwp66	AACAGATGAAGAGCCATTTCTTGAAA	26	35	57
5	hp1018|cwp84	CAACAATACCTGTCTCAGCAGAAA	24	42	56.5
6	hp1020|cwp66	TGGTTAGTAACTATATCTAATGAATCCAGAG	31	32	55.9
7	hp1021|cwp66	TGGATTATAACTATAGATGCTGATGCTAAA	30	30	56.1
8	hp1022|ermB	ACAGTCATCTATTCAACTTATCGTCAGA	28	36	57.4
9	hp1023|cdtA	AGGCTAAAGAATGAGAAAGAAAAGATCAA	29	31	57
10	hp1026|cdtA	TTGTTACCTAATAACATTTATGCACAAGA	29	28	55.6
11	hp1027|cdtA	ACTAGTATTGGTAGTGTGAATATGAGTG	28	36	55.9
12	hp1029|cdtA	TGATTGGAGTAATAAATTAACACCTAATGAAC	32	28	56.4
13	hp1030|cdtB	AAGACATACTCACAAACAACGGATT	25	36	56.5
14	hp1031|cdtB	TGAAGATATACTCCCAAACAATGGATT	27	33	55.9
15	hp1038|cdtB	GAGTTGAAGCTACTTTACAATATTCTGGA	29	34	57
16	hp1039|cdtB	AGAGTTGAAGCTACTTTACAATATTTTGGA	30	30	57.1
17	hp1040|cdtB	CAGCTATAGTTAGTCAAGCACTAGTATATC	30	37	56.4
18	hp1041|cdtB	GCCATAGTTAGTCAAGCATTAGCATA	26	38	56.6
19	hp1043|vanG	ATGGACAAAGACAAATCACATAAACTTG	28	32	56.6
20	hp1044|divIC	TGAAATACAAATAAACAGCTTAAAGAAAGATGA	33	24	56.9
21	hp1045|divIC	AATACAAATAAACGCCTTAAAGAAAGATGA	30	27	56.3
22	hp1046|divIC	CTGAAATACAAATAAACAACTTAAAGAAAGATGA	34	24	56.2
23	hp1051|vncS	ACAGTTGAAATATTGAAAGTATCTTCTTTCC	31	29	56.5
24	hp1052|vncS	AGCTCTAATGATGAAGTTGGAGAATTAA	28	32	56.2
25	hp1055|vexP1	TGGGCAAGGTAATTTCGAGTATAAA	25	36	55.8
26	hp1059|vexP1	AGTAATTGTAGATGGATTCATTAACTCAGA	30	30	56.1
27	hp1061|eutG	CATGCTATAGAAGCTTACGTATCTACAA	28	36	56.3
28	hp1062|eutG	CATGCTATAGAGGCTTATGTATCTACAA	28	36	55.8
29	hp1063|matE	GTGAAATCAAAGCCTATGGCATTAGATA	28	36	57.3
30	hp1071|bcrA	GAATCTTGACTTACATTGTGAATATATGGG	30	33	56.3
31	hp1072|bcrA	GAATCTTGATTTACACTGTGAATATATGGG	30	33	56.3
32	hp1073|bcrA	ATGTAAGAAAGGAAGTCATTTACGGATT	28	32	56.6
33	hp1079|spaK	TTGAAAGAAGGACTATTACGTCATCAAA	28	32	56.6
34	hp1080|spaK	GAGAGATATTGAAGAAAGGCATGCATAT	28	36	57.1
35	hp1082|sat	GACTAGTGGAAATCACTCAATGAAATCA	28	36	57.1
36	hp1083|sat	ACTAGTGGAAATCACACACTGAAATC	26	38	56.8
37	hp1084|CD0456	CCTTGTAGTAGAGCATGATAAAGATGTT	28	36	56.6
38	hp1087|cme	TGTCAAACAATTTATCAACATTAATTTCTTCA	32	22	55.9
39	hp1096|lmrB	AGCGATTAATAGTACACTTCGTCAGATA	28	36	57.3
40	hp1097|lmrB	GCAATTAATAACACACTCCGTCAGATA	27	37	57
41	hp1098|ydiC	AACAGAGGAAAGGCACTAGGAATT	24	42	57.2
42	hp1103|hly3	GAGACCCAATAAGCAGTTTAACTCAT	26	38	56.6
43	hp1105|tetM	ACCGTTATCTTTATCAACAAGATCGAC	27	37	57
44	hp1108|tetM	TGATTTAACTTTCTTTACAAATGGACGT	28	29	55.9
45	hp1109|tetM	ACTTTAACTCTATATACAAATGGGCAAGC	29	34	57.4
46	hp1110|tetM	GAGTAAACCTGAACAGAGAGAAATGTT	27	37	56.8
47	hp1112|tetM	CAAACCTCAACAAAGGGAAATGTTA	25	36	55.7
48	hp1113|tcdE	TGTGGATTACCAGTACCTAAGAGATTAA	28	36	56.6
49	hp1117|tcdC	GAAATAGAAACTTTAAATAGCAAATTGTCTGA	32	25	55.8
50	hp1118|tcdBp1	CTGATTCAGTAAAGACAGCTGTAGATTG	28	39	57.5
51	hp1119|tcdBp1	TCTATAGAGAAACCTAGTTCAGTAACAGT	29	34	56.4
52	hp1121|tcdBp4	AGCTATAGAATGGCAAACATTAGATGAT	28	32	56.4
53	hp1122|tcdBp4	GGAGCTGTAGAATGGAAAGAATTAGATG	28	39	57.3
54	hp1124|tcdBp2	GAACTTATATTACGAGCTGAGGCAAA	26	38	56.8
55	hp1126|tcdBp2	TGAGCTTATCCTAAGAGATAAAGCAAC	27	37	56.3
56	hp1127|tcdBp2	TGATAAGTATAGAATTCCTGATAGAATCTCTG	32	31	56
57	hp1129|tcdBp3	AGAATTAAGTGAAAGTGATGTTTGGATT	28	29	55.6
58	hp1130|tcdBp3	CATTGATGGCAAATTCAAATTCTGTTC	27	33	56.3
59	hp1132|tcdAp1	GTTGATATGCTTCCAGGTATTCACTC	26	42	57.1
60	hp1134|tcdAp2	AGTGATTGTGTAGTGAAAATAGGTGT	26	35	56
61	hp1135|tcdAp3	ACTTTATGCCTGATACTGCTATGG	24	42	55.7
62	hp1141|fliC	AGCAAACAATCAAATGGGAAGAAAC	25	36	56.5
63	hp1142|fliC	AGCAAATAACCAAATGGGAAGAAAC	25	36	56
64	hp1144|fliC	ATCAAGTTCAACAGAGTTTAACGGTAA	27	33	56.7
65	hp1145|fliC	CAGACTCTACTAAATTCAATGGTGAGAA	28	36	56.5
66	hp1147|fliC	CAGGTATAACAAGTTCTACAATAGCAAG	28	36	55.7
67	hp1149|CD2797	GCATTTATGTATGCTTCTGGATTACATG	28	36	56.8
68	hp1150|slpA	CAAATGATCAAGCTGCGACTATTG	24	42	56.4
69	hp1151|slpA	AGATTTCTTAATAACTTCTCCAGGTCAAG	29	34	56.7
70	hp1153|slpA	TTCTTAATAAGCTCTCCAGCAGAGG	25	44	57.4

PROBES

No	Name	Sequence	Length	GC %	Tm °C
71	hp1154|slpA	AACTGATGAAGCTGAATCTGGTTTAG	26	38	56.9
72	hp1155|slpA	ACATATGCATCATCTAACTATAAGCCAG	28	36	56.5
73	hp1156|slpA	CAAGGTTTCTCAACTTATAGAGCTACAA	28	36	56.7
74	hp1158|slpA	ATCAGCTGCACAACCTTCTAAATTA	25	36	56.3
75	hp1163|slpA	GATTAGATACTAAAGGAGCAACAGATATAGA	31	32	55.9
76	hp1164|slpA	ACTAAGAGTGCATCAACTGATATTGAAA	28	32	56.7
77	hp1166|slpA	ATCAGCAAATGATACAATAGCTAGTCAA	28	32	56.5
78	hp1167|slpA	AAGATGGTTCTACTCCTGGTGATG	24	46	57.1
79	hp1168|slpA	AACTATAACTGCAGCTACTTCAGAG	25	40	55.7
80	hp1169|slpA	ATCAACTGGTAATGTGTCAGGGTTAT	26	38	57.5
81	hp1170|slpA	AGCAGATAATGATATTAAAGGTTCTGATTTAT	32	25	55.5
82	hp1171|slpA	TGGTTCTGAGGATGCAGATGTAAA	24	42	57.2
83	hp1173|slpA	ATCAAGATGAAAGTTAATGAAGTTGCTG	28	32	56.5
84	hp1174|slpA	TGATAAACGATAATGAGTACAAAGTAACAAA	31	26	55.9
85	hp1175|slpA	AGTTTATAACTGATGTAGTTCCAGGTGTA	29	34	57.3
86	hp1176|slpA	AGAATATGGAATAAGTGGAACAAAGGTA	28	32	55.9
87	hp1177|slpA	GATACAGAGGCAGTTATATCTACTTCAA	28	36	55.7
88	hp1178|slpA	AAGATAATTCTACAGCTACAGACGTTAC	28	36	56.4
89	hp1180|slpA	ACTAAGAAATCAACTTCAACAAGTCAGA	28	32	56.7
90	hp1182|slpA	CTGTAGCTCCTTCCAAATATAAGCTTT	27	37	56.7
91	hp1183|slpA	CAGGTGATGCTGGTAAATATACATTAGA	28	36	56.4
92	hp1184|slpA	CTTATAATGCTGCAGAGACAATTCATAC	28	36	56.3
93	hp1186|slpA	AGAAATTTCACACTGAGGAAGAAATAGA	28	32	56.3
94	hp1188|slpA	GAGAATAAAGAGATTGTTAAGCCAGCAT	28	36	57.4
95	hp1190|slpA	ATGCTATAATTGCTGGAACATCTTCAG	27	37	57.3
96	hp1191|slpA	TGTAGTTACTAAAGTTGGTACTGCAAC	27	37	56.8
97	hp1192|slpA	CAGCTAATAATGGTAAGATAATAATTGATGCA	32	28	56.4
98	hp1193|slpA	AAGGTTCTATATTCACCAGCAACAAA	26	35	56.5
99	hp1195|slpA	ATGCTAGAGGAACTAGTGAAATAGTAGT	28	36	56.5
100	hp1196|slpA	AGTTGTTGTTGCTGGTTCTACTAATTC	27	37	57.5
101	hp1197|slpA	GATAGAGATTTACTTGCCTCTGATTTCT	28	36	56.3
102	hp1198|slpA	ACAAAGGCACTTAAAGTTAAAGATGTTG	28	32	56.8
103	hp1199|slpA	ATCAAGCGCTGAAATAAGTGGATTT	25	36	57.1
104	hp1200|slpA	GACTTATTAAAGCAGACCAATCACCTAA	28	36	56.9
105	hp1201|slpA	ACAAAGATAAGACAATTGCAACATATGA	28	29	55.8
106	hp1202|slpA	TTGCAACTTTAACAATTGGTTCTGATAA	28	29	56.3
107	hp1203|slpA	GATTCTTCAACTCCAACTGGAGATA	25	40	55.6
108	hp1204|slpA	CCTTAGCAGCTGATACTACATTGAAA	26	38	56.5
109	hp1205|slpA	CGAAAGTAGACGGTAGTGATGTAATTG	27	41	57.3
110	hp1206|slpA	AGATGCTTCTAGTGTTAGTGCCATAA	26	38	57.3
111	hp1208|slpA	CTGTTAAAGATGCAGTAAAGGCTACT	26	38	56.7
112	hp1209|slpA	TGCTTCATTAAGTGCAGATGATATACAA	28	32	56.8
113	hp1210|slpA	CAGGATCTGAAGCAGGTATAGCAA	24	46	57.4
114	hp1211|slpA	TGCTAAATCATCGTTTAAATTTGATGCA	28	29	56.7
115	hp1212|CD2797	GAACAAGAAGAAGTAAATCAAGAAGAAGT	29	31	56
116	hp1214|CD0425	AAGAAATTGACAAGGATATGCAGCTAC	27	37	57.4
117	hp1220|CD0425	AGCTACTGCCCATATTCAATTTATGAAA	28	32	56.9
118	hp1227|IPR007253	TGTTAATAAATGACTCTAGCATATCGGA	28	32	55.7
119	hp1228|CD0425	AAGATCTTAGATAGCAAGATTTCTGCTC	28	36	56.6
120	hp1229|CD0425	AGATTATTGATAAAGAAATGCAGGAGCT	28	32	56.7
121	hp1230|CD0425	AAGGTTATTGATAAGGAAATGCAGGAG	27	37	56.9
122	hp1231|CD0425	AAGGCTATTGATAAGAAAATGCAGAAG	27	33	55.8
123	hp1232|slpA	CAACTAACTATACTTCTGGAGCTGAAATA	29	34	56.3
124	hp1233|slpA	TGCTAATAATGATACACTTGCAGATGTT	28	32	57
125	hp1234|slpA	GACTACAAGTTTATTGTATGGTACGGTT	28	36	56.9
126	hp1236|slpA	GCTACAGCATTTACTGTAACTAATCCAA	28	36	57.2
127	hp1237|slpA	CAGCTGCTAATGATCTTAAAGGATCTG	27	41	57.3
128	hp1239|slpA	ATCAGCTTCTACTCTTAGAGCAAGTATA	28	36	56.5
129	hp1242|slpA	CAACTGATAAAGAGAAGGAAGTAGCAG	27	41	57
130	hp1243|slpA	AACTTAAAGGAACTAAAGGAGAAAGCAC	28	36	57.4
131	hp1244|ydiC	AGGACAAGTTATAGGTATTACTATTTCTACAA	32	28	55.8
132	hp1247|tcdAp2	ACTATAAATGTACTACCTACAATAACAGAGG	31	32	56
133	hp1249|slpA	TGATAAAGTACTTATAAATTCTGCAACAGAT	31	26	55.6
134	hp1251|spaE	TTGTATGCTGGGTTGTTTCTCTTG	24	42	57
135	hp1252|spaE	TTGTATGTTGGGTTGGTTCTCTTG	24	42	56.7

PRIMERS

No	Name	Sequence	Length	GC %	Tm °C
1	lb2053-cwp66	TGTTATGTCTCCATCATTTTCT	22	32	50.4
2	lb2059-cwp66	TCCATCATTTTCTATATGCACA	22	32	50.6
3	lb2060-bacA1	TACCAAGTAATACTGCACCT	20	40	50.6
4	lb2064-cdtA	TTCTATAGGAGCCTTGTAAGT	21	38	50.4
5	lb2065-cdtA	CAGGAGAACCTTTAGGTATAGT	22	41	51.3
6	lb2067-cdtA	GCATATAATCATTTACATCAGCAA	24	29	51.2
7	lb2079-vncS	AGTTCTTTATCGTCTTTTAACTCT	24	29	51.3
8	lb2084-eutG	TTTCAGCTAGTGCATCTGA	19	42	51.1
9	lb2085-matE	GTGTAAAGTTCACAAATAGGGA	22	36	51.3
10	lb2092-bcrA	CACTGTACTTTTACCTGCTC	20	45	51.1
11	lb2099-spaK	CTGGAGAACTATATTCTGATACT	23	35	49.6
12	lb2100-spaK	TGGAGAACTATACTCTGATACT	22	36	49.9
13	lb2101-cwp66	ACAGCTTCTACATAACAACC	20	40	50.3
14	lb2102-cwp66	TCCATCTTTGGCTCTTTCA	19	42	51.2
15	lb2105-spaK	GGAGAAACAAATTCTGCTACT	21	38	51.1
16	lb2106-cwp84	TGCAGTTTCTACTCCATCT	19	42	50.3
17	lb2108-cdtB	AAATCTTTAAAGTGTTCGTCTG	22	32	50.4
18	lb2109-cdtB	AAATCTTTAAAGTGCTCATCTG	22	32	50.1
19	lb2110-divIC	TCTTGCTATATCCTCTAAGTCT	22	36	50.2
20	lb2111-catD	AGCATTTGGCTTTCCTTC	18	44	50.5
21	lb2112-vanG	GGTACTTTAATACCTTCTGAATG	23	35	50
22	lb2113-ermB	CTTGGTGAATTAAAGTGACAC	21	38	50.4
23	lb2114-cdtA	TCTGAGAATATTTGCTTATCTCT	23	30	50
24	lb2115-vexP1	TTCAAGATCAATATTGGACCA	21	33	49.8
25	lb2116-vexP1	CCACCGGAAATCATTGAG	18	50	50.5
26	lb2117-bcrA	ACACATTCCAATACCTCTGA	20	40	50.9
27	lb2119-bcrA	GACACATTCCAATATCTCTGAT	22	36	50.7
28	lb2122-cdtB	TTGTACTAGTTTGAGCATATACA	23	30	50.3
29	lb2123-vncS	TCGTCAATTGTTCTTAATAAAGT	23	26	49.8
30	lb2124-sat	AGATAGGAAAGGTATATGTGGA	22	36	50.4
31	lb2125-sat	GATGGGAAAAGTATATGTGGA	21	38	50
32	lb2126-CD0456	ACTTCATCTGCTATGGAGAT	20	40	50.5
33	lb2128-cme	TGCATTGGAAACTATACCAAG	21	38	51.2
34	lb2132-lmrB	GCAGAACCTATAGCACCA	18	50	50.9
35	lb2133-lmrB	GCAGAACCTATAGCTCCA	18	50	50.3
36	lb2134-ydiC	CATATTTCCAATAGCAACTGC	21	38	50.7
37	lb2136-hly3	AAAAGAGATAAACAAGCACCT	21	33	50.6
38	lb2137-tetM	CTTATCCCGAACAGACTGA	19	47	50.9
39	lb2139-tetM	GTTCACAAGTAGCAATTTGAT	21	33	50.1
40	lb2140-tetM	CCAGTAGTAACATAGTACCCT	21	43	50.5
41	lb2141-tetM	GGTAGTAACATGGTACCCT	19	47	50.1
42	lb2142-tetM	GTCACTGTCGGAGATTTC	18	50	49.9
43	lb2143-tetM	GGATCACTATCTGAGATTTCC	21	43	50.3
44	lb2144-tcdE	AGCATTCATTTCATCTGTCAT	21	33	50.8
45	lb2145-tcdC	TCTTTCATTTTGAACCATGGT	21	33	51.1
46	lb2146-tcdBp1	CTTCTAACTGCATCTCTTCC	20	45	50.5
47	lb2148-tcdBp1	GCTTCTAACTTTGTCATTTCC	21	38	50.5
48	lb2149-tcdBp1	GCTTCTAATTTTACCATTTCCC	22	36	50.8
49	lb2152-tcdBp4	AAGCTTTACCTGTTTCTGG	19	42	50.1
50	lb2154-tcdBp4	AAAGCTCTACCTGTATCTGT	20	40	50.4
51	lb2155-tcdBp4	AAGCTTTGCCTGTTTCTG	18	44	50.8
52	lb2158-tcdBp2	GACTCAGCTAATGATATATGACT	23	35	50.3
53	lb2159-tcdBp2	GATTCAGCTAATGAAATATGGC	22	36	50.8
54	lb2160-tcdBp2	GCCATGACCAATGAATGT	18	44	50.2
55	lb2161-tcdBp2	CTTTGCCATGACCAATAAGT	20	40	51.2
56	lb2162-tcdBp3	TCTCTCACAACATTATCAACA	21	33	49.8
57	lb2163-tcdBp3	TCGCTATTCAATCCTATATAATCT	24	29	50
58	lb2164-tcdBp3	CGCTGTTCAATCCTATATAATCT	23	35	51.2
59	lb2165-tcdAp1	AGTCCAATAGAGCTAGGTC	19	47	49.9
60	lb2168-tcdAp2	CAAATCCATTAGAGGTACTAAAC	23	35	50
61	lb2169-tcdAp2	TCAAATCCATTAGAGCCACT	20	40	51.3
62	lb2171-tcdAp3	ACTCCATCAACACCAAAGA	19	42	51.1
63	lb2175-fliC	TCCATAGACTTACTTTGTCCA	21	38	50.9
64	lb2176-fliC	CCATAGACTTACTTTGTGCA	20	40	50.3
65	lb2178-fliC	TTCTGCTCCAACTTGTAATG	20	40	50.9
66	lb2179-fliC	AGAAGTTCCATCAAGTAGCT	20	40	50.9
67	lb2182-fliC	CTGCATCTGTTCCAGAAG	18	50	50.1
68	lb2183-fliC	TTGTTTAGCTGCATTTGTTC	20	35	50.3
69	lb2185-CD2797	CATTCATAAGCCCTGGAAC	19	47	50.7
70	lb2194-IPR007253	CTTTAGACTTAGCAAATGGTG	21	38	49.9

PRIMERS					
**No**	**Name**	**Sequence**	**Length**	**GC %**	**Tm °C**
71	lb2196-CD0425	ATGAACCTGCTCCATAGTT	19	42	50.6
72	lb2197-CD0425	GAACCTGCTCCATTGTATC	19	47	50.5
73	lb2198-CD0425	GGATGGATTTGCTTTATATTCC	22	36	50.3
74	lb2199-CD0425	AGACGGATTAGCCTTATATTCT	22	36	51.2
75	lb2200-CD0425	AGATGGATTAGCCTTATATTCC	22	36	49.9
76	lb2201-slpA	ATCTATTCAATTCGCCTGAAG	21	38	51.1
77	lb2202-slpA	TGAAATAACTTGCAAATGTTGT	22	27	50.7
78	lb2203-slpA	AGTTTACTTGTAGTTACTTTATCTC	25	28	49.9
79	lb2204-slpA	TGCATTCAATATTCTAAGTTTAGT	24	25	49.8
80	lb2205-slpA	CCTGTTAAATCTTTCAATGCA	21	33	50
81	lb2206-slpA	TGAATTTGTCTCAGTAGAACC	21	38	50.5
82	lb2208-slpA	GTATCAGCAGTTAATGTTAAGC	22	36	50.7
83	lb2210-slpA	ACTAATTTAAGAGCTGCATTTC	22	32	50.2
84	lb2212-slpA	ATCTACTCTATCACTTGTAGGT	22	36	50.1
85	lb2213-slpA	GATTATTTCCCTCAACTTTACC	22	36	49.9
86	lb2215-slpA	GAAGCATTTATTTGATCTGCA	21	33	50.2
87	lb2217-slpA	TCTCCTGCTATAACTTTATTGTT	23	30	50.5
88	lb2218-slpA	GTCTTTACATAATCTTCATTTCCA	24	29	50.4
89	lb2219-slpA	TGTATTACCATATGCAGTTCC	21	38	50.4
90	lb2220-slpA	CCATCACTAGCATCACTAAC	20	45	50.6
91	lb2222-slpA	TTGTTGTAGAAGCAACAGG	19	42	50.5
92	lb2223-slpA	ACAGATATCTTTGCATCATCTT	22	32	50.6
93	lb2224-slpA	CTGGTGGAATTGCTTCAG	18	50	50.7
94	lb2225-slpA	ACAAAGTGGAGAATTGTCAC	20	40	51.1
95	lb2226-slpA	CATCTGCTATAGTCAATGGAG	21	43	50.7
96	lb2227-slpA	TTACTAAGTTTGAAGTAGAAAGTG	24	29	50
97	lb2228-slpA	AAGTGCAACAATTTCTTCTTC	21	33	50.6
98	lb2229-slpA	GTAGATACAGTGGCATTTGAT	21	38	50.7
99	lb2230-slpA	TGCTTATCAACAAGGTTAAGT	21	33	50.4
100	lb2231-slpA	CATCTACAGTTCCTCCTGT	19	47	50.4
101	lb2233-slpA	TGTAGTTAACTCAGCATTTGT	21	33	50.4
102	lb2234-slpA	TCAGGAACTTTAGGGATATCA	21	38	50.3
103	lb2235-slpA	AGTGTTTCTAATTTGTCAGCT	21	33	50.7
104	lb2236-slpA	GTAGAACCACTAGCTTTAGG	20	45	49.8
105	lb2237-slpA	GTAGCTTCTTGTTCTCTAGC	20	45	50.6
106	lb2239-slpA	TGCTACTCCATTAGCTTGA	19	42	50.4
107	lb2240-slpA	AAGTTTGACCAGCTTGAC	18	44	50.1
108	lb2241-slpA	GCTATCTTCACAGTTAAGCA	20	40	50.4
109	lb2242-slpA	CATTTAACTTTGCTTCAGTCA	21	33	50
110	lb2243-slpA	GCTATCAACTTGTGTATCAGA	21	38	50.4
111	lb2245-slpA	AGACTCATCTGTTAACTCTGA	21	38	50.7
112	lb2246-slpA	TGTATTTGCTAGTGAAGTAGC	21	38	50.7
113	lb2247-slpA	ATCAGCCATTACTAGACCAG	20	45	51.2
114	lb2248-slpA	GCTTTAAGAACTAATTTAACAGGA	24	29	50.6
115	lb2249-slpA	ACGTAGAATCTGTACCTATACT	22	36	50.3
116	lb2251-slpA	GTGTATAAGAAGCACTTAGATCT	23	35	50.7
117	lb2252-slpA	TCTGCTACAACATCAGTAGT	20	40	50.7
118	lb2253-slpA	TCATTTCAACAACATATCCAGT	22	32	51
119	lb2254-slpA	AACTACTTTAGCTGGTGTATC	21	38	50
120	lb2255-slpA	GTCTTTGCAGTTATACTAAGC	21	38	49.8
121	lb2257-slpA	GAATCACTACCTGGATTTGT	20	40	49.9
122	lb2259-slpA	ACCTTTACTGAACCTGGAG	19	47	51
123	lb2260-slpA	TCTTAACCGCTGAAGTATAATC	22	36	50.8
124	lb2261-slpA	TGGTTTAGCAACTACTGTG	19	42	49.9
125	lb2262-slpA	CTGGAACCACTTGAATATCAT	21	38	50.6
126	lb2264-slpA	GTAGTTGCACCACTAATAGTT	21	38	50.6
127	lb2266-slpA	TGTGAACTTGAATCAACATCT	21	33	50.7
128	lb2267-slpA	CTTTACCTGCAGTTGCAT	18	44	50.2
129	lb2268-slpA	TTGGATCTCCAACTGATACA	20	40	50.6
130	lb2270-slpA	CACCTAAACCTTTAAGTTGGT	21	38	50.9
131	lb2271-slpA	TCTTGATCTACAATCTTAACTGT	23	30	50.2
132	lb2272-slpA	TGTTTATCTGCCTTAAGCAC	20	40	50.9
133	lb2273-CD0425	AGTATCATTGTCTTCTCGTG	20	40	49.9
134	lb2274-ydiC	CCTATATCCAGCTTTAATACTCA	23	35	50.2
135	lb2275-tcdAp2	TGCACCTAAGTTTATTCCG	19	42	50
136	lb2276-bcrA	ATCCGACACATTTCAATATCT	21	33	50
137	lb2278-slpA	TGAATCACTACTTGGATTTGT	21	33	50
138	lb2280-spaE	ACATACCTTTCCATGTAGCT	20	40	51
139	lb2281-cdtB	TGGATCTCCTAAATAATTACGC	22	36	50.8
140	lb2282-cdtB	TTTGTATCTCCTAAATAATTACGC	24	29	50.4
