# Supplementary material for: DNA-Microarray-based Genotyping of Clostridium difficile
Source: BMC Microbiol. 2015 Aug 5;15:158. doi: 10.1186/s12866-015-0489-2 (PMC4526300; doi:10.1186/s12866-015-0489-2)
Supplement: Additional file 2: — Full datasets for all tested strains and isolates. (PDF 437 kb) [file 12866_2015_489_MOESM2_ESM.pdf]

| STRAIN / ISOLATE | Ribotype | Sequence type | Toxin A (total) | Toxin A Individual Probes | | | | Toxin B (total) | Toxin B Individual Probes | | | | | | | | |
|---|---|---|---|---|---|---|---|---|---|---|---|---|---|---|---|---|---|
| | | | | hp1132\|tcdAp1 | hp1134\|tcdAp2 | hp1135\|tcdAp3 | hp1247\|tcdAp2 | | hp1118\|tcdBp1 | hp1119\|tcdBp1 | hp1121\|tcdBp4 | hp1122\|tcdBp4 | hp1124\|tcdBp2 | hp1126\|tcdBp2 | hp1127\|tcdBp2 | hp1129\|tcdBp3 | hp1130\|tcdBp3 |
| **CLADE 1** | | | | | | | | | | | | | | | | | |
| **>HP-01** | | | POS | POS | POS | POS | POS | POS | NEG | POS | NEG | POS | NEG | NEG | NEG | POS | NEG |
| C. difficile QCD-63q42 (GenBank ABHD) in silico prediction | | 3 | POS | POS | POS | POS | POS | POS | NEG | POS | NEG | POS | NEG | NEG | NEG | POS | NEG |
| C. difficile BI9 (GenBank FN668944) in silico prediction | | 3 | POS | POS | POS | POS | POS | POS | NEG | POS | NEG | POS | NEG | NEG | NEG | POS | NEG |
| C. difficile BI-9 | 1 | 3 | POS | POS | POS | POS | POS | POS | NEG | POS | NEG | POS | NEG | NEG | NEG | POS | NEG |
| | | | POS | POS | POS | POS | POS | POS | NEG | POS | NEG | POS | NEG | NEG | NEG | POS | NEG |
| | | | POS | POS | POS | POS | POS | POS | NEG | POS | NEG | POS | NEG | NEG | NEG | POS | NEG |
| | | | POS | POS | POS | POS | POS | POS | NEG | POS | NEG | POS | NEG | NEG | NEG | POS | NEG |
| | | | POS | POS | POS | POS | POS | POS | NEG | POS | NEG | POS | NEG | NEG | NEG | POS | NEG |
| Freiburg-1040-73.104-DBK0000249 | 1 | | POS | POS | POS | POS | POS | POS | NEG | POS | NEG | POS | NEG | NEG | NEG | POS | NEG |
| | | | POS | POS | POS | POS | POS | POS | NEG | POS | NEG | POS | NEG | NEG | NEG | POS | NEG |
| Freiburg-1052-78.1052-DBK0000294 | 1 | | POS | POS | POS | POS | POS | POS | NEG | POS | NEG | POS | NEG | NEG | NEG | POS | NEG |
| | | | POS | POS | POS | POS | POS | POS | NEG | POS | NEG | POS | NEG | NEG | NEG | POS | NEG |
| Freiburg-605-1.605-DBK0000223 | 72 | | POS | POS | POS | POS | POS | POS | NEG | POS | NEG | POS | NEG | NEG | NEG | POS | NEG |
| Freiburg-787-22.787-DBK0000288 | 72 | | POS | POS | POS | POS | POS | POS | NEG | POS | NEG | POS | NEG | NEG | NEG | POS | NEG |
| | | | POS | POS | POS | POS | POS | POS | NEG | POS | NEG | POS | NEG | NEG | NEG | POS | NEG |
| Freiburg-897-40.897-DBK0000225 | 15 | | POS | POS | POS | POS | POS | POS | NEG | POS | NEG | POS | NEG | NEG | NEG | POS | NEG |
| | | | POS | POS | POS | POS | POS | POS | NEG | POS | NEG | POS | NEG | NEG | NEG | POS | NEG |
| Dresden 06_7735 | | | POS | POS | POS | POS | POS | POS | NEG | POS | NEG | POS | NEG | NEG | NEG | POS | NEG |
| Dresden 06_7736 | | | POS | POS | POS | POS | POS | POS | NEG | POS | NEG | POS | NEG | NEG | NEG | POS | NEG |
| Dresden 07_6943 | | | POS | POS | POS | POS | POS | POS | NEG | POS | NEG | POS | NEG | NEG | NEG | POS | NEG |
| Dresden 07_7200 | | | POS | POS | AMB | AMB | POS | POS | NEG | AMB | NEG | POS | NEG | NEG | NEG | POS | NEG |
| Dresden 07_7269 | | | POS | POS | POS | POS | POS | POS | NEG | POS | NEG | POS | NEG | NEG | NEG | POS | NEG |
| Dresden 07_7678 | | | POS | POS | POS | POS | POS | POS | NEG | POS | NEG | POS | NEG | NEG | NEG | POS | NEG |
| Dresden 07_7773 | | | POS | POS | POS | POS | POS | POS | NEG | POS | NEG | POS | NEG | NEG | NEG | POS | NEG |
| Dresden 07_7832 | | | POS | POS | POS | POS | POS | POS | NEG | POS | NEG | POS | NEG | NEG | NEG | POS | NEG |
| Dresden 07_7967 | | | POS | POS | POS | POS | POS | POS | NEG | POS | NEG | POS | NEG | NEG | NEG | POS | NEG |
| Dresden 07_8099 | | | POS | POS | POS | POS | POS | POS | NEG | POS | NEG | POS | NEG | NEG | NEG | POS | NEG |
| Dresden 07_8100 | | | POS | POS | POS | POS | POS | POS | NEG | POS | NEG | POS | NEG | NEG | NEG | POS | NEG |
| Dresden 07_8170 | | | POS | POS | POS | POS | POS | POS | NEG | POS | NEG | POS | NEG | NEG | NEG | POS | NEG |
| Dresden 08_0544 | 1 | | POS | POS | POS | POS | POS | POS | NEG | POS | NEG | POS | NEG | NEG | NEG | POS | NEG |
| Dresden 08_0550 | | | POS | POS | POS | POS | POS | POS | NEG | POS | NEG | POS | NEG | NEG | NEG | POS | NEG |
| Dresden 08_0827 | | | POS | POS | POS | POS | POS | POS | NEG | POS | NEG | POS | NEG | NEG | NEG | POS | NEG |
| Dresden 08_0902 | | | POS | POS | POS | POS | POS | POS | NEG | POS | NEG | POS | NEG | NEG | NEG | POS | NEG |
| Dresden 08_0955 | | | POS | POS | POS | POS | POS | POS | NEG | POS | NEG | POS | NEG | NEG | NEG | POS | NEG |
| Dresden 08_0958 | | | POS | POS | POS | POS | POS | POS | NEG | POS | NEG | POS | NEG | NEG | NEG | POS | NEG |
| Dresden 08_1073 | | | POS | POS | POS | POS | POS | POS | NEG | POS | NEG | POS | NEG | NEG | NEG | POS | NEG |
| Dresden 08_1074 | | | POS | POS | POS | POS | POS | POS | NEG | POS | NEG | POS | NEG | NEG | NEG | POS | NEG |
| Dresden 08_1206 | | | POS | POS | POS | POS | POS | POS | NEG | POS | NEG | POS | NEG | NEG | NEG | POS | NEG |
| Dresden 08_1216 | | | POS | POS | POS | POS | POS | POS | NEG | POS | NEG | POS | NEG | NEG | NEG | POS | NEG |
| Dresden 08_1305 | | | POS | POS | POS | POS | POS | POS | NEG | POS | NEG | POS | NEG | NEG | NEG | POS | NEG |
| Dresden 07_7679 | | | POS | POS | POS | AMB | POS | POS | NEG | POS | NEG | POS | NEG | NEG | NEG | POS | NEG |
| Dresden 07_7683 | | | POS | POS | POS | POS | POS | POS | NEG | POS | NEG | POS | NEG | NEG | NEG | POS | NEG |
| Dresden 07_7980 | | | POS | POS | POS | POS | POS | POS | NEG | POS | NEG | POS | NEG | NEG | NEG | POS | NEG |
| Dresden 07_8022 | | | POS | POS | POS | AMB | POS | POS | NEG | POS | NEG | POS | NEG | NEG | NEG | POS | NEG |
| Dresden 07_8291 | | | POS | POS | POS | POS | POS | POS | NEG | POS | NEG | POS | NEG | NEG | NEG | POS | NEG |
| Dresden 07_8314 | | | POS | POS | POS | POS | POS | POS | NEG | POS | NEG | POS | NEG | NEG | NEG | POS | NEG |
| Dresden 07_8315 | | | POS | POS | POS | POS | POS | POS | NEG | POS | NEG | POS | NEG | NEG | NEG | POS | NEG |
| Dresden 07_8323 | | | POS | POS | POS | POS | POS | POS | NEG | POS | NEG | POS | NEG | NEG | NEG | POS | NEG |
| Dresden 07_8356 | | | POS | POS | POS | POS | POS | POS | NEG | POS | NEG | POS | NEG | NEG | NEG | POS | NEG |
| Dresden 07_8557 | | | POS | POS | POS | POS | POS | POS | NEG | POS | NEG | POS | NEG | NEG | NEG | POS | NEG |
| Dresden 07_8736 | | | POS | POS | POS | POS | POS | POS | NEG | POS | NEG | POS | NEG | NEG | NEG | POS | NEG |
| Dresden 07_8945 | | | POS | POS | POS | POS | POS | POS | NEG | POS | NEG | POS | NEG | NEG | NEG | POS | NEG |
| Dresden 07_9138 | | | POS | POS | POS | POS | POS | POS | NEG | POS | NEG | POS | NEG | NEG | NEG | POS | NEG |
| Dresden 07_9175 | | | POS | POS | POS | POS | POS | POS | NEG | POS | NEG | POS | NEG | NEG | NEG | POS | NEG |
| Dresden 07_9234 | | | POS | POS | POS | POS | POS | POS | NEG | POS | NEG | POS | NEG | NEG | NEG | POS | NEG |
| Dresden 07_9255 | | | POS | POS | POS | POS | POS | POS | NEG | POS | NEG | POS | NEG | NEG | NEG | POS | NEG |
| Dresden 07_9309 | | | POS | POS | POS | POS | POS | POS | NEG | POS | NEG | POS | NEG | NEG | NEG | POS | NEG |
| Dresden 07_9330 | | | POS | POS | POS | POS | POS | POS | NEG | POS | NEG | POS | NEG | NEG | NEG | POS | NEG |
| Dresden 07_9399 | | | POS | POS | POS | POS | POS | POS | NEG | POS | NEG | POS | NEG | NEG | NEG | POS | NEG |
| Dresden 08_0188 | 1 | | POS | POS | POS | POS | POS | POS | NEG | POS | NEG | POS | NEG | NEG | NEG | POS | NEG |
| Dresden 08_0190 | | | POS | POS | POS | POS | POS | POS | NEG | POS | NEG | POS | NEG | NEG | NEG | POS | NEG |
| Dresden 08_0220 | | | POS | POS | POS | POS | POS | POS | NEG | POS | NEG | POS | NEG | NEG | NEG | POS | NEG |
| Dresden 08_0326 | | | POS | POS | POS | POS | POS | POS | NEG | POS | NEG | POS | NEG | NEG | NEG | POS | NEG |
| Dresden 08_0327 | | | POS | POS | POS | POS | POS | POS | NEG | POS | NEG | POS | NEG | NEG | NEG | POS | NEG |
| Dresden 08_0648 | | | POS | POS | POS | POS | POS | POS | NEG | POS | NEG | POS | NEG | NEG | NEG | POS | NEG |
| Dresden 08_0751 | | | POS | POS | POS | POS | POS | POS | NEG | POS | NEG | POS | NEG | NEG | NEG | POS | NEG |
| Dresden 08_0769 | | | POS | POS | POS | POS | POS | POS | NEG | POS | NEG | POS | NEG | NEG | NEG | POS | NEG |
| Dresden 08_0770 | | | POS | POS | POS | POS | POS | POS | NEG | POS | NEG | POS | NEG | NEG | NEG | POS | NEG |
| Dresden 08_0771 | | | POS | POS | POS | POS | POS | POS | NEG | POS | NEG | POS | NEG | NEG | NEG | POS | NEG |
| Dresden 08_1308 | | | POS | POS | POS | POS | POS | POS | NEG | POS | NEG | POS | NEG | NEG | NEG | POS | NEG |
| Dresden 08_1311 | | | POS | POS | POS | POS | POS | POS | NEG | POS | NEG | POS | NEG | NEG | NEG | POS | NEG |
| Dresden 08_1316 | | | POS | POS | POS | POS | POS | POS | NEG | POS | NEG | POS | NEG | NEG | NEG | POS | NEG |
| Dresden 08_1542 | | | POS | POS | POS | POS | POS | POS | NEG | POS | NEG | POS | NEG | NEG | NEG | POS | NEG |
| Dresden 08_1544 | | | POS | POS | POS | POS | POS | POS | NEG | POS | NEG | POS | NEG | NEG | NEG | POS | NEG |
| Dresden 08_1581 | | | POS | POS | POS | POS | POS | POS | NEG | POS | NEG | POS | NEG | NEG | NEG | POS | NEG |
| Dresden 08_1826 | | | POS | POS | POS | POS | POS | POS | NEG | POS | NEG | POS | NEG | NEG | NEG | POS | NEG |
| Dresden 08_1817 | | | POS | POS | POS | POS | POS | POS | NEG | POS | NEG | POS | NEG | NEG | NEG | POS | NEG |
| Dresden 08_1791 | | | POS | POS | POS | POS | POS | POS | NEG | POS | NEG | POS | NEG | NEG | NEG | POS | NEG |
| Dresden 08_1643 | | | POS | POS | POS | POS | POS | POS | NEG | POS | NEG | POS | NEG | NEG | NEG | POS | NEG |
| Dresden 08_1764 | | | POS | POS | POS | POS | POS | POS | NEG | POS | NEG | POS | NEG | NEG | NEG | POS | NEG |
| Dresden 08_1748 | | | POS | POS | POS | POS | POS | POS | NEG | POS | NEG | POS | NEG | NEG | NEG | POS | NEG |
| Dresden 08_1736 | | | POS | POS | POS | POS | POS | POS | NEG | POS | NEG | POS | NEG | NEG | NEG | POS | NEG |
| Dresden 08_1582 | | | POS | POS | POS | POS | POS | POS | NEG | POS | NEG | POS | NEG | NEG | NEG | POS | NEG |
| **>HP-02** | | | POS | POS | POS | POS | POS | POS | NEG | POS | NEG | POS | NEG | NEG | NEG | POS | NEG |
| C. difficile ATCC43255 (GenBank ABKJ) in silico prediction | | 46 | POS | POS | POS | POS | POS | POS | NEG | POS | NEG | POS | NEG | NEG | NEG | POS | NEG |
| Freiburg-885-36.885-DBK0000296 | 13 | 45 | POS | POS | POS | POS | POS | POS | NEG | POS | NEG | POS | NEG | NEG | NEG | POS | NEG |
| Freiburg_37.894-II | | | POS | POS | POS | POS | POS | POS | NEG | POS | NEG | POS | NEG | NEG | NEG | POS | NEG |
| Freiburg_981-57.981-DBK0000258 | 87 | | POS | POS | POS | POS | POS | POS | NEG | POS | NEG | POS | NEG | NEG | NEG | POS | NEG |
| | | | POS | POS | POS | POS | POS | POS | NEG | POS | NEG | POS | NEG | NEG | NEG | POS | NEG |
| Jena_S0011_11 | | | POS | POS | POS | POS | POS | POS | NEG | POS | NEG | POS | NEG | NEG | NEG | POS | NEG |
| | | | POS | POS | POS | POS | POS | POS | NEG | POS | NEG | POS | NEG | NEG | NEG | POS | NEG |
| Dresden 08_1352 | | | POS | POS | POS | POS | POS | POS | NEG | POS | NEG | POS | NEG | NEG | NEG | POS | NEG |
| **>HP-03** | | | POS | POS | POS | POS | POS | POS | NEG | POS | NEG | POS | NEG | NEG | NEG | POS | NEG |
| Freiburg-938-47.938-DBK0000237 | 56 | | POS | POS | POS | POS | POS | POS | NEG | POS | NEG | POS | NEG | NEG | NEG | POS | NEG |
| | | | POS | POS | POS | POS | POS | POS | NEG | POS | NEG | POS | NEG | NEG | NEG | POS | NEG |
| Freiburg-811-25.811-DBK0000284 | 49 | | POS | POS | POS | POS | POS | POS | NEG | POS | NEG | POS | NEG | NEG | NEG | POS | NEG |
| | | | POS | POS | POS | POS | POS | POS | NEG | POS | NEG | POS | NEG | NEG | NEG | POS | NEG |
| Freiburg-987-62.987-DBK0000251 | 11 | 58 | POS | POS | POS | POS | POS | POS | NEG | POS | NEG | POS | NEG | NEG | NEG | POS | NEG |
| | | | POS | POS | POS | POS | POS | POS | NEG | POS | NEG | POS | NEG | NEG | NEG | POS | NEG |
| Freiburg-972-54.972-DBK0000247 | 56 | | POS | POS | POS | POS | POS | POS | NEG | POS | NEG | POS | NEG | NEG | NEG | POS | NEG |
| | | | POS | POS | POS | POS | POS | POS | NEG | POS | NEG | POS | NEG | NEG | NEG | POS | NEG |
| **>HP-04** | | | POS | POS | POS | POS | POS | POS | NEG | POS | NEG | POS | NEG | NEG | NEG | POS | NEG |
| Freiburg-984-60.984-DBK0000250 | 137 | | POS | POS | POS | POS | POS | POS | NEG | POS | NEG | POS | NEG | NEG | NEG | POS | NEG |
| Freiburg-1023-72.1023-DBK0000271 | 137 | 4 | POS | POS | POS | POS | POS | POS | NEG | POS | NEG | POS | NEG | NEG | NEG | POS | NEG |
| | | | POS | POS | POS | POS | POS | POS | NEG | POS | NEG | POS | NEG | NEG | NEG | POS | NEG |
| Freiburg-1043-74.1043-DBK0000268 | 150 | | POS | POS | POS | POS | POS | POS | NEG | POS | NEG | POS | NEG | NEG | NEG | POS | NEG |
| Freiburg-633-2.663-DBK0000292 | ? | | POS | POS | POS | POS | POS | POS | NEG | POS | NEG | POS | NEG | NEG | NEG | POS | NEG |
| | | | POS | POS | POS | POS | POS | POS | NEG | POS | NEG | POS | NEG | NEG | NEG | POS | NEG |
| Freiburg-782-20.782-DBK0000276 | 150 | | POS | POS | POS | POS | POS | POS | NEG | POS | NEG | POS | NEG | NEG | NEG | POS | NEG |
| | | | POS | POS | POS | POS | POS | POS | NEG | POS | NEG | POS | NEG | NEG | NEG | POS | NEG |
| **>HP-05** | | | POS | POS | POS | POS | POS | POS | NEG | POS | NEG | POS | NEG | NEG | NEG | POS | NEG |
| Freiburg-740-17.74-DBK0000234 | 163 | | POS | POS | POS | POS | POS | POS | NEG | POS | NEG | POS | NEG | NEG | NEG | POS | NEG |
| | | | POS | POS | POS | POS | POS | POS | NEG | POS | NEG | POS | NEG | NEG | NEG | POS | NEG |
| Freiburg-793-23.793-DBK0000286 | 163 | | POS | POS | POS | POS | POS | POS | NEG | POS | NEG | POS | NEG | NEG | NEG | POS | NEG |
| | | | POS | POS | POS | POS | POS | POS | NEG | POS | NEG | POS | NEG | NEG | NEG | POS | NEG |
| **>HP-06** | | | POS | POS | POS | POS | POS | POS | NEG | POS | NEG | POS | NEG | NEG | NEG | POS | NEG |
| Freiburg-874-35.874-DBK0000282 | | | POS | POS | POS | POS | POS | POS | NEG | POS | NEG | POS | NEG | NEG | NEG | POS | NEG |
| | | | POS | POS | POS | POS | POS | POS | NEG | POS | NEG | POS | NEG | NEG | NEG | POS | NEG |

| STRAIN / ISOLATE | Ribotype | Sequence type | Toxin A (total) | Toxin A Individual Probes | | | | Toxin B (total) | Toxin B Individual Probes | | | | | | | | |
|---|---|---|---|---|---|---|---|---|---|---|---|---|---|---|---|---|---|
| | | | | hp1132\|tcdAp1 | hp1134\|tcdAp2 | hp1135\|tcdAp3 | hp1247\|tcdAp2 | | hp1118\|tcdBp1 | hp1119\|tcdBp1 | hp1121\|tcdBp4 | hp1122\|tcdBp4 | hp1124\|tcdBp2 | hp1126\|tcdBp2 | hp1127\|tcdBp2 | hp1129\|tcdBp3 | hp1130\|tcdBp3 |
| >HP-07 | | | POS | POS | POS | POS | POS | POS | NEG | POS | NEG | POS | NEG | NEG | NEG | POS | NEG |
| Freiburg-923-44.923-DBK0000238 | 54 | | POS | POS | POS | POS | POS | POS | NEG | POS | NEG | POS | NEG | NEG | NEG | POS | NEG |
| | | | POS | POS | POS | POS | POS | POS | NEG | POS | NEG | POS | NEG | NEG | NEG | POS | NEG |
| >HP-08 | | | POS | POS | POS | POS | POS | POS | NEG | POS | NEG | POS | NEG | NEG | NEG | POS | NEG |
| Freiburg-1021-71.1021-DBK0000248 | 57 | 55 | POS | POS | POS | POS | POS | POS | NEG | POS | NEG | POS | NEG | NEG | NEG | POS | NEG |
| | | | POS | POS | POS | POS | POS | POS | NEG | POS | NEG | POS | NEG | NEG | NEG | POS | NEG |
| Freiburg-669-9.669-DBK0000231 | 70 | | POS | POS | POS | POS | POS | POS | NEG | POS | NEG | POS | NEG | NEG | NEG | POS | NEG |
| | | | POS | POS | POS | POS | POS | POS | NEG | POS | NEG | POS | NEG | NEG | NEG | POS | NEG |
| Freiburg-964-53.964-DBK0000267 | 57 | | POS | POS | POS | POS | POS | POS | NEG | POS | NEG | POS | NEG | NEG | NEG | POS | NEG |
| | | | POS | POS | POS | POS | POS | POS | NEG | POS | NEG | POS | NEG | NEG | NEG | POS | NEG |
| Freiburg-899-41.899-DBK0000224 | 70 | | POS | POS | POS | POS | POS | POS | NEG | POS | NEG | POS | NEG | NEG | NEG | POS | NEG |
| | | | POS | POS | POS | POS | POS | POS | NEG | POS | NEG | POS | NEG | NEG | NEG | POS | NEG |
| Freiburg-982-58.982-DBK0000257 | 94 | | POS | POS | POS | POS | POS | POS | NEG | POS | NEG | POS | NEG | NEG | NEG | POS | NEG |
| | | | POS | POS | POS | POS | POS | POS | NEG | AMB | NEG | POS | NEG | NEG | NEG | POS | NEG |
| Dresden 07_7035 | 70 | | POS | POS | POS | POS | POS | POS | NEG | POS | NEG | POS | NEG | NEG | NEG | POS | NEG |
| Dresden 07_7248 | | | POS | POS | POS | POS | POS | POS | NEG | POS | NEG | POS | NEG | NEG | NEG | POS | NEG |
| Dresden 07_8702 | | | POS | POS | POS | POS | POS | POS | NEG | POS | NEG | POS | NEG | NEG | NEG | POS | NEG |
| Dresden 07_8989 | | | POS | POS | POS | POS | POS | POS | NEG | POS | NEG | POS | NEG | NEG | NEG | POS | NEG |
| Dresden 08_0712 | | | POS | POS | POS | POS | POS | POS | NEG | POS | NEG | POS | NEG | NEG | NEG | POS | NEG |
| >HP-09 | | | POS | POS | POS | POS | POS | POS | NEG | POS | NEG | POS | NEG | NEG | NEG | POS | NEG |
| Freiburg-1056-79.1056-DBK0000261 | 2 | 8 | POS | POS | POS | POS | POS | POS | NEG | POS | NEG | POS | NEG | NEG | NEG | POS | NEG |
| Freiburg-658-8.658-DBK0000230 | 159 | | POS | POS | POS | POS | POS | POS | NEG | POS | NEG | POS | NEG | NEG | NEG | POS | NEG |
| | | | POS | POS | POS | POS | POS | POS | NEG | POS | NEG | POS | NEG | NEG | NEG | POS | NEG |
| Freiburg-963-52.963-DBK0000265 | 2 | | POS | POS | POS | POS | POS | POS | NEG | POS | NEG | POS | NEG | NEG | NEG | POS | NEG |
| | | | POS | POS | POS | POS | POS | POS | NEG | POS | NEG | POS | NEG | NEG | NEG | POS | NEG |
| Freiburg-988-63.988-DBK0000253 | 159 | | POS | POS | POS | POS | POS | POS | NEG | POS | NEG | POS | NEG | NEG | NEG | POS | NEG |
| | | | POS | POS | POS | POS | POS | POS | NEG | POS | NEG | POS | NEG | NEG | NEG | POS | NEG |
| Freiburg_79.1057 | | | POS | POS | POS | POS | POS | POS | NEG | POS | NEG | POS | NEG | NEG | NEG | POS | NEG |
| Jena_S0017.11 | | | POS | POS | POS | POS | POS | POS | NEG | POS | NEG | POS | NEG | NEG | NEG | POS | NEG |
| | | | POS | POS | POS | POS | POS | POS | NEG | POS | NEG | POS | NEG | NEG | NEG | POS | NEG |
| | | | POS | POS | POS | POS | POS | POS | NEG | POS | NEG | POS | NEG | NEG | NEG | POS | NEG |
| | | | POS | POS | POS | POS | POS | POS | NEG | POS | NEG | POS | NEG | NEG | NEG | POS | NEG |
| | | | POS | POS | POS | POS | POS | POS | NEG | POS | NEG | POS | NEG | NEG | NEG | POS | NEG |
| Jena_S0015_11 | | | POS | POS | POS | POS | POS | POS | NEG | POS | NEG | POS | NEG | NEG | NEG | POS | NEG |
| | | | POS | POS | POS | POS | POS | POS | NEG | POS | NEG | POS | NEG | NEG | NEG | POS | NEG |
| | | | POS | POS | POS | POS | POS | POS | NEG | POS | NEG | POS | NEG | NEG | NEG | POS | NEG |
| Jena_S0012_11 | | | POS | POS | POS | POS | POS | POS | NEG | POS | NEG | POS | NEG | NEG | NEG | POS | NEG |
| | | | POS | POS | POS | POS | POS | POS | NEG | POS | NEG | POS | NEG | NEG | NEG | POS | NEG |
| Dresden 07_7363 | | | POS | POS | POS | POS | POS | POS | NEG | POS | NEG | POS | NEG | NEG | NEG | POS | NEG |
| Dresden 07_8174 | | | POS | POS | POS | AMB | POS | POS | NEG | POS | NEG | POS | NEG | NEG | NEG | POS | NEG |
| Dresden 07_8224 | | | POS | POS | POS | POS | POS | POS | NEG | POS | NEG | POS | NEG | NEG | NEG | POS | NEG |
| Dresden 07_8562 | | | POS | POS | POS | POS | POS | POS | NEG | POS | NEG | POS | NEG | NEG | NEG | POS | NEG |
| Dresden 08_613 | 2 | | POS | POS | POS | POS | POS | POS | NEG | POS | NEG | POS | NEG | NEG | NEG | POS | NEG |
| Dresden 08_0422 | | | POS | POS | POS | POS | POS | POS | NEG | POS | NEG | POS | NEG | NEG | NEG | POS | NEG |
| Dresden 08_0837 | | | POS | POS | POS | POS | POS | POS | NEG | POS | NEG | POS | NEG | NEG | NEG | POS | NEG |
| Dresden 08_0878 | | | POS | POS | POS | POS | POS | POS | NEG | POS | NEG | POS | NEG | NEG | NEG | POS | NEG |
| Dresden 08_1484 | | | POS | POS | POS | POS | POS | POS | NEG | POS | NEG | POS | NEG | NEG | NEG | POS | NEG |
| >HP-10 | | | POS | POS | POS | POS | POS | POS | NEG | POS | NEG | POS | NEG | NEG | NEG | POS | NEG |
| Dresden 09_664_1 | | | POS | POS | POS | POS | POS | POS | NEG | POS | NEG | POS | NEG | NEG | NEG | POS | NEG |
| Dresden 09_664_2 | | | POS | POS | POS | POS | POS | POS | NEG | POS | NEG | POS | NEG | NEG | NEG | POS | NEG |
| Dresden 09_711_1 | | | POS | POS | POS | POS | POS | POS | NEG | POS | NEG | POS | NEG | NEG | NEG | POS | NEG |
| Dresden 09_711_2 | | | POS | POS | POS | POS | POS | POS | NEG | POS | NEG | POS | NEG | NEG | NEG | POS | NEG |
| Dresden 09_932 | | | POS | POS | POS | POS | POS | POS | NEG | POS | NEG | POS | NEG | NEG | NEG | POS | NEG |
| Dresden 09_939 | | | POS | POS | POS | POS | POS | POS | NEG | POS | NEG | POS | NEG | NEG | NEG | POS | NEG |
| Dresden 09_948 | | | POS | POS | POS | POS | POS | POS | NEG | POS | NEG | POS | NEG | NEG | NEG | POS | NEG |
| >HP-11 | | | NEG | NEG | NEG | NEG | NEG | NEG | NEG | NEG | NEG | NEG | NEG | NEG | NEG | NEG | NEG |
| C. difficile CD37 (GenBank AHJJ) in silico prediction | | 3 | NEG | NEG | NEG | NEG | NEG | NEG | NEG | NEG | NEG | NEG | NEG | NEG | NEG | NEG | NEG |
| Freiburg-684-10.684-DBK0000240 | 9 | | NEG | NEG | NEG | NEG | NEG | NEG | NEG | NEG | NEG | NEG | NEG | NEG | NEG | NEG | NEG |
| Freiburg-838-28.838-DBK0000278 | 9 | 3 | NEG | NEG | NEG | NEG | NEG | NEG | NEG | NEG | NEG | NEG | NEG | NEG | NEG | NEG | NEG |
| | | | NEG | NEG | NEG | NEG | NEG | NEG | NEG | NEG | NEG | NEG | NEG | NEG | NEG | NEG | NEG |
| >HP-12 | | | POS | POS | POS | POS | POS | POS | NEG | POS | NEG | POS | NEG | NEG | NEG | POS | NEG |
| Freiburg-1007-68.1007-DBK0000252 | 103 | | POS | POS | POS | POS | POS | POS | NEG | POS | NEG | POS | NEG | NEG | NEG | POS | NEG |
| | | | POS | POS | POS | POS | POS | POS | NEG | AMB | NEG | AMB | NEG | NEG | NEG | POS | NEG |
| Freiburg-983-59.983-DBK0000256 | 103 | | POS | POS | POS | POS | POS | POS | NEG | POS | NEG | POS | NEG | NEG | NEG | POS | NEG |
| | | | POS | POS | POS | POS | POS | POS | NEG | POS | NEG | POS | NEG | NEG | NEG | POS | NEG |
| >HP-13 | | | POS | POS | POS | POS | POS | POS | NEG | POS | NEG | POS | NEG | NEG | NEG | POS | NEG |
| C. difficile 70-100-2010 (GenBank AGAC) in silico prediction | | 42 | POS | POS | POS | POS | POS | POS | NEG | POS | NEG | POS | NEG | NEG | NEG | POS | NEG |
| Freiburg-1044-75.1044-DBK0000270 | 49 | | POS | POS | POS | POS | POS | POS | NEG | POS | NEG | POS | NEG | NEG | NEG | POS | NEG |
| | | | POS | POS | POS | POS | POS | POS | NEG | POS | NEG | POS | NEG | NEG | NEG | POS | NEG |
| Freiburg-1057-80.1057-DBK0000260 | 14 | 2 or 102 | POS | POS | POS | POS | POS | POS | NEG | POS | NEG | POS | NEG | NEG | NEG | POS | NEG |
| | | | POS | POS | POS | POS | POS | POS | NEG | POS | NEG | POS | NEG | NEG | NEG | POS | NEG |
| Freiburg-710-12.710-DBK0000241 | ? | | POS | POS | POS | POS | POS | POS | NEG | POS | NEG | POS | NEG | NEG | NEG | POS | NEG |
| | | | POS | POS | POS | POS | POS | POS | NEG | POS | NEG | POS | NEG | NEG | NEG | POS | NEG |
| Freiburg-940-48.94-DBK0000244 | ? | | POS | POS | POS | POS | POS | POS | NEG | POS | NEG | POS | NEG | NEG | NEG | POS | NEG |
| | | | POS | POS | POS | POS | POS | POS | NEG | POS | NEG | POS | NEG | NEG | NEG | POS | NEG |
| Freiburg-980-56.98-DBK0000246 | 14 | | POS | POS | POS | POS | POS | POS | NEG | POS | NEG | POS | NEG | NEG | NEG | POS | NEG |
| | | | POS | POS | POS | POS | POS | POS | NEG | POS | NEG | POS | NEG | NEG | NEG | POS | NEG |
| Jena_S0014_11 | | | POS | POS | POS | POS | POS | POS | NEG | POS | NEG | POS | NEG | NEG | NEG | POS | NEG |
| | | | POS | POS | POS | POS | POS | POS | NEG | POS | NEG | POS | NEG | NEG | NEG | POS | NEG |
| | | | POS | POS | POS | POS | POS | POS | NEG | POS | NEG | POS | NEG | NEG | NEG | POS | NEG |
| Jena_S0023_11 | | | POS | POS | POS | POS | POS | POS | NEG | POS | NEG | POS | NEG | NEG | NEG | POS | NEG |
| | | | POS | POS | POS | POS | POS | POS | NEG | POS | NEG | POS | NEG | NEG | NEG | POS | NEG |
| | | | POS | POS | POS | POS | POS | POS | NEG | POS | NEG | POS | NEG | NEG | NEG | POS | NEG |
| Dresden 07_7779 | 14 | | POS | POS | NEG | NEG | AMB | POS | NEG | POS | NEG | AMB | NEG | NEG | NEG | AMB | NEG |
| Dresden 07_7950 | | | POS | POS | POS | POS | POS | POS | NEG | POS | NEG | POS | NEG | NEG | NEG | POS | NEG |
| Dresden 07_7953 | | | POS | POS | POS | POS | POS | POS | NEG | POS | NEG | POS | NEG | NEG | NEG | POS | NEG |
| Dresden 07_8015 | | | POS | POS | POS | POS | POS | POS | NEG | POS | NEG | POS | NEG | NEG | NEG | POS | NEG |
| Dresden 07_8205 | | | POS | POS | POS | AMB | POS | POS | NEG | POS | NEG | POS | NEG | NEG | NEG | POS | NEG |
| Dresden 07_8595 | | | POS | POS | POS | POS | POS | POS | NEG | POS | NEG | POS | NEG | NEG | NEG | POS | NEG |
| Dresden 07_8613 | | | POS | POS | AMB | POS | POS | POS | NEG | POS | NEG | POS | NEG | NEG | NEG | POS | NEG |
| Dresden 07_8686 | | | POS | POS | POS | POS | POS | POS | NEG | POS | NEG | POS | NEG | NEG | NEG | POS | NEG |
| Dresden 07_9180 | | | POS | POS | POS | POS | POS | POS | NEG | POS | NEG | POS | NEG | NEG | NEG | POS | NEG |
| Dresden 08_0237 | | | POS | POS | POS | POS | POS | POS | NEG | POS | NEG | POS | NEG | NEG | NEG | POS | NEG |
| Dresden 08_0492 | | | POS | POS | POS | POS | POS | POS | NEG | POS | NEG | POS | NEG | NEG | NEG | POS | NEG |
| Dresden 08_0992 | | | POS | POS | POS | POS | POS | POS | NEG | POS | NEG | POS | NEG | NEG | NEG | POS | NEG |
| Dresden 08_1184 | | | POS | POS | POS | POS | POS | POS | NEG | POS | NEG | POS | NEG | NEG | NEG | POS | NEG |
| Dresden 08_1314 | | | POS | POS | POS | POS | POS | POS | NEG | POS | NEG | POS | NEG | NEG | NEG | POS | NEG |
| Dresden 08_1640 | | | POS | POS | POS | POS | POS | POS | NEG | POS | NEG | POS | NEG | NEG | NEG | POS | NEG |
| Dresden 08_1640 | | | POS | POS | POS | POS | POS | POS | NEG | POS | NEG | POS | NEG | NEG | NEG | POS | NEG |
| Dresden 08_1758 | | | POS | POS | POS | POS | POS | POS | NEG | POS | NEG | POS | NEG | NEG | NEG | POS | NEG |
| >HP-14 | | | POS | POS | POS | POS | POS | POS | NEG | POS | NEG | POS | NEG | NEG | NEG | POS | NEG |
| Freiburg-1019-70.1019-DBK0000255 | 15 | | POS | POS | POS | POS | POS | POS | NEG | POS | NEG | POS | NEG | NEG | NEG | POS | NEG |
| >HP-15 | | | NEG | NEG | NEG | NEG | NEG | NEG | NEG | NEG | NEG | NEG | NEG | NEG | NEG | NEG | NEG |
| Freiburg_42.907 | | | NEG | NEG | NEG | NEG | NEG | NEG | NEG | NEG | NEG | NEG | NEG | NEG | NEG | NEG | NEG |
| >HP-16 | | | POS | POS | POS | POS | POS | POS | NEG | POS | NEG | POS | NEG | NEG | NEG | POS | NEG |
| Freiburg-978-55.978-DBK0000259 | 29 | | POS | POS | POS | POS | POS | POS | NEG | POS | NEG | POS | NEG | NEG | NEG | POS | NEG |
| | | | POS | POS | POS | POS | POS | POS | NEG | POS | NEG | POS | NEG | NEG | NEG | POS | NEG |
| Dresden 08_1810 | | | POS | POS | POS | POS | POS | POS | NEG | POS | NEG | POS | NEG | NEG | NEG | POS | NEG |
| >HP-17 | | | POS | POS | POS | POS | POS | POS | NEG | POS | NEG | POS | NEG | NEG | NEG | POS | NEG |
| Freiburg-729-15.729-DBK0000226 | 64 | | POS | POS | POS | POS | POS | POS | NEG | POS | NEG | POS | NEG | NEG | NEG | POS | NEG |
| | | | POS | POS | POS | POS | POS | POS | NEG | POS | NEG | POS | NEG | NEG | NEG | POS | NEG |
| Freiburg-926-45.926-DBK0000236 | 64 | | POS | POS | POS | POS | POS | POS | NEG | POS | NEG | POS | NEG | NEG | NEG | POS | NEG |
| | | | POS | POS | POS | POS | POS | POS | NEG | POS | NEG | POS | NEG | NEG | NEG | POS | NEG |
| >HP-18 | | | POS | POS | POS | POS | POS | POS | NEG | POS | NEG | POS | NEG | NEG | NEG | POS | NEG |
| Freiburg-651-7.651-DBK0000229 | 5 | 17 | POS | POS | POS | POS | POS | POS | NEG | POS | NEG | POS | NEG | NEG | NEG | POS | NEG |
| | | | POS | POS | POS | POS | POS | POS | NEG | POS | NEG | POS | NEG | NEG | NEG | POS | NEG |
| Freiburg-725-13.725-DBK0000227 | 5 | | POS | POS | POS | POS | POS | POS | NEG | POS | NEG | POS | NEG | NEG | NEG | POS | NEG |
| | | | POS | POS | POS | POS | POS | POS | NEG | POS | NEG | POS | NEG | NEG | NEG | POS | NEG |
| Dresden 07_7176 | | | POS | POS | AMB | NEG | POS | AMB | NEG | NEG | NEG | AMB | NEG | NEG | NEG | AMB | NEG |
| Dresden 07_7209 | | | POS | POS | POS | POS | POS | POS | NEG | POS | NEG | POS | NEG | NEG | NEG | POS | NEG |
| Dresden 07_8029 | | | POS | POS | POS | POS | POS | POS | NEG | POS | NEG | POS | NEG | NEG | NEG | POS | NEG |
| Dresden 07_8575 | | | POS | POS | POS | POS | POS | POS | NEG | POS | NEG | POS | NEG | NEG | NEG | POS | NEG |
| Dresden 08_1746 | | | POS | POS | POS | POS | POS | POS | NEG | POS | NEG | POS | NEG | NEG | NEG | POS | NEG |
| >HP-19 | | | POS | POS | POS | POS | POS | POS | NEG | POS | NEG | POS | NEG | NEG | NEG | POS | NEG |
| Freiburg-894-37.894-DBK0000289 | 87 | | POS | POS | POS | POS | POS | POS | NEG | POS | NEG | POS | NEG | NEG | NEG | POS | NEG |
| | | | POS | POS | POS | POS | POS | POS | NEG | POS | NEG | POS | NEG | NEG | NEG | POS | NEG |
| Freiburg-1000-67.1-DBK0000263 | 13 | | POS | POS | POS | POS | POS | POS | NEG | POS | NEG | POS | NEG | NEG | NEG | POS | NEG |
| | | | POS | POS | POS | POS | POS | POS | NEG | POS | NEG | POS | NEG | NEG | NEG | POS | NEG |
| Dresden 07_7828 | | | POS | POS | POS | AMB | POS | POS | NEG | POS | NEG | POS | NEG | NEG | NEG | POS | NEG |
| Dresden 08_1128 | | | POS | POS | POS | POS | POS | POS | NEG | POS | NEG | POS | NEG | NEG | NEG | POS | NEG |

| STRAIN / ISOLATE | Ribotype | Sequence type | Toxin A (total) | Toxin A Individual Probes | | | | Toxin B (total) | Toxin B Individual Probes | | | | | | | | |
|---|---|---|---|---|---|---|---|---|---|---|---|---|---|---|---|---|---|
| | | | | hp1132\|tcdAp1 | hp1134\|tcdAp2 | hp1135\|tcdAp3 | hp1247\|tcdAp2 | | hp1118\|tcdBp1 | hp1119\|tcdBp1 | hp1121\|tcdBp4 | hp1122\|tcdBp4 | hp1124\|tcdBp2 | hp1126\|tcdBp2 | hp1127\|tcdBp2 | hp1129\|tcdBp3 | hp1130\|tcdBp3 |
| **>HP-20** | | | POS | POS | POS | POS | POS | POS | NEG | POS | NEG | POS | NEG | NEG | NEG | POS | NEG |
| Freiburg-963-50.963-DBK0000243 | 45 | 8 | POS | POS | POS | POS | POS | POS | NEG | POS | NEG | POS | NEG | NEG | NEG | POS | NEG |
| Freiburg-750-18.75-DBK0000232 | 5 | | POS | POS | POS | POS | POS | POS | NEG | POS | NEG | POS | NEG | NEG | NEG | POS | NEG |
| | | | POS | POS | POS | POS | POS | POS | NEG | POS | NEG | POS | NEG | NEG | NEG | POS | NEG |
| Freiburg-637-4.637-DBK0000280 | ? | | POS | POS | POS | POS | POS | POS | NEG | POS | NEG | POS | NEG | NEG | NEG | POS | NEG |
| | | | POS | POS | POS | POS | POS | POS | NEG | POS | NEG | POS | NEG | NEG | NEG | POS | NEG |
| Freiburg-856-31.856-DBK0000273 | 54 | | POS | POS | POS | POS | POS | POS | NEG | POS | NEG | POS | NEG | NEG | NEG | POS | NEG |
| | | | POS | POS | POS | POS | POS | POS | NEG | POS | NEG | POS | NEG | NEG | NEG | POS | NEG |
| Freiburg_51.956 | | | POS | POS | POS | POS | POS | POS | NEG | POS | NEG | POS | NEG | NEG | NEG | POS | NEG |
| Dresden 07_7984 | 5 | | POS | POS | POS | POS | POS | POS | NEG | POS | NEG | POS | NEG | NEG | NEG | POS | NEG |
| Dresden 07_8203 | | | POS | POS | POS | AMB | POS | POS | NEG | POS | NEG | POS | NEG | NEG | NEG | POS | NEG |
| Dresden 07_8398 | | | POS | POS | POS | POS | POS | POS | NEG | POS | NEG | POS | NEG | NEG | NEG | POS | NEG |
| Dresden 07_8798 | | | POS | POS | POS | POS | POS | POS | NEG | POS | NEG | POS | NEG | NEG | NEG | POS | NEG |
| **>HP-21** | | | NEG | NEG | NEG | NEG | NEG | NEG | NEG | NEG | NEG | NEG | NEG | NEG | NEG | NEG | NEG |
| Freiburg-823-26.823-DBK0000283 | 31 | | NEG | NEG | NEG | NEG | NEG | NEG | NEG | NEG | NEG | NEG | NEG | NEG | NEG | NEG | NEG |
| | | | NEG | NEG | NEG | NEG | NEG | NEG | NEG | NEG | NEG | NEG | NEG | NEG | NEG | NEG | NEG |
| Freiburg-952-49.952-DBK0000245 | 31 | | NEG | NEG | NEG | NEG | NEG | NEG | NEG | NEG | NEG | NEG | NEG | NEG | NEG | NEG | NEG |
| | | | NEG | NEG | NEG | NEG | NEG | NEG | NEG | NEG | NEG | NEG | NEG | NEG | NEG | NEG | NEG |
| **>HP-22** | | | POS | POS | POS | POS | POS | POS | NEG | POS | NEG | POS | NEG | NEG | NEG | POS | NEG |
| Dresden 08_0288 | | | POS | POS | POS | POS | POS | POS | NEG | POS | NEG | POS | NEG | NEG | NEG | POS | NEG |
| **>HP-23** | | | POS | POS | POS | POS | POS | POS | NEG | POS | NEG | POS | NEG | NEG | NEG | POS | NEG |
| Freiburg-786-21.786-DBK0000275 | 12 | 54 | POS | POS | POS | POS | POS | POS | NEG | POS | NEG | POS | NEG | NEG | NEG | POS | NEG |
| | | | POS | POS | POS | POS | POS | POS | NEG | POS | NEG | POS | NEG | NEG | NEG | POS | NEG |
| **>HP-24** | | | POS | POS | POS | POS | POS | POS | NEG | POS | NEG | POS | NEG | NEG | NEG | POS | NEG |
| C. difficile 6534 (GenBank ADEJ) in silico prediction | | ST54 (1 MM) | POS | POS | POS | POS | POS | POS | NEG | POS | NEG | POS | NEG | NEG | NEG | POS | NEG |
| C. difficile 630 (GenBank AM180355) in silico prediction | | 54 | POS | POS | POS | POS | POS | POS | NEG | POS | NEG | POS | NEG | NEG | NEG | POS | NEG |
| C. difficile Strain 630 | 12 | 54 | POS | POS | POS | POS | POS | POS | NEG | POS | NEG | POS | NEG | NEG | NEG | POS | NEG |
| | | | POS | POS | POS | POS | POS | POS | NEG | POS | NEG | POS | NEG | NEG | NEG | POS | NEG |
| | | | POS | POS | POS | POS | POS | POS | NEG | POS | NEG | POS | NEG | NEG | NEG | POS | NEG |
| | | | POS | POS | POS | POS | POS | POS | NEG | POS | NEG | POS | NEG | NEG | NEG | POS | NEG |
| | | | POS | POS | POS | POS | POS | POS | NEG | POS | NEG | POS | NEG | NEG | NEG | POS | NEG |
| | | | POS | POS | POS | POS | POS | POS | NEG | POS | NEG | POS | NEG | NEG | NEG | POS | NEG |
| Dresden 07_7156 | | | POS | POS | AMB | AMB | POS | POS | NEG | AMB | NEG | POS | NEG | NEG | NEG | POS | NEG |
| Dresden 07_7158 | 12 | | POS | POS | POS | POS | POS | POS | NEG | POS | NEG | POS | NEG | NEG | NEG | POS | NEG |
| Dresden 07_7203 | | | POS | POS | POS | POS | POS | POS | NEG | POS | NEG | POS | NEG | NEG | NEG | POS | NEG |
| Dresden 07_8217 | | | POS | POS | POS | POS | POS | POS | NEG | POS | NEG | POS | NEG | NEG | NEG | POS | NEG |
| Dresden 07_8270 | | | POS | POS | POS | POS | POS | POS | NEG | POS | NEG | POS | NEG | NEG | NEG | POS | NEG |
| Dresden 07_8749 | | | POS | POS | AMB | AMB | POS | POS | NEG | AMB | NEG | POS | NEG | NEG | NEG | POS | NEG |
| Dresden 07_8788 | | | POS | POS | POS | POS | POS | POS | NEG | POS | NEG | POS | NEG | NEG | NEG | POS | NEG |
| Dresden 07_9245 | | | POS | POS | POS | POS | POS | POS | NEG | POS | NEG | POS | NEG | NEG | NEG | POS | NEG |
| Dresden 08_0217 | 12 | | POS | POS | POS | POS | POS | POS | NEG | POS | NEG | POS | NEG | NEG | NEG | POS | NEG |
| Dresden 08_0219 | | | POS | POS | POS | POS | POS | POS | NEG | POS | NEG | POS | NEG | NEG | NEG | POS | NEG |
| Dresden 08_0419 | | | POS | POS | POS | POS | POS | POS | NEG | POS | NEG | POS | NEG | NEG | NEG | POS | NEG |
| Dresden 08_0484 | | | POS | POS | POS | POS | POS | POS | NEG | POS | NEG | POS | NEG | NEG | NEG | POS | NEG |
| Dresden 08_0994 | | | POS | POS | POS | POS | POS | POS | NEG | POS | NEG | POS | NEG | NEG | NEG | POS | NEG |
| Dresden 08_1148 | | | POS | POS | POS | POS | POS | POS | NEG | POS | NEG | POS | NEG | NEG | NEG | POS | NEG |
| Dresden 08_1188 | | | POS | POS | POS | POS | POS | POS | NEG | POS | NEG | POS | NEG | NEG | NEG | POS | NEG |
| Dresden 08_1205 | | | POS | POS | POS | POS | POS | POS | NEG | POS | NEG | POS | NEG | NEG | NEG | POS | NEG |
| Dresden 08_1541 | | | POS | POS | POS | POS | POS | POS | NEG | POS | NEG | POS | NEG | NEG | NEG | POS | NEG |
| Dresden 06_7896 | | | POS | POS | AMB | AMB | POS | POS | NEG | AMB | NEG | POS | NEG | NEG | NEG | POS | NEG |
| **>HP-25** | | | POS | POS | POS | POS | POS | POS | NEG | POS | NEG | POS | NEG | NEG | NEG | POS | NEG |
| Freiburg-809-24.809-DBK0000287 | 46 | 35 | POS | POS | POS | POS | POS | POS | NEG | POS | NEG | POS | NEG | NEG | NEG | POS | NEG |
| | | | POS | POS | POS | POS | POS | POS | NEG | POS | NEG | POS | NEG | NEG | NEG | POS | NEG |
| Freiburg-990-64.99-DBK0000264 | 46 | | POS | POS | POS | POS | POS | POS | NEG | POS | NEG | POS | NEG | NEG | NEG | POS | NEG |
| | | | POS | POS | POS | POS | POS | POS | NEG | POS | NEG | POS | NEG | NEG | NEG | POS | NEG |
| Dresden 07_7952 | | | POS | POS | POS | POS | POS | POS | NEG | POS | NEG | POS | NEG | NEG | NEG | POS | NEG |
| Dresden 07_8955 | | | POS | POS | POS | POS | POS | POS | NEG | POS | NEG | POS | NEG | NEG | NEG | POS | NEG |
| Dresden 07_8958 | | | POS | POS | POS | POS | POS | POS | NEG | POS | NEG | POS | NEG | NEG | NEG | POS | NEG |
| Dresden 07_9027 | | | POS | POS | POS | POS | POS | POS | NEG | POS | NEG | POS | NEG | NEG | NEG | POS | NEG |
| Dresden 08_1169 | | | POS | POS | POS | POS | POS | POS | NEG | POS | NEG | POS | NEG | NEG | NEG | POS | NEG |
| **>HP-26** | | | NEG | NEG | NEG | NEG | NEG | NEG | NEG | NEG | NEG | NEG | NEG | NEG | NEG | NEG | NEG |
| Freiburg-635-3.635-DBK0000297 | 39 | | NEG | NEG | NEG | NEG | NEG | NEG | NEG | NEG | NEG | NEG | NEG | NEG | NEG | NEG | NEG |
| | | | NEG | NEG | NEG | NEG | NEG | NEG | NEG | NEG | NEG | NEG | NEG | NEG | NEG | NEG | NEG |
| Freiburg-895-38.895-DBK0000290 | 39 | | NEG | NEG | NEG | NEG | NEG | NEG | NEG | NEG | NEG | NEG | NEG | NEG | NEG | NEG | NEG |
| | | | NEG | NEG | NEG | NEG | NEG | NEG | NEG | NEG | NEG | NEG | NEG | NEG | NEG | NEG | NEG |
| **>HP-27** | | | NEG | NEG | NEG | NEG | NEG | NEG | NEG | NEG | NEG | NEG | NEG | NEG | NEG | NEG | NEG |
| Freiburg-726-14.726-DBK0000228 | 10 | | NEG | NEG | NEG | NEG | NEG | NEG | NEG | NEG | NEG | NEG | NEG | NEG | NEG | NEG | NEG |
| | | | NEG | NEG | NEG | NEG | NEG | NEG | NEG | NEG | NEG | NEG | NEG | NEG | NEG | NEG | NEG |
| Freiburg-837-27.837-DBK0000285 | 10 | | NEG | NEG | NEG | NEG | NEG | NEG | NEG | NEG | NEG | NEG | NEG | NEG | NEG | NEG | NEG |
| | | | NEG | NEG | NEG | NEG | NEG | NEG | NEG | NEG | NEG | NEG | NEG | NEG | NEG | NEG | NEG |
| **>HP-28** | | | POS | POS | NEG | POS | POS | POS | NEG | POS | NEG | POS | NEG | NEG | NEG | POS | NEG |
| C. difficile 6407 (GenBank ADEH) in silico prediction | | 58? | POS | POS | NEG | POS | POS | POS | NEG | POS | NEG | POS | NEG | NEG | NEG | POS | NEG |
| **>HP-29** | | | NEG | NEG | NEG | NEG | NEG | NEG | NEG | NEG | NEG | NEG | NEG | NEG | NEG | NEG | NEG |
| Freiburg-852-29.852-DBK0000279 | 71 | | NEG | NEG | NEG | NEG | NEG | NEG | NEG | NEG | NEG | NEG | NEG | NEG | NEG | NEG | NEG |
| | | | NEG | NEG | NEG | NEG | NEG | NEG | NEG | NEG | NEG | NEG | NEG | NEG | NEG | NEG | NEG |
| Freiburg-858-32.858-DBK0000222 | 71 | | NEG | NEG | NEG | NEG | NEG | NEG | NEG | NEG | NEG | NEG | NEG | NEG | NEG | NEG | NEG |
| **>HP-30** | | | POS | POS | POS | POS | POS | POS | NEG | POS | NEG | POS | NEG | NEG | NEG | POS | NEG |
| Freiburg-1047-76.1047-DBK0000269 | 81 | | POS | POS | POS | POS | POS | POS | NEG | POS | NEG | POS | NEG | NEG | NEG | POS | NEG |
| | | | POS | POS | POS | POS | POS | POS | NEG | POS | NEG | POS | NEG | NEG | NEG | POS | NEG |
| Freiburg-999-66.999-DBK0000262 | 94 | | POS | POS | POS | POS | POS | POS | NEG | POS | NEG | POS | NEG | NEG | NEG | POS | NEG |
| | 81 | | POS | POS | POS | POS | POS | POS | NEG | POS | NEG | POS | NEG | NEG | NEG | POS | NEG |
| Freiburg-896-39.896-DBK0000291 | 81 | | POS | POS | POS | POS | POS | POS | NEG | POS | NEG | POS | NEG | NEG | NEG | POS | NEG |
| | | | POS | POS | POS | POS | POS | POS | NEG | POS | NEG | POS | NEG | NEG | NEG | POS | NEG |
| Freiburg-871-33.871-DBK0000272 | 29 | 9 | POS | POS | POS | POS | POS | POS | NEG | POS | NEG | POS | NEG | NEG | NEG | POS | NEG |
| | | | POS | POS | POS | POS | POS | POS | NEG | POS | NEG | POS | NEG | NEG | NEG | POS | NEG |
| Dresden 08_1596 | | | POS | POS | POS | POS | POS | POS | NEG | POS | NEG | POS | NEG | NEG | NEG | POS | NEG |

| STRAIN / ISOLATE | Ribotype | Sequence type | Toxin A (total) | Toxin A Individual Probes | | | | Toxin B (total) | Toxin B Individual Probes | | | | | | | | |
|---|---|---|---|---|---|---|---|---|---|---|---|---|---|---|---|---|---|
| | | | | hp1132\|tcdAp1 | hp1134\|tcdAp2 | hp1135\|tcdAp3 | hp1247\|tcdAp2 | | hp1118\|tcdBp1 | hp1119\|tcdBp1 | hp1121\|tcdBp4 | hp1122\|tcdBp4 | hp1124\|tcdBp2 | hp1126\|tcdBp2 | hp1127\|tcdBp2 | hp1129\|tcdBp3 | hp1130\|tcdBp3 |
| **CLADE 2 (CC1 /Ribo 27 Complex)** | | | | | | | | | | | | | | | | | |
| **>HP-31** | | | POS | POS | POS | POS | POS | POS | NEG | POS | POS | NEG | NEG | POS | NEG | NEG | POS |
| C. difficile BI1 (GenBank FN668941) in silico prediction | | 1 | POS | POS | POS | POS | POS | POS | NEG | POS | POS | NEG | NEG | POS | NEG | NEG | POS |
| C. difficile QCD-97b34 (GenBank ABHF) in silico prediction | | 1 | POS | POS | POS | POS | POS | POS | NEG | POS | POS | NEG | NEG | POS | NEG | NEG | POS |
| C. difficile QCD-76w55 (GenBank ABHE) in silico prediction | | 1 | POS | POS | POS | POS | POS | POS | NEG | POS | POS | NEG | NEG | POS | NEG | NEG | POS |
| C. difficile CIP107932 (GenBank ABKK) in silico prediction | | 1 | POS | POS | POS | POS | POS | POS | NEG | POS | POS | NEG | NEG | POS | NEG | NEG | POS |
| C. difficile CD196 (GenBank FN538970) in silico prediction | | 1 | POS | POS | POS | POS | POS | POS | NEG | POS | POS | NEG | NEG | POS | NEG | NEG | POS |
| C. difficile Strain CD196 | 27 | 1 | POS | POS | POS | POS | POS | POS | NEG | POS | POS | NEG | NEG | NEG | NEG | NEG | POS |
| | | | POS | POS | POS | POS | POS | POS | NEG | POS | POS | NEG | NEG | AMB | NEG | NEG | POS |
| | | | POS | POS | POS | POS | POS | POS | NEG | POS | POS | NEG | NEG | NEG | NEG | NEG | POS |
| | | | POS | POS | POS | POS | POS | POS | NEG | POS | POS | NEG | NEG | AMB | NEG | NEG | POS |
| | | | POS | POS | POS | POS | POS | POS | NEG | POS | POS | NEG | NEG | AMB | NEG | NEG | POS |
| Dresden 07-7829 | 27 | | POS | POS | POS | POS | POS | POS | NEG | POS | POS | NEG | NEG | AMB | NEG | NEG | POS |
| Dresden 07-8176 | | | POS | POS | POS | POS | POS | POS | NEG | POS | POS | NEG | NEG | POS | NEG | NEG | POS |
| **>HP-32** | | | POS | POS | POS | POS | POS | POS | NEG | POS | POS | NEG | NEG | POS | NEG | NEG | POS |
| C. difficile 2007855 (GenBank FN665654) in silico prediction | | 1 | POS | POS | POS | POS | POS | POS | NEG | POS | POS | NEG | NEG | POS | NEG | NEG | POS |
| C. difficile QCD-32g58 (GenBank AAML) in silico prediction | | 1 | POS | POS | POS | POS | POS | POS | NEG | POS | POS | NEG | NEG | POS | NEG | NEG | POS |
| C. difficile QCD-37x79 (GenBank ABHG) in silico prediction | | 1 | POS | POS | POS | POS | POS | POS | NEG | POS | POS | NEG | NEG | POS | NEG | NEG | POS |
| C. difficile QCD-66c26 (GenBank ABFD) in silico prediction | | 1 | POS | POS | POS | POS | POS | POS | NEG | POS | POS | NEG | NEG | POS | NEG | NEG | POS |
| C. difficile R20291 (GenBank FN545816) in silico prediction | | 1 | POS | POS | POS | POS | POS | POS | NEG | POS | POS | NEG | NEG | POS | NEG | NEG | POS |
| C. difficile Strain R20291 | 27 | 1 | POS | POS | POS | POS | POS | POS | NEG | POS | POS | NEG | NEG | NEG | NEG | NEG | POS |
| | | | POS | POS | POS | POS | POS | POS | NEG | POS | POS | NEG | NEG | AMB | NEG | NEG | POS |
| | | | POS | POS | POS | POS | POS | POS | NEG | POS | POS | NEG | NEG | AMB | NEG | NEG | POS |
| | | | POS | POS | POS | POS | POS | POS | NEG | POS | POS | NEG | NEG | AMB | NEG | NEG | POS |
| **CLADE 3 (CC5 /Ribo 23 Complex)** | | | | | | | | | | | | | | | | | |
| **>HP-33** | | | POS | POS | POS | POS | POS | POS | NEG | POS | NEG | POS | NEG | NEG | NEG | POS | NEG |
| Freiburg872-34.872-DBK0000295 | 23 | | POS | POS | POS | POS | POS | POS | NEG | POS | NEG | POS | NEG | NEG | NEG | POS | NEG |
| | | | POS | POS | POS | POS | POS | POS | NEG | POS | NEG | POS | NEG | NEG | NEG | POS | NEG |
| Freiburg-773-19.773-DBK0000274 | 23 | | POS | POS | POS | POS | POS | POS | NEG | POS | NEG | POS | NEG | NEG | NEG | POS | NEG |
| | | | POS | POS | POS | POS | POS | POS | NEG | POS | NEG | POS | NEG | NEG | NEG | POS | NEG |
| **>HP-34** | | | POS | POS | POS | POS | POS | POS | NEG | POS | NEG | POS | NEG | NEG | NEG | POS | NEG |
| Jena_0018/11 | | | POS | POS | POS | POS | POS | POS | NEG | POS | NEG | POS | NEG | NEG | NEG | POS | NEG |
| **CLADE 4 (CC37/Ribo 17 Complex)** | | | | | | | | | | | | | | | | | |
| **>HP-35** | | | POS | POS | NEG | POS | POS | POS | POS | NEG | NEG | POS | NEG | NEG | POS | POS | NEG |
| C. difficile 002-P50-2011 (GenBank AGAA) in silico prediction | | 37 | POS | POS | NEG | POS | POS | POS | POS | NEG | NEG | POS | NEG | NEG | POS | POS | NEG |
| C. difficile 050-P50-2011 (GenBank AGAB) in silico prediction | | 37 | POS | POS | NEG | POS | POS | POS | POS | NEG | NEG | POS | NEG | NEG | POS | POS | NEG |
| C. difficile M68 (GenBank FN668375) in silico prediction | 17 | 37 | POS | POS | NEG | POS | POS | POS | POS | NEG | NEG | POS | NEG | NEG | POS | POS | NEG |
| C. difficile CF5 (GenBank FN665652) in silico prediction | | 86 | POS | POS | NEG | POS | POS | POS | POS | NEG | NEG | POS | NEG | NEG | POS | POS | NEG |
| C. difficile Strain CF5 | 17 | | POS | POS | NEG | POS | POS | POS | POS | NEG | NEG | POS | NEG | NEG | POS | POS | NEG |
| | | | POS | POS | NEG | POS | POS | POS | POS | NEG | NEG | POS | NEG | NEG | POS | POS | NEG |
| | | | POS | POS | NEG | POS | POS | POS | POS | NEG | NEG | POS | NEG | NEG | POS | POS | NEG |
| | | | POS | POS | NEG | POS | POS | POS | POS | NEG | NEG | POS | NEG | NEG | POS | POS | NEG |
| | | | POS | POS | NEG | POS | POS | POS | POS | NEG | NEG | POS | NEG | NEG | POS | POS | NEG |
| | | | POS | POS | NEG | POS | POS | POS | POS | NEG | NEG | POS | NEG | NEG | POS | POS | NEG |
| Freiburg-736-16.736-DBK0000233 | 17 | | POS | POS | NEG | POS | POS | POS | POS | NEG | NEG | POS | NEG | NEG | POS | POS | NEG |
| | | | POS | POS | NEG | POS | POS | POS | POS | NEG | NEG | POS | NEG | NEG | POS | POS | NEG |
| **>HP-36** | | | POS | POS | NEG | POS | POS | POS | POS | NEG | NEG | POS | NEG | NEG | POS | POS | NEG |
| Freiburg-642-5.642-DBK0000281 | 17 | | POS | POS | NEG | POS | POS | POS | POS | NEG | NEG | POS | NEG | NEG | POS | POS | NEG |
| | | | POS | POS | NEG | POS | POS | POS | POS | NEG | NEG | POS | NEG | NEG | POS | POS | NEG |
| **CLADE 5 (CC11 /Ribo 78 Complex)** | | | | | | | | | | | | | | | | | |
| **>HP-37** | | | POS | POS | POS | POS | POS | POS | NEG | POS | NEG | POS | NEG | NEG | NEG | POS | NEG |
| C. difficile NAP08 (GenBank ADNX) in silico prediction | | | POS | POS | POS | POS | POS | POS | NEG | POS | NEG | POS | NEG | NEG | NEG | POS | NEG |
| C. difficile NAP07 (GenBank ADVM) in silico prediction | | | POS | POS | POS | POS | POS | POS | NEG | POS | NEG | POS | NEG | NEG | NEG | POS | NEG |
| C. difficile M120 (GenBank FN665653) in silico prediction | | 11 | POS | POS | POS | POS | POS | POS | NEG | POS | NEG | POS | NEG | NEG | NEG | POS | NEG |
| C. difficile Strain M120 | 78 | | POS | POS | POS | POS | POS | POS | NEG | POS | NEG | POS | NEG | NEG | NEG | POS | NEG |
| | | | POS | POS | POS | POS | POS | POS | NEG | POS | NEG | POS | NEG | NEG | NEG | POS | NEG |
| | | | POS | POS | POS | POS | POS | POS | NEG | POS | NEG | POS | NEG | NEG | NEG | POS | NEG |
| | | | POS | POS | POS | POS | POS | POS | NEG | POS | NEG | POS | NEG | NEG | NEG | POS | NEG |
| | | | POS | POS | POS | POS | POS | POS | NEG | POS | NEG | POS | NEG | NEG | NEG | POS | NEG |
| Freiburg_69.1017 | | | POS | POS | POS | POS | POS | POS | NEG | POS | NEG | POS | NEG | NEG | NEG | POS | NEG |
| Freiburg-1051-77.1051-DBK0000293 | 78 | 11 | POS | POS | POS | POS | POS | POS | NEG | POS | NEG | POS | NEG | NEG | NEG | POS | NEG |
| | | | POS | POS | POS | POS | POS | POS | NEG | POS | NEG | POS | NEG | NEG | NEG | POS | NEG |
| Freiburg-649-6.649-DBK0000235 | 78 | | POS | POS | POS | POS | POS | POS | NEG | POS | NEG | POS | NEG | NEG | NEG | POS | NEG |
| | | | POS | POS | POS | POS | POS | POS | NEG | POS | NEG | POS | NEG | NEG | NEG | POS | NEG |
| Freiburg-700-11.7-DBK0000242 | 78 | | POS | POS | POS | POS | POS | POS | NEG | POS | NEG | POS | NEG | NEG | NEG | POS | NEG |
| | | | POS | POS | POS | POS | POS | POS | NEG | POS | NEG | POS | NEG | NEG | NEG | POS | NEG |
| Freiburg-855-30.855-DBK0000277 | 78 | | POS | POS | POS | POS | POS | POS | NEG | POS | NEG | POS | NEG | NEG | NEG | POS | NEG |
| | | | POS | POS | POS | POS | POS | POS | NEG | POS | NEG | POS | NEG | NEG | NEG | POS | NEG |
| Dresden 08_1353 | | | POS | POS | POS | POS | POS | POS | NEG | POS | NEG | POS | NEG | NEG | NEG | POS | NEG |
| Dresden 08_574 | | | POS | POS | POS | POS | POS | POS | NEG | POS | NEG | POS | NEG | NEG | NEG | POS | NEG |
| Dresden 09_2650 | | | POS | POS | POS | POS | POS | POS | NEG | POS | NEG | POS | NEG | NEG | NEG | POS | NEG |
| **>HP-38** | | | POS | POS | POS | POS | POS | POS | NEG | POS | NEG | POS | NEG | NEG | NEG | POS | NEG |
| C. difficile 6466 (GenBank ADDE) in silico prediction | | 11 (1MM) | POS | POS | POS | POS | POS | POS | NEG | POS | NEG | POS | NEG | NEG | NEG | POS | NEG |
| **>HP-39** | | | POS | POS | POS | POS | POS | POS | NEG | POS | NEG | POS | NEG | NEG | NEG | POS | NEG |
| C. difficile QCD-23m63 (GenBank ABKL) in silico prediction | | 11 (1MM) | POS | POS | POS | POS | POS | POS | NEG | POS | NEG | POS | NEG | NEG | NEG | POS | NEG |
| Freiburg-956-51.956-DBK0000266 | | | POS | POS | POS | POS | POS | POS | NEG | POS | NEG | POS | NEG | NEG | NEG | POS | NEG |
| Freiburg_50.963 | | | POS | POS | POS | POS | POS | POS | NEG | POS | NEG | POS | NEG | NEG | NEG | POS | NEG |
| **"CLADE 6"** | | | | | | | | | | | | | | | | | |
| **>HP-40** | | | NEG | NEG | NEG | NEG | NEG | NEG | NEG | NEG | NEG | NEG | NEG | NEG | NEG | NEG | NEG |
| C. difficile 6503 (GenBank ADEI) in silico prediction | | CC127, 11-7-10-14-6-20-15, 2 MM | NEG | NEG | NEG | NEG | NEG | NEG | NEG | NEG | NEG | NEG | NEG | NEG | NEG | NEG | NEG |

| STRAIN / ISOLATE | chloramphenicol acetyl transferase (cat) | rRNA methyl-transferase (ermB) | tetM (total) | tetracycline resistance protein, individual probes | | | | | streptogramin A acetyltransferase | | lincomycin resist. protein lmrBNAP07 | lincomycin resist. protein lmrB630 | lantibiotic two-component sensor histidine kinase | | putative lantibiotic ABC transporter, permease protein | | bacitracin resistance protein locus 1 | bacitracin ATP binding cassette transporter, ABC protein BcrA | | | ABC-type transport system, ATP-binding protein | putative efflux pump | MATE efflux family protein | efflux pump antibiotic resistance protein | | ABC-type transport system, permease Tn1549 | | two-component sensor histidine kinase Tn154c | |
|---|---|---|---|---|---|---|---|---|---|---|---|---|---|---|---|---|---|---|---|---|---|---|---|---|---|---|---|---|---|
| | hp1014\|catD | hp1022\|ermB | | hp1105\|tetM | hp1108\|tetM | hp1109\|tetM | hp1110\|tetM | hp1112\|tetM | hp1082\|sat | hp1083\|sat | hp1096\|lmrB | hp1097\|lmrB | hp1079\|spaK | hp1080\|spaK | hp1251\|spaE | hp1252\|spaE | hp1013\|bacA1 | hp1071\|bcrA | hp1072\|bcrA | hp1073\|bcrA | hp1084\|CD0456 | hp1087\|cme | hp1063\|matE | hp1098\|ydiC | hp1244\|ydiC | hp1055\|vexP1 | hp1059\|vexP1 | hp1051\|vncS | hp1052\|vncS |
| **CLADE 1** | | | | | | | | | | | | | | | | | | | | | | | | | | | | | |
| **>HP-01** | NEG | VAR | NEG | NEG | NEG | NEG | NEG | NEG | POS | NEG | NEG | POS | AMB | POS | POS | NEG | POS | NEG | POS | NEG | NEG | POS | NEG | NEG | POS | NEG | POS | NEG | POS |
| C. difficile QCD-63q42 (GenBank ABHD) in silico prediction | NEG | NEG | NEG | NEG | NEG | NEG | NEG | NEG | POS | NEG | NEG | POS | NEG | POS | POS | NEG | POS | NEG | POS | NEG | NEG | POS | NEG | NEG | POS | NEG | POS | NEG | POS |
| C. difficile BI9 (GenBank FN668944) in silico prediction | NEG | POS | NEG | NEG | NEG | NEG | NEG | NEG | POS | NEG | NEG | POS | NEG | POS | POS | NEG | POS | NEG | POS | NEG | NEG | POS | NEG | NEG | POS | NEG | POS | NEG | POS |
| C. difficile BI-9 | NEG | POS | NEG | NEG | NEG | NEG | NEG | NEG | POS | NEG | NEG | POS | POS | POS | POS | NEG | POS | NEG | POS | NEG | NEG | POS | NEG | NEG | POS | NEG | POS | NEG | POS |
| | NEG | POS | NEG | NEG | NEG | NEG | NEG | NEG | POS | NEG | NEG | POS | NEG | POS | POS | NEG | POS | NEG | POS | NEG | NEG | POS | NEG | NEG | POS | NEG | POS | NEG | POS |
| | NEG | POS | NEG | NEG | NEG | NEG | NEG | NEG | POS | NEG | NEG | POS | NEG | POS | POS | POS | POS | AMB | POS | NEG | NEG | POS | NEG | NEG | POS | NEG | POS | NEG | POS |
| | NEG | POS | NEG | NEG | NEG | NEG | NEG | NEG | POS | NEG | NEG | POS | NEG | POS | POS | NEG | POS | NEG | POS | NEG | NEG | POS | NEG | NEG | POS | NEG | POS | NEG | POS |
| | NEG | POS | NEG | NEG | NEG | NEG | NEG | NEG | POS | NEG | NEG | POS | NEG | POS | POS | NEG | POS | NEG | POS | NEG | NEG | POS | NEG | NEG | POS | NEG | POS | NEG | POS |
| Freiburg-1040-73.104-DBK0000249 | NEG | POS | NEG | NEG | NEG | NEG | NEG | NEG | POS | NEG | NEG | POS | NEG | POS | POS | AMB | POS | NEG | POS | NEG | NEG | POS | NEG | NEG | POS | NEG | POS | NEG | POS |
| | NEG | POS | NEG | NEG | NEG | NEG | NEG | NEG | POS | NEG | NEG | POS | NEG | POS | POS | NEG | POS | NEG | POS | NEG | NEG | POS | NEG | NEG | POS | NEG | POS | NEG | POS |
| Freiburg-1052-78.1052-DBK0000294 | NEG | POS | NEG | NEG | NEG | NEG | NEG | NEG | POS | NEG | NEG | POS | NEG | POS | POS | NEG | POS | NEG | POS | NEG | NEG | POS | NEG | NEG | POS | NEG | POS | NEG | POS |
| | NEG | POS | NEG | NEG | NEG | NEG | NEG | NEG | POS | NEG | NEG | POS | NEG | POS | POS | NEG | POS | NEG | POS | NEG | NEG | POS | NEG | NEG | POS | NEG | POS | NEG | POS |
| Freiburg-605-1.605-DBK0000223 | NEG | NEG | NEG | NEG | NEG | NEG | NEG | NEG | POS | NEG | NEG | POS | AMB | POS | POS | AMB | POS | NEG | POS | NEG | NEG | POS | NEG | NEG | POS | NEG | POS | NEG | POS |
| Freiburg-787-22.787-DBK0000288 | NEG | NEG | NEG | NEG | NEG | NEG | NEG | NEG | POS | NEG | NEG | POS | NEG | POS | POS | NEG | POS | NEG | POS | NEG | NEG | POS | NEG | NEG | POS | NEG | POS | NEG | POS |
| | NEG | NEG | NEG | NEG | NEG | NEG | NEG | NEG | POS | NEG | NEG | POS | NEG | POS | POS | NEG | POS | NEG | POS | NEG | NEG | POS | NEG | NEG | POS | NEG | POS | NEG | POS |
| Freiburg-897-40.897-DBK0000225 | NEG | NEG | NEG | NEG | NEG | NEG | NEG | NEG | POS | NEG | NEG | POS | AMB | POS | POS | AMB | POS | NEG | POS | NEG | NEG | POS | NEG | NEG | POS | NEG | POS | NEG | POS |
| | NEG | NEG | NEG | NEG | NEG | NEG | NEG | NEG | POS | NEG | NEG | POS | NEG | POS | POS | NEG | POS | NEG | POS | NEG | NEG | POS | NEG | NEG | POS | NEG | POS | NEG | AMB |
| Dresden 06_7735 | NEG | POS | NEG | NEG | NEG | NEG | NEG | NEG | POS | NEG | NEG | POS | AMB | POS | POS | NEG | POS | NEG | POS | NEG | NEG | POS | NEG | NEG | POS | NEG | POS | NEG | POS |
| Dresden 06_7736 | NEG | POS | NEG | NEG | NEG | NEG | NEG | NEG | POS | NEG | NEG | POS | AMB | AMB | POS | NEG | POS | NEG | POS | NEG | NEG | POS | NEG | NEG | POS | NEG | POS | NEG | AMB |
| Dresden 07_6943 | NEG | POS | NEG | NEG | NEG | NEG | NEG | NEG | POS | NEG | NEG | POS | AMB | POS | POS | AMB | POS | NEG | POS | NEG | NEG | POS | NEG | NEG | POS | NEG | POS | NEG | POS |
| Dresden 07_7200 | NEG | NEG | NEG | NEG | NEG | NEG | NEG | NEG | POS | NEG | NEG | POS | AMB | NEG | POS | NEG | POS | NEG | POS | NEG | NEG | AMB | NEG | NEG | POS | NEG | POS | NEG | NEG |
| Dresden 07_7269 | NEG | POS | NEG | NEG | NEG | NEG | NEG | NEG | POS | NEG | NEG | POS | NEG | POS | POS | NEG | POS | NEG | POS | NEG | NEG | POS | NEG | NEG | POS | NEG | POS | NEG | POS |
| Dresden 07_7678 | NEG | NEG | NEG | NEG | NEG | NEG | NEG | NEG | POS | NEG | NEG | POS | POS | POS | POS | NEG | POS | NEG | POS | NEG | NEG | POS | NEG | NEG | POS | NEG | POS | NEG | POS |
| Dresden 07_7773 | NEG | NEG | NEG | NEG | NEG | NEG | NEG | NEG | POS | NEG | NEG | POS | NEG | POS | POS | NEG | POS | NEG | POS | NEG | NEG | POS | NEG | NEG | POS | NEG | POS | NEG | POS |
| Dresden 07_7832 | NEG | POS | NEG | NEG | NEG | NEG | NEG | NEG | POS | NEG | NEG | POS | NEG | POS | POS | NEG | POS | NEG | POS | NEG | NEG | POS | NEG | NEG | POS | NEG | POS | NEG | POS |
| Dresden 07_7967 | NEG | POS | NEG | NEG | NEG | NEG | NEG | NEG | POS | NEG | NEG | POS | AMB | POS | POS | NEG | POS | NEG | POS | NEG | NEG | POS | NEG | NEG | POS | NEG | POS | NEG | POS |
| Dresden 07_8099 | NEG | POS | NEG | NEG | NEG | NEG | NEG | NEG | POS | NEG | NEG | POS | NEG | POS | POS | AMB | POS | NEG | POS | NEG | NEG | POS | NEG | NEG | POS | NEG | POS | NEG | POS |
| Dresden 07_8100 | NEG | POS | NEG | NEG | NEG | NEG | NEG | NEG | POS | NEG | NEG | POS | NEG | POS | POS | NEG | POS | NEG | POS | NEG | NEG | POS | NEG | NEG | POS | NEG | POS | NEG | POS |
| Dresden 07_8170 | NEG | POS | NEG | NEG | NEG | NEG | NEG | NEG | POS | NEG | NEG | POS | POS | POS | POS | NEG | POS | NEG | POS | NEG | NEG | POS | NEG | NEG | POS | NEG | POS | NEG | POS |
| Dresden 08_0544 | NEG | POS | NEG | NEG | NEG | NEG | NEG | NEG | POS | NEG | NEG | POS | AMB | POS | POS | NEG | POS | NEG | POS | NEG | NEG | POS | NEG | NEG | POS | NEG | POS | NEG | POS |
| Dresden 08_0550 | NEG | POS | NEG | NEG | NEG | NEG | NEG | NEG | POS | NEG | NEG | POS | AMB | POS | POS | NEG | POS | NEG | POS | NEG | NEG | POS | NEG | NEG | POS | NEG | POS | NEG | POS |
| Dresden 08_0827 | NEG | NEG | NEG | NEG | NEG | NEG | NEG | NEG | POS | NEG | NEG | POS | AMB | POS | POS | NEG | POS | NEG | POS | NEG | NEG | POS | NEG | NEG | POS | NEG | POS | NEG | POS |
| Dresden 08_0902 | NEG | POS | NEG | NEG | NEG | NEG | NEG | NEG | POS | NEG | NEG | POS | AMB | POS | POS | NEG | POS | NEG | POS | NEG | NEG | POS | NEG | NEG | POS | NEG | POS | NEG | POS |
| Dresden 08_0955 | NEG | POS | NEG | NEG | NEG | NEG | NEG | NEG | POS | NEG | NEG | POS | AMB | POS | POS | NEG | POS | NEG | POS | NEG | NEG | POS | NEG | NEG | POS | NEG | POS | NEG | POS |
| Dresden 08_0958 | NEG | NEG | NEG | NEG | NEG | NEG | NEG | NEG | POS | NEG | NEG | POS | AMB | POS | POS | NEG | POS | NEG | POS | NEG | NEG | POS | NEG | NEG | POS | NEG | POS | NEG | POS |
| Dresden 08_1073 | NEG | NEG | NEG | NEG | NEG | NEG | NEG | NEG | POS | NEG | NEG | POS | AMB | POS | POS | NEG | POS | NEG | POS | NEG | NEG | POS | NEG | NEG | POS | NEG | POS | NEG | POS |
| Dresden 08_1074 | NEG | NEG | NEG | NEG | NEG | NEG | NEG | NEG | POS | NEG | NEG | POS | AMB | POS | POS | NEG | POS | NEG | POS | NEG | NEG | POS | NEG | NEG | POS | NEG | POS | NEG | POS |
| Dresden 08_1206 | NEG | NEG | NEG | NEG | NEG | NEG | NEG | NEG | POS | NEG | NEG | POS | AMB | POS | POS | NEG | POS | NEG | POS | NEG | NEG | POS | NEG | NEG | POS | NEG | POS | NEG | POS |
| Dresden 08_1216 | NEG | POS | NEG | NEG | NEG | NEG | NEG | NEG | POS | NEG | NEG | POS | AMB | POS | POS | NEG | POS | NEG | POS | NEG | NEG | POS | NEG | NEG | POS | NEG | POS | NEG | POS |
| Dresden 08_1305 | NEG | POS | NEG | NEG | NEG | NEG | NEG | NEG | POS | NEG | NEG | POS | AMB | POS | POS | NEG | POS | NEG | POS | NEG | NEG | POS | NEG | NEG | POS | NEG | POS | NEG | POS |
| Dresden 07_7679 | NEG | POS | NEG | NEG | NEG | NEG | NEG | NEG | POS | NEG | NEG | POS | NEG | POS | POS | NEG | POS | NEG | POS | NEG | NEG | AMB | NEG | NEG | POS | NEG | POS | NEG | POS |
| Dresden 07_7683 | NEG | POS | NEG | NEG | NEG | NEG | NEG | NEG | POS | NEG | NEG | POS | AMB | POS | POS | NEG | POS | NEG | POS | NEG | NEG | POS | NEG | NEG | POS | NEG | POS | NEG | POS |
| Dresden 07_7980 | NEG | NEG | NEG | NEG | NEG | NEG | NEG | NEG | POS | NEG | NEG | POS | AMB | POS | POS | NEG | POS | NEG | POS | NEG | NEG | POS | NEG | NEG | POS | NEG | POS | NEG | POS |
| Dresden 07_8022 | NEG | POS | NEG | NEG | NEG | NEG | NEG | NEG | POS | NEG | NEG | POS | NEG | POS | POS | NEG | POS | NEG | POS | NEG | NEG | POS | NEG | NEG | POS | NEG | POS | NEG | POS |
| Dresden 07_8291 | NEG | POS | NEG | NEG | NEG | NEG | NEG | NEG | POS | NEG | NEG | POS | AMB | AMB | POS | NEG | POS | NEG | POS | NEG | NEG | AMB | NEG | NEG | POS | NEG | POS | NEG | AMB |
| Dresden 07_8314 | NEG | POS | NEG | NEG | NEG | NEG | NEG | NEG | POS | NEG | NEG | POS | NEG | POS | POS | AMB | POS | NEG | POS | NEG | NEG | POS | NEG | NEG | POS | NEG | POS | NEG | POS |
| Dresden 07_8315 | NEG | POS | NEG | NEG | NEG | NEG | NEG | NEG | POS | NEG | NEG | POS | AMB | POS | POS | NEG | POS | NEG | POS | NEG | NEG | POS | NEG | NEG | POS | NEG | POS | NEG | POS |
| Dresden 07_8323 | NEG | POS | NEG | NEG | NEG | NEG | NEG | NEG | POS | NEG | NEG | POS | AMB | POS | POS | NEG | POS | NEG | POS | NEG | NEG | AMB | NEG | NEG | POS | NEG | POS | NEG | AMB |
| Dresden 07_8356 | NEG | POS | NEG | NEG | NEG | NEG | NEG | NEG | POS | NEG | NEG | POS | NEG | POS | POS | NEG | POS | NEG | POS | NEG | NEG | POS | NEG | NEG | POS | NEG | POS | NEG | POS |
| Dresden 07_8557 | NEG | POS | NEG | NEG | NEG | NEG | NEG | NEG | POS | NEG | NEG | POS | NEG | AMB | POS | NEG | POS | NEG | POS | NEG | NEG | AMB | NEG | NEG | POS | NEG | POS | NEG | AMB |
| Dresden 07_8736 | NEG | POS | NEG | NEG | NEG | NEG | NEG | NEG | POS | NEG | NEG | POS | NEG | POS | POS | NEG | POS | NEG | POS | NEG | NEG | POS | NEG | NEG | POS | NEG | POS | NEG | POS |
| Dresden 07_8945 | NEG | POS | NEG | NEG | NEG | NEG | NEG | NEG | POS | NEG | NEG | POS | AMB | POS | POS | NEG | POS | NEG | POS | NEG | NEG | POS | NEG | NEG | POS | NEG | POS | NEG | POS |
| Dresden 07_9138 | NEG | POS | NEG | NEG | NEG | NEG | NEG | NEG | POS | NEG | NEG | POS | AMB | POS | POS | AMB | POS | NEG | POS | NEG | NEG | POS | NEG | NEG | POS | NEG | POS | NEG | POS |
| Dresden 07_9175 | NEG | NEG | NEG | NEG | NEG | NEG | NEG | NEG | POS | NEG | NEG | POS | NEG | POS | POS | NEG | POS | NEG | POS | NEG | NEG | POS | NEG | NEG | POS | NEG | POS | NEG | POS |
| Dresden 07_9234 | NEG | POS | NEG | NEG | NEG | NEG | NEG | NEG | POS | NEG | NEG | POS | NEG | POS | POS | NEG | POS | NEG | POS | NEG | NEG | POS | NEG | NEG | POS | NEG | POS | NEG | POS |
| Dresden 07_9255 | NEG | POS | NEG | NEG | NEG | NEG | NEG | NEG | POS | NEG | NEG | POS | NEG | POS | POS | NEG | POS | NEG | POS | NEG | NEG | POS | NEG | NEG | POS | NEG | POS | NEG | POS |
| Dresden 07_9309 | NEG | POS | NEG | NEG | NEG | NEG | NEG | NEG | POS | NEG | NEG | POS | NEG | POS | POS | NEG | POS | NEG | POS | NEG | NEG | POS | NEG | NEG | POS | NEG | POS | NEG | POS |
| Dresden 07_9330 | NEG | NEG | NEG | NEG | NEG | NEG | NEG | NEG | POS | NEG | NEG | POS | NEG | POS | POS | NEG | POS | NEG | POS | NEG | NEG | POS | NEG | NEG | POS | NEG | POS | NEG | POS |
| Dresden 07_9399 | NEG | POS | NEG | NEG | NEG | NEG | NEG | NEG | POS | NEG | NEG | POS | AMB | POS | POS | NEG | POS | NEG | POS | NEG | NEG | POS | NEG | NEG | POS | NEG | POS | NEG | POS |
| Dresden 08_0188 | NEG | POS | NEG | NEG | NEG | NEG | NEG | NEG | POS | NEG | NEG | POS | AMB | POS | POS | NEG | POS | NEG | POS | NEG | NEG | POS | NEG | NEG | POS | NEG | POS | NEG | POS |
| Dresden 08_0190 | NEG | NEG | NEG | NEG | NEG | NEG | NEG | NEG | POS | NEG | NEG | POS | NEG | POS | POS | NEG | POS | NEG | POS | NEG | NEG | POS | NEG | NEG | POS | NEG | POS | NEG | POS |
| Dresden 08_0220 | NEG | NEG | NEG | NEG | NEG | NEG | NEG | NEG | POS | NEG | NEG | POS | NEG | POS | POS | AMB | POS | NEG | POS | NEG | NEG | POS | NEG | NEG | POS | NEG | POS | NEG | POS |
| Dresden 08_0326 | NEG | POS | NEG | NEG | NEG | NEG | NEG | NEG | POS | NEG | NEG | POS | AMB | POS | POS | NEG | POS | NEG | POS | NEG | NEG | POS | NEG | NEG | POS | NEG | POS | NEG | POS |
| Dresden 08_0327 | NEG | POS | NEG | NEG | NEG | NEG | NEG | NEG | POS | NEG | NEG | POS | AMB | POS | POS | NEG | POS | NEG | POS | NEG | NEG | POS | NEG | NEG | POS | NEG | POS | NEG | POS |
| Dresden 08_0648 | NEG | POS | NEG | NEG | NEG | NEG | NEG | NEG | POS | NEG | NEG | POS | NEG | POS | POS | NEG | POS | NEG | POS | NEG | NEG | POS | NEG | NEG | POS | NEG | POS | NEG | POS |
| Dresden 08_0751 | NEG | NEG | NEG | NEG | NEG | NEG | NEG | NEG | POS | NEG | NEG | POS | NEG | POS | POS | NEG | POS | NEG | POS | NEG | NEG | POS | NEG | NEG | POS | NEG | POS | NEG | POS |
| Dresden 08_0769 | NEG | NEG | NEG | NEG | NEG | NEG | NEG | NEG | POS | NEG | NEG | POS | NEG | POS | POS | NEG | POS | NEG | POS | NEG | NEG | POS | NEG | NEG | POS | NEG | POS | NEG | POS |
| Dresden 08_0770 | NEG | NEG | NEG | NEG | NEG | NEG | NEG | NEG | POS | NEG | NEG | POS | NEG | POS | POS | NEG | POS | NEG | POS | NEG | NEG | POS | NEG | NEG | POS | NEG | POS | NEG | POS |
| Dresden 08_0771 | NEG | POS | NEG | NEG | NEG | NEG | NEG | NEG | POS | NEG | NEG | POS | NEG | POS | POS | NEG | POS | NEG | POS | NEG | NEG | POS | NEG | NEG | POS | NEG | POS | NEG | POS |
| Dresden 08_1308 | NEG | NEG | NEG | NEG | NEG | NEG | NEG | NEG | POS | NEG | NEG | POS | AMB | POS | POS | AMB | POS | NEG | POS | NEG | NEG | POS | NEG | NEG | POS | NEG | POS | NEG | POS |
| Dresden 08_1311 | NEG | NEG | NEG | NEG | NEG | NEG | NEG | NEG | POS | NEG | NEG | POS | AMB | POS | POS | NEG | POS | NEG | POS | NEG | NEG | POS | NEG | NEG | POS | NEG | POS | NEG | POS |
| Dresden 08_1316 | NEG | POS | NEG | NEG | NEG | NEG | NEG | NEG | POS | NEG | NEG | POS | AMB | POS | POS | NEG | POS | NEG | POS | NEG | NEG | AMB | NEG | NEG | POS | NEG | POS | NEG | POS |
| Dresden 08_1542 | NEG | POS | NEG | NEG | NEG | NEG | NEG | NEG | POS | NEG | NEG | POS | AMB | POS | POS | POS | POS | AMB | POS | NEG | NEG | POS | NEG | NEG | POS | NEG | POS | NEG | POS |
| Dresden 08_1544 | NEG | POS | NEG | NEG | NEG | NEG | NEG | NEG | POS | NEG | NEG | POS | AMB | POS | POS | AMB | POS | NEG | POS | NEG | NEG | POS | NEG | NEG | POS | NEG | POS | NEG | POS |
| Dresden 08_1581 | NEG | NEG | NEG | NEG | NEG | NEG | NEG | NEG | POS | NEG | NEG | POS | AMB | POS | POS | AMB | POS | AMB | POS | NEG | NEG | POS | NEG | NEG | POS | NEG | POS | NEG | POS |
| Dresden 08_1826 | NEG | NEG | NEG | NEG | NEG | NEG | NEG | NEG | POS | NEG | NEG | POS | AMB | POS | POS | NEG | POS | NEG | POS | NEG | NEG | POS | NEG | NEG | POS | NEG | POS | NEG | POS |
| Dresden 08_1817 | NEG | NEG | NEG | NEG | NEG | NEG | NEG | NEG | POS | NEG | NEG | POS | AMB | POS | POS | NEG | POS | NEG | POS | NEG | NEG | AMB | NEG | NEG | POS | NEG | POS | NEG | POS |
| Dresden 08_1791 | NEG | NEG | NEG | NEG | NEG | NEG | NEG | NEG | POS | NEG | NEG | POS | NEG | POS | POS | NEG | POS | NEG | POS | NEG | NEG | POS | NEG | NEG | POS | NEG | POS | NEG | POS |
| Dresden 08_1643 | NEG | NEG | NEG | NEG | NEG | NEG | NEG | NEG | POS | NEG | NEG | POS | NEG | POS | POS | NEG | POS | NEG | POS | NEG | NEG | POS | NEG | NEG | POS | NEG | POS | NEG | POS |
| Dresden 08_1764 | NEG | POS | NEG | NEG | NEG | NEG | NEG | NEG | POS | NEG | NEG | POS | AMB | POS | POS | NEG | POS | NEG | POS | NEG | NEG | POS | NEG | NEG | POS | NEG | POS | NEG | POS |
| Dresden 08_1748 | NEG | POS | NEG | NEG | NEG | NEG | NEG | NEG | POS | NEG | NEG | POS | AMB | POS | POS | NEG | POS | NEG | POS | NEG | NEG | POS | NEG | NEG | POS | NEG | POS | NEG | POS |
| Dresden 08_1736 | NEG | NEG | NEG | NEG | NEG | NEG | NEG | NEG | POS | NEG | NEG | POS | AMB | POS | POS | NEG | POS | NEG | POS | NEG | NEG | POS | NEG | NEG | POS | NEG | POS | NEG | POS |
| Dresden 08_1582 | NEG | NEG | NEG | NEG | NEG | NEG | NEG | NEG | POS | NEG | NEG | POS | NEG | POS | POS | NEG | POS | NEG | POS | NEG | NEG | POS | NEG | NEG | POS | NEG | POS | NEG | POS |
| **>HP-02** | NEG | NEG | NEG | NEG | NEG | NEG | NEG | NEG | POS | NEG | NEG | POS | NEG | POS | POS | NEG | POS | NEG | POS | NEG | NEG | POS | NEG | NEG | POS | NEG | NEG | NEG | NEG |
| C. difficile ATCC43255 (GenBank ABKJ) in silico prediction | NEG | NEG | NEG | NEG | NEG | NEG | NEG | NEG | POS | NEG | NEG | POS | NEG | POS | POS | NEG | POS | NEG | POS | NEG | NEG | POS | NEG | NEG | POS | NEG | NEG | NEG | NEG |
| Freiburg-885-36.885-DBK0000296 | NEG | NEG | NEG | NEG | NEG | NEG | NEG | NEG | POS | NEG | NEG | POS | POS | POS | POS | NEG | POS | NEG | POS | NEG | NEG | POS | NEG | NEG | POS | NEG | NEG | NEG | NEG |
| Freiburg_37.894-II | NEG | NEG | NEG | NEG | NEG | NEG | NEG | NEG | POS | NEG | NEG | POS | NEG | POS | POS | NEG | POS | NEG | POS | NEG | NEG | POS | NEG | NEG | POS | NEG | NEG | NEG | NEG |
| Freiburg_981-57.981-DBK0000258 | NEG | NEG | NEG | NEG | NEG | NEG | NEG | NEG | POS | NEG | NEG | POS | NEG | AMB | POS | NEG | POS | NEG | POS | NEG | NEG | AMB | NEG | NEG | POS | NEG | NEG | NEG | NEG |
| | NEG | NEG | NEG | NEG | NEG | NEG | NEG | NEG | POS | NEG | NEG | POS | AMB | POS | POS | NEG | POS | NEG | POS | NEG | NEG | POS | NEG | NEG | POS | NEG | NEG | NEG | NEG |
| Jena_S0011_11 | NEG | NEG | NEG | NEG | NEG | NEG | NEG | NEG | POS | NEG | NEG | POS | AMB | NEG | POS | NEG | POS | NEG | POS | NEG | NEG | POS | NEG | NEG | POS | NEG | NEG | NEG | NEG |
| | NEG | NEG | NEG | NEG | NEG | NEG | NEG | NEG | POS | NEG | NEG | POS | NEG | NEG | POS | NEG | POS | NEG | POS | NEG | NEG | POS | NEG | NEG | POS | NEG | NEG | NEG | NEG |
| Dresden 08_1352 | NEG | NEG | NEG | NEG | NEG | NEG | NEG | NEG | POS | NEG | NEG | POS | AMB | POS | POS | NEG | POS | AMB | POS | NEG | NEG | AMB | NEG | NEG | POS | NEG | NEG | NEG | NEG |
| **>HP-03** | NEG | VAR | VAR | NEG | VAR | NEG | NEG | VAR | POS | NEG | NEG | POS | AMB | POS | POS | NEG | POS | NEG | POS | NEG | NEG | POS | NEG | NEG | POS | NEG | NEG | NEG | NEG |
| Freiburg-938-47.938-DBK0000237 | NEG | NEG | NEG | NEG | NEG | NEG | NEG | NEG | POS | NEG | NEG | POS | AMB | POS | POS | NEG | POS | NEG | POS | NEG | NEG | POS | NEG | NEG | POS | NEG | NEG | NEG | NEG |
| | NEG | NEG | NEG | NEG | NEG | NEG | NEG | NEG | POS | NEG | NEG | POS | NEG | POS | POS | NEG | POS | NEG | POS | NEG | NEG | AMB | NEG | NEG | POS | NEG | NEG | NEG | NEG |
| Freiburg-811-25.811-DBK0000284 | NEG | NEG | POS | NEG | POS | NEG | NEG | POS | POS | NEG | NEG | POS | AMB | POS | POS | NEG | POS | NEG | POS | NEG | NEG | POS | NEG | NEG | POS | NEG | NEG | NEG | NEG |
| | NEG | NEG | POS | NEG | POS | NEG | NEG | AMB | POS | NEG | NEG | POS | NEG | POS | POS | NEG | POS | NEG | POS | NEG | NEG | POS | NEG | NEG | POS | NEG | NEG | NEG | NEG |
| Freiburg-987-62.987-DBK0000251 | NEG | NEG | NEG | NEG | NEG | NEG | NEG | NEG | POS | NEG | NEG | POS | AMB | POS | POS | NEG | POS | NEG | POS | NEG | NEG | POS | NEG | NEG | POS | NEG | NEG | NEG | NEG |
| | NEG | NEG | NEG | NEG | NEG | NEG | NEG | NEG | POS | NEG | NEG | POS | AMB | AMB | POS | NEG | POS | NEG | POS | NEG | NEG | AMB | NEG | NEG | POS | NEG | NEG | NEG | NEG |
| Freiburg-972-54.972-DBK0000247 | NEG | POS | NEG | NEG | NEG | NEG | NEG | NEG | POS | NEG | NEG | POS | AMB | POS | POS | NEG | POS | NEG | POS | NEG | NEG | POS | NEG | NEG | POS | NEG | NEG | NEG | NEG |
| | NEG | POS | NEG | NEG | NEG | NEG | NEG | NEG | POS | NEG | NEG | POS | NEG | POS | POS | NEG | POS | NEG | POS | NEG | NEG | POS | NEG | NEG | POS | NEG | NEG | NEG | NEG |
| **>HP-04** | NEG | NEG | NEG | NEG | NEG | NEG | NEG | NEG | POS | NEG | NEG | POS | AMB | POS | POS | NEG | POS | NEG | POS | NEG | AMB | POS | NEG | NEG | POS | NEG | NEG | NEG | NEG |
| Freiburg-984-60.984-DBK0000250 | NEG | NEG | NEG | NEG | NEG | NEG | NEG | NEG | POS | NEG | NEG | POS | AMB | POS | POS | NEG | POS | NEG | POS | NEG | POS | POS | NEG | NEG | POS | NEG | NEG | NEG | NEG |
| Freiburg-1023-72.1023-DBK0000271 | NEG | NEG | NEG | NEG | NEG | NEG | NEG | NEG | POS | NEG | NEG | POS | AMB | POS | POS | POS | POS | AMB | POS | NEG | POS | POS | NEG | NEG | POS | NEG | NEG | NEG | NEG |
| | NEG | NEG | NEG | NEG | NEG | NEG | NEG | NEG | POS | NEG | NEG | POS | NEG | POS | POS | AMB | POS | NEG | POS | NEG | POS | POS | NEG | NEG | POS | NEG | NEG | NEG | NEG |
| Freiburg-1043-74.1043-DBK0000268 | NEG | NEG | NEG | NEG | NEG | NEG | NEG | NEG | POS | NEG | NEG | POS | NEG | POS | POS | NEG | POS | NEG | POS | NEG | NEG | POS | NEG | NEG | POS | NEG | NEG | NEG | NEG |
| Freiburg-633-2.663-DBK0000292 | NEG | NEG | NEG | NEG | NEG | NEG | NEG | NEG | POS | NEG | NEG | POS | POS | POS | POS | AMB | POS | NEG | POS | NEG | NEG | POS | NEG | NEG | POS | NEG | NEG | NEG | NEG |
| | NEG | NEG | NEG | NEG | NEG | NEG | NEG | NEG | POS | NEG | NEG | POS | NEG | POS | POS | NEG | POS | NEG | POS | NEG | NEG | AMB | NEG | NEG | POS | NEG | NEG | NEG | NEG |
| Freiburg-782-20.782-DBK0000276 | NEG | NEG | NEG | NEG | NEG | NEG | NEG | NEG | POS | NEG | NEG | POS | AMB | POS | POS | NEG | POS | NEG | POS | NEG | NEG | POS | NEG | NEG | POS | NEG | NEG | NEG | NEG |
| | NEG | NEG | NEG | NEG | NEG | NEG | NEG | NEG | POS | NEG | NEG | POS | NEG | POS | POS | NEG | POS | NEG | POS | NEG | NEG | POS | NEG | NEG | POS | NEG | NEG | NEG | NEG |
| **>HP-05** | NEG | NEG | NEG | NEG | NEG | NEG | NEG | NEG | POS | NEG | NEG | POS | NEG | POS | POS | NEG | POS | NEG | POS | NEG | NEG | POS | NEG | NEG | POS | NEG | NEG | NEG | NEG |
| Freiburg-740-17.74-DBK0000234 | NEG | NEG | NEG | NEG | NEG | NEG | NEG | NEG | POS | NEG | NEG | POS | AMB | POS | POS | AMB | POS | NEG | POS | NEG | NEG | POS | NEG | NEG | POS | NEG | NEG | NEG | NEG |
| | NEG | NEG | NEG | NEG | NEG | NEG | NEG | NEG | POS | NEG | NEG | POS | NEG | POS | POS | NEG | POS | NEG | POS | NEG | NEG | POS | NEG | NEG | POS | NEG | NEG | NEG | NEG |
| Freiburg-793-23.793-DBK0000286 | NEG | NEG | NEG | NEG | NEG | NEG | NEG | NEG | POS | NEG | NEG | POS | AMB | POS | POS | NEG | POS | NEG | POS | NEG | NEG | POS | NEG | NEG | POS | NEG | NEG | NEG | NEG |
| | NEG | NEG | NEG | NEG | NEG | NEG | NEG | NEG | POS | NEG | NEG | POS | NEG | POS | POS | NEG | POS | NEG | POS | NEG | NEG | POS | NEG | NEG | POS | NEG | NEG | NEG | NEG |
| **>HP-06** | NEG | NEG | NEG | NEG | NEG | NEG | NEG | NEG | POS | NEG | NEG | POS | AMB | POS | POS | NEG | POS | NEG | POS | NEG | NEG | POS | NEG | NEG | POS | NEG | NEG | NEG | NEG |
| Freiburg-874-35.874-DBK0000282 | NEG | NEG | NEG | NEG | NEG | NEG | NEG | NEG | POS | NEG | NEG | POS | POS | POS | POS | AMB | POS | NEG | POS | NEG | NEG | POS | NEG | NEG | POS | NEG | NEG | NEG | NEG |
| | NEG | NEG | NEG | NEG | NEG | NEG | NEG | NEG | POS | NEG | NEG | POS | NEG | POS | POS | NEG | POS | NEG | POS | NEG | NEG | POS | NEG | NEG | POS | NEG | NEG | NEG | NEG |

| STRAIN / ISOLATE | chloramphenicol acetyl transferase (cat) | rRNA methyl-transferase (ermB) | tetM (total) | tetracycline resistance protein, individual probes | | | | | streptogramin A acetyltransferase | | lincomycin resist. protein lmrBNAP07 | lincomycin resist. protein lmrB630 | lantibiotic two-component sensor histidine kinase | | putative lantibiotic ABC transporter, permease protein | | bacitracin resistance protein locus 1 | bacitracin ATP binding cassette transporter, ABC protein BcrA | | | ABC-type transport system, ATP-binding protein | putative efflux pump | MATE efflux family protein | efflux pump antibiotic resistance protein | | ABC-type transport system, permease Tn1549 | | two-component sensor histidine kinase Tn154c | |
|---|---|---|---|---|---|---|---|---|---|---|---|---|---|---|---|---|---|---|---|---|---|---|---|---|---|---|---|---|---|
| | hp1014\|catD | hp1022\|ermB | | hp1105\|tetM | hp1108\|tetM | hp1109\|tetM | hp1110\|tetM | hp1112\|tetM | hp1082\|sat | hp1083\|sat | hp1096\|lmrB | hp1097\|lmrB | hp1079\|spaK | hp1080\|spaK | hp1251\|spaE | hp1252\|spaE | hp1013\|bacA1 | hp1071\|bcrA | hp1072\|bcrA | hp1073\|bcrA | hp1084\|CD0456 | hp1087\|cme | hp1063\|matE | hp1098\|ydiC | hp1244\|ydiC | hp1055\|vexP1 | hp1059\|vexP1 | hp1051\|vncS | hp1052\|vncS |
| **>HP-07** | NEG | NEG | NEG | NEG | NEG | NEG | NEG | NEG | POS | NEG | NEG | POS | AMB | POS | POS | NEG | POS | NEG | POS | NEG | NEG | POS | NEG | NEG | POS | NEG | NEG | NEG | NEG |
| Freiburg-923-44.923-DBK0000238 | NEG | NEG | NEG | NEG | NEG | NEG | NEG | NEG | POS | NEG | NEG | POS | AMB | POS | POS | AMB | POS | NEG | POS | NEG | NEG | POS | NEG | NEG | POS | NEG | NEG | NEG | NEG |
| | NEG | NEG | NEG | NEG | NEG | NEG | NEG | NEG | POS | NEG | NEG | POS | NEG | POS | POS | NEG | POS | NEG | POS | NEG | NEG | POS | NEG | NEG | POS | NEG | NEG | NEG | NEG |
| **>HP-08** | NEG | NEG | NEG | NEG | NEG | NEG | NEG | NEG | POS | NEG | NEG | POS | AMB | POS | POS | NEG | POS | NEG | POS | NEG | POS | POS | NEG | NEG | POS | NEG | NEG | NEG | NEG |
| Freiburg-1021-71.1021-DBK0000248 | NEG | NEG | NEG | NEG | NEG | NEG | NEG | NEG | POS | NEG | NEG | POS | NEG | POS | POS | AMB | POS | NEG | POS | NEG | POS | POS | NEG | NEG | POS | NEG | NEG | NEG | NEG |
| | NEG | NEG | NEG | NEG | NEG | NEG | NEG | NEG | POS | NEG | NEG | POS | NEG | POS | POS | NEG | POS | NEG | POS | NEG | POS | POS | NEG | NEG | POS | NEG | NEG | NEG | NEG |
| Freiburg-669-9.669-DBK0000231 | NEG | NEG | NEG | NEG | NEG | NEG | NEG | NEG | POS | NEG | NEG | POS | NEG | POS | POS | NEG | POS | NEG | POS | NEG | POS | NEG | NEG | NEG | POS | NEG | NEG | NEG | NEG |
| | NEG | NEG | NEG | NEG | NEG | NEG | NEG | NEG | POS | NEG | NEG | POS | POS | POS | POS | AMB | POS | NEG | POS | NEG | POS | POS | NEG | NEG | POS | NEG | NEG | NEG | NEG |
| Freiburg-964-53.964-DBK0000267 | NEG | NEG | NEG | NEG | NEG | NEG | NEG | NEG | POS | NEG | NEG | POS | AMB | POS | POS | AMB | POS | NEG | POS | NEG | POS | POS | NEG | NEG | POS | NEG | NEG | NEG | NEG |
| | NEG | NEG | NEG | NEG | NEG | NEG | NEG | NEG | POS | NEG | NEG | POS | NEG | POS | POS | NEG | POS | NEG | POS | NEG | POS | POS | NEG | NEG | POS | NEG | NEG | NEG | NEG |
| Freiburg-899-41.899-DBK0000224 | NEG | NEG | NEG | NEG | NEG | NEG | NEG | NEG | POS | NEG | NEG | POS | AMB | POS | POS | AMB | POS | NEG | POS | NEG | POS | POS | NEG | NEG | POS | NEG | NEG | NEG | NEG |
| | NEG | NEG | NEG | NEG | NEG | NEG | NEG | NEG | POS | NEG | NEG | POS | NEG | AMB | POS | NEG | POS | NEG | POS | NEG | POS | AMB | NEG | NEG | AMB | NEG | NEG | NEG | NEG |
| Freiburg-982-58.982-DBK0000257 | NEG | NEG | NEG | NEG | NEG | NEG | NEG | NEG | POS | NEG | NEG | POS | NEG | AMB | POS | NEG | POS | NEG | POS | NEG | POS | POS | NEG | NEG | POS | NEG | NEG | NEG | NEG |
| | NEG | NEG | NEG | NEG | NEG | NEG | NEG | NEG | POS | NEG | NEG | POS | NEG | NEG | POS | NEG | POS | NEG | POS | NEG | POS | AMB | NEG | NEG | POS | NEG | NEG | NEG | NEG |
| Dresden 07_7035 | NEG | NEG | NEG | NEG | NEG | NEG | NEG | NEG | POS | NEG | NEG | POS | AMB | POS | POS | NEG | POS | NEG | POS | NEG | POS | POS | NEG | NEG | POS | NEG | NEG | NEG | NEG |
| Dresden 07_7248 | NEG | NEG | NEG | NEG | NEG | NEG | NEG | NEG | POS | NEG | NEG | POS | AMB | POS | POS | POS | POS | AMB | POS | NEG | POS | POS | NEG | NEG | POS | NEG | NEG | NEG | NEG |
| Dresden 07_8702 | NEG | NEG | NEG | NEG | NEG | NEG | NEG | NEG | POS | NEG | NEG | POS | NEG | POS | POS | NEG | POS | NEG | POS | NEG | POS | POS | NEG | NEG | POS | NEG | NEG | NEG | NEG |
| Dresden 07_8989 | NEG | NEG | NEG | NEG | NEG | NEG | NEG | NEG | POS | NEG | NEG | POS | AMB | POS | POS | NEG | POS | NEG | POS | NEG | POS | POS | NEG | NEG | POS | NEG | NEG | NEG | NEG |
| Dresden 08_0712 | NEG | NEG | NEG | NEG | NEG | NEG | NEG | NEG | POS | NEG | NEG | POS | NEG | POS | POS | NEG | POS | NEG | POS | NEG | POS | POS | NEG | NEG | POS | NEG | NEG | NEG | NEG |
| **>HP-09** | NEG | NEG | VAR | NEG | NEG | NEG | VAR | NEG | POS | NEG | NEG | POS | AMB | POS | POS | NEG | POS | NEG | POS | NEG | NEG | POS | NEG | NEG | POS | NEG | NEG | NEG | NEG |
| Freiburg-1056-79.1056-DBK0000261 | NEG | NEG | NEG | NEG | NEG | NEG | NEG | NEG | POS | NEG | NEG | POS | NEG | POS | POS | NEG | POS | NEG | POS | NEG | NEG | POS | NEG | NEG | POS | NEG | NEG | NEG | NEG |
| Freiburg-658-8.658-DBK0000230 | NEG | NEG | NEG | NEG | NEG | NEG | NEG | NEG | POS | NEG | NEG | POS | AMB | POS | POS | AMB | POS | AMB | POS | NEG | NEG | POS | NEG | NEG | POS | NEG | NEG | NEG | NEG |
| | NEG | NEG | NEG | NEG | NEG | NEG | NEG | NEG | POS | NEG | NEG | POS | NEG | POS | POS | NEG | POS | NEG | POS | NEG | NEG | POS | NEG | NEG | POS | NEG | NEG | NEG | NEG |
| Freiburg-963-52.963-DBK0000265 | NEG | NEG | NEG | NEG | NEG | NEG | NEG | NEG | POS | NEG | NEG | POS | AMB | POS | POS | NEG | POS | NEG | POS | NEG | NEG | POS | NEG | NEG | POS | NEG | NEG | NEG | NEG |
| | NEG | NEG | NEG | NEG | NEG | NEG | NEG | NEG | POS | NEG | NEG | POS | NEG | POS | POS | NEG | POS | NEG | POS | NEG | NEG | POS | NEG | NEG | POS | NEG | NEG | NEG | NEG |
| Freiburg-988-63.988-DBK0000253 | NEG | NEG | NEG | NEG | NEG | NEG | NEG | NEG | POS | NEG | NEG | POS | AMB | POS | POS | AMB | POS | NEG | POS | NEG | NEG | POS | NEG | NEG | POS | NEG | NEG | NEG | NEG |
| | NEG | NEG | NEG | NEG | NEG | NEG | NEG | NEG | POS | NEG | NEG | POS | NEG | POS | POS | NEG | POS | NEG | POS | NEG | NEG | POS | NEG | NEG | POS | NEG | NEG | NEG | NEG |
| Freiburg_79.1057 | NEG | NEG | NEG | NEG | NEG | NEG | NEG | NEG | POS | NEG | NEG | POS | NEG | POS | POS | NEG | POS | NEG | POS | NEG | NEG | POS | NEG | NEG | POS | NEG | NEG | NEG | NEG |
| Jena_S0017.11 | NEG | NEG | NEG | NEG | NEG | NEG | NEG | NEG | POS | NEG | NEG | POS | AMB | POS | POS | NEG | POS | NEG | POS | NEG | NEG | AMB | NEG | NEG | POS | NEG | NEG | NEG | NEG |
| | NEG | NEG | NEG | NEG | NEG | NEG | NEG | NEG | POS | NEG | NEG | POS | POS | POS | POS | NEG | POS | NEG | POS | NEG | NEG | POS | NEG | NEG | POS | NEG | NEG | NEG | NEG |
| | NEG | NEG | NEG | NEG | NEG | NEG | NEG | NEG | POS | NEG | NEG | POS | NEG | POS | POS | NEG | POS | NEG | POS | NEG | NEG | POS | NEG | NEG | POS | NEG | NEG | NEG | NEG |
| | NEG | NEG | NEG | NEG | NEG | NEG | NEG | NEG | POS | NEG | NEG | POS | NEG | POS | POS | POS | POS | AMB | POS | NEG | NEG | POS | NEG | NEG | POS | NEG | NEG | NEG | NEG |
| | NEG | NEG | NEG | NEG | NEG | NEG | NEG | NEG | POS | NEG | NEG | POS | NEG | POS | POS | NEG | POS | NEG | POS | NEG | NEG | POS | NEG | NEG | POS | NEG | NEG | NEG | NEG |
| Jena_S0015_11 | NEG | NEG | AMB | NEG | AMB | NEG | NEG | NEG | POS | NEG | NEG | POS | AMB | AMB | POS | NEG | POS | NEG | POS | NEG | NEG | AMB | NEG | NEG | POS | NEG | NEG | NEG | NEG |
| | NEG | NEG | NEG | NEG | NEG | NEG | NEG | NEG | POS | NEG | NEG | POS | AMB | POS | POS | NEG | POS | NEG | POS | NEG | NEG | POS | NEG | NEG | POS | NEG | NEG | NEG | NEG |
| | NEG | NEG | NEG | NEG | NEG | NEG | NEG | NEG | POS | NEG | NEG | POS | NEG | POS | POS | NEG | POS | NEG | POS | NEG | NEG | POS | NEG | NEG | POS | NEG | NEG | NEG | NEG |
| Jena_S0012_11 | NEG | NEG | POS | NEG | NEG | NEG | POS | NEG | POS | NEG | NEG | POS | AMB | POS | POS | NEG | POS | NEG | POS | NEG | NEG | AMB | NEG | NEG | POS | NEG | NEG | NEG | NEG |
| | NEG | NEG | NEG | NEG | NEG | NEG | NEG | NEG | POS | NEG | NEG | POS | NEG | POS | POS | NEG | POS | NEG | POS | NEG | NEG | POS | NEG | NEG | POS | NEG | NEG | NEG | NEG |
| Dresden 07_7363 | NEG | NEG | NEG | NEG | NEG | NEG | NEG | NEG | POS | NEG | NEG | POS | NEG | POS | POS | NEG | POS | NEG | POS | NEG | NEG | POS | NEG | NEG | POS | NEG | NEG | NEG | NEG |
| Dresden 07_8174 | NEG | NEG | NEG | NEG | NEG | NEG | NEG | NEG | POS | NEG | NEG | POS | NEG | POS | POS | NEG | POS | NEG | POS | NEG | NEG | AMB | NEG | NEG | POS | NEG | NEG | NEG | NEG |
| Dresden 07_8224 | NEG | NEG | NEG | NEG | NEG | NEG | NEG | NEG | POS | NEG | NEG | POS | AMB | AMB | POS | NEG | POS | NEG | POS | NEG | NEG | AMB | NEG | NEG | POS | NEG | NEG | NEG | NEG |
| Dresden 07_8562 | NEG | NEG | NEG | NEG | NEG | NEG | NEG | NEG | POS | NEG | NEG | POS | NEG | POS | POS | NEG | POS | NEG | POS | NEG | NEG | POS | NEG | NEG | POS | NEG | NEG | NEG | NEG |
| Dresden 08_613 | NEG | NEG | NEG | NEG | NEG | NEG | NEG | NEG | POS | NEG | NEG | POS | AMB | POS | POS | NEG | POS | NEG | POS | NEG | NEG | AMB | NEG | NEG | POS | NEG | NEG | NEG | NEG |
| Dresden 08_0422 | NEG | NEG | NEG | NEG | NEG | NEG | NEG | NEG | POS | NEG | NEG | POS | AMB | POS | POS | NEG | POS | NEG | POS | NEG | NEG | POS | NEG | NEG | POS | NEG | NEG | NEG | NEG |
| Dresden 08_0837 | NEG | NEG | NEG | NEG | NEG | NEG | NEG | NEG | POS | NEG | NEG | POS | AMB | POS | POS | NEG | POS | NEG | POS | NEG | NEG | POS | NEG | NEG | POS | NEG | NEG | NEG | NEG |
| Dresden 08_0878 | NEG | NEG | NEG | NEG | NEG | NEG | NEG | NEG | POS | NEG | NEG | POS | AMB | AMB | POS | NEG | POS | NEG | POS | NEG | NEG | AMB | NEG | NEG | POS | NEG | NEG | NEG | NEG |
| Dresden 08_1484 | NEG | NEG | NEG | NEG | NEG | NEG | NEG | NEG | POS | NEG | NEG | POS | NEG | POS | POS | AMB | POS | AMB | POS | NEG | NEG | POS | NEG | NEG | POS | NEG | NEG | NEG | NEG |
| **>HP-10** | NEG | NEG | NEG | NEG | NEG | NEG | NEG | NEG | POS | NEG | NEG | POS | NEG | POS | POS | NEG | POS | NEG | POS | NEG | NEG | POS | NEG | NEG | POS | NEG | NEG | NEG | NEG |
| Dresden 09_664_1 | NEG | NEG | NEG | NEG | NEG | NEG | NEG | NEG | POS | NEG | NEG | POS | AMB | POS | POS | NEG | POS | NEG | POS | NEG | NEG | POS | NEG | NEG | POS | NEG | NEG | NEG | NEG |
| Dresden 09_664_2 | NEG | NEG | NEG | NEG | NEG | NEG | NEG | NEG | POS | NEG | NEG | POS | AMB | AMB | POS | NEG | POS | NEG | POS | NEG | NEG | AMB | NEG | NEG | POS | NEG | NEG | NEG | NEG |
| Dresden 09_711_1 | NEG | NEG | NEG | NEG | NEG | NEG | NEG | NEG | POS | NEG | NEG | POS | AMB | AMB | POS | NEG | POS | NEG | POS | NEG | NEG | AMB | NEG | NEG | POS | NEG | NEG | NEG | NEG |
| Dresden 09_711_2 | NEG | NEG | NEG | NEG | NEG | NEG | NEG | NEG | POS | NEG | NEG | POS | NEG | AMB | POS | NEG | POS | NEG | POS | NEG | NEG | AMB | NEG | NEG | POS | NEG | NEG | NEG | NEG |
| Dresden 09_932 | NEG | NEG | NEG | NEG | NEG | NEG | NEG | NEG | POS | NEG | NEG | POS | NEG | POS | POS | NEG | POS | NEG | POS | NEG | NEG | POS | NEG | NEG | POS | NEG | NEG | NEG | NEG |
| Dresden 09_939 | NEG | NEG | NEG | NEG | NEG | NEG | NEG | NEG | POS | NEG | NEG | POS | NEG | POS | POS | NEG | POS | NEG | POS | NEG | NEG | POS | NEG | NEG | POS | NEG | NEG | NEG | NEG |
| Dresden 09_948 | NEG | NEG | NEG | NEG | NEG | NEG | NEG | NEG | POS | NEG | NEG | POS | NEG | POS | POS | NEG | POS | NEG | POS | NEG | NEG | POS | NEG | NEG | POS | NEG | NEG | NEG | NEG |
| **>HP-11** | NEG | NEG | VAR | NEG | VAR | NEG | NEG | VAR | POS | NEG | NEG | POS | AMB | POS | POS | NEG | POS | NEG | POS | NEG | NEG | POS | NEG | NEG | POS | NEG | NEG | NEG | NEG |
| C. difficile CD37 (GenBank AHJJ) in silico prediction | NEG | NEG | NEG | NEG | NEG | NEG | NEG | NEG | POS | NEG | NEG | POS | NEG | POS | POS | NEG | POS | NEG | POS | NEG | NEG | POS | NEG | NEG | POS | NEG | NEG | NEG | NEG |
| Freiburg-684-10.684-DBK0000240 | NEG | NEG | POS | NEG | POS | NEG | NEG | POS | POS | NEG | NEG | POS | POS | POS | POS | AMB | POS | AMB | POS | NEG | NEG | POS | NEG | NEG | POS | NEG | NEG | NEG | NEG |
| Freiburg-838-28.838-DBK0000278 | NEG | NEG | NEG | NEG | NEG | NEG | NEG | NEG | POS | NEG | NEG | POS | POS | POS | POS | AMB | POS | NEG | POS | NEG | NEG | POS | NEG | NEG | POS | NEG | NEG | NEG | NEG |
| | NEG | NEG | NEG | NEG | NEG | NEG | NEG | NEG | POS | NEG | NEG | POS | NEG | POS | POS | NEG | POS | NEG | POS | NEG | NEG | POS | NEG | NEG | POS | NEG | NEG | NEG | NEG |
| **>HP-12** | NEG | NEG | NEG | NEG | NEG | NEG | NEG | NEG | POS | NEG | NEG | POS | AMB | POS | POS | NEG | POS | NEG | POS | NEG | NEG | POS | NEG | NEG | POS | NEG | NEG | NEG | NEG |
| Freiburg-1007-68.1007-DBK0000252 | NEG | NEG | NEG | NEG | NEG | NEG | NEG | NEG | POS | NEG | NEG | POS | NEG | POS | POS | AMB | POS | NEG | POS | NEG | NEG | POS | NEG | NEG | POS | NEG | NEG | NEG | NEG |
| | NEG | NEG | NEG | NEG | NEG | NEG | NEG | NEG | POS | NEG | NEG | POS | NEG | NEG | POS | NEG | POS | NEG | POS | NEG | NEG | NEG | NEG | NEG | POS | NEG | NEG | NEG | NEG |
| Freiburg-983-59.983-DBK0000256 | NEG | NEG | NEG | NEG | NEG | NEG | NEG | NEG | POS | NEG | NEG | POS | AMB | POS | POS | NEG | POS | NEG | POS | NEG | NEG | POS | NEG | NEG | POS | NEG | NEG | NEG | NEG |
| | NEG | NEG | NEG | NEG | NEG | NEG | NEG | NEG | POS | NEG | NEG | POS | AMB | POS | POS | NEG | POS | NEG | POS | NEG | NEG | NEG | NEG | NEG | POS | NEG | NEG | NEG | NEG |
| **>HP-13** | NEG | VAR | NEG | NEG | NEG | NEG | NEG | NEG | POS | NEG | NEG | POS | AMB | POS | POS | NEG | POS | NEG | POS | NEG | NEG | POS | NEG | NEG | POS | NEG | NEG | NEG | NEG |
| C. difficile 70-100-2010 (GenBank AGAC) in silico prediction | NEG | NEG | NEG | NEG | NEG | NEG | NEG | NEG | POS | NEG | NEG | POS | NEG | POS | POS | NEG | POS | NEG | POS | NEG | NEG | POS | NEG | NEG | POS | NEG | NEG | NEG | NEG |
| Freiburg-1044-75.1044-DBK0000270 | NEG | NEG | NEG | NEG | NEG | NEG | NEG | NEG | POS | NEG | NEG | POS | NEG | POS | POS | AMB | POS | NEG | POS | NEG | NEG | POS | NEG | NEG | POS | NEG | NEG | NEG | NEG |
| | NEG | NEG | NEG | NEG | NEG | NEG | NEG | NEG | POS | NEG | NEG | POS | NEG | POS | POS | NEG | POS | NEG | POS | NEG | NEG | POS | NEG | NEG | POS | NEG | NEG | NEG | NEG |
| Freiburg-1057-80.1057-DBK0000260 | NEG | NEG | NEG | NEG | NEG | NEG | NEG | NEG | POS | NEG | NEG | POS | POS | POS | POS | AMB | POS | NEG | POS | NEG | NEG | POS | NEG | NEG | POS | NEG | NEG | NEG | NEG |
| | NEG | NEG | NEG | NEG | NEG | NEG | NEG | NEG | POS | NEG | NEG | POS | NEG | POS | POS | NEG | POS | NEG | POS | NEG | NEG | POS | NEG | NEG | POS | NEG | NEG | NEG | NEG |
| Freiburg-710-12.710-DBK0000241 | NEG | NEG | NEG | NEG | NEG | NEG | NEG | NEG | POS | NEG | NEG | POS | NEG | POS | POS | NEG | POS | NEG | POS | NEG | POS | POS | NEG | NEG | POS | NEG | NEG | NEG | NEG |
| | NEG | NEG | NEG | NEG | NEG | NEG | NEG | NEG | POS | NEG | NEG | POS | NEG | POS | POS | NEG | POS | NEG | POS | NEG | POS | AMB | NEG | NEG | POS | NEG | NEG | NEG | NEG |
| Freiburg-940-48.94-DBK0000244 | NEG | NEG | NEG | NEG | NEG | NEG | NEG | NEG | POS | NEG | NEG | POS | AMB | POS | POS | AMB | POS | NEG | POS | NEG | POS | POS | NEG | NEG | POS | NEG | NEG | NEG | NEG |
| | NEG | NEG | NEG | NEG | NEG | NEG | NEG | NEG | POS | NEG | NEG | POS | NEG | POS | POS | NEG | POS | NEG | POS | NEG | POS | POS | NEG | NEG | POS | NEG | NEG | NEG | NEG |
| Freiburg-980-56.98-DBK0000246 | NEG | NEG | NEG | NEG | NEG | NEG | NEG | NEG | POS | NEG | NEG | POS | AMB | POS | POS | NEG | POS | NEG | POS | NEG | NEG | POS | NEG | NEG | POS | NEG | NEG | NEG | NEG |
| | NEG | NEG | NEG | NEG | NEG | NEG | NEG | NEG | POS | NEG | NEG | POS | NEG | POS | POS | NEG | POS | NEG | POS | NEG | NEG | POS | NEG | NEG | POS | NEG | NEG | NEG | NEG |
| Jena_S0014_11 | NEG | NEG | NEG | NEG | NEG | NEG | NEG | NEG | POS | NEG | NEG | POS | AMB | POS | POS | NEG | POS | NEG | POS | NEG | NEG | POS | NEG | NEG | POS | NEG | NEG | NEG | NEG |
| | NEG | NEG | NEG | NEG | NEG | NEG | NEG | NEG | POS | NEG | NEG | POS | POS | POS | POS | NEG | POS | NEG | POS | NEG | NEG | POS | NEG | NEG | POS | NEG | NEG | NEG | NEG |
| | NEG | NEG | NEG | NEG | NEG | NEG | NEG | NEG | POS | NEG | NEG | POS | NEG | POS | POS | NEG | POS | NEG | POS | NEG | NEG | POS | NEG | NEG | POS | NEG | NEG | NEG | NEG |
| Jena_S0023_11 | NEG | NEG | NEG | NEG | NEG | NEG | NEG | NEG | POS | NEG | NEG | POS | AMB | POS | POS | NEG | POS | NEG | POS | NEG | NEG | AMB | NEG | NEG | POS | NEG | NEG | NEG | NEG |
| | NEG | NEG | NEG | NEG | NEG | NEG | NEG | NEG | POS | NEG | NEG | POS | POS | POS | POS | NEG | POS | NEG | POS | NEG | NEG | POS | NEG | NEG | POS | NEG | NEG | NEG | NEG |
| | NEG | NEG | NEG | NEG | NEG | NEG | NEG | NEG | POS | NEG | NEG | POS | NEG | AMB | POS | NEG | POS | NEG | POS | NEG | NEG | AMB | NEG | NEG | POS | NEG | NEG | NEG | NEG |
| Dresden 07_7779 | NEG | NEG | NEG | NEG | NEG | NEG | NEG | NEG | NEG | NEG | NEG | POS | NEG | NEG | POS | NEG | POS | NEG | POS | NEG | NEG | NEG | NEG | NEG | NEG | NEG | NEG | NEG | NEG |
| Dresden 07_7950 | NEG | NEG | NEG | NEG | NEG | NEG | NEG | NEG | POS | NEG | NEG | POS | POS | POS | POS | NEG | POS | NEG | POS | NEG | NEG | POS | NEG | NEG | POS | NEG | NEG | NEG | NEG |
| Dresden 07_7953 | NEG | POS | NEG | NEG | NEG | NEG | NEG | NEG | POS | NEG | NEG | POS | NEG | POS | POS | NEG | POS | NEG | POS | NEG | NEG | POS | NEG | NEG | POS | NEG | POS | NEG | NEG |
| Dresden 07_8015 | NEG | NEG | NEG | NEG | NEG | NEG | NEG | NEG | POS | NEG | NEG | POS | NEG | POS | POS | NEG | POS | NEG | POS | NEG | NEG | POS | NEG | NEG | POS | NEG | NEG | NEG | NEG |
| Dresden 07_8205 | NEG | NEG | NEG | NEG | NEG | NEG | NEG | NEG | POS | NEG | NEG | POS | AMB | AMB | POS | NEG | POS | NEG | POS | NEG | NEG | POS | NEG | NEG | POS | NEG | NEG | NEG | NEG |
| Dresden 07_8595 | NEG | NEG | NEG | NEG | NEG | NEG | NEG | NEG | POS | NEG | NEG | POS | NEG | POS | POS | NEG | POS | NEG | POS | NEG | NEG | POS | NEG | NEG | POS | NEG | NEG | NEG | NEG |
| Dresden 07_8613 | NEG | NEG | NEG | NEG | NEG | NEG | NEG | NEG | POS | NEG | NEG | POS | AMB | AMB | POS | NEG | POS | NEG | POS | NEG | NEG | AMB | NEG | NEG | POS | NEG | NEG | NEG | NEG |
| Dresden 07_8686 | NEG | NEG | NEG | NEG | NEG | NEG | NEG | NEG | POS | NEG | NEG | POS | NEG | POS | POS | NEG | POS | NEG | POS | NEG | NEG | POS | NEG | NEG | POS | NEG | NEG | NEG | NEG |
| Dresden 07_9180 | NEG | NEG | NEG | NEG | NEG | NEG | NEG | NEG | POS | NEG | NEG | POS | NEG | POS | POS | NEG | POS | NEG | POS | NEG | NEG | POS | NEG | NEG | POS | NEG | NEG | NEG | NEG |
| Dresden 08_0237 | NEG | NEG | NEG | NEG | NEG | NEG | NEG | NEG | POS | NEG | NEG | POS | AMB | AMB | POS | NEG | POS | NEG | POS | NEG | NEG | POS | NEG | NEG | POS | NEG | NEG | NEG | NEG |
| Dresden 08_0492 | NEG | AMB | NEG | NEG | NEG | NEG | NEG | NEG | POS | NEG | NEG | POS | AMB | POS | POS | NEG | POS | NEG | POS | NEG | NEG | POS | NEG | NEG | POS | NEG | POS | NEG | NEG |
| Dresden 08_0992 | NEG | NEG | NEG | NEG | NEG | NEG | NEG | NEG | POS | NEG | NEG | POS | AMB | POS | POS | NEG | POS | NEG | POS | NEG | NEG | POS | NEG | NEG | POS | NEG | NEG | NEG | NEG |
| Dresden 08_1184 | NEG | NEG | NEG | NEG | NEG | NEG | NEG | NEG | POS | NEG | NEG | POS | NEG | POS | POS | NEG | POS | NEG | POS | NEG | NEG | POS | NEG | NEG | POS | NEG | NEG | NEG | NEG |
| Dresden 08_1314 | NEG | NEG | NEG | NEG | NEG | NEG | NEG | NEG | POS | NEG | NEG | POS | AMB | POS | POS | NEG | POS | NEG | POS | NEG | NEG | POS | NEG | NEG | POS | NEG | NEG | NEG | NEG |
| Dresden 08_1640 | NEG | NEG | NEG | NEG | NEG | NEG | NEG | NEG | POS | NEG | NEG | POS | NEG | AMB | POS | NEG | POS | NEG | POS | NEG | NEG | AMB | NEG | NEG | POS | NEG | NEG | NEG | NEG |
| Dresden 08_1640 | NEG | NEG | NEG | NEG | NEG | NEG | NEG | NEG | POS | NEG | NEG | POS | NEG | AMB | POS | NEG | POS | NEG | POS | NEG | NEG | AMB | NEG | NEG | POS | NEG | NEG | NEG | NEG |
| Dresden 08_1758 | NEG | NEG | NEG | NEG | NEG | NEG | NEG | NEG | POS | NEG | NEG | POS | AMB | POS | POS | NEG | POS | NEG | POS | NEG | POS | NEG | NEG | NEG | POS | NEG | NEG | NEG | NEG |
| **>HP-14** | NEG | NEG | NEG | NEG | NEG | NEG | NEG | NEG | POS | NEG | NEG | POS | NEG | POS | POS | AMB | POS | NEG | POS | NEG | NEG | POS | NEG | NEG | POS | NEG | NEG | NEG | NEG |
| Freiburg-1019-70.1019-DBK0000255 | NEG | NEG | NEG | NEG | NEG | NEG | NEG | NEG | POS | NEG | NEG | POS | NEG | POS | POS | AMB | POS | NEG | POS | NEG | NEG | POS | NEG | NEG | POS | NEG | NEG | NEG | NEG |
| **>HP-15** | NEG | NEG | NEG | NEG | NEG | NEG | NEG | NEG | POS | NEG | NEG | POS | NEG | POS | POS | NEG | POS | NEG | POS | NEG | NEG | POS | NEG | NEG | POS | NEG | NEG | NEG | NEG |
| Freiburg_42.907 | NEG | NEG | NEG | NEG | NEG | NEG | NEG | NEG | POS | NEG | NEG | POS | NEG | POS | POS | NEG | POS | NEG | POS | NEG | NEG | POS | NEG | NEG | POS | NEG | NEG | NEG | NEG |
| **>HP-16** | NEG | NEG | NEG | NEG | NEG | NEG | NEG | NEG | POS | NEG | NEG | POS | AMB | POS | POS | NEG | POS | NEG | POS | NEG | NEG | POS | NEG | NEG | POS | NEG | NEG | NEG | NEG |
| Freiburg-978-55.978-DBK0000259 | NEG | NEG | NEG | NEG | NEG | NEG | NEG | NEG | POS | NEG | NEG | POS | AMB | POS | POS | NEG | POS | NEG | POS | NEG | NEG | POS | NEG | NEG | POS | NEG | NEG | NEG | NEG |
| | NEG | NEG | NEG | NEG | NEG | NEG | NEG | NEG | POS | NEG | NEG | POS | NEG | AMB | POS | NEG | POS | NEG | POS | NEG | NEG | AMB | NEG | NEG | POS | NEG | NEG | NEG | NEG |
| Dresden 08_1810 | NEG | NEG | NEG | NEG | NEG | NEG | NEG | NEG | POS | NEG | NEG | POS | AMB | NEG | POS | NEG | POS | NEG | POS | NEG | NEG | AMB | NEG | NEG | POS | NEG | NEG | NEG | NEG |
| **>HP-17** | NEG | NEG | VAR | NEG | VAR | NEG | VAR | NEG | POS | NEG | NEG | POS | AMB | POS | POS | NEG | POS | NEG | POS | NEG | NEG | NEG | NEG | NEG | POS | NEG | NEG | NEG | NEG |
| Freiburg-729-15.729-DBK0000226 | NEG | NEG | POS | AMB | POS | NEG | POS | NEG | POS | NEG | NEG | POS | AMB | AMB | POS | NEG | POS | NEG | POS | NEG | NEG | NEG | NEG | NEG | POS | NEG | NEG | NEG | NEG |
| | NEG | NEG | POS | NEG | POS | NEG | AMB | NEG | POS | NEG | NEG | POS | POS | AMB | POS | NEG | POS | NEG | POS | NEG | NEG | NEG | NEG | NEG | POS | NEG | NEG | NEG | NEG |
| Freiburg-926-45.926-DBK0000236 | NEG | NEG | NEG | NEG | NEG | NEG | NEG | NEG | POS | NEG | NEG | POS | AMB | POS | POS | NEG | POS | NEG | POS | NEG | NEG | AMB | NEG | NEG | POS | NEG | NEG | NEG | NEG |
| | NEG | NEG | NEG | NEG | NEG | NEG | NEG | NEG | POS | NEG | NEG | POS | NEG | POS | POS | NEG | POS | NEG | POS | NEG | NEG | AMB | NEG | NEG | POS | NEG | NEG | NEG | NEG |
| **>HP-18** | NEG | NEG | VAR | NEG | VAR | NEG | NEG | VAR | POS | NEG | NEG | POS | AMB | POS | POS | NEG | POS | NEG | POS | NEG | NEG | POS | NEG | NEG | POS | NEG | NEG | NEG | NEG |
| Freiburg-651-7.651-DBK0000229 | NEG | NEG | POS | NEG | POS | NEG | NEG | POS | POS | NEG | NEG | POS | AMB | POS | POS | AMB | POS | NEG | POS | NEG | NEG | POS | NEG | NEG | POS | NEG | NEG | NEG | NEG |
| | NEG | NEG | POS | NEG | POS | NEG | NEG | POS | POS | NEG | NEG | POS | NEG | POS | POS | NEG | POS | NEG | POS | NEG | NEG | POS | NEG | NEG | POS | NEG | NEG | NEG | NEG |
| Freiburg-725-13.725-DBK0000227 | NEG | NEG | NEG | NEG | NEG | NEG | NEG | NEG | POS | NEG | NEG | POS | AMB | NEG | POS | NEG | POS | NEG | POS | NEG | NEG | POS | NEG | NEG | POS | NEG | NEG | NEG | NEG |
| | NEG | NEG | NEG | NEG | NEG | NEG | NEG | NEG | POS | NEG | NEG | POS | AMB | AMB | POS | NEG | POS | NEG | POS | NEG | NEG | POS | NEG | NEG | POS | NEG | NEG | NEG | NEG |
| Dresden 07_7176 | NEG | NEG | NEG | NEG | NEG | NEG | NEG | NEG | AMB | NEG | NEG | POS | POS | NEG | POS | NEG | POS | NEG | POS | NEG | NEG | NEG | NEG | NEG | AMB | NEG | NEG | NEG | NEG |
| Dresden 07_7209 | NEG | NEG | NEG | NEG | NEG | NEG | NEG | NEG | POS | NEG | NEG | POS | AMB | POS | POS | POS | POS | AMB | POS | NEG | NEG | POS | NEG | NEG | POS | NEG | NEG | NEG | NEG |
| Dresden 07_8029 | NEG | NEG | NEG | NEG | NEG | NEG | NEG | NEG | POS | NEG | NEG | POS | NEG | POS | POS | NEG | POS | NEG | POS | NEG | NEG | POS | NEG | NEG | POS | NEG | NEG | NEG | NEG |
| Dresden 07_8575 | NEG | NEG | NEG | NEG | NEG | NEG | NEG | NEG | POS | NEG | NEG | POS | NEG | POS | POS | NEG | POS | NEG | POS | NEG | NEG | POS | NEG | NEG | POS | NEG | NEG | NEG | NEG |
| Dresden 08_1746 | NEG | NEG | NEG | NEG | NEG | NEG | NEG | NEG | POS | NEG | NEG | POS | AMB | POS | POS | NEG | POS | NEG | POS | NEG | NEG | AMB | NEG | NEG | POS | NEG | NEG | NEG | NEG |
| **>HP-19** | NEG | NEG | NEG | NEG | NEG | NEG | NEG | NEG | POS | NEG | NEG | POS | NEG | POS | POS | NEG | POS | NEG | POS | NEG | NEG | AMB | NEG | NEG | POS | NEG | NEG | NEG | NEG |
| Freiburg-894-37.894-DBK0000289 | NEG | NEG | NEG | NEG | NEG | NEG | NEG | NEG | POS | NEG | NEG | POS | NEG | POS | POS | NEG | POS | NEG | POS | NEG | NEG | POS | NEG | NEG | POS | NEG | NEG | NEG | NEG |
| | NEG | NEG | NEG | NEG | NEG | NEG | NEG | NEG | POS | NEG | NEG | POS | NEG | POS | POS | NEG | POS | NEG | POS | NEG | NEG | NEG | NEG | NEG | POS | NEG | NEG | NEG | NEG |
| Freiburg-1000-67.1-DBK0000263 | NEG | NEG | NEG | NEG | NEG | NEG | NEG | NEG | POS | NEG | NEG | POS | AMB | POS | POS | AMB | POS | AMB | POS | NEG | NEG | POS | NEG | NEG | POS | NEG | NEG | NEG | NEG |
| | NEG | NEG | NEG | NEG | NEG | NEG | NEG | NEG | POS | NEG | NEG | POS | NEG | POS | POS | NEG | POS | NEG | POS | NEG | NEG | AMB | NEG | NEG | POS | NEG | NEG | NEG | NEG |
| Dresden 07_7828 | NEG | NEG | NEG | NEG | NEG | NEG | NEG | NEG | POS | NEG | NEG | POS | NEG | POS | POS | NEG | POS | NEG | POS | NEG | NEG | POS | NEG | NEG | POS | NEG | NEG | NEG | NEG |
| Dresden 08_1128 | NEG | NEG | NEG | NEG | NEG | NEG | NEG | NEG | POS | NEG | NEG | POS | AMB | POS | POS | NEG | POS | NEG | POS | NEG | NEG | NEG | NEG | NEG | POS | NEG | NEG | NEG | NEG |

| STRAIN / ISOLATE | chloramphenicol acetyl transferase (cat) | rRNA methyl-transferase (ermB) | tetM (total) | tetracycline resistance protein, individual probes | | | | | streptogramin A acetyltransferase | | lincomycin resist. protein lmrBNAP07 | lincomycin resist. protein lmrB630 | lantibiotic two-component sensor histidine kinase | | putative lantibiotic ABC transporter, permease protein | | bacitracin resistance protein locus 1 | bacitracin ATP binding cassette transporter, ABC protein BcrA | | | ABC-type transport system, ATP-binding protein | putative efflux pump | MATE efflux family protein | efflux pump antibiotic resistance protein | | ABC-type transport system, permease Tn1549 | | two-component sensor histidine kinase Tn154c | |
|---|---|---|---|---|---|---|---|---|---|---|---|---|---|---|---|---|---|---|---|---|---|---|---|---|---|---|---|---|---|
| | hp1014\|catD | hp1022\|ermB | | hp1105\|tetM | hp1108\|tetM | hp1109\|tetM | hp1110\|tetM | hp1112\|tetM | hp1082\|sat | hp1083\|sat | hp1096\|lmrB | hp1097\|lmrB | hp1079\|spaK | hp1080\|spaK | hp1251\|spaE | hp1252\|spaE | hp1013\|bacA1 | hp1071\|bcrA | hp1072\|bcrA | hp1073\|bcrA | hp1084\|CD0456 | hp1087\|cme | hp1063\|matE | hp1098\|ydiC | hp1244\|ydiC | hp1055\|vexP1 | hp1059\|vexP1 | hp1051\|vncS | hp1052\|vncS |
| **>HP-20** | NEG | NEG | NEG | NEG | NEG | NEG | NEG | NEG | POS | NEG | NEG | POS | AMB | POS | POS | NEG | POS | NEG | POS | NEG | NEG | AMB | NEG | NEG | POS | NEG | NEG | NEG | NEG |
| Freiburg-963-50.963-DBK0000243 | NEG | NEG | NEG | NEG | NEG | NEG | NEG | NEG | POS | NEG | NEG | POS | AMB | POS | POS | NEG | POS | NEG | POS | NEG | NEG | POS | NEG | NEG | POS | NEG | NEG | NEG | NEG |
| Freiburg-750-18.75-DBK0000232 | NEG | NEG | NEG | NEG | NEG | NEG | NEG | NEG | POS | NEG | NEG | POS | AMB | POS | POS | AMB | POS | NEG | POS | NEG | NEG | POS | NEG | NEG | POS | NEG | NEG | NEG | NEG |
| | NEG | NEG | NEG | NEG | NEG | NEG | NEG | NEG | POS | NEG | NEG | POS | NEG | NEG | POS | NEG | POS | NEG | POS | NEG | NEG | NEG | NEG | NEG | POS | NEG | NEG | NEG | NEG |
| Freiburg-637-4.637-DBK0000280 | NEG | NEG | NEG | NEG | NEG | NEG | NEG | NEG | POS | NEG | NEG | POS | POS | POS | POS | AMB | POS | AMB | POS | NEG | NEG | NEG | NEG | NEG | POS | NEG | NEG | NEG | NEG |
| | NEG | NEG | NEG | NEG | NEG | NEG | NEG | NEG | POS | NEG | NEG | POS | NEG | POS | POS | NEG | POS | NEG | POS | NEG | NEG | NEG | NEG | NEG | POS | NEG | NEG | NEG | NEG |
| Freiburg-856-31.856-DBK0000273 | NEG | NEG | NEG | NEG | NEG | NEG | NEG | NEG | POS | NEG | NEG | POS | NEG | POS | POS | NEG | POS | NEG | POS | NEG | NEG | POS | NEG | NEG | POS | NEG | NEG | NEG | NEG |
| | NEG | NEG | NEG | NEG | NEG | NEG | NEG | NEG | POS | NEG | NEG | POS | NEG | POS | POS | NEG | POS | NEG | POS | NEG | NEG | POS | NEG | NEG | POS | NEG | NEG | NEG | NEG |
| Freiburg_51.956 | NEG | NEG | NEG | NEG | NEG | NEG | NEG | NEG | POS | NEG | NEG | POS | NEG | POS | POS | NEG | POS | NEG | POS | NEG | NEG | POS | NEG | NEG | POS | NEG | NEG | NEG | NEG |
| Dresden 07_7984 | NEG | NEG | NEG | NEG | NEG | NEG | NEG | NEG | POS | NEG | NEG | POS | NEG | POS | POS | NEG | POS | NEG | POS | NEG | NEG | NEG | NEG | NEG | POS | NEG | NEG | NEG | NEG |
| Dresden 07_8203 | NEG | NEG | NEG | NEG | NEG | NEG | NEG | NEG | POS | NEG | NEG | POS | AMB | AMB | POS | NEG | POS | NEG | POS | NEG | NEG | AMB | NEG | NEG | POS | NEG | NEG | NEG | NEG |
| Dresden 07_8398 | NEG | NEG | NEG | NEG | NEG | NEG | NEG | NEG | POS | NEG | NEG | POS | NEG | POS | POS | NEG | POS | NEG | POS | NEG | NEG | NEG | NEG | NEG | POS | NEG | NEG | NEG | NEG |
| Dresden 07_8798 | NEG | NEG | NEG | NEG | NEG | NEG | NEG | NEG | POS | NEG | NEG | POS | AMB | POS | POS | NEG | POS | NEG | POS | NEG | NEG | NEG | NEG | NEG | POS | NEG | NEG | NEG | NEG |
| **>HP-21** | NEG | NEG | NEG | NEG | NEG | NEG | NEG | NEG | POS | NEG | NEG | POS | AMB | POS | POS | NEG | POS | NEG | POS | NEG | NEG | POS | NEG | NEG | POS | NEG | NEG | NEG | NEG |
| Freiburg-823-26.823-DBK0000283 | NEG | NEG | NEG | NEG | NEG | NEG | NEG | NEG | POS | NEG | NEG | POS | POS | POS | POS | AMB | POS | AMB | POS | NEG | NEG | POS | NEG | NEG | POS | NEG | NEG | NEG | NEG |
| | NEG | NEG | NEG | NEG | NEG | NEG | NEG | NEG | POS | NEG | NEG | POS | NEG | POS | POS | NEG | POS | NEG | POS | NEG | NEG | AMB | NEG | NEG | POS | NEG | NEG | NEG | NEG |
| Freiburg-952-49.952-DBK0000245 | NEG | NEG | NEG | NEG | NEG | NEG | NEG | NEG | POS | NEG | NEG | POS | NEG | POS | POS | NEG | POS | NEG | POS | NEG | NEG | POS | NEG | NEG | POS | NEG | NEG | NEG | NEG |
| | NEG | NEG | NEG | NEG | NEG | NEG | NEG | NEG | POS | NEG | NEG | POS | AMB | POS | POS | NEG | POS | NEG | POS | NEG | NEG | POS | NEG | NEG | POS | NEG | NEG | NEG | NEG |
| **>HP-22** | NEG | POS | POS | NEG | POS | NEG | POS | NEG | POS | NEG | NEG | POS | AMB | POS | POS | NEG | POS | NEG | POS | NEG | POS | POS | NEG | NEG | POS | NEG | POS | NEG | POS |
| Dresden 08_0288 | NEG | POS | POS | NEG | POS | NEG | POS | NEG | POS | NEG | NEG | POS | AMB | POS | POS | NEG | POS | NEG | POS | NEG | POS | POS | NEG | NEG | POS | NEG | POS | NEG | POS |
| **>HP-23** | NEG | POS | POS | NEG | POS | NEG | NEG | POS | POS | NEG | NEG | POS | NEG | POS | POS | NEG | POS | NEG | POS | NEG | POS | POS | NEG | NEG | POS | NEG | POS | NEG | POS |
| Freiburg-786-21.786-DBK0000275 | NEG | POS | POS | NEG | POS | NEG | NEG | POS | POS | NEG | NEG | POS | AMB | POS | POS | NEG | POS | NEG | POS | NEG | POS | POS | NEG | NEG | POS | NEG | POS | NEG | POS |
| | NEG | POS | POS | NEG | POS | NEG | NEG | POS | POS | NEG | NEG | POS | NEG | POS | POS | NEG | POS | NEG | POS | NEG | POS | POS | NEG | NEG | POS | NEG | POS | NEG | POS |
| **>HP-24** | VAR | VAR | VAR | VAR | VAR | NEG | NEG | VAR | POS | NEG | NEG | POS | AMB | POS | POS | NEG | POS | NEG | POS | NEG | POS | POS | POS | NEG | POS | NEG | POS | NEG | POS |
| C. difficile 6534 (GenBank ADEJ) in silico prediction | NEG | POS | POS | NEG | POS | NEG | NEG | POS | POS | NEG | NEG | POS | NEG | POS | POS | NEG | POS | NEG | POS | NEG | POS | POS | POS | NEG | POS | POS | POS | POS | POS |
| C. difficile 630 (GenBank AM180355) in silico prediction | NEG | POS | POS | NEG | POS | NEG | NEG | POS | POS | NEG | NEG | POS | NEG | POS | POS | NEG | POS | NEG | POS | NEG | POS | POS | POS | NEG | POS | NEG | POS | NEG | POS |
| C. difficile Strain 630 | NEG | POS | POS | NEG | POS | NEG | NEG | POS | POS | NEG | NEG | POS | NEG | POS | POS | AMB | POS | NEG | POS | NEG | POS | POS | POS | NEG | POS | NEG | POS | NEG | POS |
| | NEG | POS | POS | NEG | POS | NEG | NEG | POS | POS | NEG | NEG | POS | NEG | POS | POS | POS | POS | AMB | POS | NEG | POS | POS | POS | NEG | POS | NEG | POS | NEG | POS |
| | NEG | POS | POS | NEG | POS | NEG | NEG | POS | POS | NEG | NEG | POS | NEG | POS | POS | NEG | POS | NEG | POS | NEG | POS | POS | POS | NEG | POS | NEG | POS | NEG | POS |
| | NEG | POS | POS | NEG | POS | NEG | NEG | POS | POS | NEG | NEG | POS | AMB | POS | POS | NEG | POS | NEG | POS | NEG | POS | AMB | AMB | NEG | POS | NEG | POS | NEG | POS |
| | NEG | POS | POS | NEG | POS | NEG | NEG | POS | POS | NEG | NEG | POS | AMB | POS | POS | NEG | POS | NEG | POS | NEG | POS | POS | POS | NEG | POS | NEG | POS | NEG | POS |
| | NEG | POS | POS | NEG | POS | NEG | NEG | POS | POS | NEG | NEG | POS | NEG | AMB | POS | NEG | POS | NEG | POS | NEG | POS | AMB | POS | NEG | POS | NEG | POS | NEG | AMB |
| Dresden 07_7156 | POS | POS | POS | NEG | POS | NEG | NEG | AMB | POS | NEG | NEG | POS | AMB | NEG | POS | NEG | POS | NEG | POS | NEG | POS | AMB | AMB | NEG | AMB | NEG | POS | NEG | NEG |
| Dresden 07_7158 | POS | POS | POS | NEG | POS | NEG | NEG | POS | POS | NEG | NEG | POS | AMB | POS | POS | NEG | POS | NEG | POS | NEG | POS | POS | POS | NEG | POS | NEG | POS | NEG | POS |
| Dresden 07_7203 | POS | POS | POS | NEG | POS | NEG | NEG | POS | POS | NEG | NEG | POS | AMB | POS | POS | NEG | POS | NEG | POS | NEG | POS | POS | POS | NEG | POS | NEG | POS | NEG | POS |
| Dresden 07_8217 | NEG | POS | POS | POS | NEG | NEG | NEG | NEG | POS | NEG | NEG | POS | AMB | POS | POS | NEG | POS | NEG | POS | NEG | POS | POS | POS | NEG | POS | NEG | POS | NEG | POS |
| Dresden 07_8270 | POS | POS | POS | NEG | POS | NEG | NEG | POS | POS | NEG | NEG | POS | AMB | POS | POS | NEG | POS | NEG | POS | NEG | POS | POS | POS | NEG | POS | NEG | POS | NEG | POS |
| Dresden 07_8749 | POS | POS | POS | NEG | POS | NEG | NEG | NEG | POS | NEG | NEG | POS | AMB | NEG | POS | NEG | POS | NEG | POS | NEG | AMB | NEG | AMB | NEG | POS | NEG | POS | NEG | NEG |
| Dresden 07_8788 | POS | POS | POS | NEG | POS | NEG | NEG | POS | POS | NEG | NEG | POS | NEG | POS | POS | NEG | POS | NEG | POS | NEG | POS | POS | POS | NEG | POS | NEG | POS | NEG | POS |
| Dresden 07_9245 | NEG | POS | POS | POS | POS | NEG | NEG | POS | POS | NEG | NEG | POS | NEG | POS | POS | NEG | POS | NEG | POS | NEG | POS | POS | POS | NEG | POS | NEG | POS | NEG | POS |
| Dresden 08_0217 | POS | POS | POS | NEG | POS | NEG | NEG | POS | POS | NEG | NEG | POS | NEG | POS | POS | NEG | POS | NEG | POS | NEG | POS | POS | POS | NEG | POS | NEG | POS | NEG | POS |
| Dresden 08_0219 | NEG | POS | POS | POS | POS | NEG | NEG | POS | POS | NEG | NEG | POS | AMB | POS | POS | NEG | POS | NEG | POS | NEG | POS | POS | POS | NEG | POS | NEG | POS | NEG | POS |
| Dresden 08_0419 | NEG | POS | POS | POS | POS | NEG | NEG | POS | POS | NEG | NEG | POS | AMB | POS | POS | NEG | POS | NEG | POS | NEG | POS | POS | POS | NEG | POS | NEG | POS | NEG | POS |
| Dresden 08_0484 | POS | POS | POS | NEG | POS | NEG | NEG | POS | POS | NEG | NEG | POS | AMB | POS | POS | NEG | POS | NEG | POS | NEG | POS | POS | POS | NEG | POS | NEG | POS | NEG | POS |
| Dresden 08_0994 | POS | POS | POS | NEG | POS | NEG | NEG | POS | POS | NEG | NEG | POS | AMB | POS | POS | NEG | POS | NEG | POS | NEG | POS | POS | POS | NEG | POS | NEG | POS | NEG | POS |
| Dresden 08_1148 | NEG | POS | POS | POS | NEG | NEG | NEG | NEG | POS | NEG | NEG | POS | AMB | POS | POS | NEG | POS | NEG | POS | NEG | POS | POS | POS | NEG | POS | NEG | POS | NEG | POS |
| Dresden 08_1188 | POS | POS | POS | NEG | POS | NEG | NEG | POS | POS | NEG | NEG | POS | AMB | POS | POS | NEG | POS | NEG | POS | NEG | POS | POS | POS | NEG | POS | NEG | POS | NEG | POS |
| Dresden 08_1205 | POS | POS | POS | NEG | POS | NEG | NEG | POS | POS | NEG | NEG | POS | AMB | POS | POS | NEG | POS | NEG | POS | NEG | POS | POS | POS | NEG | POS | NEG | POS | NEG | POS |
| Dresden 08_1541 | AMB | POS | NEG | NEG | NEG | NEG | NEG | NEG | POS | NEG | NEG | POS | AMB | POS | POS | AMB | POS | NEG | POS | NEG | POS | AMB | POS | NEG | POS | NEG | POS | NEG | POS |
| Dresden 06_7896 | NEG | NEG | NEG | NEG | NEG | NEG | NEG | NEG | POS | NEG | NEG | POS | AMB | NEG | POS | NEG | POS | NEG | POS | NEG | NEG | AMB | NEG | NEG | AMB | NEG | NEG | NEG | NEG |
| **>HP-25** | POS | POS | POS | NEG | POS | NEG | NEG | POS | POS | NEG | NEG | POS | NEG | POS | POS | NEG | POS | NEG | POS | NEG | NEG | POS | NEG | NEG | POS | NEG | POS | NEG | POS |
| Freiburg-809-24.809-DBK0000287 | POS | POS | POS | NEG | POS | NEG | NEG | POS | POS | NEG | NEG | POS | AMB | POS | POS | NEG | POS | NEG | POS | NEG | NEG | POS | NEG | NEG | POS | NEG | POS | NEG | POS |
| | POS | POS | POS | NEG | POS | NEG | NEG | POS | POS | NEG | NEG | POS | NEG | POS | POS | NEG | POS | NEG | POS | NEG | NEG | POS | NEG | NEG | POS | NEG | POS | NEG | POS |
| Freiburg-990-64.99-DBK0000264 | POS | POS | POS | NEG | POS | NEG | NEG | POS | POS | NEG | NEG | POS | AMB | POS | POS | NEG | POS | NEG | POS | NEG | NEG | POS | NEG | NEG | POS | NEG | POS | NEG | POS |
| | POS | POS | POS | NEG | POS | NEG | NEG | POS | POS | NEG | NEG | POS | NEG | POS | POS | NEG | POS | NEG | POS | NEG | NEG | POS | NEG | NEG | POS | NEG | POS | NEG | POS |
| Dresden 07_7952 | POS | POS | POS | NEG | POS | NEG | NEG | POS | POS | NEG | NEG | POS | NEG | POS | POS | NEG | POS | NEG | POS | NEG | NEG | POS | NEG | NEG | POS | NEG | POS | NEG | POS |
| Dresden 07_8955 | POS | POS | POS | NEG | POS | NEG | NEG | POS | POS | NEG | NEG | POS | AMB | POS | POS | NEG | POS | NEG | POS | NEG | NEG | POS | NEG | NEG | POS | NEG | POS | NEG | POS |
| Dresden 07_8958 | POS | POS | POS | NEG | POS | NEG | NEG | POS | POS | NEG | NEG | POS | AMB | POS | POS | NEG | POS | NEG | POS | NEG | NEG | POS | NEG | NEG | POS | NEG | POS | NEG | POS |
| Dresden 07_9027 | POS | POS | POS | NEG | POS | NEG | NEG | POS | POS | NEG | NEG | POS | AMB | POS | POS | AMB | POS | NEG | POS | NEG | NEG | POS | NEG | NEG | POS | NEG | POS | NEG | POS |
| Dresden 08_1169 | POS | POS | POS | NEG | POS | NEG | NEG | POS | POS | NEG | NEG | POS | AMB | AMB | POS | NEG | POS | NEG | POS | NEG | NEG | AMB | NEG | NEG | POS | NEG | POS | NEG | POS |
| **>HP-26** | NEG | POS | POS | NEG | POS | NEG | NEG | POS | POS | NEG | NEG | POS | AMB | POS | POS | NEG | POS | NEG | POS | NEG | NEG | POS | NEG | NEG | POS | NEG | POS | NEG | POS |
| Freiburg-635-3.635-DBK0000297 | NEG | POS | POS | NEG | POS | NEG | NEG | POS | POS | NEG | NEG | POS | AMB | POS | POS | POS | POS | AMB | POS | NEG | NEG | POS | NEG | NEG | POS | NEG | POS | NEG | POS |
| | NEG | POS | POS | NEG | POS | NEG | NEG | POS | POS | NEG | NEG | POS | NEG | POS | POS | NEG | POS | NEG | POS | NEG | NEG | POS | NEG | NEG | POS | NEG | POS | NEG | POS |
| Freiburg-895-38.895-DBK0000290 | NEG | POS | POS | NEG | POS | NEG | NEG | POS | POS | NEG | NEG | POS | AMB | POS | POS | NEG | POS | NEG | POS | NEG | NEG | POS | NEG | NEG | POS | NEG | POS | NEG | POS |
| | NEG | POS | POS | NEG | POS | NEG | NEG | POS | POS | NEG | NEG | POS | NEG | POS | POS | NEG | POS | NEG | POS | NEG | NEG | POS | NEG | NEG | POS | NEG | POS | NEG | POS |
| **>HP-27** | NEG | POS | NEG | NEG | NEG | NEG | NEG | NEG | POS | NEG | NEG | POS | NEG | POS | POS | AMB | POS | NEG | POS | NEG | POS | POS | NEG | NEG | POS | NEG | NEG | NEG | NEG |
| Freiburg-726-14.726-DBK0000228 | NEG | POS | NEG | NEG | NEG | NEG | NEG | NEG | POS | NEG | NEG | POS | AMB | POS | POS | AMB | POS | AMB | POS | NEG | POS | POS | NEG | NEG | POS | NEG | NEG | NEG | NEG |
| | NEG | POS | NEG | NEG | NEG | NEG | NEG | NEG | POS | NEG | NEG | POS | NEG | POS | POS | AMB | POS | NEG | POS | NEG | POS | POS | NEG | NEG | POS | NEG | NEG | NEG | NEG |
| Freiburg-837-27.837-DBK0000285 | NEG | POS | NEG | NEG | NEG | NEG | NEG | NEG | POS | NEG | NEG | POS | NEG | POS | POS | AMB | POS | NEG | POS | NEG | POS | POS | NEG | NEG | POS | NEG | NEG | NEG | NEG |
| | NEG | POS | NEG | NEG | NEG | NEG | NEG | NEG | POS | NEG | NEG | POS | NEG | POS | POS | NEG | POS | NEG | POS | NEG | POS | POS | NEG | NEG | POS | NEG | NEG | NEG | NEG |
| **>HP-28** | NEG | NEG | NEG | NEG | NEG | NEG | NEG | NEG | POS | NEG | NEG | POS | NEG | NEG | POS | NEG | POS | NEG | POS | NEG | NEG | POS | NEG | NEG | POS | NEG | NEG | NEG | NEG |
| C. difficile 6407 (GenBank ADEH) in silico prediction | NEG | NEG | NEG | NEG | NEG | NEG | NEG | NEG | POS | NEG | NEG | POS | NEG | NEG | POS | NEG | POS | NEG | POS | NEG | NEG | POS | NEG | NEG | POS | NEG | NEG | NEG | NEG |
| **>HP-29** | NEG | NEG | NEG | NEG | NEG | NEG | NEG | NEG | POS | NEG | NEG | POS | AMB | POS | POS | AMB | POS | NEG | POS | NEG | NEG | POS | NEG | NEG | POS | NEG | NEG | NEG | NEG |
| Freiburg-852-29.852-DBK0000279 | NEG | NEG | NEG | NEG | NEG | NEG | NEG | NEG | POS | NEG | NEG | POS | NEG | POS | POS | NEG | POS | NEG | POS | NEG | NEG | POS | NEG | NEG | POS | NEG | NEG | NEG | NEG |
| | NEG | NEG | NEG | NEG | NEG | NEG | NEG | NEG | POS | NEG | NEG | POS | NEG | POS | POS | NEG | POS | NEG | POS | NEG | NEG | POS | NEG | NEG | POS | NEG | NEG | NEG | NEG |
| Freiburg-858-32.858-DBK0000222 | NEG | NEG | NEG | NEG | NEG | NEG | NEG | NEG | POS | NEG | NEG | POS | POS | POS | POS | AMB | POS | NEG | POS | NEG | NEG | POS | NEG | NEG | POS | NEG | NEG | NEG | NEG |
| **>HP-30** | NEG | VAR | NEG | NEG | NEG | NEG | NEG | NEG | POS | NEG | NEG | POS | NEG | POS | POS | NEG | POS | NEG | POS | NEG | NEG | POS | NEG | NEG | POS | NEG | NEG | NEG | NEG |
| Freiburg-1047-76.1047-DBK0000269 | NEG | NEG | NEG | NEG | NEG | NEG | NEG | NEG | POS | NEG | NEG | POS | NEG | POS | POS | NEG | POS | NEG | POS | NEG | NEG | POS | NEG | NEG | POS | NEG | NEG | NEG | NEG |
| | NEG | NEG | NEG | NEG | NEG | NEG | NEG | NEG | POS | NEG | NEG | POS | NEG | POS | POS | NEG | POS | NEG | POS | NEG | NEG | POS | NEG | NEG | POS | NEG | NEG | NEG | NEG |
| Freiburg-999-66.999-DBK0000262 | NEG | POS | NEG | NEG | NEG | NEG | NEG | NEG | POS | NEG | NEG | POS | NEG | POS | POS | AMB | POS | NEG | POS | NEG | NEG | POS | NEG | NEG | POS | NEG | NEG | NEG | NEG |
| | NEG | POS | NEG | NEG | NEG | NEG | NEG | NEG | POS | NEG | NEG | POS | NEG | POS | POS | NEG | POS | NEG | POS | NEG | NEG | POS | NEG | NEG | POS | NEG | NEG | NEG | NEG |
| Freiburg-896-39.896-DBK0000291 | NEG | NEG | NEG | NEG | NEG | NEG | NEG | NEG | POS | NEG | NEG | POS | AMB | POS | POS | NEG | POS | NEG | POS | NEG | NEG | POS | NEG | NEG | POS | NEG | NEG | NEG | NEG |
| | NEG | NEG | NEG | NEG | NEG | NEG | NEG | NEG | POS | NEG | NEG | POS | NEG | POS | POS | NEG | POS | NEG | POS | NEG | NEG | POS | NEG | NEG | POS | NEG | NEG | NEG | NEG |
| Freiburg-871-33.871-DBK0000272 | NEG | NEG | NEG | NEG | NEG | NEG | NEG | NEG | POS | NEG | NEG | POS | AMB | POS | POS | NEG | POS | NEG | POS | NEG | NEG | POS | NEG | NEG | POS | NEG | NEG | NEG | NEG |
| | NEG | NEG | NEG | NEG | NEG | NEG | NEG | NEG | POS | NEG | NEG | POS | NEG | POS | POS | NEG | POS | NEG | POS | NEG | NEG | POS | NEG | NEG | POS | NEG | NEG | NEG | NEG |
| Dresden 08_1596 | NEG | NEG | NEG | NEG | NEG | NEG | NEG | NEG | POS | NEG | NEG | POS | AMB | POS | POS | NEG | POS | NEG | POS | NEG | NEG | POS | NEG | NEG | POS | NEG | NEG | NEG | NEG |

| STRAIN / ISOLATE | chloramphenicol acetyl transferase (cat) | rRNA methyl-transferase (ermB) | tetM (total) | tetracycline resistance protein, individual probes | | | | | streptogramin A acetyltransferase | | lincomycin resist. protein lmrBNAP07 | lincomycin resist. protein lmrB630 | lantibiotic two-component sensor histidine kinase | | putative lantibiotic ABC transporter, permease protein | | bacitracin resistance protein locus 1 | bacitracin ATP binding cassette transporter, ABC protein BcrA | | | ABC-type transport system, ATP-binding protein | putative efflux pump | MATE efflux family protein | efflux pump antibiotic resistance protein | | ABC-type transport system, permease Tn1549 | | two-component sensor histidine kinase Tn154c | |
|---|---|---|---|---|---|---|---|---|---|---|---|---|---|---|---|---|---|---|---|---|---|---|---|---|---|---|---|---|---|
| | hp1014\|catD | hp1022\|ermB | | hp1105\|tetM | hp1108\|tetM | hp1109\|tetM | hp1110\|tetM | hp1112\|tetM | hp1082\|sat | hp1083\|sat | hp1096\|lmrB | hp1097\|lmrB | hp1079\|spaK | hp1080\|spaK | hp1251\|spaE | hp1252\|spaE | hp1013\|bacA1 | hp1071\|bcrA | hp1072\|bcrA | hp1073\|bcrA | hp1084\|CD0456 | hp1087\|cme | hp1063\|matE | hp1098\|ydiC | hp1244\|ydiC | hp1055\|vexP1 | hp1059\|vexP1 | hp1051\|vncS | hp1052\|vncS |
| **CLADE 2 (CC1 /Ribo 27 Complex)** | | | | | | | | | | | | | | | | | | | | | | | | | | | | | |
| **>HP-31** | NEG | NEG | NEG | NEG | NEG | NEG | NEG | NEG | POS | NEG | NEG | POS | NEG | POS | POS | NEG | POS | NEG | POS | NEG | POS | POS | NEG | NEG | POS | NEG | NEG | NEG | NEG |
| C. difficile BI1 (GenBank FN668941) in silico prediction | NEG | NEG | NEG | NEG | NEG | NEG | NEG | NEG | POS | NEG | NEG | POS | NEG | POS | POS | NEG | POS | NEG | POS | NEG | POS | POS | NEG | NEG | POS | NEG | NEG | NEG | NEG |
| C. difficile QCD-97b34 (GenBank ABHF) in silico prediction | NEG | NEG | NEG | NEG | NEG | NEG | NEG | NEG | POS | NEG | NEG | POS | NEG | POS | POS | NEG | POS | NEG | POS | NEG | POS | POS | NEG | NEG | POS | NEG | NEG | NEG | NEG |
| C. difficile QCD-76w55 (GenBank ABHE) in silico prediction | NEG | NEG | NEG | NEG | NEG | NEG | NEG | NEG | POS | NEG | NEG | POS | NEG | POS | POS | NEG | POS | NEG | POS | NEG | POS | POS | NEG | NEG | POS | NEG | NEG | NEG | NEG |
| C. difficile CIP107932 (GenBank ABKK) in silico prediction | NEG | NEG | NEG | NEG | NEG | NEG | NEG | NEG | POS | NEG | NEG | POS | NEG | POS | POS | NEG | POS | NEG | POS | NEG | POS | POS | NEG | NEG | POS | NEG | NEG | NEG | NEG |
| C. difficile CD196 (GenBank FN538970) in silico prediction | NEG | NEG | NEG | NEG | NEG | NEG | NEG | NEG | POS | NEG | NEG | POS | NEG | POS | POS | NEG | POS | NEG | POS | NEG | POS | POS | NEG | NEG | POS | NEG | NEG | NEG | NEG |
| C. difficile Strain CD196 | NEG | NEG | NEG | NEG | NEG | NEG | NEG | NEG | POS | NEG | NEG | POS | NEG | POS | POS | NEG | POS | NEG | POS | NEG | POS | POS | NEG | NEG | POS | NEG | NEG | NEG | NEG |
| | NEG | NEG | NEG | NEG | NEG | NEG | NEG | NEG | POS | NEG | NEG | POS | POS | POS | POS | NEG | POS | NEG | POS | NEG | POS | POS | NEG | NEG | POS | NEG | NEG | NEG | NEG |
| | NEG | NEG | NEG | NEG | NEG | NEG | NEG | NEG | POS | NEG | NEG | POS | NEG | POS | POS | POS | POS | AMB | POS | NEG | POS | POS | NEG | NEG | POS | NEG | NEG | NEG | NEG |
| | NEG | NEG | NEG | NEG | NEG | NEG | NEG | NEG | POS | NEG | NEG | POS | NEG | POS | POS | NEG | POS | NEG | POS | NEG | POS | POS | NEG | NEG | POS | NEG | NEG | NEG | NEG |
| | NEG | NEG | NEG | NEG | NEG | NEG | NEG | NEG | POS | NEG | NEG | POS | NEG | POS | POS | NEG | POS | NEG | POS | NEG | POS | POS | NEG | NEG | POS | NEG | NEG | NEG | NEG |
| Dresden 07-7829 | NEG | NEG | NEG | NEG | NEG | NEG | NEG | NEG | POS | NEG | NEG | POS | NEG | POS | POS | NEG | POS | NEG | POS | NEG | POS | POS | NEG | NEG | POS | NEG | NEG | NEG | NEG |
| Dresden 07-8176 | NEG | NEG | NEG | NEG | NEG | NEG | NEG | NEG | POS | NEG | NEG | POS | POS | POS | POS | AMB | POS | NEG | POS | NEG | POS | POS | NEG | NEG | POS | NEG | NEG | NEG | NEG |
| **>HP-32** | NEG | VAR | NEG | NEG | NEG | NEG | NEG | NEG | POS | NEG | NEG | POS | NEG | POS | POS | NEG | POS | NEG | POS | NEG | POS | POS | NEG | NEG | POS | NEG | POS | NEG | POS |
| C. difficile 2007855 (GenBank FN665654) in silico prediction | NEG | POS | NEG | NEG | NEG | NEG | NEG | NEG | POS | NEG | NEG | POS | NEG | POS | POS | NEG | POS | NEG | POS | NEG | POS | POS | NEG | NEG | POS | NEG | POS | NEG | POS |
| C. difficile QCD-32g58 (GenBank AAML) in silico prediction | NEG | NEG | NEG | NEG | NEG | NEG | NEG | NEG | POS | NEG | NEG | POS | NEG | POS | POS | NEG | POS | NEG | POS | NEG | POS | POS | NEG | NEG | POS | NEG | POS | NEG | POS |
| C. difficile QCD-37x79 (GenBank ABHG) in silico prediction | NEG | NEG | NEG | NEG | NEG | NEG | NEG | NEG | POS | NEG | NEG | POS | NEG | POS | POS | NEG | POS | NEG | POS | NEG | POS | POS | NEG | NEG | POS | NEG | POS | NEG | POS |
| C. difficile QCD-66c26 (GenBank ABFD) in silico prediction | NEG | NEG | NEG | NEG | NEG | NEG | NEG | NEG | POS | NEG | NEG | POS | NEG | POS | POS | NEG | POS | NEG | POS | NEG | POS | POS | NEG | NEG | POS | NEG | POS | NEG | POS |
| C. difficile R20291 (GenBank FN545816) in silico prediction | NEG | NEG | NEG | NEG | NEG | NEG | NEG | NEG | POS | NEG | NEG | POS | NEG | POS | POS | NEG | POS | NEG | POS | NEG | POS | POS | NEG | NEG | POS | NEG | POS | NEG | POS |
| C. difficile Strain R20291 | NEG | NEG | NEG | NEG | NEG | NEG | NEG | NEG | POS | NEG | NEG | POS | NEG | POS | POS | NEG | POS | NEG | POS | NEG | POS | POS | NEG | NEG | POS | NEG | POS | NEG | POS |
| | NEG | NEG | NEG | NEG | NEG | NEG | NEG | NEG | POS | NEG | NEG | POS | NEG | POS | POS | POS | POS | POS | POS | NEG | POS | POS | NEG | NEG | POS | NEG | POS | NEG | POS |
| | NEG | NEG | NEG | NEG | NEG | NEG | NEG | NEG | POS | NEG | NEG | POS | NEG | POS | POS | NEG | POS | NEG | POS | NEG | POS | POS | NEG | NEG | POS | NEG | POS | NEG | POS |
| | NEG | NEG | NEG | NEG | NEG | NEG | NEG | NEG | POS | NEG | NEG | POS | NEG | POS | POS | NEG | POS | NEG | POS | NEG | POS | POS | NEG | NEG | POS | NEG | POS | NEG | POS |
| **CLADE 3 (CC5 /Ribo 23 Complex)** | | | | | | | | | | | | | | | | | | | | | | | | | | | | | |
| **>HP-33** | NEG | VAR | NEG | NEG | NEG | NEG | NEG | NEG | POS | NEG | NEG | POS | NEG | AMB | POS | NEG | POS | NEG | NEG | NEG | NEG | POS | NEG | NEG | NEG | NEG | NEG | NEG | NEG |
| Freiburg872-34.872-DBK0000295 | NEG | POS | NEG | NEG | NEG | NEG | NEG | NEG | POS | NEG | NEG | POS | AMB | POS | POS | AMB | POS | NEG | NEG | NEG | NEG | POS | NEG | NEG | NEG | NEG | NEG | NEG | NEG |
| | NEG | POS | NEG | NEG | NEG | NEG | NEG | NEG | POS | NEG | NEG | POS | NEG | NEG | POS | NEG | POS | NEG | NEG | NEG | NEG | AMB | NEG | NEG | NEG | NEG | NEG | NEG | NEG |
| Freiburg-773-19.773-DBK0000274 | NEG | NEG | NEG | NEG | NEG | NEG | NEG | NEG | POS | NEG | NEG | POS | AMB | AMB | POS | NEG | POS | NEG | NEG | NEG | NEG | POS | NEG | NEG | NEG | NEG | NEG | NEG | NEG |
| | NEG | NEG | NEG | NEG | NEG | NEG | NEG | NEG | POS | NEG | NEG | POS | NEG | NEG | POS | NEG | POS | NEG | NEG | NEG | NEG | POS | NEG | NEG | NEG | NEG | NEG | NEG | NEG |
| **>HP-34** | NEG | NEG | NEG | NEG | NEG | NEG | NEG | NEG | POS | NEG | NEG | POS | NEG | NEG | POS | NEG | POS | NEG | NEG | NEG | NEG | AMB | NEG | NEG | NEG | NEG | NEG | NEG | NEG |
| Jena_0018/11 | NEG | NEG | NEG | NEG | NEG | NEG | NEG | NEG | POS | NEG | NEG | POS | NEG | NEG | POS | NEG | POS | NEG | NEG | NEG | NEG | AMB | NEG | NEG | NEG | NEG | NEG | NEG | NEG |
| **CLADE 4 (CC37/Ribo 17 Complex)** | | | | | | | | | | | | | | | | | | | | | | | | | | | | | |
| **>HP-35** | NEG | VAR | VAR | NEG | VAR | NEG | VAR | NEG | POS | NEG | NEG | POS | NEG | POS | POS | NEG | POS | POS | NEG | NEG | POS | POS | NEG | NEG | POS | NEG | POS | NEG | POS |
| C. difficile 002-P50-2011 (GenBank AGAA) in silico prediction | NEG | NEG | NEG | NEG | NEG | NEG | NEG | NEG | POS | NEG | NEG | POS | NEG | POS | POS | NEG | POS | POS | NEG | NEG | POS | POS | NEG | NEG | POS | NEG | POS | NEG | POS |
| C. difficile 050-P50-2011 (GenBank AGAB) in silico prediction | NEG | NEG | NEG | NEG | NEG | NEG | NEG | NEG | POS | NEG | NEG | POS | NEG | POS | POS | NEG | POS | POS | NEG | NEG | POS | POS | NEG | NEG | POS | NEG | POS | NEG | POS |
| C. difficile M68 (GenBank FN668375) in silico prediction | NEG | POS | POS | NEG | POS | NEG | POS | NEG | POS | NEG | NEG | POS | NEG | POS | POS | NEG | POS | POS | NEG | NEG | POS | POS | NEG | NEG | POS | NEG | POS | NEG | POS |
| C. difficile CF5 (GenBank FN665652) in silico prediction | NEG | NEG | NEG | NEG | NEG | NEG | NEG | NEG | POS | NEG | NEG | POS | NEG | POS | POS | NEG | POS | POS | NEG | NEG | POS | POS | NEG | NEG | POS | NEG | POS | NEG | POS |
| C. difficile Strain CF5 | NEG | NEG | NEG | NEG | NEG | NEG | NEG | NEG | POS | NEG | NEG | POS | NEG | POS | POS | NEG | POS | POS | NEG | NEG | POS | POS | NEG | NEG | POS | NEG | POS | NEG | POS |
| | NEG | NEG | NEG | NEG | NEG | NEG | NEG | NEG | POS | NEG | NEG | POS | NEG | POS | POS | POS | POS | POS | AMB | NEG | POS | POS | NEG | NEG | POS | NEG | POS | NEG | POS |
| | NEG | NEG | NEG | NEG | NEG | NEG | NEG | NEG | POS | NEG | NEG | POS | NEG | POS | POS | NEG | POS | POS | NEG | NEG | POS | POS | NEG | NEG | POS | NEG | POS | NEG | POS |
| | NEG | NEG | NEG | NEG | NEG | NEG | NEG | NEG | POS | NEG | NEG | POS | AMB | AMB | POS | NEG | POS | POS | NEG | NEG | POS | AMB | NEG | NEG | POS | NEG | POS | NEG | POS |
| | NEG | NEG | NEG | NEG | NEG | NEG | NEG | NEG | POS | NEG | NEG | POS | POS | POS | POS | NEG | POS | POS | NEG | NEG | POS | POS | NEG | NEG | POS | NEG | POS | NEG | POS |
| | NEG | NEG | NEG | NEG | NEG | NEG | NEG | NEG | POS | NEG | NEG | POS | NEG | POS | POS | NEG | POS | POS | NEG | NEG | POS | POS | NEG | NEG | POS | NEG | POS | NEG | POS |
| Freiburg-736-16.736-DBK0000233 | NEG | NEG | NEG | NEG | NEG | NEG | NEG | NEG | POS | NEG | NEG | POS | NEG | POS | POS | POS | POS | POS | AMB | NEG | POS | POS | NEG | NEG | POS | NEG | POS | NEG | POS |
| | NEG | NEG | NEG | NEG | NEG | NEG | NEG | NEG | POS | NEG | NEG | POS | NEG | POS | POS | NEG | POS | POS | NEG | NEG | POS | POS | NEG | NEG | POS | NEG | POS | NEG | POS |
| **>HP-36** | NEG | NEG | NEG | NEG | NEG | NEG | NEG | NEG | POS | NEG | NEG | POS | AMB | POS | POS | NEG | POS | POS | NEG | NEG | POS | POS | NEG | NEG | POS | NEG | NEG | NEG | NEG |
| Freiburg-642-5.642-DBK0000281 | NEG | NEG | NEG | NEG | NEG | NEG | NEG | NEG | POS | NEG | NEG | POS | POS | POS | POS | NEG | POS | POS | NEG | NEG | POS | POS | NEG | NEG | POS | NEG | NEG | NEG | NEG |
| | NEG | NEG | NEG | NEG | NEG | NEG | NEG | NEG | POS | NEG | NEG | POS | NEG | POS | POS | NEG | POS | POS | NEG | NEG | POS | POS | NEG | NEG | POS | NEG | NEG | NEG | NEG |
| **CLADE 5 (CC11 /Ribo 78 Complex)** | | | | | | | | | | | | | | | | | | | | | | | | | | | | | |
| **>HP-37** | NEG | VAR | VAR | NEG | VAR | VAR | NEG | VAR | NEG | POS | POS | NEG | POS | NEG | NEG | POS | POS | POS | NEG | POS | NEG | POS | NEG | POS | POS | NEG | NEG | NEG | NEG |
| C. difficile NAP08 (GenBank ADNX) in silico prediction | NEG | POS | POS | NEG | POS | NEG | POS | NEG | NEG | POS | POS | NEG | POS | NEG | NEG | POS | POS | POS | NEG | POS | NEG | POS | NEG | POS | POS | NEG | NEG | NEG | NEG |
| C. difficile NAP07 (GenBank ADVM) in silico prediction | NEG | POS | POS | NEG | POS | NEG | NEG | POS | NEG | POS | POS | NEG | POS | NEG | NEG | POS | POS | POS | NEG | POS | NEG | POS | NEG | POS | POS | NEG | NEG | NEG | NEG |
| C. difficile M120 (GenBank FN665653) in silico prediction | NEG | NEG | POS | NEG | POS | POS | NEG | POS | NEG | POS | POS | NEG | POS | NEG | NEG | POS | POS | POS | NEG | POS | NEG | POS | NEG | POS | POS | NEG | NEG | NEG | NEG |
| C. difficile Strain M120 | NEG | NEG | POS | NEG | POS | POS | NEG | POS | NEG | POS | POS | NEG | POS | NEG | NEG | POS | POS | POS | NEG | POS | NEG | POS | NEG | POS | POS | NEG | NEG | NEG | NEG |
| | NEG | NEG | POS | NEG | POS | POS | NEG | POS | AMB | POS | POS | NEG | POS | NEG | NEG | POS | POS | POS | AMB | POS | NEG | POS | NEG | POS | POS | NEG | NEG | NEG | NEG |
| | NEG | NEG | POS | NEG | POS | POS | NEG | POS | NEG | POS | POS | NEG | POS | NEG | NEG | POS | POS | POS | NEG | POS | NEG | POS | NEG | POS | POS | NEG | NEG | NEG | NEG |
| | NEG | NEG | POS | NEG | POS | POS | NEG | POS | NEG | POS | POS | NEG | POS | NEG | NEG | POS | POS | POS | NEG | POS | NEG | POS | NEG | AMB | POS | NEG | NEG | NEG | NEG |
| | NEG | NEG | POS | NEG | POS | POS | NEG | POS | NEG | POS | POS | NEG | POS | NEG | NEG | POS | POS | POS | AMB | POS | NEG | POS | NEG | POS | POS | NEG | NEG | NEG | NEG |
| Freiburg_69.1017 | NEG | NEG | NEG | NEG | NEG | NEG | NEG | NEG | NEG | POS | POS | NEG | AMB | NEG | NEG | POS | POS | POS | NEG | POS | NEG | NEG | NEG | AMB | POS | NEG | NEG | NEG | NEG |
| Freiburg-1051-77.1051-DBK0000293 | NEG | NEG | NEG | NEG | NEG | NEG | NEG | NEG | AMB | POS | POS | NEG | POS | NEG | NEG | POS | POS | POS | NEG | POS | NEG | POS | NEG | POS | POS | NEG | NEG | NEG | NEG |
| | NEG | NEG | NEG | NEG | NEG | NEG | NEG | NEG | NEG | POS | POS | NEG | POS | NEG | NEG | POS | POS | POS | NEG | POS | NEG | POS | NEG | POS | POS | NEG | NEG | NEG | NEG |
| Freiburg-649-6.649-DBK0000235 | NEG | NEG | NEG | NEG | NEG | NEG | NEG | NEG | AMB | POS | POS | NEG | POS | NEG | NEG | POS | POS | POS | AMB | POS | NEG | POS | NEG | POS | POS | NEG | NEG | NEG | NEG |
| | NEG | NEG | NEG | NEG | NEG | NEG | NEG | NEG | NEG | POS | POS | NEG | POS | NEG | NEG | POS | POS | POS | NEG | POS | NEG | POS | NEG | POS | POS | NEG | NEG | NEG | NEG |
| Freiburg-700-11.7-DBK0000242 | NEG | NEG | POS | NEG | POS | NEG | POS | NEG | NEG | POS | POS | NEG | POS | NEG | NEG | POS | POS | POS | NEG | POS | NEG | POS | NEG | POS | POS | NEG | NEG | NEG | NEG |
| | NEG | NEG | POS | NEG | POS | NEG | POS | NEG | NEG | POS | POS | NEG | POS | NEG | NEG | POS | POS | POS | NEG | POS | NEG | POS | NEG | POS | POS | NEG | NEG | NEG | NEG |
| Freiburg-855-30.855-DBK0000277 | NEG | POS | POS | NEG | POS | NEG | POS | NEG | AMB | POS | POS | NEG | POS | NEG | NEG | POS | POS | POS | NEG | POS | NEG | POS | NEG | POS | POS | NEG | NEG | NEG | NEG |
| | NEG | POS | POS | NEG | POS | NEG | POS | NEG | NEG | POS | POS | NEG | POS | NEG | NEG | POS | POS | POS | NEG | POS | NEG | POS | NEG | POS | POS | NEG | NEG | NEG | NEG |
| Dresden 08_1353 | NEG | NEG | NEG | NEG | NEG | NEG | NEG | NEG | AMB | POS | POS | NEG | POS | NEG | NEG | POS | POS | POS | NEG | POS | NEG | POS | NEG | POS | POS | NEG | NEG | NEG | NEG |
| Dresden 08_574 | NEG | NEG | NEG | NEG | NEG | NEG | NEG | NEG | AMB | POS | POS | NEG | POS | NEG | NEG | POS | POS | POS | NEG | POS | NEG | POS | NEG | POS | POS | NEG | NEG | NEG | NEG |
| Dresden 09_2650 | NEG | NEG | NEG | NEG | NEG | NEG | NEG | NEG | NEG | POS | POS | NEG | POS | NEG | NEG | POS | POS | POS | NEG | POS | NEG | POS | NEG | POS | POS | NEG | NEG | NEG | NEG |
| **>HP-38** | NEG | NEG | POS | NEG | POS | NEG | NEG | POS | NEG | POS | POS | NEG | POS | NEG | NEG | POS | POS | POS | NEG | POS | NEG | POS | NEG | POS | POS | NEG | POS | NEG | POS |
| C. difficile 6466 (GenBank ADDE) in silico prediction | NEG | NEG | POS | NEG | POS | NEG | NEG | POS | NEG | POS | POS | NEG | POS | NEG | NEG | POS | POS | POS | NEG | POS | NEG | POS | NEG | POS | POS | NEG | POS | NEG | POS |
| **>HP-39** | NEG | NEG | NEG | NEG | NEG | NEG | NEG | NEG | NEG | POS | POS | NEG | POS | NEG | NEG | POS | POS | POS | NEG | POS | NEG | POS | NEG | POS | POS | NEG | NEG | NEG | NEG |
| C. difficile QCD-23m63 (GenBank ABKL) in silico prediction | NEG | NEG | NEG | NEG | NEG | NEG | NEG | NEG | NEG | POS | POS | NEG | POS | NEG | NEG | POS | POS | POS | NEG | POS | NEG | POS | NEG | POS | POS | NEG | NEG | NEG | NEG |
| Freiburg-956-51.956-DBK0000266 | NEG | NEG | NEG | NEG | NEG | NEG | NEG | NEG | AMB | POS | POS | NEG | POS | NEG | NEG | POS | POS | POS | NEG | POS | NEG | POS | NEG | POS | POS | NEG | NEG | NEG | NEG |
| Freiburg_50.963 | NEG | NEG | NEG | NEG | NEG | NEG | NEG | NEG | NEG | POS | POS | NEG | POS | NEG | NEG | POS | POS | POS | NEG | POS | NEG | POS | NEG | POS | POS | NEG | NEG | NEG | NEG |
| **"CLADE 6"** | | | | | | | | | | | | | | | | | | | | | | | | | | | | | |
| **>HP-40** | NEG | NEG | NEG | NEG | NEG | NEG | NEG | NEG | POS | NEG | NEG | POS | NEG | POS | POS | NEG | POS | POS | NEG | NEG | POS | POS | NEG | NEG | POS | NEG | NEG | NEG | NEG |
| C. difficile 6503 (GenBank ADEI) in silico prediction | NEG | NEG | NEG | NEG | NEG | NEG | NEG | NEG | POS | NEG | NEG | POS | NEG | POS | POS | NEG | POS | POS | NEG | NEG | POS | POS | NEG | NEG | POS | NEG | NEG | NEG | NEG |

| STRAIN / ISOLATE | Clostridium difficile binary toxin component A | | | | Clostridium difficile binary toxin component B | | | | | | channel-forming hemolysin | holin-like pore-forming protein | putative negative regulator of pathogenicity locus | cellwall protein 66 | | | | cellwall protein 84 | septum formation initiation protein | | | flagellin subunit C | | | | |
|---|---|---|---|---|---|---|---|---|---|---|---|---|---|---|---|---|---|---|---|---|---|---|---|---|---|---|
| | hp1023\|cdtA | hp1026\|cdtA | hp1027\|cdtA | hp1029\|cdtA | hp1030\|cdtB | hp1031\|cdtB | hp1038\|cdtB | hp1039\|cdtB | hp1040\|cdtB | hp1041\|cdtB | hp1103\|hly3 | hp1113\|tcdE | hp1117\|tcdC | hp1012\|cwp66 | hp1016\|cwp66 | hp1020\|cwp66 | hp1021\|cwp66 | hp1018\|cwp84 | hp1044\|divIC | hp1045\|divIC | hp1046\|divIC | hp1141\|fliC | hp1142\|fliC | hp1144\|fliC | hp1145\|fliC | hp1147\|fliC |
| **CLADE 1** | | | | | | | | | | | | | | | | | | | | | | | | | | |
| **>HP-01** | AMB | POS | NEG | NEG | NEG | NEG | POS | POS | NEG | NEG | POS | POS | POS | POS | NEG | NEG | NEG | POS | POS | NEG | NEG | NEG | POS | NEG | NEG | POS |
| C. difficile QCD-63q42 (GenBank ABHD) in silico prediction | POS | POS | NEG | NEG | NEG | NEG | POS | POS | NEG | NEG | POS | POS | POS | POS | NEG | NEG | NEG | POS | POS | NEG | POS | NEG | POS | NEG | NEG | POS |
| C. difficile BI9 (GenBank FN668944) in silico prediction | POS | POS | NEG | NEG | NEG | NEG | POS | POS | NEG | NEG | POS | POS | POS | POS | NEG | NEG | NEG | POS | POS | NEG | POS | NEG | POS | NEG | NEG | POS |
| C. difficile BI-9 | NEG | POS | NEG | NEG | NEG | NEG | AMB | POS | NEG | NEG | POS | POS | POS | POS | NEG | NEG | NEG | POS | AMB | NEG | NEG | NEG | NEG | NEG | NEG | POS |
| | AMB | POS | NEG | NEG | NEG | NEG | POS | POS | NEG | NEG | POS | POS | POS | POS | NEG | NEG | NEG | POS | AMB | NEG | NEG | NEG | AMB | NEG | NEG | POS |
| | AMB | POS | NEG | NEG | NEG | NEG | POS | POS | NEG | NEG | POS | POS | POS | POS | NEG | NEG | NEG | POS | POS | NEG | NEG | NEG | POS | NEG | NEG | POS |
| | AMB | POS | NEG | NEG | NEG | NEG | POS | POS | NEG | NEG | POS | POS | POS | POS | NEG | NEG | NEG | POS | AMB | NEG | NEG | NEG | AMB | NEG | NEG | POS |
| | NEG | AMB | NEG | NEG | NEG | NEG | POS | POS | NEG | NEG | POS | POS | AMB | POS | NEG | NEG | NEG | POS | AMB | NEG | NEG | NEG | AMB | NEG | NEG | POS |
| Freiburg-1040-73.104-DBK0000249 | POS | POS | NEG | NEG | NEG | NEG | POS | POS | NEG | NEG | POS | POS | POS | POS | NEG | NEG | NEG | POS | POS | NEG | AMB | NEG | POS | NEG | NEG | POS |
| | NEG | AMB | NEG | NEG | NEG | NEG | POS | POS | NEG | NEG | POS | POS | AMB | POS | NEG | NEG | NEG | POS | AMB | NEG | NEG | NEG | AMB | NEG | NEG | POS |
| Freiburg-1052-78.1052-DBK0000294 | POS | POS | NEG | NEG | NEG | NEG | POS | POS | NEG | NEG | POS | POS | POS | POS | NEG | NEG | NEG | POS | POS | NEG | NEG | NEG | POS | NEG | NEG | POS |
| | NEG | POS | NEG | NEG | NEG | NEG | POS | POS | NEG | NEG | POS | POS | POS | POS | NEG | NEG | NEG | POS | POS | NEG | NEG | NEG | POS | NEG | NEG | POS |
| Freiburg-605-1.605-DBK0000223 | POS | POS | NEG | NEG | NEG | NEG | POS | POS | NEG | NEG | POS | POS | POS | POS | NEG | NEG | NEG | POS | POS | NEG | AMB | NEG | POS | NEG | NEG | POS |
| Freiburg-787-22.787-DBK0000288 | AMB | POS | NEG | NEG | NEG | NEG | POS | POS | NEG | NEG | POS | POS | POS | POS | NEG | NEG | NEG | POS | POS | NEG | NEG | NEG | POS | NEG | NEG | POS |
| | AMB | POS | NEG | NEG | NEG | NEG | POS | POS | NEG | NEG | POS | POS | POS | POS | NEG | NEG | NEG | POS | POS | NEG | NEG | NEG | POS | NEG | NEG | POS |
| Freiburg-897-40.897-DBK0000225 | POS | POS | NEG | NEG | NEG | NEG | POS | POS | NEG | NEG | POS | POS | POS | POS | NEG | NEG | NEG | POS | POS | NEG | NEG | NEG | POS | NEG | NEG | POS |
| | NEG | NEG | NEG | NEG | NEG | NEG | POS | POS | NEG | NEG | POS | POS | POS | AMB | NEG | NEG | NEG | POS | NEG | NEG | NEG | NEG | NEG | NEG | NEG | AMB |
| Dresden 06_7735 | NEG | AMB | NEG | NEG | NEG | NEG | POS | POS | NEG | NEG | POS | POS | POS | POS | NEG | NEG | NEG | POS | AMB | NEG | NEG | NEG | AMB | NEG | NEG | POS |
| Dresden 06_7736 | NEG | NEG | NEG | NEG | NEG | NEG | AMB | POS | NEG | NEG | POS | POS | AMB | AMB | NEG | NEG | NEG | POS | NEG | NEG | NEG | NEG | AMB | NEG | NEG | AMB |
| Dresden 07_6943 | AMB | AMB | NEG | NEG | NEG | NEG | POS | POS | NEG | NEG | POS | POS | POS | POS | NEG | NEG | NEG | POS | AMB | NEG | NEG | NEG | POS | NEG | NEG | POS |
| Dresden 07_7200 | NEG | NEG | NEG | NEG | NEG | NEG | NEG | POS | NEG | NEG | AMB | AMB | AMB | NEG | NEG | NEG | NEG | AMB | NEG | NEG | NEG | NEG | NEG | NEG | NEG | NEG |
| Dresden 07_7269 | AMB | AMB | NEG | NEG | NEG | NEG | POS | POS | NEG | NEG | POS | POS | POS | POS | NEG | NEG | NEG | POS | POS | NEG | NEG | NEG | POS | NEG | NEG | POS |
| Dresden 07_7678 | AMB | POS | NEG | NEG | NEG | NEG | POS | POS | NEG | NEG | POS | POS | POS | POS | NEG | NEG | NEG | POS | POS | NEG | NEG | NEG | POS | NEG | NEG | POS |
| Dresden 07_7773 | AMB | POS | NEG | NEG | NEG | NEG | POS | POS | NEG | NEG | POS | POS | POS | POS | NEG | NEG | NEG | POS | POS | NEG | NEG | NEG | POS | NEG | NEG | POS |
| Dresden 07_7832 | AMB | AMB | NEG | NEG | NEG | NEG | POS | POS | NEG | NEG | POS | POS | POS | POS | NEG | NEG | NEG | POS | AMB | NEG | NEG | NEG | AMB | NEG | NEG | POS |
| Dresden 07_7967 | NEG | AMB | NEG | NEG | NEG | NEG | POS | POS | NEG | NEG | POS | POS | POS | POS | NEG | NEG | NEG | POS | AMB | NEG | NEG | NEG | AMB | NEG | NEG | POS |
| Dresden 07_8099 | AMB | POS | NEG | NEG | NEG | NEG | POS | POS | NEG | NEG | POS | POS | POS | POS | NEG | NEG | NEG | POS | POS | NEG | NEG | NEG | POS | NEG | NEG | POS |
| Dresden 07_8100 | POS | POS | NEG | NEG | NEG | NEG | POS | POS | NEG | NEG | POS | POS | POS | POS | NEG | NEG | NEG | POS | POS | NEG | NEG | NEG | POS | NEG | NEG | POS |
| Dresden 07_8170 | AMB | POS | NEG | NEG | NEG | NEG | POS | POS | NEG | NEG | POS | POS | POS | POS | NEG | NEG | NEG | POS | POS | NEG | NEG | NEG | POS | NEG | NEG | POS |
| Dresden 08_0544 | AMB | AMB | NEG | NEG | NEG | NEG | POS | POS | NEG | NEG | POS | POS | POS | POS | NEG | NEG | NEG | POS | POS | NEG | NEG | NEG | POS | NEG | NEG | POS |
| Dresden 08_0550 | NEG | AMB | NEG | NEG | NEG | NEG | POS | POS | NEG | NEG | POS | POS | POS | POS | NEG | NEG | NEG | POS | AMB | NEG | NEG | NEG | AMB | NEG | NEG | POS |
| Dresden 08_0827 | AMB | POS | NEG | NEG | NEG | NEG | POS | POS | NEG | NEG | POS | POS | POS | POS | NEG | NEG | NEG | POS | POS | NEG | NEG | NEG | POS | NEG | NEG | POS |
| Dresden 08_0902 | NEG | AMB | NEG | NEG | NEG | NEG | POS | POS | NEG | NEG | POS | POS | POS | POS | NEG | NEG | NEG | POS | POS | NEG | NEG | NEG | POS | NEG | NEG | POS |
| Dresden 08_0955 | AMB | POS | NEG | NEG | NEG | NEG | POS | POS | NEG | NEG | POS | POS | POS | POS | NEG | NEG | NEG | POS | POS | NEG | NEG | NEG | POS | NEG | NEG | POS |
| Dresden 08_0958 | NEG | AMB | NEG | NEG | NEG | NEG | POS | POS | NEG | NEG | POS | POS | AMB | POS | NEG | NEG | NEG | POS | AMB | NEG | NEG | NEG | AMB | NEG | NEG | POS |
| Dresden 08_1073 | NEG | AMB | NEG | NEG | NEG | NEG | POS | POS | NEG | NEG | POS | POS | POS | POS | NEG | NEG | NEG | POS | AMB | NEG | NEG | NEG | POS | NEG | NEG | POS |
| Dresden 08_1074 | NEG | AMB | NEG | NEG | NEG | NEG | POS | POS | NEG | NEG | POS | POS | POS | POS | NEG | NEG | NEG | POS | AMB | NEG | NEG | NEG | AMB | NEG | NEG | POS |
| Dresden 08_1206 | NEG | AMB | NEG | NEG | NEG | NEG | POS | POS | NEG | NEG | POS | POS | POS | POS | NEG | NEG | NEG | POS | AMB | NEG | NEG | NEG | POS | NEG | NEG | POS |
| Dresden 08_1216 | NEG | NEG | NEG | NEG | NEG | NEG | AMB | POS | NEG | NEG | POS | POS | AMB | AMB | NEG | NEG | NEG | POS | NEG | NEG | NEG | NEG | AMB | NEG | NEG | POS |
| Dresden 08_1305 | NEG | AMB | NEG | NEG | NEG | NEG | POS | POS | NEG | NEG | POS | POS | AMB | POS | NEG | NEG | NEG | POS | POS | NEG | NEG | NEG | POS | NEG | NEG | POS |
| Dresden 07_7679 | NEG | NEG | NEG | NEG | NEG | NEG | AMB | POS | NEG | NEG | POS | POS | AMB | AMB | NEG | NEG | NEG | POS | NEG | NEG | NEG | NEG | NEG | NEG | NEG | AMB |
| Dresden 07_7683 | NEG | AMB | NEG | NEG | NEG | NEG | POS | POS | NEG | NEG | POS | POS | POS | POS | NEG | NEG | NEG | POS | AMB | NEG | NEG | NEG | AMB | NEG | NEG | POS |
| Dresden 07_7980 | NEG | NEG | NEG | NEG | NEG | NEG | AMB | POS | NEG | NEG | POS | POS | POS | AMB | NEG | NEG | NEG | POS | NEG | NEG | NEG | NEG | AMB | NEG | NEG | AMB |
| Dresden 07_8022 | NEG | AMB | NEG | NEG | NEG | NEG | POS | POS | NEG | NEG | POS | POS | POS | POS | NEG | NEG | NEG | POS | AMB | NEG | NEG | NEG | AMB | NEG | NEG | POS |
| Dresden 07_8291 | NEG | NEG | NEG | NEG | NEG | NEG | AMB | POS | NEG | NEG | POS | POS | AMB | AMB | NEG | NEG | NEG | POS | NEG | NEG | NEG | NEG | NEG | NEG | NEG | AMB |
| Dresden 07_8314 | AMB | POS | NEG | NEG | NEG | NEG | POS | POS | NEG | NEG | POS | POS | POS | POS | NEG | NEG | NEG | POS | POS | NEG | NEG | NEG | POS | NEG | NEG | POS |
| Dresden 07_8315 | NEG | NEG | NEG | NEG | NEG | NEG | AMB | POS | NEG | NEG | POS | POS | POS | POS | NEG | NEG | NEG | POS | NEG | NEG | NEG | NEG | AMB | NEG | NEG | AMB |
| Dresden 07_8323 | NEG | NEG | NEG | NEG | NEG | NEG | NEG | POS | NEG | NEG | POS | POS | AMB | AMB | NEG | NEG | NEG | POS | NEG | NEG | NEG | NEG | NEG | NEG | NEG | AMB |
| Dresden 07_8356 | NEG | AMB | NEG | NEG | NEG | NEG | POS | POS | NEG | NEG | POS | POS | POS | POS | NEG | NEG | NEG | POS | AMB | NEG | NEG | NEG | AMB | NEG | NEG | POS |
| Dresden 07_8557 | NEG | NEG | NEG | NEG | NEG | NEG | NEG | POS | NEG | NEG | POS | AMB | AMB | AMB | NEG | NEG | NEG | AMB | NEG | NEG | NEG | NEG | NEG | NEG | NEG | AMB |
| Dresden 07_8736 | NEG | AMB | NEG | NEG | NEG | NEG | AMB | POS | NEG | NEG | POS | POS | POS | POS | NEG | NEG | NEG | POS | NEG | NEG | NEG | NEG | AMB | NEG | NEG | POS |
| Dresden 07_8945 | NEG | AMB | NEG | NEG | NEG | NEG | POS | POS | NEG | NEG | POS | POS | POS | POS | NEG | NEG | NEG | POS | AMB | NEG | NEG | NEG | POS | NEG | NEG | POS |
| Dresden 07_9138 | NEG | POS | NEG | NEG | NEG | NEG | POS | POS | NEG | NEG | POS | POS | POS | POS | NEG | NEG | NEG | POS | POS | NEG | NEG | NEG | POS | NEG | NEG | POS |
| Dresden 07_9175 | AMB | POS | NEG | NEG | NEG | NEG | POS | POS | NEG | NEG | POS | POS | POS | POS | NEG | NEG | NEG | POS | POS | NEG | NEG | NEG | POS | NEG | NEG | POS |
| Dresden 07_9234 | AMB | POS | NEG | NEG | NEG | NEG | POS | POS | NEG | NEG | POS | POS | POS | POS | NEG | NEG | NEG | POS | POS | NEG | NEG | NEG | POS | NEG | NEG | POS |
| Dresden 07_9255 | POS | POS | NEG | NEG | NEG | NEG | POS | POS | NEG | NEG | POS | POS | POS | POS | NEG | NEG | NEG | POS | POS | NEG | NEG | NEG | POS | NEG | NEG | POS |
| Dresden 07_9309 | AMB | POS | NEG | NEG | NEG | NEG | POS | POS | NEG | NEG | POS | POS | POS | POS | NEG | NEG | NEG | POS | POS | NEG | NEG | NEG | POS | NEG | NEG | POS |
| Dresden 07_9330 | AMB | POS | NEG | NEG | NEG | NEG | POS | POS | NEG | NEG | POS | POS | POS | POS | NEG | NEG | NEG | POS | POS | NEG | NEG | NEG | POS | NEG | NEG | POS |
| Dresden 07_9399 | AMB | POS | NEG | NEG | NEG | NEG | POS | POS | NEG | NEG | POS | POS | POS | POS | NEG | NEG | NEG | POS | POS | NEG | NEG | NEG | POS | NEG | NEG | POS |
| Dresden 08_0188 | POS | POS | NEG | NEG | NEG | NEG | POS | POS | NEG | NEG | POS | POS | POS | POS | NEG | NEG | NEG | POS | POS | NEG | NEG | NEG | POS | NEG | NEG | POS |
| Dresden 08_0190 | AMB | POS | NEG | NEG | NEG | NEG | POS | POS | NEG | NEG | POS | POS | POS | POS | NEG | NEG | NEG | POS | POS | NEG | NEG | NEG | POS | NEG | NEG | POS |
| Dresden 08_0220 | POS | POS | NEG | NEG | NEG | NEG | POS | POS | NEG | NEG | POS | POS | POS | POS | NEG | NEG | NEG | POS | POS | NEG | NEG | NEG | POS | NEG | NEG | POS |
| Dresden 08_0326 | AMB | POS | NEG | NEG | NEG | NEG | POS | POS | NEG | NEG | POS | POS | POS | POS | NEG | NEG | NEG | POS | POS | NEG | NEG | NEG | POS | NEG | NEG | POS |
| Dresden 08_0327 | POS | POS | NEG | NEG | NEG | NEG | POS | POS | NEG | NEG | POS | POS | POS | POS | NEG | NEG | NEG | POS | POS | NEG | NEG | NEG | POS | NEG | NEG | POS |
| Dresden 08_0648 | POS | POS | NEG | NEG | NEG | NEG | POS | POS | NEG | NEG | POS | POS | POS | POS | NEG | NEG | NEG | POS | POS | NEG | AMB | NEG | POS | NEG | NEG | POS |
| Dresden 08_0751 | POS | POS | NEG | NEG | NEG | NEG | POS | POS | NEG | NEG | POS | POS | POS | POS | NEG | NEG | NEG | POS | POS | NEG | NEG | NEG | POS | NEG | NEG | POS |
| Dresden 08_0769 | POS | POS | NEG | NEG | NEG | NEG | POS | POS | NEG | NEG | POS | POS | POS | POS | NEG | NEG | NEG | POS | POS | NEG | NEG | NEG | POS | NEG | NEG | POS |
| Dresden 08_0770 | POS | POS | NEG | NEG | NEG | NEG | POS | POS | NEG | NEG | POS | POS | POS | POS | NEG | NEG | NEG | POS | POS | NEG | NEG | NEG | POS | NEG | NEG | POS |
| Dresden 08_0771 | POS | POS | NEG | NEG | NEG | NEG | POS | POS | NEG | NEG | POS | POS | POS | POS | NEG | NEG | NEG | POS | POS | NEG | AMB | NEG | POS | NEG | NEG | POS |
| Dresden 08_1308 | POS | POS | NEG | NEG | NEG | NEG | POS | POS | NEG | NEG | POS | POS | POS | POS | NEG | NEG | NEG | POS | POS | NEG | NEG | NEG | POS | NEG | NEG | POS |
| Dresden 08_1311 | AMB | POS | NEG | NEG | NEG | NEG | POS | POS | NEG | NEG | POS | POS | POS | POS | NEG | NEG | NEG | POS | POS | NEG | NEG | NEG | POS | NEG | NEG | POS |
| Dresden 08_1316 | AMB | POS | NEG | NEG | NEG | NEG | POS | POS | NEG | NEG | POS | POS | POS | POS | NEG | NEG | NEG | POS | POS | NEG | NEG | NEG | POS | NEG | NEG | POS |
| Dresden 08_1542 | POS | POS | NEG | NEG | NEG | NEG | POS | POS | NEG | NEG | POS | POS | POS | POS | NEG | NEG | NEG | POS | POS | NEG | AMB | NEG | POS | NEG | NEG | POS |
| Dresden 08_1544 | POS | POS | NEG | NEG | NEG | NEG | POS | POS | NEG | NEG | POS | POS | POS | POS | NEG | NEG | NEG | POS | POS | NEG | NEG | NEG | POS | NEG | NEG | POS |
| Dresden 08_1581 | AMB | POS | NEG | NEG | NEG | NEG | POS | POS | NEG | NEG | POS | POS | POS | POS | NEG | NEG | NEG | POS | POS | NEG | NEG | NEG | POS | NEG | NEG | POS |
| Dresden 08_1826 | AMB | POS | NEG | NEG | NEG | NEG | POS | POS | NEG | NEG | POS | POS | POS | POS | NEG | NEG | NEG | POS | POS | NEG | NEG | NEG | POS | NEG | NEG | POS |
| Dresden 08_1817 | AMB | POS | NEG | NEG | NEG | NEG | POS | POS | NEG | NEG | POS | POS | POS | POS | NEG | NEG | NEG | POS | POS | NEG | NEG | NEG | POS | NEG | NEG | POS |
| Dresden 08_1791 | NEG | AMB | NEG | NEG | NEG | NEG | POS | POS | NEG | NEG | POS | POS | POS | POS | NEG | NEG | NEG | POS | POS | NEG | NEG | NEG | POS | NEG | NEG | POS |
| Dresden 08_1643 | NEG | POS | NEG | NEG | NEG | NEG | POS | POS | NEG | NEG | POS | POS | POS | POS | NEG | NEG | NEG | POS | POS | NEG | NEG | NEG | POS | NEG | NEG | POS |
| Dresden 08_1764 | NEG | AMB | NEG | NEG | NEG | NEG | POS | POS | NEG | NEG | POS | POS | POS | POS | NEG | NEG | NEG | POS | POS | NEG | NEG | NEG | POS | NEG | NEG | POS |
| Dresden 08_1748 | AMB | POS | NEG | NEG | NEG | NEG | POS | POS | NEG | NEG | POS | POS | POS | POS | NEG | NEG | NEG | POS | POS | NEG | NEG | NEG | POS | NEG | NEG | POS |
| Dresden 08_1736 | AMB | POS | NEG | NEG | NEG | NEG | POS | POS | NEG | NEG | POS | POS | POS | POS | NEG | NEG | NEG | POS | POS | NEG | NEG | NEG | POS | NEG | NEG | POS |
| Dresden 08_1582 | AMB | POS | NEG | NEG | NEG | NEG | POS | POS | NEG | NEG | POS | POS | POS | POS | NEG | NEG | NEG | POS | POS | NEG | NEG | NEG | POS | NEG | NEG | POS |
| **>HP-02** | AMB | POS | NEG | NEG | NEG | NEG | POS | POS | NEG | NEG | POS | POS | POS | POS | NEG | NEG | NEG | POS | POS | NEG | NEG | NEG | POS | NEG | NEG | POS |
| C. difficile ATCC43255 (GenBank ABKJ) in silico prediction | POS | POS | NEG | NEG | NEG | NEG | POS | POS | NEG | NEG | POS | POS | POS | POS | NEG | NEG | NEG | POS | POS | NEG | POS | NEG | POS | NEG | NEG | POS |
| Freiburg-885-36.885-DBK0000296 | AMB | POS | NEG | NEG | NEG | NEG | POS | POS | NEG | NEG | POS | POS | POS | POS | NEG | NEG | NEG | POS | AMB | NEG | NEG | NEG | AMB | NEG | NEG | POS |
| Freiburg_37.894-II | NEG | NEG | NEG | NEG | NEG | NEG | AMB | POS | NEG | NEG | POS | POS | AMB | POS | NEG | NEG | NEG | POS | AMB | NEG | NEG | NEG | AMB | NEG | NEG | POS |
| Freiburg_981-57.981-DBK0000258 | NEG | AMB | NEG | NEG | NEG | NEG | AMB | POS | NEG | NEG | POS | POS | AMB | POS | NEG | NEG | NEG | POS | NEG | NEG | NEG | NEG | NEG | NEG | NEG | POS |
| | POS | POS | NEG | NEG | NEG | NEG | POS | POS | NEG | NEG | POS | POS | POS | POS | NEG | NEG | NEG | POS | POS | NEG | NEG | NEG | POS | NEG | NEG | POS |
| Jena_S0011_11 | NEG | POS | NEG | NEG | NEG | NEG | POS | POS | NEG | NEG | POS | POS | POS | POS | NEG | NEG | NEG | POS | POS | NEG | NEG | NEG | POS | NEG | NEG | POS |
| | NEG | POS | NEG | NEG | NEG | NEG | POS | POS | NEG | NEG | POS | POS | POS | POS | NEG | NEG | NEG | POS | POS | NEG | NEG | NEG | POS | NEG | NEG | POS |
| Dresden 08_1352 | POS | POS | NEG | NEG | NEG | NEG | POS | POS | NEG | NEG | POS | POS | POS | POS | NEG | NEG | NEG | POS | POS | NEG | AMB | NEG | POS | NEG | NEG | POS |
| **>HP-03** | AMB | POS | NEG | NEG | NEG | NEG | POS | POS | NEG | NEG | POS | POS | POS | NEG | NEG | POS | NEG | POS | POS | NEG | NEG | NEG | POS | NEG | NEG | POS |
| Freiburg-938-47.938-DBK0000237 | AMB | POS | NEG | NEG | NEG | NEG | POS | POS | NEG | NEG | POS | POS | POS | NEG | NEG | POS | NEG | POS | POS | NEG | NEG | NEG | POS | NEG | NEG | POS |
| | AMB | POS | NEG | NEG | NEG | NEG | POS | POS | NEG | NEG | POS | POS | POS | NEG | NEG | POS | NEG | POS | POS | NEG | NEG | NEG | POS | NEG | NEG | POS |
| Freiburg-811-25.811-DBK0000284 | NEG | AMB | NEG | NEG | NEG | NEG | AMB | POS | NEG | NEG | POS | POS | POS | NEG | NEG | AMB | NEG | POS | AMB | NEG | NEG | NEG | AMB | NEG | NEG | POS |
| | NEG | AMB | NEG | NEG | NEG | NEG | POS | POS | NEG | NEG | POS | POS | POS | NEG | NEG | AMB | NEG | POS | AMB | NEG | NEG | NEG | POS | NEG | NEG | POS |
| Freiburg-987-62.987-DBK0000251 | NEG | POS | NEG | NEG | NEG | NEG | POS | POS | NEG | NEG | POS | POS | POS | NEG | NEG | POS | NEG | POS | POS | NEG | NEG | NEG | POS | NEG | NEG | POS |
| | NEG | AMB | NEG | NEG | NEG | NEG | POS | POS | NEG | NEG | POS | POS | AMB | NEG | NEG | NEG | NEG | POS | NEG | NEG | NEG | NEG | NEG | NEG | NEG | POS |
| Freiburg-972-54.972-DBK0000247 | AMB | POS | NEG | NEG | NEG | NEG | POS | POS | NEG | NEG | POS | POS | POS | NEG | NEG | POS | NEG | POS | POS | NEG | NEG | NEG | POS | NEG | NEG | POS |
| | NEG | AMB | NEG | NEG | NEG | NEG | POS | POS | NEG | NEG | POS | POS | POS | NEG | NEG | POS | NEG | POS | POS | NEG | NEG | NEG | POS | NEG | NEG | POS |
| **>HP-04** | VAR | VAR | NEG | NEG | NEG | NEG | VAR | VAR | NEG | NEG | POS | POS | POS | NEG | POS | NEG | NEG | POS | POS | NEG | NEG | NEG | POS | NEG | NEG | POS |
| Freiburg-984-60.984-DBK0000250 | NEG | POS | NEG | NEG | NEG | NEG | POS | POS | NEG | NEG | POS | POS | POS | NEG | POS | NEG | NEG | POS | POS | NEG | NEG | NEG | POS | NEG | NEG | POS |
| Freiburg-1023-72.1023-DBK0000271 | NEG | POS | NEG | NEG | NEG | NEG | POS | POS | NEG | NEG | POS | POS | POS | NEG | POS | NEG | NEG | POS | POS | NEG | AMB | NEG | POS | NEG | NEG | POS |
| | NEG | POS | NEG | NEG | NEG | NEG | POS | POS | NEG | NEG | POS | POS | POS | NEG | POS | NEG | NEG | POS | POS | NEG | NEG | NEG | POS | NEG | NEG | POS |
| Freiburg-1043-74.1043-DBK0000268 | AMB | POS | NEG | NEG | NEG | NEG | POS | POS | NEG | NEG | POS | POS | POS | NEG | POS | NEG | NEG | POS | POS | NEG | NEG | NEG | POS | NEG | NEG | POS |
| Freiburg-633-2.663-DBK0000292 | NEG | NEG | NEG | NEG | NEG | NEG | NEG | AMB | NEG | NEG | POS | POS | POS | NEG | POS | NEG | NEG | POS | POS | NEG | NEG | NEG | POS | NEG | NEG | POS |
| | NEG | NEG | NEG | NEG | NEG | NEG | NEG | NEG | NEG | NEG | POS | POS | POS | NEG | POS | NEG | NEG | POS | NEG | NEG | NEG | NEG | NEG | NEG | NEG | POS |
| Freiburg-782-20.782-DBK0000276 | AMB | POS | NEG | NEG | NEG | NEG | POS | POS | NEG | NEG | POS | POS | POS | NEG | POS | NEG | NEG | POS | POS | NEG | NEG | NEG | POS | NEG | NEG | POS |
| | NEG | AMB | NEG | NEG | NEG | NEG | POS | POS | NEG | NEG | POS | POS | AMB | NEG | POS | NEG | NEG | POS | AMB | NEG | NEG | NEG | POS | NEG | NEG | POS |
| **>HP-05** | NEG | POS | NEG | NEG | NEG | NEG | POS | POS | NEG | NEG | POS | POS | POS | NEG | NEG | NEG | NEG | POS | POS | NEG | NEG | NEG | POS | NEG | NEG | POS |
| Freiburg-740-17.74-DBK0000234 | NEG | POS | NEG | NEG | NEG | NEG | POS | POS | NEG | NEG | POS | POS | POS | NEG | NEG | NEG | NEG | POS | POS | NEG | NEG | NEG | POS | NEG | NEG | POS |
| | NEG | AMB | NEG | NEG | NEG | NEG | POS | POS | NEG | NEG | POS | POS | POS | NEG | NEG | NEG | NEG | POS | AMB | NEG | NEG | NEG | POS | NEG | NEG | POS |
| Freiburg-793-23.793-DBK0000286 | NEG | POS | NEG | NEG | NEG | NEG | POS | POS | NEG | NEG | POS | POS | POS | NEG | NEG | NEG | NEG | POS | POS | NEG | NEG | NEG | POS | NEG | NEG | POS |
| | NEG | AMB | NEG | NEG | NEG | NEG | POS | POS | NEG | NEG | POS | POS | POS | NEG | NEG | NEG | NEG | POS | AMB | NEG | NEG | NEG | POS | NEG | NEG | POS |
| **>HP-06** | POS | POS | NEG | NEG | NEG | NEG | POS | POS | NEG | NEG | POS | POS | POS | NEG | NEG | NEG | NEG | POS | POS | NEG | NEG | NEG | POS | NEG | NEG | POS |
| Freiburg-874-35.874-DBK0000282 | POS | POS | NEG | NEG | NEG | NEG | POS | POS | NEG | NEG | POS | POS | POS | NEG | NEG | NEG | NEG | POS | POS | NEG | NEG | NEG | POS | NEG | NEG | POS |
| | AMB | POS | NEG | NEG | NEG | NEG | POS | POS | NEG | NEG | POS | POS | POS | NEG | NEG | NEG | NEG | POS | POS | NEG | NEG | NEG | POS | NEG | NEG | POS |

| STRAIN / ISOLATE | Clostridium difficile binary toxin component A | | | | Clostridium difficile binary toxin component B | | | | | | channel-forming hemolysin | holin-like pore-forming protein | putative negative regulator of pathogenicity locus | cellwall protein 66 | | | | cellwall protein 84 | septum formation initiation protein | | | flagellin subunit C | | | | |
|---|---|---|---|---|---|---|---|---|---|---|---|---|---|---|---|---|---|---|---|---|---|---|---|---|---|---|
| | hp1023\|cdtA | hp1026\|cdtA | hp1027\|cdtA | hp1029\|cdtA | hp1030\|cdtB | hp1031\|cdtB | hp1038\|cdtB | hp1039\|cdtB | hp1040\|cdtB | hp1041\|cdtB | hp1103\|hly3 | hp1113\|tcdE | hp1117\|tcdC | hp1012\|cwp66 | hp1016\|cwp66 | hp1020\|cwp66 | hp1021\|cwp66 | hp1018\|cwp84 | hp1044\|divIC | hp1045\|divIC | hp1046\|divIC | hp1141\|fliC | hp1142\|fliC | hp1144\|fliC | hp1145\|fliC | hp1147\|fliC |
| **>HP-07** | POS | POS | NEG | NEG | NEG | NEG | POS | POS | NEG | NEG | POS | POS | POS | NEG | NEG | NEG | NEG | POS | POS | NEG | NEG | NEG | POS | NEG | NEG | POS |
| Freiburg-923-44.923-DBK0000238 | POS | POS | NEG | NEG | NEG | NEG | POS | POS | NEG | NEG | POS | POS | POS | NEG | NEG | NEG | NEG | POS | POS | NEG | NEG | NEG | POS | NEG | NEG | POS |
| | AMB | POS | NEG | NEG | NEG | NEG | POS | POS | NEG | NEG | POS | POS | POS | NEG | NEG | NEG | NEG | POS | POS | NEG | NEG | NEG | POS | NEG | NEG | POS |
| **>HP-08** | AMB | POS | NEG | NEG | NEG | NEG | POS | POS | NEG | NEG | POS | POS | POS | NEG | NEG | NEG | NEG | POS | POS | NEG | NEG | NEG | POS | NEG | NEG | POS |
| Freiburg-1021-71.1021-DBK0000248 | POS | POS | NEG | NEG | NEG | NEG | POS | POS | NEG | NEG | POS | POS | POS | NEG | NEG | NEG | NEG | POS | POS | NEG | NEG | NEG | POS | NEG | NEG | POS |
| | NEG | AMB | NEG | NEG | NEG | NEG | POS | POS | NEG | NEG | POS | POS | POS | NEG | NEG | NEG | NEG | POS | POS | NEG | NEG | NEG | POS | NEG | NEG | POS |
| Freiburg-669-9.669-DBK0000231 | AMB | AMB | NEG | NEG | NEG | NEG | POS | POS | NEG | NEG | POS | POS | POS | NEG | NEG | NEG | NEG | POS | POS | NEG | NEG | NEG | POS | NEG | NEG | POS |
| | POS | POS | NEG | NEG | NEG | NEG | POS | POS | NEG | NEG | POS | POS | POS | NEG | NEG | NEG | NEG | POS | POS | NEG | NEG | NEG | POS | NEG | NEG | POS |
| Freiburg-964-53.964-DBK0000267 | AMB | POS | NEG | NEG | NEG | NEG | POS | POS | NEG | NEG | POS | POS | POS | NEG | NEG | NEG | NEG | POS | POS | NEG | NEG | NEG | POS | NEG | NEG | POS |
| | NEG | POS | NEG | NEG | NEG | NEG | POS | POS | NEG | NEG | POS | POS | POS | NEG | NEG | NEG | NEG | POS | POS | NEG | NEG | NEG | POS | NEG | NEG | POS |
| Freiburg-899-41.899-DBK0000224 | POS | POS | NEG | NEG | NEG | NEG | POS | POS | NEG | NEG | POS | POS | POS | NEG | NEG | NEG | NEG | POS | POS | NEG | AMB | NEG | POS | NEG | NEG | POS |
| | NEG | NEG | NEG | NEG | NEG | NEG | NEG | POS | NEG | NEG | AMB | AMB | AMB | NEG | NEG | NEG | NEG | POS | NEG | NEG | NEG | NEG | NEG | NEG | NEG | NEG |
| Freiburg-982-58.982-DBK0000257 | AMB | POS | NEG | NEG | NEG | NEG | POS | POS | NEG | NEG | POS | POS | POS | NEG | NEG | NEG | NEG | POS | POS | NEG | NEG | NEG | POS | NEG | NEG | POS |
| | NEG | NEG | NEG | NEG | NEG | NEG | NEG | POS | NEG | NEG | POS | POS | NEG | NEG | NEG | NEG | NEG | AMB | NEG | NEG | NEG | NEG | NEG | NEG | NEG | AMB |
| Dresden 07_7035 | NEG | AMB | NEG | NEG | NEG | NEG | POS | POS | NEG | NEG | POS | POS | POS | NEG | NEG | NEG | NEG | POS | AMB | NEG | NEG | NEG | POS | NEG | NEG | POS |
| Dresden 07_7248 | POS | POS | NEG | NEG | NEG | NEG | POS | POS | NEG | NEG | POS | POS | POS | NEG | NEG | NEG | NEG | POS | POS | NEG | AMB | NEG | POS | NEG | NEG | POS |
| Dresden 07_8702 | NEG | AMB | NEG | NEG | NEG | NEG | POS | POS | NEG | NEG | POS | POS | POS | NEG | NEG | NEG | NEG | POS | AMB | NEG | NEG | NEG | POS | NEG | NEG | POS |
| Dresden 07_8989 | NEG | AMB | NEG | NEG | NEG | NEG | POS | POS | NEG | NEG | POS | POS | POS | NEG | NEG | NEG | NEG | POS | AMB | NEG | NEG | NEG | POS | NEG | NEG | POS |
| Dresden 08_0712 | POS | POS | NEG | NEG | NEG | NEG | POS | POS | NEG | NEG | POS | POS | POS | NEG | NEG | NEG | NEG | POS | POS | NEG | NEG | NEG | POS | NEG | NEG | POS |
| **>HP-09** | AMB | POS | NEG | NEG | NEG | NEG | POS | POS | NEG | NEG | POS | POS | POS | NEG | NEG | NEG | NEG | NEG | POS | NEG | NEG | NEG | POS | NEG | NEG | POS |
| Freiburg-1056-79.1056-DBK0000261 | AMB | POS | NEG | NEG | NEG | NEG | POS | POS | NEG | NEG | POS | POS | POS | NEG | NEG | NEG | NEG | NEG | POS | NEG | NEG | NEG | POS | NEG | NEG | POS |
| Freiburg-658-8.658-DBK0000230 | POS | POS | NEG | NEG | NEG | NEG | POS | POS | NEG | NEG | POS | POS | POS | NEG | NEG | NEG | NEG | NEG | POS | NEG | AMB | NEG | POS | NEG | NEG | POS |
| | NEG | AMB | NEG | NEG | NEG | NEG | POS | POS | NEG | NEG | POS | POS | POS | NEG | NEG | NEG | NEG | NEG | POS | NEG | NEG | NEG | POS | NEG | NEG | POS |
| Freiburg-963-52.963-DBK0000265 | AMB | POS | NEG | NEG | NEG | NEG | POS | POS | NEG | NEG | POS | POS | POS | NEG | NEG | NEG | NEG | NEG | POS | NEG | NEG | NEG | POS | NEG | NEG | POS |
| | NEG | POS | NEG | NEG | NEG | NEG | POS | POS | NEG | NEG | POS | POS | POS | NEG | NEG | NEG | NEG | NEG | AMB | NEG | NEG | NEG | AMB | NEG | NEG | POS |
| Freiburg-988-63.988-DBK0000253 | AMB | POS | NEG | NEG | NEG | NEG | POS | POS | NEG | NEG | POS | POS | POS | NEG | NEG | NEG | NEG | NEG | POS | NEG | NEG | NEG | POS | NEG | NEG | POS |
| | NEG | AMB | NEG | NEG | NEG | NEG | POS | POS | NEG | NEG | POS | POS | AMB | NEG | NEG | NEG | NEG | NEG | AMB | NEG | NEG | NEG | AMB | NEG | NEG | POS |
| Freiburg_79.1057 | NEG | POS | NEG | NEG | NEG | NEG | POS | POS | NEG | NEG | POS | POS | POS | NEG | NEG | NEG | NEG | NEG | POS | NEG | NEG | NEG | POS | NEG | NEG | POS |
| Jena_S0017.11 | NEG | POS | NEG | NEG | NEG | NEG | POS | POS | NEG | NEG | POS | POS | POS | NEG | NEG | NEG | NEG | NEG | AMB | NEG | NEG | NEG | AMB | NEG | NEG | POS |
| | NEG | POS | NEG | NEG | NEG | NEG | POS | POS | NEG | NEG | POS | POS | POS | NEG | NEG | NEG | NEG | NEG | POS | NEG | NEG | NEG | POS | NEG | NEG | POS |
| | NEG | AMB | NEG | NEG | NEG | NEG | POS | POS | NEG | NEG | POS | POS | POS | NEG | NEG | NEG | NEG | NEG | AMB | NEG | NEG | NEG | AMB | NEG | NEG | POS |
| | NEG | POS | NEG | NEG | NEG | NEG | POS | POS | NEG | NEG | POS | POS | POS | NEG | NEG | NEG | NEG | NEG | POS | NEG | NEG | NEG | POS | NEG | NEG | POS |
| | NEG | POS | NEG | NEG | NEG | NEG | AMB | POS | NEG | NEG | POS | POS | POS | NEG | NEG | NEG | NEG | NEG | AMB | NEG | NEG | NEG | AMB | NEG | NEG | POS |
| Jena_S0015_11 | NEG | AMB | NEG | NEG | NEG | NEG | POS | POS | NEG | NEG | POS | POS | AMB | NEG | NEG | NEG | NEG | NEG | AMB | NEG | NEG | NEG | AMB | NEG | NEG | POS |
| | NEG | POS | NEG | NEG | NEG | NEG | POS | POS | NEG | NEG | POS | POS | POS | NEG | NEG | NEG | NEG | NEG | POS | NEG | NEG | NEG | POS | NEG | NEG | POS |
| | NEG | POS | NEG | NEG | NEG | NEG | POS | POS | NEG | NEG | POS | POS | POS | NEG | NEG | NEG | NEG | NEG | POS | NEG | NEG | NEG | POS | NEG | NEG | POS |
| Jena_S0012_11 | NEG | POS | NEG | NEG | NEG | NEG | POS | POS | NEG | NEG | POS | POS | POS | NEG | NEG | NEG | NEG | NEG | NEG | NEG | NEG | NEG | AMB | NEG | NEG | POS |
| | NEG | AMB | NEG | NEG | NEG | NEG | POS | POS | NEG | NEG | POS | POS | AMB | NEG | NEG | NEG | NEG | NEG | NEG | NEG | NEG | NEG | AMB | NEG | NEG | POS |
| Dresden 07_7363 | POS | POS | NEG | NEG | NEG | NEG | POS | POS | NEG | NEG | NEG | POS | POS | NEG | NEG | NEG | NEG | NEG | POS | NEG | NEG | NEG | POS | NEG | NEG | POS |
| Dresden 07_8174 | NEG | NEG | NEG | NEG | NEG | NEG | AMB | POS | NEG | NEG | POS | POS | AMB | NEG | NEG | NEG | NEG | NEG | NEG | NEG | NEG | NEG | AMB | NEG | NEG | AMB |
| Dresden 07_8224 | NEG | NEG | NEG | NEG | NEG | NEG | AMB | POS | NEG | NEG | POS | POS | AMB | NEG | NEG | NEG | NEG | NEG | NEG | NEG | NEG | NEG | NEG | NEG | NEG | AMB |
| Dresden 07_8562 | NEG | AMB | NEG | NEG | NEG | NEG | POS | POS | NEG | NEG | POS | POS | POS | NEG | NEG | NEG | NEG | NEG | AMB | NEG | NEG | NEG | POS | NEG | NEG | POS |
| Dresden 08_613 | NEG | POS | NEG | NEG | NEG | NEG | NEG | AMB | NEG | NEG | POS | POS | POS | NEG | NEG | NEG | NEG | NEG | AMB | NEG | NEG | NEG | AMB | NEG | NEG | POS |
| Dresden 08_0422 | NEG | AMB | NEG | NEG | NEG | NEG | POS | POS | NEG | NEG | POS | POS | POS | NEG | NEG | NEG | NEG | NEG | POS | NEG | NEG | NEG | POS | NEG | NEG | POS |
| Dresden 08_0837 | NEG | AMB | NEG | NEG | NEG | NEG | POS | POS | NEG | NEG | POS | POS | POS | NEG | NEG | NEG | NEG | NEG | POS | NEG | NEG | NEG | POS | NEG | NEG | POS |
| Dresden 08_0878 | NEG | NEG | NEG | NEG | NEG | NEG | NEG | POS | NEG | NEG | POS | POS | NEG | NEG | NEG | NEG | NEG | NEG | NEG | NEG | NEG | NEG | NEG | NEG | NEG | AMB |
| Dresden 08_1484 | POS | POS | NEG | NEG | NEG | NEG | POS | POS | NEG | NEG | POS | POS | POS | NEG | NEG | NEG | NEG | NEG | POS | NEG | NEG | NEG | POS | NEG | NEG | POS |
| **>HP-10** | AMB | POS | NEG | NEG | NEG | NEG | POS | POS | NEG | NEG | POS | POS | POS | NEG | NEG | POS | NEG | POS | AMB | NEG | NEG | NEG | AMB | NEG | NEG | POS |
| Dresden 09_664_1 | NEG | AMB | NEG | NEG | NEG | NEG | POS | POS | NEG | NEG | POS | POS | POS | NEG | NEG | AMB | NEG | POS | AMB | NEG | NEG | NEG | AMB | NEG | NEG | POS |
| Dresden 09_664_2 | NEG | NEG | NEG | NEG | NEG | NEG | AMB | POS | NEG | NEG | POS | POS | AMB | NEG | NEG | AMB | NEG | POS | AMB | NEG | NEG | NEG | AMB | NEG | NEG | POS |
| Dresden 09_711_1 | NEG | AMB | NEG | NEG | NEG | NEG | AMB | POS | NEG | NEG | POS | POS | POS | NEG | NEG | NEG | NEG | POS | NEG | NEG | NEG | NEG | NEG | NEG | NEG | POS |
| Dresden 09_711_2 | NEG | NEG | NEG | NEG | NEG | NEG | NEG | POS | NEG | NEG | POS | AMB | AMB | NEG | NEG | AMB | NEG | AMB | NEG | NEG | NEG | NEG | NEG | NEG | NEG | AMB |
| Dresden 09_932 | AMB | POS | NEG | NEG | NEG | NEG | POS | POS | NEG | NEG | POS | POS | POS | NEG | NEG | POS | NEG | POS | POS | NEG | NEG | NEG | POS | NEG | NEG | POS |
| Dresden 09_939 | NEG | POS | NEG | NEG | NEG | NEG | POS | POS | NEG | NEG | POS | POS | POS | NEG | NEG | POS | NEG | POS | POS | NEG | NEG | NEG | POS | NEG | NEG | POS |
| Dresden 09_948 | AMB | AMB | NEG | NEG | NEG | NEG | POS | POS | NEG | NEG | POS | POS | POS | NEG | NEG | POS | NEG | POS | POS | NEG | NEG | NEG | POS | NEG | NEG | POS |
| **>HP-11** | NEG | NEG | NEG | NEG | NEG | NEG | NEG | AMB | NEG | NEG | POS | NEG | NEG | NEG | NEG | POS | NEG | POS | POS | NEG | AMB | POS | NEG | POS | NEG | NEG |
| C. difficile CD37 (GenBank AHJJ) in silico prediction | NEG | NEG | NEG | NEG | NEG | NEG | NEG | NEG | NEG | NEG | POS | NEG | NEG | NEG | NEG | POS | NEG | POS | POS | NEG | POS | POS | NEG | POS | NEG | NEG |
| Freiburg-684-10.684-DBK0000240 | NEG | NEG | NEG | NEG | NEG | NEG | NEG | AMB | NEG | NEG | POS | NEG | NEG | NEG | NEG | POS | NEG | POS | POS | NEG | AMB | POS | NEG | POS | NEG | NEG |
| Freiburg-838-28.838-DBK0000278 | NEG | NEG | NEG | NEG | NEG | NEG | NEG | POS | NEG | NEG | POS | NEG | NEG | NEG | NEG | POS | NEG | POS | POS | NEG | NEG | POS | NEG | POS | NEG | NEG |
| | NEG | NEG | NEG | NEG | NEG | NEG | NEG | NEG | NEG | NEG | POS | NEG | NEG | NEG | NEG | NEG | NEG | POS | AMB | NEG | NEG | POS | NEG | POS | NEG | NEG |
| **>HP-12** | POS | POS | NEG | NEG | NEG | NEG | POS | POS | NEG | NEG | POS | POS | POS | NEG | NEG | NEG | NEG | POS | AMB | NEG | NEG | NEG | AMB | NEG | NEG | POS |
| Freiburg-1007-68.1007-DBK0000252 | POS | POS | NEG | NEG | NEG | NEG | POS | POS | NEG | NEG | POS | POS | POS | NEG | NEG | NEG | NEG | POS | POS | NEG | NEG | NEG | POS | NEG | NEG | POS |
| | NEG | NEG | NEG | NEG | NEG | NEG | NEG | POS | NEG | NEG | POS | POS | NEG | NEG | NEG | NEG | NEG | POS | NEG | NEG | NEG | NEG | NEG | NEG | NEG | POS |
| Freiburg-983-59.983-DBK0000256 | AMB | POS | NEG | NEG | NEG | NEG | POS | POS | NEG | NEG | POS | POS | POS | NEG | NEG | NEG | NEG | POS | POS | NEG | NEG | NEG | POS | NEG | NEG | POS |
| | NEG | AMB | NEG | NEG | NEG | NEG | POS | POS | NEG | NEG | POS | POS | AMB | NEG | NEG | NEG | NEG | POS | NEG | NEG | NEG | NEG | NEG | NEG | NEG | POS |
| **>HP-13** | POS | POS | NEG | NEG | NEG | NEG | POS | POS | NEG | NEG | POS | POS | POS | NEG | NEG | NEG | NEG | POS | POS | NEG | NEG | NEG | POS | NEG | NEG | POS |
| C. difficile 70-100-2010 (GenBank AGAC) in silico prediction | POS | POS | NEG | NEG | NEG | NEG | POS | POS | NEG | NEG | POS | POS | POS | NEG | NEG | NEG | NEG | POS | POS | NEG | POS | NEG | POS | NEG | NEG | POS |
| Freiburg-1044-75.1044-DBK0000270 | AMB | POS | NEG | NEG | NEG | NEG | POS | POS | NEG | NEG | POS | POS | POS | NEG | NEG | NEG | NEG | POS | POS | NEG | NEG | NEG | POS | NEG | NEG | POS |
| | NEG | POS | NEG | NEG | NEG | NEG | POS | POS | NEG | NEG | POS | POS | POS | NEG | NEG | NEG | NEG | POS | POS | NEG | NEG | NEG | POS | NEG | NEG | POS |
| Freiburg-1057-80.1057-DBK0000260 | POS | POS | NEG | NEG | NEG | NEG | POS | POS | NEG | NEG | POS | POS | POS | NEG | NEG | NEG | NEG | POS | POS | NEG | AMB | NEG | POS | NEG | NEG | POS |
| | NEG | AMB | NEG | NEG | NEG | NEG | POS | POS | NEG | NEG | POS | POS | AMB | NEG | NEG | NEG | NEG | POS | AMB | NEG | NEG | NEG | POS | NEG | NEG | POS |
| Freiburg-710-12.710-DBK0000241 | AMB | POS | NEG | NEG | NEG | NEG | POS | POS | NEG | NEG | POS | POS | POS | NEG | NEG | NEG | NEG | POS | POS | NEG | NEG | NEG | AMB | NEG | NEG | POS |
| | AMB | POS | NEG | NEG | NEG | NEG | POS | POS | NEG | NEG | POS | POS | POS | NEG | NEG | NEG | NEG | POS | POS | NEG | NEG | NEG | POS | NEG | NEG | POS |
| Freiburg-940-48.94-DBK0000244 | POS | POS | NEG | NEG | NEG | NEG | POS | POS | NEG | NEG | POS | POS | POS | NEG | NEG | NEG | NEG | POS | POS | NEG | NEG | NEG | POS | NEG | NEG | POS |
| | POS | POS | NEG | NEG | NEG | NEG | POS | POS | NEG | NEG | POS | POS | POS | NEG | NEG | NEG | NEG | POS | POS | NEG | NEG | NEG | POS | NEG | NEG | POS |
| Freiburg-980-56.98-DBK0000246 | AMB | POS | NEG | NEG | NEG | NEG | POS | POS | NEG | NEG | POS | POS | POS | NEG | NEG | NEG | NEG | POS | POS | NEG | NEG | NEG | POS | NEG | NEG | POS |
| | NEG | AMB | NEG | NEG | NEG | NEG | POS | POS | NEG | NEG | POS | POS | POS | NEG | NEG | NEG | NEG | POS | AMB | NEG | NEG | NEG | POS | NEG | NEG | POS |
| Jena_S0014_11 | NEG | POS | NEG | NEG | NEG | NEG | POS | POS | NEG | NEG | POS | POS | POS | NEG | NEG | NEG | NEG | POS | AMB | NEG | NEG | NEG | AMB | NEG | NEG | POS |
| | NEG | POS | NEG | NEG | NEG | NEG | POS | POS | NEG | NEG | POS | POS | POS | NEG | NEG | NEG | NEG | POS | POS | NEG | NEG | NEG | POS | NEG | NEG | POS |
| | NEG | AMB | NEG | NEG | NEG | NEG | POS | POS | NEG | NEG | POS | POS | AMB | NEG | NEG | NEG | NEG | POS | AMB | NEG | NEG | NEG | AMB | NEG | NEG | POS |
| Jena_S0023_11 | NEG | POS | NEG | NEG | NEG | NEG | POS | POS | NEG | NEG | POS | POS | POS | NEG | NEG | NEG | NEG | POS | AMB | NEG | NEG | NEG | AMB | NEG | NEG | POS |
| | NEG | POS | NEG | NEG | NEG | NEG | POS | POS | NEG | NEG | POS | POS | POS | NEG | NEG | NEG | NEG | POS | AMB | NEG | NEG | NEG | AMB | NEG | NEG | POS |
| | NEG | NEG | NEG | NEG | NEG | NEG | NEG | POS | NEG | NEG | POS | POS | AMB | NEG | NEG | NEG | NEG | POS | NEG | NEG | NEG | NEG | NEG | NEG | NEG | AMB |
| Dresden 07_7779 | NEG | NEG | NEG | NEG | NEG | NEG | NEG | AMB | NEG | NEG | NEG | NEG | NEG | NEG | NEG | NEG | NEG | NEG | NEG | NEG | NEG | NEG | NEG | NEG | NEG | NEG |
| Dresden 07_7950 | POS | POS | NEG | NEG | NEG | NEG | POS | POS | NEG | NEG | POS | POS | POS | NEG | NEG | NEG | NEG | POS | POS | NEG | NEG | NEG | POS | NEG | NEG | POS |
| Dresden 07_7953 | AMB | AMB | NEG | NEG | NEG | NEG | POS | POS | NEG | NEG | POS | POS | POS | NEG | NEG | NEG | NEG | POS | AMB | NEG | NEG | NEG | POS | NEG | NEG | POS |
| Dresden 07_8015 | NEG | AMB | NEG | NEG | NEG | NEG | POS | POS | NEG | NEG | POS | POS | POS | NEG | NEG | NEG | NEG | POS | AMB | NEG | NEG | NEG | AMB | NEG | NEG | POS |
| Dresden 07_8205 | NEG | NEG | NEG | NEG | NEG | NEG | AMB | POS | NEG | NEG | POS | POS | AMB | NEG | NEG | NEG | NEG | POS | NEG | NEG | NEG | NEG | AMB | NEG | NEG | POS |
| Dresden 07_8595 | AMB | POS | NEG | NEG | NEG | NEG | POS | POS | NEG | NEG | POS | POS | POS | NEG | NEG | NEG | NEG | POS | POS | NEG | NEG | NEG | POS | NEG | NEG | POS |
| Dresden 07_8613 | NEG | NEG | NEG | NEG | NEG | NEG | NEG | POS | NEG | NEG | POS | AMB | AMB | NEG | NEG | NEG | NEG | AMB | NEG | NEG | NEG | NEG | NEG | NEG | NEG | NEG |
| Dresden 07_8686 | NEG | NEG | NEG | NEG | NEG | NEG | AMB | POS | NEG | NEG | POS | POS | POS | NEG | NEG | NEG | NEG | POS | AMB | NEG | NEG | NEG | AMB | NEG | NEG | POS |
| Dresden 07_9180 | AMB | POS | NEG | NEG | NEG | NEG | POS | POS | NEG | NEG | POS | POS | POS | NEG | NEG | NEG | NEG | POS | POS | NEG | NEG | NEG | POS | NEG | NEG | POS |
| Dresden 08_0237 | NEG | NEG | NEG | NEG | NEG | NEG | AMB | POS | NEG | NEG | POS | POS | POS | NEG | NEG | NEG | NEG | POS | NEG | NEG | NEG | NEG | AMB | NEG | NEG | POS |
| Dresden 08_0492 | NEG | AMB | NEG | NEG | NEG | NEG | POS | POS | NEG | NEG | POS | POS | AMB | NEG | NEG | NEG | NEG | POS | AMB | NEG | NEG | NEG | AMB | NEG | NEG | POS |
| Dresden 08_0992 | NEG | AMB | NEG | NEG | NEG | NEG | POS | POS | NEG | NEG | POS | POS | AMB | NEG | NEG | NEG | NEG | POS | AMB | NEG | NEG | NEG | AMB | NEG | NEG | POS |
| Dresden 08_1184 | POS | POS | NEG | NEG | NEG | NEG | POS | POS | NEG | NEG | POS | POS | POS | NEG | NEG | NEG | NEG | POS | POS | NEG | NEG | NEG | POS | NEG | NEG | POS |
| Dresden 08_1314 | AMB | AMB | NEG | NEG | NEG | NEG | POS | POS | NEG | NEG | POS | POS | POS | NEG | NEG | NEG | NEG | POS | AMB | NEG | NEG | NEG | POS | NEG | NEG | POS |
| Dresden 08_1640 | NEG | AMB | NEG | NEG | NEG | NEG | POS | POS | NEG | NEG | POS | POS | AMB | NEG | NEG | NEG | NEG | POS | AMB | NEG | NEG | NEG | NEG | NEG | NEG | POS |
| Dresden 08_1640 | NEG | AMB | NEG | NEG | NEG | NEG | POS | POS | NEG | NEG | POS | POS | AMB | NEG | NEG | NEG | NEG | POS | AMB | NEG | NEG | NEG | NEG | NEG | NEG | POS |
| Dresden 08_1758 | NEG | AMB | NEG | NEG | NEG | NEG | AMB | POS | NEG | NEG | POS | POS | POS | NEG | NEG | NEG | NEG | POS | AMB | NEG | NEG | NEG | AMB | NEG | NEG | POS |
| **>HP-14** | POS | POS | NEG | NEG | NEG | NEG | POS | POS | NEG | NEG | POS | POS | POS | NEG | NEG | NEG | NEG | POS | POS | NEG | NEG | NEG | POS | NEG | NEG | POS |
| Freiburg-1019-70.1019-DBK0000255 | AMB | POS | NEG | NEG | NEG | NEG | POS | POS | NEG | NEG | POS | POS | POS | NEG | NEG | NEG | NEG | POS | POS | NEG | NEG | NEG | POS | NEG | NEG | POS |
| **>HP-15** | NEG | NEG | NEG | NEG | NEG | NEG | NEG | NEG | NEG | NEG | POS | NEG | NEG | NEG | NEG | NEG | NEG | POS | POS | NEG | NEG | NEG | POS | NEG | NEG | POS |
| Freiburg_42.907 | NEG | NEG | NEG | NEG | NEG | NEG | NEG | NEG | NEG | NEG | POS | NEG | NEG | NEG | NEG | NEG | NEG | POS | POS | NEG | NEG | NEG | POS | NEG | NEG | POS |
| **>HP-16** | POS | POS | NEG | NEG | NEG | NEG | POS | POS | NEG | NEG | POS | POS | POS | NEG | NEG | NEG | POS | POS | AMB | NEG | NEG | NEG | AMB | NEG | NEG | POS |
| Freiburg-978-55.978-DBK0000259 | AMB | POS | NEG | NEG | NEG | NEG | POS | POS | NEG | NEG | POS | POS | POS | NEG | NEG | NEG | POS | POS | POS | NEG | NEG | NEG | POS | NEG | NEG | POS |
| | NEG | AMB | NEG | NEG | NEG | NEG | POS | POS | NEG | NEG | POS | POS | AMB | NEG | NEG | NEG | POS | POS | AMB | NEG | NEG | NEG | AMB | NEG | NEG | POS |
| Dresden 08_1810 | NEG | NEG | NEG | NEG | NEG | NEG | NEG | POS | NEG | NEG | POS | AMB | NEG | NEG | NEG | NEG | AMB | AMB | NEG | NEG | NEG | NEG | NEG | NEG | NEG | AMB |
| **>HP-17** | AMB | AMB | NEG | NEG | NEG | NEG | POS | POS | NEG | NEG | POS | POS | POS | NEG | NEG | NEG | NEG | POS | AMB | NEG | NEG | NEG | AMB | NEG | NEG | POS |
| Freiburg-729-15.729-DBK0000226 | NEG | NEG | NEG | NEG | NEG | NEG | NEG | POS | NEG | NEG | POS | POS | NEG | NEG | NEG | NEG | NEG | AMB | NEG | NEG | NEG | NEG | NEG | NEG | NEG | AMB |
| | NEG | NEG | NEG | NEG | NEG | NEG | AMB | POS | NEG | NEG | AMB | NEG | AMB | NEG | NEG | NEG | POS | POS | NEG | NEG | NEG | NEG | NEG | NEG | NEG | AMB |
| Freiburg-926-45.926-DBK0000236 | POS | POS | NEG | NEG | NEG | NEG | POS | POS | NEG | NEG | POS | POS | POS | NEG | NEG | NEG | NEG | POS | POS | NEG | NEG | NEG | POS | NEG | NEG | POS |
| | POS | POS | NEG | NEG | NEG | NEG | POS | POS | NEG | NEG | POS | POS | POS | NEG | NEG | NEG | NEG | POS | POS | NEG | NEG | NEG | POS | NEG | NEG | POS |
| **>HP-18** | NEG | POS | NEG | NEG | NEG | NEG | POS | POS | NEG | NEG | POS | POS | POS | NEG | NEG | NEG | NEG | POS | AMB | NEG | NEG | NEG | AMB | NEG | NEG | POS |
| Freiburg-651-7.651-DBK0000229 | NEG | POS | NEG | NEG | NEG | NEG | POS | POS | NEG | NEG | POS | POS | POS | NEG | NEG | NEG | NEG | POS | POS | NEG | AMB | NEG | POS | NEG | NEG | POS |
| | NEG | POS | NEG | NEG | NEG | NEG | POS | POS | NEG | NEG | POS | POS | POS | NEG | NEG | NEG | NEG | POS | POS | NEG | NEG | NEG | POS | NEG | NEG | POS |
| Freiburg-725-13.725-DBK0000227 | NEG | NEG | NEG | NEG | NEG | NEG | AMB | POS | NEG | NEG | POS | POS | AMB | NEG | NEG | NEG | NEG | POS | NEG | NEG | NEG | NEG | NEG | NEG | NEG | POS |
| | NEG | NEG | NEG | NEG | NEG | NEG | AMB | POS | NEG | NEG | POS | POS | AMB | NEG | NEG | NEG | NEG | POS | NEG | NEG | NEG | NEG | AMB | NEG | NEG | AMB |
| Dresden 07_7176 | NEG | NEG | NEG | NEG | NEG | NEG | NEG | POS | NEG | NEG | AMB | NEG | NEG | NEG | NEG | NEG | NEG | NEG | NEG | NEG | NEG | NEG | NEG | NEG | NEG | NEG |
| Dresden 07_7209 | NEG | POS | NEG | NEG | NEG | NEG | POS | POS | NEG | NEG | POS | POS | POS | NEG | NEG | NEG | NEG | POS | POS | NEG | NEG | NEG | POS | NEG | NEG | POS |
| Dresden 07_8029 | NEG | AMB | NEG | NEG | NEG | NEG | POS | POS | NEG | NEG | POS | POS | POS | NEG | NEG | NEG | NEG | POS | AMB | NEG | NEG | NEG | AMB | NEG | NEG | POS |
| Dresden 07_8575 | NEG | AMB | NEG | NEG | NEG | NEG | AMB | POS | NEG | NEG | POS | POS | POS | NEG | NEG | NEG | NEG | POS | AMB | NEG | NEG | NEG | AMB | NEG | NEG | POS |
| Dresden 08_1746 | NEG | AMB | NEG | NEG | NEG | NEG | POS | POS | NEG | NEG | POS | POS | AMB | NEG | NEG | NEG | NEG | POS | AMB | NEG | NEG | NEG | AMB | NEG | NEG | POS |
| **>HP-19** | NEG | NEG | NEG | NEG | NEG | NEG | NEG | NEG | NEG | NEG | POS | POS | POS | NEG | NEG | NEG | NEG | POS | POS | NEG | NEG | NEG | POS | NEG | NEG | POS |
| Freiburg-894-37.894-DBK0000289 | NEG | NEG | NEG | NEG | NEG | NEG | NEG | NEG | NEG | NEG | POS | POS | POS | NEG | NEG | NEG | NEG | POS | POS | NEG | NEG | NEG | POS | NEG | NEG | POS |
| | NEG | NEG | NEG | NEG | NEG | NEG | NEG | NEG | NEG | NEG | POS | POS | POS | NEG | NEG | NEG | NEG | POS | AMB | NEG | NEG | NEG | AMB | NEG | NEG | POS |
| Freiburg-1000-67.1-DBK0000263 | NEG | NEG | NEG | NEG | NEG | NEG | NEG | NEG | NEG | NEG | POS | POS | POS | NEG | NEG | NEG | NEG | POS | POS | NEG | AMB | NEG | POS | NEG | NEG | POS |
| | NEG | NEG | NEG | NEG | NEG | NEG | NEG | NEG | NEG | NEG | POS | POS | POS | NEG | NEG | NEG | NEG | POS | POS | NEG | NEG | NEG | POS | NEG | NEG | POS |
| Dresden 07_7828 | NEG | NEG | NEG | NEG | NEG | NEG | NEG | NEG | NEG | NEG | POS | AMB | POS | NEG | NEG | NEG | NEG | AMB | NEG | NEG | NEG | NEG | NEG | NEG | NEG | POS |
| Dresden 08_1128 | NEG | NEG | NEG | NEG | NEG | NEG | NEG | AMB | NEG | NEG | POS | POS | AMB | NEG | NEG | NEG | NEG | POS | AMB | NEG | NEG | NEG | AMB | NEG | NEG | POS |

| STRAIN / ISOLATE | Clostridium difficile binary toxin component A | | | | Clostridium difficile binary toxin component B | | | | | | channel-forming hemolysin | holin-like pore-forming protein | putative negative regulator of pathogenicity locus | cellwall protein 66 | | | | cellwall protein 84 | septum formation initiation protein | | | flagellin subunit C | | | | |
|---|---|---|---|---|---|---|---|---|---|---|---|---|---|---|---|---|---|---|---|---|---|---|---|---|---|---|
| | hp1023\|cdtA | hp1026\|cdtA | hp1027\|cdtA | hp1029\|cdtA | hp1030\|cdtB | hp1031\|cdtB | hp1038\|cdtB | hp1039\|cdtB | hp1040\|cdtB | hp1041\|cdtB | hp1103\|hly3 | hp1113\|tcdE | hp1117\|tcdC | hp1012\|cwp66 | hp1016\|cwp66 | hp1020\|cwp66 | hp1021\|cwp66 | hp1018\|cwp84 | hp1044\|divIC | hp1045\|divIC | hp1046\|divIC | hp1141\|fliC | hp1142\|fliC | hp1144\|fliC | hp1145\|fliC | hp1147\|fliC |
| **>HP-20** | AMB | POS | NEG | NEG | NEG | NEG | POS | POS | NEG | NEG | POS | POS | POS | NEG | NEG | NEG | NEG | POS | POS | NEG | NEG | NEG | POS | NEG | NEG | POS |
| Freiburg-963-50.963-DBK0000243 | AMB | POS | NEG | NEG | NEG | NEG | POS | POS | NEG | NEG | POS | POS | POS | NEG | NEG | NEG | NEG | POS | POS | NEG | NEG | NEG | POS | NEG | NEG | POS |
| Freiburg-750-18.75-DBK0000232 | AMB | POS | NEG | NEG | NEG | NEG | POS | POS | NEG | NEG | POS | POS | POS | NEG | NEG | NEG | NEG | POS | POS | NEG | NEG | NEG | POS | NEG | NEG | POS |
| | NEG | NEG | NEG | NEG | NEG | NEG | NEG | POS | NEG | NEG | POS | AMB | NEG | NEG | NEG | NEG | NEG | AMB | NEG | NEG | NEG | NEG | NEG | NEG | NEG | AMB |
| Freiburg-637-4.637-DBK0000280 | AMB | POS | NEG | NEG | NEG | NEG | POS | POS | NEG | NEG | POS | POS | POS | NEG | NEG | NEG | NEG | POS | POS | NEG | NEG | NEG | POS | NEG | NEG | POS |
| | NEG | AMB | NEG | NEG | NEG | NEG | POS | POS | NEG | NEG | POS | POS | POS | NEG | NEG | NEG | NEG | POS | AMB | NEG | NEG | NEG | AMB | NEG | NEG | POS |
| Freiburg-856-31.856-DBK0000273 | AMB | POS | NEG | NEG | NEG | NEG | POS | POS | NEG | NEG | POS | POS | POS | NEG | NEG | NEG | NEG | POS | POS | NEG | NEG | NEG | POS | NEG | NEG | POS |
| | AMB | POS | NEG | NEG | NEG | NEG | POS | POS | NEG | NEG | POS | POS | POS | NEG | NEG | NEG | NEG | POS | POS | NEG | NEG | NEG | POS | NEG | NEG | POS |
| Freiburg_51.956 | NEG | POS | NEG | NEG | NEG | NEG | POS | POS | NEG | NEG | POS | POS | POS | NEG | NEG | NEG | NEG | POS | POS | NEG | NEG | NEG | POS | NEG | NEG | POS |
| Dresden 07_7984 | NEG | NEG | NEG | NEG | NEG | NEG | AMB | POS | NEG | NEG | POS | POS | AMB | NEG | NEG | NEG | NEG | POS | AMB | NEG | NEG | NEG | AMB | NEG | NEG | AMB |
| Dresden 07_8203 | NEG | NEG | NEG | NEG | NEG | NEG | POS | POS | NEG | NEG | POS | POS | POS | NEG | NEG | NEG | NEG | POS | AMB | NEG | NEG | NEG | AMB | NEG | NEG | POS |
| Dresden 07_8398 | NEG | AMB | NEG | NEG | NEG | NEG | AMB | POS | NEG | NEG | POS | POS | POS | NEG | NEG | NEG | NEG | POS | NEG | NEG | NEG | NEG | AMB | NEG | NEG | AMB |
| Dresden 07_8798 | NEG | NEG | NEG | NEG | NEG | NEG | AMB | POS | NEG | NEG | POS | POS | POS | NEG | NEG | NEG | NEG | POS | NEG | NEG | NEG | NEG | AMB | NEG | NEG | POS |
| **>HP-21** | NEG | NEG | NEG | NEG | NEG | NEG | NEG | NEG | NEG | NEG | POS | NEG | NEG | NEG | NEG | NEG | NEG | POS | POS | NEG | NEG | NEG | POS | NEG | NEG | POS |
| Freiburg-823-26.823-DBK0000283 | NEG | NEG | NEG | NEG | NEG | NEG | NEG | AMB | NEG | NEG | POS | NEG | NEG | NEG | NEG | NEG | NEG | POS | POS | NEG | NEG | NEG | POS | NEG | NEG | POS |
| | NEG | NEG | NEG | NEG | NEG | NEG | NEG | NEG | NEG | NEG | POS | NEG | NEG | NEG | NEG | NEG | NEG | POS | POS | NEG | NEG | NEG | POS | NEG | NEG | POS |
| Freiburg-952-49.952-DBK0000245 | NEG | NEG | NEG | NEG | NEG | NEG | NEG | NEG | NEG | NEG | POS | NEG | NEG | NEG | NEG | NEG | NEG | POS | POS | NEG | NEG | NEG | POS | NEG | NEG | POS |
| | NEG | NEG | NEG | NEG | NEG | NEG | NEG | AMB | NEG | NEG | POS | NEG | NEG | NEG | NEG | NEG | NEG | POS | POS | NEG | NEG | NEG | POS | NEG | NEG | POS |
| **>HP-22** | AMB | POS | NEG | NEG | NEG | NEG | POS | POS | NEG | NEG | POS | POS | POS | NEG | NEG | NEG | NEG | POS | POS | NEG | NEG | POS | NEG | POS | NEG | NEG |
| Dresden 08_0288 | AMB | POS | NEG | NEG | NEG | NEG | POS | POS | NEG | NEG | POS | POS | POS | NEG | NEG | NEG | NEG | POS | POS | NEG | NEG | POS | NEG | POS | NEG | NEG |
| **>HP-23** | POS | POS | NEG | NEG | NEG | NEG | POS | POS | NEG | NEG | POS | POS | POS | NEG | NEG | NEG | NEG | POS | POS | NEG | NEG | POS | NEG | POS | NEG | NEG |
| Freiburg-786-21.786-DBK0000275 | AMB | POS | NEG | NEG | NEG | NEG | POS | POS | NEG | NEG | POS | POS | POS | NEG | NEG | NEG | NEG | POS | POS | NEG | NEG | POS | NEG | POS | NEG | NEG |
| | NEG | AMB | NEG | NEG | NEG | NEG | POS | POS | NEG | NEG | POS | POS | AMB | NEG | NEG | NEG | NEG | POS | AMB | NEG | NEG | POS | NEG | POS | NEG | NEG |
| **>HP-24** | AMB | POS | NEG | NEG | NEG | NEG | POS | POS | NEG | NEG | POS | POS | POS | NEG | POS | NEG | NEG | POS | AMB | NEG | NEG | POS | NEG | POS | NEG | NEG |
| C. difficile 6534 (GenBank ADEJ) in silico prediction | POS | POS | NEG | NEG | NEG | NEG | POS | POS | NEG | NEG | POS | POS | POS | NEG | POS | NEG | NEG | POS | POS | NEG | POS | POS | NEG | POS | NEG | NEG |
| C. difficile 630 (GenBank AM180355) in silico prediction | POS | POS | NEG | NEG | NEG | NEG | POS | POS | NEG | NEG | POS | POS | POS | NEG | POS | NEG | NEG | POS | POS | NEG | POS | POS | NEG | POS | NEG | NEG |
| C. difficile Strain 630 | AMB | AMB | NEG | NEG | NEG | NEG | POS | POS | NEG | NEG | POS | POS | POS | NEG | POS | NEG | NEG | POS | AMB | NEG | NEG | POS | NEG | POS | NEG | NEG |
| | AMB | POS | NEG | NEG | NEG | NEG | POS | POS | NEG | NEG | POS | POS | POS | NEG | POS | NEG | NEG | POS | POS | NEG | NEG | POS | NEG | POS | NEG | NEG |
| | AMB | POS | NEG | NEG | NEG | NEG | POS | POS | NEG | NEG | POS | POS | POS | NEG | POS | NEG | NEG | POS | POS | NEG | NEG | POS | NEG | POS | NEG | NEG |
| | NEG | AMB | NEG | NEG | NEG | NEG | AMB | POS | NEG | NEG | POS | POS | AMB | NEG | POS | NEG | NEG | POS | NEG | NEG | NEG | POS | NEG | POS | NEG | NEG |
| | AMB | POS | NEG | NEG | NEG | NEG | POS | POS | NEG | NEG | POS | POS | POS | NEG | POS | NEG | NEG | POS | POS | NEG | NEG | POS | NEG | POS | NEG | NEG |
| | NEG | NEG | NEG | NEG | NEG | NEG | NEG | POS | NEG | NEG | POS | POS | NEG | NEG | POS | NEG | NEG | POS | NEG | NEG | NEG | AMB | NEG | POS | NEG | NEG |
| Dresden 07_7156 | NEG | NEG | NEG | NEG | NEG | NEG | NEG | POS | NEG | NEG | AMB | AMB | NEG | NEG | AMB | NEG | NEG | AMB | NEG | NEG | NEG | NEG | NEG | POS | NEG | NEG |
| Dresden 07_7158 | NEG | AMB | NEG | NEG | NEG | NEG | POS | POS | NEG | NEG | POS | POS | POS | NEG | POS | NEG | NEG | POS | AMB | NEG | NEG | POS | NEG | POS | NEG | NEG |
| Dresden 07_7203 | AMB | POS | NEG | NEG | NEG | NEG | POS | POS | NEG | NEG | POS | POS | POS | NEG | POS | NEG | NEG | POS | AMB | NEG | NEG | POS | NEG | POS | NEG | NEG |
| Dresden 07_8217 | NEG | AMB | NEG | NEG | NEG | NEG | POS | POS | NEG | NEG | POS | POS | POS | NEG | POS | NEG | NEG | POS | AMB | NEG | NEG | POS | NEG | POS | NEG | NEG |
| Dresden 07_8270 | NEG | AMB | NEG | NEG | NEG | NEG | POS | POS | NEG | NEG | POS | POS | POS | NEG | POS | NEG | NEG | POS | AMB | NEG | NEG | POS | NEG | POS | NEG | NEG |
| Dresden 07_8749 | NEG | NEG | NEG | NEG | NEG | NEG | NEG | POS | NEG | NEG | AMB | AMB | NEG | NEG | AMB | NEG | NEG | AMB | NEG | NEG | NEG | NEG | NEG | POS | NEG | NEG |
| Dresden 07_8788 | NEG | AMB | NEG | NEG | NEG | NEG | POS | POS | NEG | NEG | POS | POS | POS | NEG | POS | NEG | NEG | POS | POS | NEG | NEG | POS | NEG | POS | NEG | NEG |
| Dresden 07_9245 | NEG | AMB | NEG | NEG | NEG | NEG | AMB | POS | NEG | NEG | POS | POS | POS | NEG | POS | NEG | NEG | POS | AMB | NEG | NEG | POS | NEG | POS | NEG | NEG |
| Dresden 08_0217 | NEG | AMB | NEG | NEG | NEG | NEG | POS | POS | NEG | NEG | POS | POS | POS | NEG | POS | NEG | NEG | POS | POS | NEG | NEG | POS | NEG | POS | NEG | NEG |
| Dresden 08_0219 | AMB | POS | NEG | NEG | NEG | NEG | POS | POS | NEG | NEG | POS | POS | POS | NEG | POS | NEG | NEG | POS | POS | NEG | NEG | POS | NEG | POS | NEG | NEG |
| Dresden 08_0419 | NEG | POS | NEG | NEG | NEG | NEG | POS | POS | NEG | NEG | POS | POS | POS | NEG | POS | NEG | NEG | POS | POS | NEG | NEG | POS | NEG | POS | NEG | NEG |
| Dresden 08_0484 | AMB | POS | NEG | NEG | NEG | NEG | POS | POS | NEG | NEG | POS | POS | POS | NEG | POS | NEG | NEG | POS | POS | NEG | NEG | POS | NEG | POS | NEG | NEG |
| Dresden 08_0994 | NEG | POS | NEG | NEG | NEG | NEG | POS | POS | NEG | NEG | POS | POS | POS | NEG | POS | NEG | NEG | POS | POS | NEG | NEG | POS | NEG | POS | NEG | NEG |
| Dresden 08_1148 | NEG | AMB | NEG | NEG | NEG | NEG | POS | POS | NEG | NEG | POS | POS | AMB | NEG | POS | NEG | NEG | POS | AMB | NEG | NEG | POS | NEG | POS | NEG | NEG |
| Dresden 08_1188 | NEG | AMB | NEG | NEG | NEG | NEG | POS | POS | NEG | NEG | POS | POS | POS | NEG | POS | NEG | NEG | POS | POS | NEG | NEG | POS | NEG | POS | NEG | NEG |
| Dresden 08_1205 | NEG | AMB | NEG | NEG | NEG | NEG | POS | POS | NEG | NEG | POS | POS | AMB | NEG | POS | NEG | NEG | POS | AMB | NEG | NEG | POS | NEG | POS | NEG | NEG |
| Dresden 08_1541 | POS | POS | NEG | NEG | NEG | NEG | POS | POS | NEG | NEG | POS | POS | POS | NEG | POS | NEG | NEG | POS | POS | NEG | NEG | POS | NEG | POS | NEG | NEG |
| Dresden 06_7896 | NEG | NEG | NEG | NEG | NEG | NEG | NEG | AMB | NEG | NEG | AMB | AMB | AMB | NEG | AMB | NEG | NEG | AMB | NEG | NEG | NEG | NEG | NEG | NEG | NEG | NEG |
| **>HP-25** | NEG | NEG | NEG | NEG | NEG | NEG | NEG | NEG | NEG | NEG | POS | POS | POS | NEG | NEG | NEG | NEG | POS | POS | NEG | NEG | POS | NEG | POS | NEG | NEG |
| Freiburg-809-24.809-DBK0000287 | NEG | NEG | NEG | NEG | NEG | NEG | NEG | NEG | NEG | NEG | POS | POS | POS | NEG | NEG | NEG | NEG | POS | POS | NEG | NEG | POS | NEG | POS | NEG | NEG |
| | NEG | NEG | NEG | NEG | NEG | NEG | NEG | NEG | NEG | NEG | POS | POS | POS | NEG | NEG | NEG | NEG | POS | POS | NEG | NEG | POS | NEG | POS | NEG | NEG |
| Freiburg-990-64.99-DBK0000264 | NEG | NEG | NEG | NEG | NEG | NEG | NEG | AMB | NEG | NEG | POS | POS | POS | NEG | NEG | NEG | NEG | POS | AMB | NEG | NEG | POS | NEG | POS | NEG | NEG |
| | NEG | NEG | NEG | NEG | NEG | NEG | NEG | NEG | NEG | NEG | POS | POS | POS | NEG | NEG | NEG | NEG | POS | POS | NEG | NEG | POS | NEG | POS | NEG | NEG |
| Dresden 07_7952 | NEG | NEG | NEG | NEG | NEG | NEG | NEG | NEG | NEG | NEG | POS | POS | POS | NEG | NEG | NEG | NEG | POS | POS | NEG | NEG | POS | NEG | POS | NEG | NEG |
| Dresden 07_8955 | NEG | NEG | NEG | NEG | NEG | NEG | NEG | NEG | NEG | NEG | POS | POS | POS | NEG | NEG | NEG | NEG | POS | POS | NEG | NEG | POS | NEG | POS | NEG | NEG |
| Dresden 07_8958 | NEG | NEG | NEG | NEG | NEG | NEG | NEG | AMB | NEG | NEG | POS | POS | POS | NEG | NEG | NEG | NEG | POS | AMB | NEG | NEG | POS | NEG | POS | NEG | NEG |
| Dresden 07_9027 | NEG | NEG | NEG | NEG | NEG | NEG | NEG | NEG | NEG | NEG | POS | POS | POS | NEG | NEG | NEG | NEG | POS | POS | NEG | NEG | POS | NEG | POS | NEG | NEG |
| Dresden 08_1169 | NEG | NEG | NEG | NEG | NEG | NEG | NEG | AMB | NEG | NEG | POS | POS | AMB | NEG | NEG | NEG | NEG | POS | NEG | NEG | NEG | AMB | NEG | POS | NEG | NEG |
| **>HP-26** | NEG | NEG | NEG | NEG | NEG | NEG | NEG | NEG | NEG | NEG | POS | NEG | NEG | NEG | NEG | NEG | POS | POS | POS | NEG | NEG | NEG | POS | NEG | NEG | POS |
| Freiburg-635-3.635-DBK0000297 | NEG | NEG | NEG | NEG | NEG | NEG | NEG | AMB | NEG | NEG | POS | NEG | NEG | NEG | NEG | NEG | POS | POS | POS | NEG | AMB | NEG | POS | NEG | NEG | POS |
| | NEG | NEG | NEG | NEG | NEG | NEG | NEG | NEG | NEG | NEG | POS | NEG | NEG | NEG | NEG | NEG | POS | POS | POS | NEG | NEG | NEG | POS | NEG | NEG | POS |
| Freiburg-895-38.895-DBK0000290 | NEG | NEG | NEG | NEG | NEG | NEG | NEG | AMB | NEG | NEG | POS | NEG | NEG | NEG | NEG | NEG | POS | POS | POS | NEG | NEG | NEG | POS | NEG | NEG | POS |
| | NEG | NEG | NEG | NEG | NEG | NEG | NEG | NEG | NEG | NEG | POS | NEG | NEG | NEG | NEG | NEG | POS | POS | AMB | NEG | NEG | NEG | POS | NEG | NEG | POS |
| **>HP-27** | NEG | NEG | NEG | NEG | NEG | NEG | NEG | NEG | NEG | NEG | POS | NEG | NEG | NEG | NEG | NEG | NEG | POS | POS | NEG | NEG | NEG | POS | NEG | NEG | POS |
| Freiburg-726-14.726-DBK0000228 | NEG | NEG | NEG | NEG | NEG | NEG | NEG | AMB | NEG | NEG | POS | NEG | NEG | NEG | NEG | NEG | NEG | POS | POS | NEG | AMB | NEG | POS | NEG | NEG | POS |
| | NEG | NEG | NEG | NEG | NEG | NEG | NEG | NEG | NEG | NEG | POS | NEG | NEG | NEG | NEG | NEG | NEG | POS | POS | NEG | NEG | NEG | POS | NEG | NEG | POS |
| Freiburg-837-27.837-DBK0000285 | NEG | NEG | NEG | NEG | NEG | NEG | NEG | NEG | NEG | NEG | POS | NEG | NEG | NEG | NEG | NEG | NEG | POS | POS | NEG | NEG | NEG | POS | NEG | NEG | POS |
| | NEG | NEG | NEG | NEG | NEG | NEG | NEG | NEG | NEG | NEG | POS | NEG | NEG | NEG | NEG | NEG | NEG | POS | POS | NEG | NEG | NEG | AMB | NEG | NEG | POS |
| **>HP-28** | NEG | NEG | NEG | NEG | NEG | NEG | NEG | NEG | NEG | NEG | POS | POS | POS | NEG | NEG | POS | NEG | POS | POS | NEG | POS | NEG | POS | NEG | NEG | POS |
| C. difficile 6407 (GenBank ADEH) in silico prediction | NEG | NEG | NEG | NEG | NEG | NEG | NEG | NEG | NEG | NEG | POS | POS | POS | NEG | NEG | POS | NEG | POS | POS | NEG | POS | NEG | POS | NEG | NEG | POS |
| **>HP-29** | NEG | NEG | NEG | NEG | NEG | NEG | NEG | NEG | NEG | NEG | POS | NEG | NEG | NEG | NEG | POS | NEG | POS | POS | NEG | NEG | NEG | POS | NEG | NEG | POS |
| Freiburg-852-29.852-DBK0000279 | NEG | NEG | NEG | NEG | NEG | NEG | NEG | NEG | NEG | NEG | POS | NEG | NEG | NEG | NEG | POS | NEG | POS | POS | NEG | NEG | NEG | POS | NEG | NEG | POS |
| | NEG | NEG | NEG | NEG | NEG | NEG | NEG | NEG | NEG | NEG | POS | NEG | NEG | NEG | NEG | NEG | NEG | POS | AMB | NEG | NEG | NEG | POS | NEG | NEG | POS |
| Freiburg-858-32.858-DBK0000222 | NEG | NEG | NEG | NEG | NEG | NEG | NEG | POS | NEG | NEG | POS | NEG | NEG | NEG | NEG | POS | NEG | POS | POS | NEG | NEG | NEG | POS | NEG | NEG | POS |
| **>HP-30** | NEG | NEG | NEG | NEG | NEG | NEG | NEG | NEG | NEG | NEG | POS | POS | POS | NEG | NEG | NEG | POS | POS | POS | NEG | NEG | NEG | POS | NEG | NEG | POS |
| Freiburg-1047-76.1047-DBK0000269 | NEG | NEG | NEG | NEG | NEG | NEG | NEG | NEG | NEG | NEG | POS | POS | POS | NEG | NEG | NEG | POS | POS | POS | NEG | NEG | NEG | POS | NEG | NEG | POS |
| | NEG | NEG | NEG | NEG | NEG | NEG | NEG | NEG | NEG | NEG | POS | POS | POS | NEG | NEG | NEG | POS | POS | POS | NEG | NEG | NEG | POS | NEG | NEG | POS |
| Freiburg-999-66.999-DBK0000262 | NEG | NEG | NEG | NEG | NEG | NEG | NEG | NEG | NEG | NEG | POS | POS | POS | NEG | NEG | NEG | POS | POS | POS | NEG | NEG | NEG | POS | NEG | NEG | POS |
| | NEG | NEG | NEG | NEG | NEG | NEG | NEG | NEG | NEG | NEG | POS | POS | POS | NEG | NEG | NEG | POS | POS | POS | NEG | NEG | NEG | POS | NEG | NEG | POS |
| Freiburg-896-39.896-DBK0000291 | NEG | NEG | NEG | NEG | NEG | NEG | NEG | AMB | NEG | NEG | POS | POS | POS | NEG | NEG | NEG | POS | POS | POS | NEG | NEG | NEG | POS | NEG | NEG | POS |
| | NEG | NEG | NEG | NEG | NEG | NEG | NEG | NEG | NEG | NEG | POS | POS | POS | NEG | NEG | NEG | POS | POS | POS | NEG | NEG | NEG | POS | NEG | NEG | POS |
| Freiburg-871-33.871-DBK0000272 | NEG | NEG | NEG | NEG | NEG | NEG | NEG | NEG | NEG | NEG | POS | POS | POS | NEG | NEG | NEG | POS | POS | POS | NEG | NEG | NEG | POS | NEG | NEG | POS |
| | NEG | NEG | NEG | NEG | NEG | NEG | NEG | NEG | NEG | NEG | POS | POS | POS | NEG | NEG | NEG | POS | POS | POS | NEG | NEG | NEG | POS | NEG | NEG | POS |
| Dresden 08_1596 | NEG | NEG | NEG | NEG | NEG | NEG | NEG | NEG | NEG | NEG | POS | POS | POS | NEG | NEG | NEG | POS | POS | AMB | NEG | NEG | NEG | AMB | NEG | NEG | POS |

| STRAIN / ISOLATE | Clostridium difficile binary toxin component A | | | | Clostridium difficile binary toxin component B | | | | | | channel-forming hemolysin | holin-like pore-forming protein | putative negative regulator of pathogenicity locus | cellwall protein 66 | | | | cellwall protein 84 | septum formation initiation protein | | | flagellin subunit C | | | | |
|---|---|---|---|---|---|---|---|---|---|---|---|---|---|---|---|---|---|---|---|---|---|---|---|---|---|---|
| | hp1023\|cdtA | hp1026\|cdtA | hp1027\|cdtA | hp1029\|cdtA | hp1030\|cdtB | hp1031\|cdtB | hp1038\|cdtB | hp1039\|cdtB | hp1040\|cdtB | hp1041\|cdtB | hp1103\|hly3 | hp1113\|tcdE | hp1117\|tcdC | hp1012\|cwp66 | hp1016\|cwp66 | hp1020\|cwp66 | hp1021\|cwp66 | hp1018\|cwp84 | hp1044\|divIC | hp1045\|divIC | hp1046\|divIC | hp1141\|fliC | hp1142\|fliC | hp1144\|fliC | hp1145\|fliC | hp1147\|fliC |
| **CLADE 2 (CC1 /Ribo 27 Complex)** | | | | | | | | | | | | | | | | | | | | | | | | | | |
| **>HP-31** | NEG | POS | POS | POS | NEG | POS | POS | AMB | POS | NEG | POS | POS | POS | POS | NEG | NEG | NEG | POS | POS | NEG | AMB | NEG | POS | NEG | NEG | POS |
| C. difficile BI1 (GenBank FN668941) in silico prediction | NEG | POS | POS | POS | NEG | POS | POS | AMB | POS | NEG | POS | POS | POS | POS | NEG | NEG | NEG | POS | POS | NEG | POS | NEG | POS | NEG | NEG | POS |
| C. difficile QCD-97b34 (GenBank ABHF) in silico prediction | NEG | POS | POS | POS | NEG | POS | POS | AMB | POS | NEG | POS | POS | POS | POS | NEG | NEG | NEG | POS | POS | NEG | POS | NEG | POS | NEG | NEG | POS |
| C. difficile QCD-76w55 (GenBank ABHE) in silico prediction | NEG | POS | POS | POS | NEG | POS | POS | AMB | POS | NEG | POS | POS | POS | POS | NEG | NEG | NEG | POS | POS | NEG | POS | NEG | POS | NEG | NEG | POS |
| C. difficile CIP107932 (GenBank ABKK) in silico prediction | NEG | POS | POS | POS | NEG | POS | POS | AMB | POS | NEG | POS | POS | POS | POS | NEG | NEG | NEG | POS | POS | NEG | POS | NEG | POS | NEG | NEG | POS |
| C. difficile CD196 (GenBank FN538970) in silico prediction | NEG | POS | POS | POS | NEG | POS | POS | AMB | POS | NEG | POS | POS | POS | POS | NEG | NEG | NEG | POS | POS | NEG | POS | NEG | POS | NEG | NEG | POS |
| C. difficile Strain CD196 | NEG | AMB | POS | POS | NEG | POS | POS | AMB | POS | NEG | POS | POS | POS | POS | NEG | NEG | NEG | POS | AMB | NEG | NEG | NEG | AMB | NEG | NEG | POS |
| | NEG | POS | POS | POS | NEG | POS | POS | POS | POS | NEG | POS | POS | POS | POS | NEG | NEG | NEG | POS | AMB | NEG | NEG | NEG | AMB | NEG | NEG | POS |
| | NEG | POS | POS | POS | NEG | POS | POS | POS | POS | NEG | POS | POS | POS | POS | NEG | NEG | NEG | POS | POS | NEG | NEG | NEG | POS | NEG | NEG | POS |
| | NEG | POS | POS | POS | NEG | POS | POS | AMB | POS | NEG | POS | POS | POS | POS | NEG | NEG | NEG | POS | AMB | NEG | NEG | NEG | AMB | NEG | NEG | POS |
| | NEG | AMB | POS | POS | NEG | POS | POS | POS | POS | NEG | POS | POS | POS | POS | NEG | NEG | NEG | POS | POS | NEG | NEG | NEG | POS | NEG | NEG | POS |
| Dresden 07-7829 | NEG | AMB | POS | POS | NEG | POS | POS | AMB | POS | NEG | POS | POS | POS | POS | NEG | NEG | NEG | POS | AMB | NEG | NEG | NEG | POS | NEG | NEG | POS |
| Dresden 07-8176 | NEG | POS | POS | POS | NEG | POS | POS | POS | POS | NEG | POS | POS | POS | POS | NEG | NEG | NEG | POS | POS | NEG | NEG | NEG | POS | NEG | NEG | POS |
| **>HP-32** | NEG | POS | POS | POS | NEG | POS | POS | AMB | POS | NEG | POS | POS | POS | POS | NEG | NEG | NEG | POS | POS | NEG | AMB | NEG | POS | NEG | NEG | POS |
| C. difficile 2007855 (GenBank FN665654) in silico prediction | NEG | POS | POS | POS | NEG | POS | POS | AMB | POS | NEG | POS | POS | POS | POS | NEG | NEG | NEG | POS | POS | NEG | POS | NEG | POS | NEG | NEG | POS |
| C. difficile QCD-32g58 (GenBank AAML) in silico prediction | NEG | POS | POS | POS | NEG | POS | POS | AMB | POS | NEG | POS | POS | POS | POS | NEG | NEG | NEG | POS | POS | NEG | POS | NEG | POS | NEG | NEG | POS |
| C. difficile QCD-37x79 (GenBank ABHG) in silico prediction | NEG | POS | POS | POS | NEG | POS | POS | AMB | POS | NEG | POS | POS | POS | POS | NEG | NEG | NEG | POS | POS | NEG | POS | NEG | POS | NEG | NEG | POS |
| C. difficile QCD-66c26 (GenBank ABFD) in silico prediction | NEG | POS | POS | POS | NEG | POS | POS | AMB | POS | NEG | POS | POS | POS | POS | NEG | NEG | NEG | POS | POS | NEG | POS | NEG | POS | NEG | NEG | POS |
| C. difficile R20291 (GenBank FN545816) in silico prediction | NEG | POS | POS | POS | NEG | POS | POS | AMB | POS | NEG | POS | POS | POS | POS | NEG | NEG | NEG | POS | POS | NEG | POS | NEG | POS | NEG | NEG | POS |
| C. difficile Strain R20291 | NEG | POS | POS | POS | NEG | POS | POS | POS | POS | NEG | POS | POS | POS | POS | NEG | NEG | NEG | POS | AMB | NEG | NEG | NEG | AMB | NEG | NEG | POS |
| | NEG | POS | POS | POS | NEG | POS | POS | POS | POS | NEG | POS | POS | POS | POS | NEG | NEG | NEG | POS | POS | NEG | AMB | NEG | POS | NEG | NEG | POS |
| | NEG | POS | POS | POS | NEG | POS | POS | AMB | POS | NEG | POS | POS | POS | POS | NEG | NEG | NEG | POS | POS | NEG | NEG | NEG | AMB | NEG | NEG | POS |
| | NEG | AMB | POS | POS | NEG | POS | POS | POS | POS | NEG | POS | POS | POS | POS | NEG | NEG | NEG | POS | POS | NEG | NEG | NEG | POS | NEG | NEG | POS |
| **CLADE 3 (CC5 /Ribo 23 Complex)** | | | | | | | | | | | | | | | | | | | | | | | | | | |
| **>HP-33** | NEG | NEG | POS | POS | NEG | AMB | POS | POS | AMB | NEG | POS | POS | POS | NEG | NEG | NEG | NEG | POS | POS | NEG | NEG | NEG | POS | NEG | NEG | POS |
| Freiburg872-34.872-DBK0000295 | NEG | NEG | POS | POS | NEG | POS | POS | POS | POS | NEG | POS | POS | POS | NEG | NEG | NEG | NEG | POS | POS | NEG | NEG | NEG | POS | NEG | NEG | POS |
| | NEG | NEG | POS | POS | NEG | NEG | POS | POS | NEG | NEG | POS | POS | POS | NEG | NEG | NEG | NEG | POS | AMB | NEG | NEG | NEG | AMB | NEG | NEG | POS |
| Freiburg-773-19.773-DBK0000274 | NEG | NEG | POS | POS | NEG | POS | POS | POS | AMB | NEG | POS | POS | POS | NEG | NEG | NEG | NEG | POS | POS | NEG | NEG | NEG | POS | NEG | NEG | POS |
| | NEG | NEG | POS | POS | NEG | NEG | POS | POS | NEG | NEG | POS | POS | POS | NEG | NEG | NEG | NEG | POS | AMB | NEG | NEG | NEG | POS | NEG | NEG | POS |
| **>HP-34** | NEG | NEG | POS | POS | NEG | NEG | POS | AMB | NEG | NEG | POS | POS | AMB | NEG | NEG | NEG | NEG | POS | NEG | NEG | NEG | NEG | NEG | NEG | NEG | POS |
| Jena_0018/11 | NEG | NEG | POS | POS | NEG | NEG | POS | AMB | NEG | NEG | POS | POS | AMB | NEG | NEG | NEG | NEG | POS | NEG | NEG | NEG | NEG | NEG | NEG | NEG | POS |
| **CLADE 4 (CC37/Ribo 17 Complex)** | | | | | | | | | | | | | | | | | | | | | | | | | | |
| **>HP-35** | NEG | NEG | NEG | NEG | NEG | NEG | NEG | NEG | NEG | NEG | POS | POS | POS | NEG | POS | NEG | NEG | POS | AMB | NEG | POS | POS | NEG | POS | NEG | NEG |
| C. difficile 002-P50-2011 (GenBank AGAA) in silico prediction | NEG | NEG | NEG | NEG | NEG | NEG | NEG | NEG | NEG | NEG | POS | POS | POS | NEG | POS | NEG | NEG | POS | POS | NEG | POS | POS | NEG | POS | NEG | NEG |
| C. difficile 050-P50-2011 (GenBank AGAB) in silico prediction | NEG | NEG | NEG | NEG | NEG | NEG | NEG | NEG | NEG | NEG | POS | POS | POS | NEG | POS | NEG | NEG | POS | POS | NEG | POS | POS | NEG | POS | NEG | NEG |
| C. difficile M68 (GenBank FN668375) in silico prediction | NEG | NEG | NEG | NEG | NEG | NEG | NEG | NEG | NEG | NEG | POS | POS | POS | NEG | POS | NEG | NEG | POS | POS | NEG | POS | POS | NEG | POS | NEG | NEG |
| C. difficile CF5 (GenBank FN665652) in silico prediction | NEG | NEG | NEG | NEG | NEG | NEG | NEG | NEG | NEG | NEG | POS | POS | POS | NEG | POS | NEG | NEG | POS | POS | NEG | POS | POS | NEG | POS | NEG | NEG |
| C. difficile Strain CF5 | NEG | NEG | NEG | NEG | NEG | NEG | NEG | NEG | NEG | NEG | POS | POS | POS | NEG | POS | NEG | NEG | POS | NEG | NEG | NEG | POS | NEG | POS | NEG | NEG |
| | NEG | NEG | NEG | NEG | NEG | NEG | NEG | NEG | NEG | NEG | POS | POS | POS | NEG | POS | NEG | NEG | POS | NEG | NEG | POS | POS | NEG | POS | NEG | NEG |
| | NEG | NEG | NEG | NEG | NEG | NEG | NEG | NEG | NEG | NEG | POS | POS | POS | NEG | POS | NEG | NEG | POS | NEG | NEG | NEG | POS | NEG | POS | NEG | NEG |
| | NEG | NEG | NEG | NEG | NEG | NEG | NEG | AMB | NEG | NEG | POS | POS | AMB | NEG | POS | NEG | NEG | POS | NEG | NEG | NEG | POS | NEG | POS | NEG | NEG |
| | NEG | NEG | NEG | NEG | NEG | NEG | NEG | AMB | NEG | NEG | POS | POS | POS | NEG | POS | NEG | NEG | POS | NEG | NEG | AMB | POS | NEG | POS | NEG | NEG |
| | NEG | NEG | NEG | NEG | NEG | NEG | NEG | NEG | NEG | NEG | POS | POS | POS | NEG | POS | NEG | NEG | POS | NEG | NEG | AMB | POS | NEG | POS | NEG | NEG |
| Freiburg-736-16.736-DBK0000233 | NEG | NEG | NEG | NEG | NEG | NEG | NEG | NEG | NEG | NEG | POS | POS | POS | NEG | POS | NEG | NEG | POS | AMB | NEG | POS | POS | NEG | POS | NEG | NEG |
| | NEG | NEG | NEG | NEG | NEG | NEG | NEG | NEG | NEG | NEG | POS | POS | POS | NEG | POS | NEG | NEG | POS | NEG | NEG | AMB | POS | NEG | POS | NEG | NEG |
| **>HP-36** | NEG | NEG | NEG | NEG | NEG | NEG | NEG | AMB | NEG | NEG | POS | POS | POS | NEG | NEG | NEG | POS | POS | POS | NEG | NEG | POS | NEG | POS | NEG | NEG |
| Freiburg-642-5.642-DBK0000281 | NEG | NEG | NEG | NEG | NEG | NEG | NEG | AMB | NEG | NEG | POS | POS | POS | NEG | NEG | NEG | POS | POS | POS | NEG | NEG | POS | NEG | POS | NEG | NEG |
| | NEG | NEG | NEG | NEG | NEG | NEG | NEG | NEG | NEG | NEG | POS | POS | AMB | NEG | NEG | NEG | POS | POS | AMB | NEG | NEG | POS | NEG | POS | NEG | NEG |
| **CLADE 5 (CC11 /Ribo 78 Complex)** | | | | | | | | | | | | | | | | | | | | | | | | | | |
| **>HP-37** | NEG | POS | POS | POS | POS | NEG | POS | POS | NEG | POS | POS | POS | POS | NEG | NEG | NEG | POS | POS | NEG | POS | NEG | NEG | POS | NEG | NEG | POS |
| C. difficile NAP08 (GenBank ADNX) in silico prediction | NEG | POS | POS | POS | POS | NEG | POS | AMB | NEG | POS | POS | POS | POS | NEG | NEG | NEG | POS | POS | NEG | POS | NEG | NEG | POS | NEG | NEG | POS |
| C. difficile NAP07 (GenBank ADVM) in silico prediction | NEG | POS | POS | POS | POS | NEG | POS | AMB | NEG | POS | POS | POS | POS | NEG | NEG | NEG | POS | POS | NEG | POS | NEG | NEG | POS | NEG | NEG | POS |
| C. difficile M120 (GenBank FN665653) in silico prediction | NEG | POS | POS | POS | POS | NEG | POS | AMB | NEG | POS | POS | POS | POS | NEG | NEG | NEG | POS | POS | NEG | POS | NEG | NEG | POS | NEG | NEG | POS |
| C. difficile Strain M120 | NEG | POS | POS | POS | POS | NEG | POS | AMB | NEG | POS | POS | POS | POS | NEG | NEG | NEG | POS | POS | NEG | POS | NEG | NEG | AMB | NEG | NEG | POS |
| | NEG | POS | POS | POS | POS | NEG | POS | POS | NEG | POS | POS | POS | POS | NEG | NEG | NEG | POS | POS | NEG | POS | NEG | NEG | POS | NEG | NEG | POS |
| | NEG | POS | POS | POS | POS | NEG | POS | AMB | NEG | POS | POS | POS | POS | NEG | NEG | NEG | POS | POS | NEG | POS | NEG | NEG | AMB | NEG | NEG | POS |
| | NEG | POS | POS | POS | POS | NEG | POS | POS | NEG | POS | POS | POS | POS | NEG | NEG | NEG | POS | POS | NEG | AMB | NEG | NEG | NEG | NEG | NEG | POS |
| | NEG | AMB | POS | POS | POS | NEG | POS | POS | NEG | POS | POS | POS | POS | NEG | NEG | NEG | POS | POS | NEG | POS | NEG | NEG | AMB | NEG | NEG | POS |
| Freiburg_69.1017 | NEG | NEG | POS | POS | POS | NEG | POS | AMB | NEG | POS | POS | POS | AMB | NEG | NEG | NEG | NEG | AMB | NEG | NEG | NEG | NEG | NEG | NEG | NEG | POS |
| Freiburg-1051-77.1051-DBK0000293 | NEG | POS | POS | POS | POS | NEG | POS | POS | NEG | POS | POS | POS | POS | NEG | NEG | NEG | POS | POS | NEG | POS | NEG | NEG | POS | NEG | NEG | POS |
| | NEG | POS | POS | POS | POS | NEG | POS | POS | NEG | POS | POS | POS | POS | NEG | NEG | NEG | POS | POS | NEG | POS | NEG | NEG | POS | NEG | NEG | POS |
| Freiburg-649-6.649-DBK0000235 | NEG | POS | POS | POS | POS | NEG | POS | POS | NEG | POS | POS | POS | POS | NEG | NEG | NEG | POS | POS | NEG | POS | AMB | NEG | POS | NEG | NEG | POS |
| | NEG | POS | POS | POS | POS | NEG | POS | POS | NEG | POS | POS | POS | POS | NEG | NEG | NEG | POS | POS | NEG | POS | NEG | NEG | POS | NEG | NEG | POS |
| Freiburg-700-11.7-DBK0000242 | NEG | POS | POS | POS | POS | NEG | POS | POS | NEG | POS | POS | POS | POS | NEG | NEG | NEG | POS | POS | NEG | POS | NEG | NEG | POS | NEG | NEG | POS |
| | NEG | AMB | POS | POS | POS | NEG | POS | POS | NEG | POS | POS | POS | POS | NEG | NEG | NEG | POS | POS | NEG | POS | NEG | NEG | POS | NEG | NEG | POS |
| Freiburg-855-30.855-DBK0000277 | NEG | POS | POS | POS | POS | NEG | POS | POS | NEG | POS | POS | POS | POS | NEG | NEG | NEG | POS | POS | NEG | POS | NEG | NEG | POS | NEG | NEG | POS |
| | NEG | POS | POS | POS | POS | NEG | POS | POS | NEG | POS | POS | POS | POS | NEG | NEG | NEG | POS | POS | NEG | POS | NEG | NEG | POS | NEG | NEG | POS |
| Dresden 08_1353 | NEG | POS | POS | POS | POS | NEG | POS | POS | NEG | POS | POS | POS | POS | NEG | NEG | NEG | POS | POS | NEG | POS | AMB | NEG | POS | NEG | NEG | POS |
| Dresden 08_574 | NEG | POS | POS | POS | POS | NEG | POS | POS | NEG | POS | POS | POS | POS | NEG | NEG | NEG | POS | POS | NEG | POS | NEG | NEG | POS | NEG | NEG | POS |
| Dresden 09_2650 | NEG | AMB | POS | POS | POS | NEG | POS | POS | NEG | POS | POS | POS | POS | NEG | NEG | NEG | POS | POS | NEG | AMB | NEG | NEG | AMB | NEG | NEG | POS |
| **>HP-38** | NEG | POS | POS | POS | POS | NEG | POS | AMB | NEG | POS | POS | POS | POS | NEG | NEG | NEG | POS | POS | NEG | POS | NEG | NEG | POS | NEG | NEG | POS |
| C. difficile 6466 (GenBank ADDE) in silico prediction | NEG | POS | POS | POS | POS | NEG | POS | AMB | NEG | POS | POS | POS | POS | NEG | NEG | NEG | POS | POS | NEG | POS | NEG | NEG | POS | NEG | NEG | POS |
| **>HP-39** | NEG | POS | POS | POS | POS | NEG | POS | POS | NEG | POS | POS | POS | POS | NEG | NEG | POS | NEG | POS | NEG | POS | NEG | NEG | POS | NEG | NEG | POS |
| C. difficile QCD-23m63 (GenBank ABKL) in silico prediction | NEG | POS | POS | POS | POS | NEG | POS | AMB | NEG | POS | POS | POS | POS | NEG | NEG | POS | NEG | POS | NEG | POS | NEG | NEG | POS | NEG | NEG | POS |
| Freiburg-956-51.956-DBK0000266 | NEG | POS | POS | POS | POS | NEG | POS | POS | NEG | POS | POS | POS | POS | NEG | NEG | POS | NEG | POS | NEG | POS | NEG | NEG | POS | NEG | NEG | POS |
| Freiburg_50.963 | NEG | AMB | POS | POS | POS | NEG | POS | POS | NEG | POS | POS | POS | POS | NEG | NEG | POS | NEG | POS | NEG | POS | NEG | NEG | POS | NEG | NEG | POS |
| **"CLADE 6"** | | | | | | | | | | | | | | | | | | | | | | | | | | |
| **>HP-40** | NEG | NEG | NEG | NEG | NEG | NEG | NEG | NEG | NEG | NEG | POS | NEG | NEG | NEG | NEG | NEG | POS | POS | POS | NEG | POS | POS | NEG | NEG | POS | NEG |
| C. difficile 6503 (GenBank ADEI) in silico prediction | NEG | NEG | NEG | NEG | NEG | NEG | NEG | NEG | NEG | NEG | POS | NEG | NEG | NEG | NEG | NEG | POS | POS | POS | NEG | POS | POS | NEG | NEG | POS | NEG |

| STRAIN / ISOLATE | putative endonuclease relaxase Tn1549-like | | | | | | putative calcium-binding adhesion protein | | ethanolamine iron-dependent alcohol dehydrogenase | | leucine Rich repeat-containing domain protein | splA Surface layer protein A | | | | | | | | | | | | | | | | | | | |
|---|---|---|---|---|---|---|---|---|---|---|---|---|---|---|---|---|---|---|---|---|---|---|---|---|---|---|---|---|---|---|---|
| | hp1214\|CD0425 | hp1220\|CD0425 | hp1228\|CD0425 | hp1229\|CD0425 | hp1230\|CD0425 | hp1231\|CD0425 | hp1149\|CD2797 | hp1212\|CD2797 | hp1061\|eutG | hp1062\|eutG | hp1227\|IPR007253 | hp1150\|slpA | hp1151\|slpA | hp1153\|slpA | hp1154\|slpA | hp1155\|slpA | hp1156\|slpA | hp1158\|slpA | hp1164\|slpA | hp1166\|slpA | hp1167\|slpA | hp1168\|slpA | hp1169\|slpA | hp1170\|slpA | hp1171\|slpA | hp1173\|slpA | hp1174\|slpA | hp1175\|slpA | hp1176\|slpA | hp1177\|slpA | hp1178\|slpA |
| **CLADE 1** | | | | | | | | | | | | | | | | | | | | | | | | | | | | | | | |
| **>HP-01** | NEG | NEG | POS | NEG | AMB | NEG | POS | POS | POS | NEG | NEG | NEG | POS | NEG | NEG | NEG | NEG | NEG | NEG | NEG | NEG | NEG | NEG | NEG | NEG | NEG | NEG | NEG | NEG | NEG | NEG |
| C. difficile QCD-63q42 (GenBank ABHD) in silico prediction | NEG | NEG | POS | NEG | NEG | NEG | POS | POS | POS | NEG | NEG | NEG | POS | NEG | NEG | NEG | NEG | NEG | NEG | NEG | NEG | NEG | NEG | NEG | NEG | NEG | NEG | NEG | NEG | NEG | NEG |
| C. difficile BI9 (GenBank FN668944) in silico prediction | NEG | NEG | POS | NEG | NEG | NEG | POS | POS | POS | NEG | NEG | NEG | POS | NEG | NEG | NEG | NEG | NEG | NEG | NEG | NEG | NEG | NEG | NEG | NEG | NEG | NEG | NEG | NEG | NEG | NEG |
| C. difficile BI-9 | NEG | NEG | POS | NEG | NEG | NEG | POS | AMB | POS | NEG | NEG | NEG | POS | NEG | NEG | NEG | NEG | NEG | NEG | NEG | NEG | NEG | NEG | NEG | NEG | NEG | NEG | NEG | NEG | NEG | NEG |
| | NEG | NEG | POS | AMB | NEG | NEG | POS | NEG | POS | NEG | NEG | NEG | POS | NEG | NEG | NEG | NEG | NEG | NEG | NEG | AMB | NEG | NEG | NEG | NEG | NEG | NEG | NEG | NEG | NEG | NEG |
| | NEG | NEG | POS | AMB | POS | NEG | POS | NEG | POS | NEG | NEG | NEG | POS | NEG | NEG | NEG | NEG | NEG | NEG | NEG | AMB | NEG | NEG | NEG | NEG | NEG | NEG | NEG | NEG | NEG | NEG |
| | NEG | NEG | POS | NEG | NEG | NEG | POS | NEG | POS | NEG | NEG | NEG | POS | NEG | NEG | NEG | NEG | NEG | NEG | NEG | NEG | NEG | NEG | NEG | NEG | NEG | NEG | NEG | NEG | NEG | NEG |
| | NEG | NEG | POS | NEG | NEG | NEG | POS | POS | POS | NEG | NEG | NEG | POS | NEG | NEG | NEG | NEG | NEG | NEG | NEG | NEG | NEG | NEG | NEG | NEG | NEG | NEG | NEG | NEG | NEG | NEG |
| Freiburg-1040-73.104-DBK0000249 | NEG | NEG | POS | NEG | AMB | NEG | POS | POS | POS | NEG | NEG | NEG | POS | NEG | NEG | NEG | NEG | NEG | NEG | NEG | NEG | NEG | NEG | NEG | NEG | NEG | NEG | NEG | NEG | NEG | NEG |
| | NEG | NEG | POS | NEG | NEG | NEG | POS | AMB | POS | NEG | NEG | NEG | POS | NEG | NEG | NEG | NEG | NEG | NEG | NEG | NEG | NEG | NEG | NEG | NEG | NEG | NEG | NEG | NEG | NEG | NEG |
| Freiburg-1052-78.1052-DBK0000294 | NEG | NEG | POS | AMB | POS | NEG | POS | POS | POS | NEG | NEG | NEG | POS | NEG | NEG | NEG | NEG | NEG | NEG | NEG | NEG | NEG | NEG | NEG | NEG | NEG | NEG | NEG | NEG | NEG | NEG |
| | NEG | NEG | POS | NEG | NEG | NEG | POS | POS | POS | NEG | NEG | NEG | POS | NEG | NEG | NEG | NEG | NEG | NEG | NEG | NEG | NEG | NEG | NEG | NEG | NEG | NEG | NEG | NEG | NEG | NEG |
| Freiburg-605-1.605-DBK0000223 | NEG | NEG | POS | POS | POS | AMB | POS | POS | POS | NEG | NEG | NEG | POS | NEG | NEG | NEG | NEG | NEG | NEG | NEG | NEG | NEG | NEG | NEG | NEG | NEG | NEG | NEG | NEG | NEG | NEG |
| Freiburg-787-22.787-DBK0000288 | NEG | NEG | POS | AMB | AMB | NEG | POS | POS | POS | NEG | NEG | NEG | POS | NEG | NEG | NEG | NEG | NEG | NEG | NEG | NEG | NEG | NEG | NEG | NEG | NEG | NEG | NEG | NEG | NEG | NEG |
| | NEG | NEG | POS | NEG | NEG | NEG | POS | POS | POS | NEG | NEG | NEG | POS | NEG | NEG | NEG | NEG | NEG | NEG | NEG | NEG | NEG | NEG | NEG | NEG | NEG | NEG | NEG | NEG | NEG | NEG |
| Freiburg-897-40.897-DBK0000225 | NEG | NEG | POS | POS | POS | NEG | POS | POS | POS | NEG | NEG | NEG | POS | NEG | NEG | NEG | NEG | NEG | NEG | NEG | NEG | NEG | NEG | NEG | NEG | NEG | NEG | NEG | NEG | NEG | NEG |
| | NEG | NEG | POS | NEG | NEG | NEG | POS | POS | POS | NEG | NEG | NEG | POS | NEG | NEG | NEG | NEG | NEG | NEG | NEG | NEG | NEG | NEG | NEG | NEG | NEG | NEG | NEG | NEG | NEG | NEG |
| Dresden 06_7735 | NEG | NEG | POS | AMB | NEG | NEG | POS | AMB | POS | NEG | NEG | NEG | POS | NEG | NEG | NEG | NEG | NEG | NEG | NEG | NEG | NEG | NEG | NEG | NEG | NEG | NEG | NEG | NEG | NEG | NEG |
| Dresden 06_7736 | NEG | NEG | POS | AMB | NEG | NEG | POS | NEG | AMB | NEG | NEG | NEG | POS | NEG | NEG | NEG | NEG | NEG | NEG | NEG | NEG | NEG | NEG | NEG | NEG | NEG | NEG | NEG | NEG | NEG | NEG |
| Dresden 07_6943 | NEG | NEG | POS | AMB | AMB | NEG | POS | POS | POS | NEG | NEG | NEG | POS | NEG | NEG | NEG | NEG | NEG | NEG | NEG | NEG | NEG | NEG | NEG | NEG | NEG | NEG | NEG | NEG | NEG | NEG |
| Dresden 07_7200 | NEG | NEG | POS | NEG | NEG | NEG | POS | NEG | NEG | NEG | NEG | NEG | POS | NEG | NEG | NEG | NEG | NEG | NEG | NEG | NEG | NEG | NEG | NEG | NEG | NEG | NEG | NEG | NEG | NEG | NEG |
| Dresden 07_7269 | NEG | NEG | POS | AMB | POS | NEG | POS | POS | POS | NEG | NEG | NEG | POS | NEG | NEG | NEG | NEG | NEG | NEG | NEG | NEG | NEG | NEG | NEG | NEG | NEG | NEG | NEG | NEG | NEG | NEG |
| Dresden 07_7678 | NEG | NEG | POS | AMB | AMB | NEG | POS | POS | POS | NEG | NEG | NEG | POS | NEG | NEG | NEG | NEG | NEG | NEG | NEG | NEG | NEG | NEG | NEG | NEG | NEG | NEG | NEG | NEG | NEG | NEG |
| Dresden 07_7773 | NEG | NEG | POS | NEG | NEG | NEG | POS | POS | POS | NEG | NEG | NEG | POS | NEG | NEG | NEG | NEG | NEG | NEG | NEG | NEG | NEG | NEG | NEG | NEG | NEG | NEG | NEG | NEG | NEG | NEG |
| Dresden 07_7832 | NEG | NEG | POS | AMB | POS | NEG | POS | AMB | POS | NEG | NEG | NEG | POS | NEG | NEG | NEG | NEG | NEG | NEG | NEG | NEG | NEG | NEG | NEG | NEG | NEG | NEG | NEG | NEG | NEG | NEG |
| Dresden 07_7967 | NEG | NEG | POS | POS | POS | NEG | POS | AMB | POS | NEG | NEG | NEG | POS | NEG | NEG | NEG | NEG | NEG | NEG | NEG | NEG | NEG | NEG | NEG | NEG | NEG | NEG | NEG | NEG | NEG | NEG |
| Dresden 07_8099 | NEG | NEG | POS | NEG | AMB | NEG | POS | AMB | POS | NEG | NEG | NEG | POS | NEG | NEG | NEG | NEG | NEG | NEG | NEG | NEG | NEG | NEG | NEG | NEG | NEG | NEG | NEG | NEG | NEG | NEG |
| Dresden 07_8100 | NEG | NEG | POS | AMB | POS | NEG | POS | POS | POS | NEG | NEG | NEG | POS | NEG | NEG | NEG | NEG | NEG | NEG | NEG | NEG | NEG | NEG | NEG | NEG | NEG | NEG | NEG | NEG | NEG | NEG |
| Dresden 07_8170 | NEG | NEG | POS | AMB | AMB | NEG | POS | POS | POS | NEG | NEG | NEG | POS | NEG | NEG | NEG | NEG | NEG | NEG | NEG | NEG | NEG | NEG | NEG | NEG | NEG | NEG | NEG | NEG | NEG | NEG |
| Dresden 08_0544 | NEG | NEG | POS | NEG | NEG | NEG | POS | POS | POS | NEG | NEG | NEG | POS | NEG | NEG | NEG | NEG | NEG | NEG | NEG | NEG | NEG | NEG | NEG | NEG | NEG | NEG | NEG | NEG | NEG | NEG |
| Dresden 08_0550 | NEG | NEG | POS | NEG | NEG | NEG | POS | AMB | POS | NEG | NEG | NEG | POS | NEG | NEG | NEG | NEG | NEG | NEG | NEG | NEG | NEG | NEG | NEG | NEG | NEG | NEG | NEG | NEG | NEG | NEG |
| Dresden 08_0827 | NEG | NEG | POS | NEG | NEG | NEG | POS | POS | POS | NEG | NEG | NEG | POS | NEG | NEG | NEG | NEG | NEG | NEG | NEG | NEG | NEG | NEG | NEG | NEG | NEG | NEG | NEG | NEG | NEG | NEG |
| Dresden 08_0902 | NEG | NEG | POS | AMB | NEG | NEG | POS | POS | POS | NEG | NEG | NEG | POS | NEG | NEG | NEG | NEG | NEG | NEG | NEG | NEG | NEG | NEG | NEG | NEG | NEG | NEG | NEG | NEG | NEG | NEG |
| Dresden 08_0955 | NEG | NEG | POS | AMB | AMB | NEG | POS | POS | POS | NEG | NEG | NEG | POS | NEG | NEG | NEG | NEG | NEG | NEG | NEG | NEG | NEG | NEG | NEG | NEG | NEG | NEG | NEG | NEG | NEG | NEG |
| Dresden 08_0958 | NEG | NEG | POS | NEG | NEG | NEG | POS | AMB | POS | NEG | NEG | NEG | POS | NEG | NEG | NEG | NEG | NEG | NEG | NEG | NEG | NEG | NEG | NEG | NEG | NEG | NEG | NEG | NEG | NEG | NEG |
| Dresden 08_1073 | NEG | NEG | POS | AMB | NEG | NEG | POS | POS | POS | NEG | NEG | NEG | POS | NEG | NEG | NEG | NEG | NEG | NEG | NEG | NEG | NEG | NEG | NEG | NEG | NEG | NEG | NEG | NEG | NEG | NEG |
| Dresden 08_1074 | NEG | NEG | POS | NEG | NEG | NEG | POS | POS | POS | NEG | NEG | NEG | POS | NEG | NEG | NEG | NEG | NEG | NEG | NEG | NEG | NEG | NEG | NEG | NEG | NEG | NEG | NEG | NEG | NEG | NEG |
| Dresden 08_1206 | NEG | NEG | POS | NEG | NEG | NEG | POS | POS | POS | NEG | NEG | NEG | POS | NEG | NEG | NEG | NEG | NEG | NEG | NEG | NEG | NEG | NEG | NEG | NEG | NEG | NEG | NEG | NEG | NEG | NEG |
| Dresden 08_1216 | NEG | NEG | POS | POS | AMB | NEG | POS | AMB | POS | NEG | NEG | NEG | POS | NEG | NEG | NEG | NEG | NEG | NEG | NEG | NEG | NEG | NEG | NEG | NEG | NEG | NEG | NEG | NEG | NEG | NEG |
| Dresden 08_1305 | NEG | NEG | POS | AMB | AMB | NEG | POS | POS | POS | NEG | NEG | NEG | POS | NEG | NEG | NEG | NEG | NEG | NEG | NEG | NEG | NEG | NEG | NEG | NEG | NEG | NEG | NEG | NEG | NEG | NEG |
| Dresden 07_7679 | NEG | NEG | POS | NEG | NEG | NEG | POS | NEG | AMB | NEG | NEG | NEG | POS | NEG | NEG | NEG | NEG | NEG | NEG | NEG | NEG | NEG | NEG | NEG | NEG | NEG | NEG | NEG | NEG | NEG | NEG |
| Dresden 07_7683 | NEG | NEG | POS | NEG | AMB | NEG | POS | AMB | POS | NEG | NEG | NEG | POS | NEG | NEG | NEG | NEG | NEG | NEG | NEG | NEG | NEG | NEG | NEG | NEG | NEG | NEG | NEG | NEG | NEG | NEG |
| Dresden 07_7980 | NEG | NEG | POS | NEG | NEG | NEG | POS | NEG | AMB | NEG | NEG | NEG | POS | NEG | NEG | NEG | NEG | NEG | NEG | NEG | NEG | NEG | NEG | NEG | NEG | NEG | NEG | NEG | NEG | NEG | NEG |
| Dresden 07_8022 | NEG | NEG | POS | NEG | POS | NEG | POS | AMB | POS | NEG | NEG | NEG | POS | NEG | NEG | NEG | NEG | NEG | NEG | NEG | NEG | NEG | NEG | NEG | NEG | NEG | NEG | NEG | NEG | NEG | NEG |
| Dresden 07_8291 | NEG | NEG | POS | NEG | NEG | NEG | POS | NEG | AMB | NEG | NEG | NEG | POS | NEG | NEG | NEG | NEG | NEG | NEG | NEG | NEG | NEG | NEG | NEG | NEG | NEG | NEG | NEG | NEG | NEG | NEG |
| Dresden 07_8314 | NEG | NEG | POS | AMB | POS | NEG | POS | POS | POS | NEG | NEG | NEG | POS | NEG | NEG | NEG | NEG | NEG | NEG | NEG | NEG | NEG | NEG | NEG | NEG | NEG | NEG | NEG | NEG | NEG | NEG |
| Dresden 07_8315 | NEG | NEG | POS | NEG | NEG | NEG | POS | NEG | AMB | NEG | NEG | NEG | POS | NEG | NEG | NEG | NEG | NEG | NEG | NEG | NEG | NEG | NEG | NEG | NEG | NEG | NEG | NEG | NEG | NEG | NEG |
| Dresden 07_8323 | NEG | NEG | POS | NEG | NEG | NEG | POS | NEG | AMB | NEG | NEG | NEG | POS | NEG | NEG | NEG | NEG | NEG | NEG | NEG | NEG | NEG | NEG | NEG | NEG | NEG | NEG | NEG | NEG | NEG | NEG |
| Dresden 07_8356 | NEG | NEG | POS | NEG | NEG | NEG | POS | AMB | POS | NEG | NEG | NEG | POS | NEG | NEG | NEG | NEG | NEG | NEG | NEG | NEG | NEG | NEG | NEG | NEG | NEG | NEG | NEG | NEG | NEG | NEG |
| Dresden 07_8557 | NEG | NEG | POS | NEG | NEG | NEG | POS | NEG | AMB | NEG | NEG | NEG | POS | NEG | NEG | NEG | NEG | NEG | NEG | NEG | NEG | NEG | NEG | NEG | NEG | NEG | NEG | NEG | NEG | NEG | NEG |
| Dresden 07_8736 | NEG | NEG | POS | NEG | NEG | NEG | POS | NEG | AMB | NEG | NEG | NEG | POS | NEG | NEG | NEG | NEG | NEG | NEG | NEG | NEG | NEG | NEG | NEG | NEG | NEG | NEG | NEG | NEG | NEG | NEG |
| Dresden 07_8945 | NEG | NEG | POS | NEG | AMB | NEG | POS | AMB | POS | NEG | NEG | NEG | POS | NEG | NEG | NEG | NEG | NEG | NEG | NEG | NEG | NEG | NEG | NEG | NEG | NEG | NEG | NEG | NEG | NEG | NEG |
| Dresden 07_9138 | NEG | NEG | POS | NEG | POS | NEG | POS | POS | POS | NEG | NEG | NEG | POS | NEG | NEG | NEG | NEG | NEG | NEG | NEG | NEG | NEG | NEG | NEG | NEG | NEG | NEG | NEG | NEG | NEG | NEG |
| Dresden 07_9175 | NEG | NEG | POS | NEG | NEG | NEG | POS | POS | POS | NEG | NEG | NEG | POS | NEG | NEG | NEG | NEG | NEG | NEG | NEG | NEG | NEG | NEG | NEG | NEG | NEG | NEG | NEG | NEG | NEG | NEG |
| Dresden 07_9234 | NEG | NEG | POS | AMB | POS | NEG | POS | POS | POS | NEG | NEG | NEG | POS | NEG | NEG | NEG | NEG | NEG | NEG | NEG | NEG | NEG | NEG | NEG | NEG | NEG | NEG | NEG | NEG | NEG | NEG |
| Dresden 07_9255 | NEG | NEG | POS | AMB | POS | NEG | POS | POS | POS | NEG | NEG | NEG | POS | NEG | NEG | NEG | NEG | NEG | NEG | NEG | NEG | NEG | NEG | NEG | NEG | NEG | NEG | NEG | NEG | NEG | NEG |
| Dresden 07_9309 | NEG | NEG | POS | AMB | AMB | NEG | POS | POS | POS | NEG | NEG | NEG | POS | NEG | NEG | NEG | NEG | NEG | NEG | NEG | NEG | NEG | NEG | NEG | NEG | NEG | NEG | NEG | NEG | NEG | NEG |
| Dresden 07_9330 | NEG | NEG | POS | NEG | NEG | NEG | POS | POS | POS | NEG | NEG | NEG | POS | NEG | NEG | NEG | NEG | NEG | NEG | NEG | NEG | NEG | NEG | NEG | NEG | NEG | NEG | NEG | NEG | NEG | NEG |
| Dresden 07_9399 | NEG | NEG | POS | NEG | AMB | NEG | POS | POS | POS | NEG | NEG | NEG | POS | NEG | NEG | NEG | NEG | NEG | NEG | NEG | NEG | NEG | NEG | NEG | NEG | NEG | NEG | NEG | NEG | NEG | NEG |
| Dresden 08_0188 | NEG | NEG | POS | AMB | AMB | NEG | POS | POS | POS | NEG | NEG | NEG | POS | NEG | NEG | NEG | NEG | NEG | NEG | NEG | NEG | NEG | NEG | NEG | NEG | NEG | NEG | NEG | NEG | NEG | NEG |
| Dresden 08_0190 | NEG | NEG | POS | NEG | NEG | NEG | POS | POS | POS | NEG | NEG | NEG | POS | NEG | NEG | NEG | NEG | NEG | NEG | NEG | NEG | NEG | NEG | NEG | NEG | NEG | NEG | NEG | NEG | NEG | NEG |
| Dresden 08_0220 | NEG | NEG | POS | NEG | NEG | NEG | POS | POS | POS | NEG | NEG | NEG | POS | NEG | NEG | NEG | NEG | NEG | NEG | NEG | NEG | NEG | NEG | NEG | NEG | NEG | NEG | NEG | NEG | NEG | NEG |
| Dresden 08_0326 | NEG | NEG | POS | NEG | AMB | NEG | POS | POS | POS | NEG | NEG | NEG | POS | NEG | NEG | NEG | NEG | NEG | NEG | NEG | NEG | NEG | NEG | NEG | NEG | NEG | NEG | NEG | NEG | NEG | NEG |
| Dresden 08_0327 | NEG | NEG | POS | NEG | AMB | NEG | POS | POS | POS | NEG | NEG | NEG | POS | NEG | NEG | NEG | NEG | NEG | NEG | NEG | NEG | NEG | NEG | NEG | NEG | NEG | NEG | NEG | NEG | NEG | NEG |
| Dresden 08_0648 | NEG | NEG | POS | AMB | AMB | NEG | POS | POS | POS | NEG | NEG | NEG | POS | NEG | NEG | NEG | NEG | NEG | NEG | NEG | NEG | NEG | NEG | NEG | NEG | NEG | NEG | NEG | NEG | NEG | NEG |
| Dresden 08_0751 | NEG | NEG | POS | NEG | NEG | NEG | POS | POS | POS | NEG | NEG | NEG | POS | NEG | NEG | NEG | NEG | NEG | NEG | NEG | NEG | NEG | NEG | NEG | NEG | NEG | NEG | NEG | NEG | NEG | NEG |
| Dresden 08_0769 | NEG | NEG | POS | NEG | NEG | NEG | POS | POS | POS | NEG | NEG | NEG | POS | NEG | NEG | NEG | NEG | NEG | NEG | NEG | NEG | NEG | NEG | NEG | NEG | NEG | NEG | NEG | NEG | NEG | NEG |
| Dresden 08_0770 | NEG | NEG | POS | NEG | NEG | NEG | POS | POS | POS | NEG | NEG | NEG | POS | NEG | NEG | NEG | NEG | NEG | NEG | NEG | NEG | NEG | NEG | NEG | NEG | NEG | NEG | NEG | NEG | NEG | NEG |
| Dresden 08_0771 | NEG | NEG | POS | AMB | AMB | NEG | POS | POS | POS | NEG | NEG | NEG | POS | NEG | NEG | NEG | NEG | NEG | NEG | NEG | NEG | NEG | NEG | NEG | NEG | NEG | NEG | NEG | NEG | NEG | NEG |
| Dresden 08_1308 | NEG | NEG | POS | AMB | AMB | NEG | POS | POS | POS | NEG | NEG | NEG | POS | NEG | NEG | NEG | NEG | NEG | NEG | NEG | NEG | NEG | NEG | NEG | NEG | NEG | NEG | NEG | NEG | NEG | NEG |
| Dresden 08_1311 | NEG | NEG | POS | NEG | NEG | NEG | POS | POS | POS | NEG | NEG | NEG | POS | NEG | NEG | NEG | NEG | NEG | NEG | NEG | NEG | NEG | NEG | NEG | NEG | NEG | NEG | NEG | NEG | NEG | NEG |
| Dresden 08_1316 | NEG | NEG | POS | NEG | NEG | NEG | POS | POS | POS | NEG | NEG | NEG | POS | NEG | NEG | NEG | NEG | NEG | NEG | NEG | NEG | NEG | NEG | NEG | NEG | NEG | NEG | NEG | NEG | NEG | NEG |
| Dresden 08_1542 | NEG | NEG | POS | AMB | POS | NEG | POS | POS | POS | NEG | NEG | NEG | POS | NEG | NEG | NEG | NEG | NEG | NEG | NEG | NEG | NEG | NEG | NEG | NEG | NEG | NEG | NEG | NEG | NEG | NEG |
| Dresden 08_1544 | NEG | NEG | POS | NEG | NEG | NEG | POS | POS | POS | NEG | NEG | NEG | POS | NEG | NEG | NEG | NEG | NEG | NEG | NEG | NEG | NEG | NEG | NEG | NEG | NEG | NEG | NEG | NEG | NEG | NEG |
| Dresden 08_1581 | NEG | NEG | POS | AMB | AMB | NEG | POS | POS | POS | NEG | NEG | NEG | POS | NEG | NEG | NEG | NEG | NEG | NEG | NEG | NEG | NEG | NEG | NEG | NEG | NEG | NEG | NEG | NEG | NEG | NEG |
| Dresden 08_1826 | NEG | NEG | POS | AMB | AMB | NEG | POS | POS | POS | NEG | NEG | NEG | POS | NEG | NEG | NEG | NEG | NEG | NEG | NEG | NEG | NEG | NEG | NEG | NEG | NEG | NEG | NEG | NEG | NEG | NEG |
| Dresden 08_1817 | NEG | NEG | POS | NEG | NEG | NEG | POS | POS | POS | NEG | NEG | NEG | POS | NEG | NEG | NEG | NEG | NEG | NEG | NEG | NEG | NEG | NEG | NEG | NEG | NEG | NEG | NEG | NEG | NEG | NEG |
| Dresden 08_1791 | NEG | NEG | POS | NEG | NEG | NEG | POS | AMB | POS | NEG | NEG | NEG | POS | NEG | NEG | NEG | NEG | NEG | NEG | NEG | NEG | NEG | NEG | NEG | NEG | NEG | NEG | NEG | NEG | NEG | NEG |
| Dresden 08_1643 | NEG | NEG | POS | NEG | NEG | NEG | POS | POS | POS | NEG | NEG | NEG | POS | NEG | NEG | NEG | NEG | NEG | NEG | NEG | NEG | NEG | NEG | NEG | NEG | NEG | NEG | NEG | NEG | NEG | NEG |
| Dresden 08_1764 | NEG | NEG | POS | NEG | NEG | NEG | POS | POS | POS | NEG | NEG | NEG | POS | NEG | NEG | NEG | NEG | NEG | NEG | NEG | NEG | NEG | NEG | NEG | NEG | NEG | NEG | NEG | NEG | NEG | NEG |
| Dresden 08_1748 | NEG | NEG | POS | NEG | AMB | NEG | POS | POS | POS | NEG | NEG | NEG | POS | NEG | NEG | NEG | NEG | NEG | NEG | NEG | NEG | NEG | NEG | NEG | NEG | NEG | NEG | NEG | NEG | NEG | NEG |
| Dresden 08_1736 | NEG | NEG | POS | NEG | NEG | NEG | POS | POS | POS | NEG | NEG | NEG | POS | NEG | NEG | NEG | NEG | NEG | NEG | NEG | NEG | NEG | NEG | NEG | NEG | NEG | NEG | NEG | NEG | NEG | NEG |
| Dresden 08_1582 | NEG | NEG | POS | NEG | NEG | NEG | POS | POS | POS | NEG | NEG | NEG | POS | NEG | NEG | NEG | NEG | NEG | NEG | NEG | NEG | NEG | NEG | NEG | NEG | NEG | NEG | NEG | NEG | NEG | NEG |
| **>HP-02** | NEG | NEG | NEG | AMB | AMB | NEG | POS | POS | POS | NEG | NEG | NEG | POS | NEG | NEG | NEG | NEG | NEG | NEG | NEG | NEG | NEG | NEG | NEG | NEG | NEG | NEG | NEG | NEG | NEG | NEG |
| C. difficile ATCC43255 (GenBank ABKJ) in silico prediction | NEG | NEG | NEG | NEG | NEG | NEG | POS | POS | POS | NEG | NEG | NEG | POS | NEG | NEG | NEG | NEG | NEG | NEG | NEG | NEG | NEG | NEG | NEG | NEG | NEG | NEG | NEG | NEG | NEG | NEG |
| Freiburg-885-36.885-DBK0000296 | NEG | NEG | NEG | AMB | POS | NEG | POS | POS | POS | NEG | NEG | NEG | POS | NEG | NEG | NEG | NEG | NEG | NEG | NEG | NEG | NEG | NEG | NEG | NEG | NEG | NEG | NEG | NEG | NEG | NEG |
| Freiburg_37.894-II | NEG | NEG | NEG | NEG | NEG | NEG | POS | AMB | POS | NEG | NEG | NEG | POS | NEG | NEG | NEG | NEG | NEG | NEG | NEG | NEG | NEG | NEG | NEG | NEG | NEG | NEG | NEG | NEG | NEG | NEG |
| Freiburg_981-57.981-DBK0000258 | NEG | NEG | NEG | NEG | NEG | NEG | POS | NEG | POS | NEG | NEG | NEG | POS | NEG | NEG | NEG | NEG | NEG | NEG | NEG | NEG | NEG | NEG | NEG | NEG | NEG | NEG | NEG | NEG | NEG | NEG |
| | NEG | NEG | NEG | POS | POS | AMB | POS | POS | POS | NEG | NEG | NEG | POS | NEG | NEG | NEG | NEG | NEG | NEG | NEG | NEG | NEG | NEG | NEG | NEG | NEG | NEG | NEG | NEG | NEG | NEG |
| Jena_S0011_11 | NEG | NEG | NEG | NEG | AMB | NEG | POS | POS | POS | NEG | NEG | NEG | POS | NEG | NEG | NEG | NEG | NEG | NEG | NEG | NEG | NEG | NEG | NEG | NEG | NEG | NEG | NEG | NEG | NEG | NEG |
| | NEG | NEG | NEG | NEG | NEG | NEG | POS | POS | POS | NEG | NEG | NEG | POS | NEG | NEG | NEG | NEG | NEG | NEG | NEG | NEG | NEG | NEG | NEG | NEG | NEG | NEG | NEG | NEG | NEG | NEG |
| Dresden 08_1352 | NEG | NEG | NEG | AMB | POS | NEG | POS | POS | POS | NEG | NEG | NEG | POS | NEG | NEG | NEG | NEG | NEG | NEG | NEG | NEG | NEG | NEG | NEG | NEG | NEG | NEG | NEG | NEG | NEG | NEG |
| **>HP-03** | NEG | NEG | NEG | AMB | AMB | NEG | POS | POS | POS | NEG | AMB | NEG | NEG | NEG | NEG | NEG | NEG | NEG | NEG | NEG | POS | POS | NEG | NEG | NEG | NEG | NEG | NEG | NEG | NEG | NEG |
| Freiburg-938-47.938-DBK0000237 | NEG | NEG | NEG | AMB | POS | NEG | POS | POS | POS | NEG | POS | NEG | NEG | NEG | NEG | NEG | NEG | NEG | NEG | NEG | POS | POS | NEG | NEG | NEG | NEG | NEG | NEG | NEG | NEG | NEG |
| | NEG | NEG | NEG | NEG | AMB | NEG | POS | POS | POS | NEG | POS | NEG | NEG | NEG | NEG | NEG | NEG | NEG | NEG | NEG | POS | POS | NEG | NEG | NEG | NEG | NEG | NEG | NEG | NEG | NEG |
| Freiburg-811-25.811-DBK0000284 | NEG | NEG | NEG | NEG | NEG | NEG | POS | AMB | POS | NEG | NEG | NEG | NEG | NEG | NEG | NEG | NEG | NEG | NEG | NEG | POS | POS | NEG | NEG | NEG | NEG | NEG | NEG | NEG | NEG | NEG |
| | NEG | NEG | NEG | NEG | NEG | NEG | POS | POS | POS | NEG | NEG | NEG | NEG | NEG | NEG | NEG | NEG | NEG | NEG | NEG | POS | POS | NEG | NEG | NEG | NEG | NEG | NEG | NEG | NEG | NEG |
| Freiburg-987-62.987-DBK0000251 | NEG | NEG | NEG | POS | POS | NEG | POS | POS | POS | NEG | POS | NEG | NEG | NEG | NEG | NEG | NEG | NEG | NEG | NEG | POS | POS | NEG | NEG | NEG | NEG | NEG | NEG | NEG | NEG | NEG |
| | NEG | NEG | NEG | NEG | NEG | NEG | POS | NEG | POS | NEG | NEG | NEG | NEG | NEG | NEG | NEG | NEG | NEG | NEG | NEG | POS | POS | NEG | NEG | NEG | NEG | NEG | NEG | NEG | NEG | NEG |
| Freiburg-972-54.972-DBK0000247 | NEG | NEG | NEG | POS | POS | NEG | POS | POS | POS | NEG | NEG | NEG | NEG | NEG | NEG | NEG | NEG | NEG | NEG | NEG | POS | POS | NEG | NEG | NEG | NEG | NEG | NEG | NEG | NEG | NEG |
| | NEG | NEG | NEG | NEG | NEG | NEG | POS | AMB | POS | NEG | NEG | NEG | NEG | NEG | NEG | NEG | NEG | NEG | NEG | NEG | POS | POS | NEG | NEG | NEG | NEG | NEG | NEG | NEG | NEG | NEG |
| **>HP-04** | NEG | NEG | NEG | AMB | POS | NEG | POS | POS | POS | NEG | NEG | NEG | NEG | NEG | NEG | NEG | NEG | NEG | NEG | POS | NEG | NEG | NEG | NEG | NEG | NEG | NEG | NEG | NEG | NEG | NEG |
| Freiburg-984-60.984-DBK0000250 | NEG | NEG | NEG | POS | POS | NEG | POS | POS | POS | NEG | NEG | NEG | NEG | NEG | NEG | NEG | NEG | NEG | NEG | POS | NEG | NEG | NEG | NEG | NEG | NEG | NEG | NEG | NEG | NEG | NEG |
| Freiburg-1023-72.1023-DBK0000271 | NEG | NEG | NEG | POS | POS | NEG | POS | POS | POS | NEG | NEG | NEG | NEG | NEG | NEG | NEG | NEG | NEG | NEG | POS | NEG | NEG | NEG | NEG | NEG | NEG | NEG | NEG | NEG | NEG | NEG |
| | NEG | NEG | NEG | AMB | AMB | NEG | POS | POS | POS | NEG | NEG | NEG | NEG | NEG | NEG | NEG | NEG | NEG | NEG | POS | NEG | NEG | NEG | NEG | NEG | NEG | NEG | NEG | NEG | NEG | NEG |
| Freiburg-1043-74.1043-DBK0000268 | NEG | NEG | NEG | AMB | POS | NEG | POS | POS | POS | NEG | NEG | NEG | NEG | NEG | NEG | NEG | NEG | NEG | NEG | POS | NEG | NEG | NEG | NEG | NEG | NEG | NEG | NEG | NEG | NEG | NEG |
| Freiburg-633-2.663-DBK0000292 | NEG | NEG | NEG | POS | POS | AMB | POS | POS | POS | NEG | NEG | NEG | NEG | NEG | NEG | NEG | NEG | NEG | NEG | POS | NEG | NEG | NEG | NEG | NEG | NEG | NEG | NEG | NEG | NEG | NEG |
| | NEG | NEG | NEG | NEG | NEG | NEG | POS | NEG | POS | NEG | NEG | NEG | NEG | NEG | NEG | NEG | NEG | NEG | NEG | POS | NEG | NEG | NEG | NEG | NEG | NEG | NEG | NEG | NEG | NEG | NEG |
| Freiburg-782-20.782-DBK0000276 | NEG | NEG | NEG | AMB | POS | NEG | POS | POS | POS | NEG | NEG | NEG | NEG | NEG | NEG | NEG | NEG | NEG | NEG | POS | NEG | NEG | NEG | NEG | NEG | NEG | NEG | NEG | NEG | NEG | NEG |
| | NEG | NEG | NEG | NEG | NEG | NEG | POS | AMB | POS | NEG | NEG | NEG | NEG | NEG | NEG | NEG | NEG | NEG | NEG | POS | NEG | NEG | NEG | NEG | NEG | NEG | NEG | NEG | NEG | NEG | NEG |
| **>HP-05** | NEG | NEG | NEG | AMB | AMB | NEG | POS | POS | POS | NEG | NEG | NEG | NEG | NEG | NEG | NEG | NEG | NEG | NEG | NEG | NEG | NEG | NEG | NEG | NEG | NEG | NEG | NEG | NEG | NEG | NEG |
| Freiburg-740-17.74-DBK0000234 | NEG | NEG | NEG | POS | POS | NEG | POS | POS | POS | NEG | NEG | NEG | NEG | NEG | NEG | NEG | NEG | NEG | NEG | NEG | NEG | NEG | NEG | NEG | NEG | NEG | NEG | NEG | NEG | NEG | NEG |
| | NEG | NEG | NEG | NEG | NEG | NEG | POS | NEG | POS | NEG | NEG | NEG | NEG | NEG | NEG | NEG | NEG | NEG | NEG | NEG | NEG | NEG | NEG | NEG | NEG | NEG | NEG | NEG | NEG | NEG | NEG |
| Freiburg-793-23.793-DBK0000286 | NEG | NEG | NEG | POS | POS | NEG | POS | POS | POS | NEG | NEG | NEG | NEG | NEG | NEG | NEG | NEG | NEG | NEG | NEG | NEG | NEG | NEG | NEG | NEG | NEG | NEG | NEG | NEG | NEG | NEG |
| | NEG | NEG | NEG | AMB | AMB | NEG | POS | AMB | POS | NEG | NEG | NEG | NEG | NEG | NEG | NEG | NEG | NEG | NEG | NEG | NEG | NEG | NEG | NEG | NEG | NEG | NEG | NEG | NEG | NEG | NEG |
| **>HP-06** | NEG | NEG | NEG | AMB | AMB | NEG | POS | POS | POS | NEG | NEG | NEG | NEG | NEG | NEG | NEG | NEG | NEG | NEG | NEG | NEG | NEG | NEG | NEG | NEG | NEG | NEG | NEG | NEG | NEG | NEG |
| Freiburg-874-35.874-DBK0000282 | NEG | NEG | NEG | AMB | POS | NEG | POS | POS | POS | NEG | NEG | NEG | NEG | NEG | NEG | NEG | NEG | NEG | NEG | NEG | NEG | NEG | NEG | NEG | NEG | NEG | NEG | NEG | NEG | NEG | NEG |
| | NEG | NEG | NEG | AMB | AMB | NEG | POS | POS | POS | NEG | NEG | NEG | NEG | NEG | NEG | NEG | NEG | NEG | NEG | NEG | NEG | NEG | NEG | NEG | NEG | NEG | NEG | NEG | NEG | NEG | NEG |

| STRAIN / ISOLATE | putative endonuclease relaxase Tn1549-like | | | | | | putative calcium-binding adhesion protein | | ethanolamine iron-dependent alcohol dehydrogenase | | leucine Rich repeat-containing domain protein | splA Surface layer protein A | | | | | | | | | | | | | | | | | | | |
|---|---|---|---|---|---|---|---|---|---|---|---|---|---|---|---|---|---|---|---|---|---|---|---|---|---|---|---|---|---|---|---|
| | hp1214\|CD0425 | hp1220\|CD0425 | hp1228\|CD0425 | hp1229\|CD0425 | hp1230\|CD0425 | hp1231\|CD0425 | hp1149\|CD2797 | hp1212\|CD2797 | hp1061\|eutG | hp1062\|eutG | hp1227\|IPR007253 | hp1150\|slpA | hp1151\|slpA | hp1153\|slpA | hp1154\|slpA | hp1155\|slpA | hp1156\|slpA | hp1158\|slpA | hp1164\|slpA | hp1166\|slpA | hp1167\|slpA | hp1168\|slpA | hp1169\|slpA | hp1170\|slpA | hp1171\|slpA | hp1173\|slpA | hp1174\|slpA | hp1175\|slpA | hp1176\|slpA | hp1177\|slpA | hp1178\|slpA |
| **>HP-07** | NEG | NEG | NEG | POS | POS | NEG | POS | NEG | POS | NEG | POS | NEG | NEG | NEG | NEG | NEG | POS | NEG | NEG | NEG | NEG | NEG | NEG | NEG | NEG | NEG | NEG | NEG | NEG | NEG | NEG |
| Freiburg-923-44.923-DBK0000238 | NEG | NEG | NEG | POS | POS | AMB | POS | NEG | POS | NEG | POS | NEG | NEG | NEG | NEG | NEG | POS | NEG | NEG | NEG | NEG | NEG | NEG | NEG | NEG | NEG | NEG | NEG | NEG | NEG | NEG |
| | NEG | NEG | NEG | NEG | AMB | NEG | POS | NEG | POS | NEG | POS | NEG | NEG | NEG | NEG | NEG | POS | NEG | NEG | NEG | NEG | NEG | NEG | NEG | NEG | NEG | NEG | NEG | NEG | NEG | NEG |
| **>HP-08** | NEG | NEG | NEG | AMB | AMB | NEG | POS | POS | POS | NEG | NEG | NEG | NEG | NEG | NEG | NEG | NEG | NEG | NEG | NEG | NEG | NEG | NEG | NEG | NEG | NEG | NEG | NEG | NEG | NEG | NEG |
| Freiburg-1021-71.1021-DBK0000248 | NEG | NEG | NEG | AMB | POS | NEG | POS | POS | POS | NEG | NEG | NEG | NEG | NEG | NEG | NEG | NEG | NEG | NEG | NEG | NEG | NEG | NEG | NEG | NEG | NEG | NEG | NEG | NEG | NEG | NEG |
| | NEG | NEG | NEG | NEG | NEG | NEG | POS | POS | POS | NEG | NEG | NEG | NEG | NEG | NEG | NEG | NEG | NEG | NEG | NEG | NEG | NEG | NEG | NEG | NEG | NEG | NEG | NEG | NEG | NEG | NEG |
| Freiburg-669-9.669-DBK0000231 | NEG | NEG | NEG | NEG | NEG | NEG | POS | POS | POS | NEG | NEG | NEG | NEG | NEG | NEG | NEG | NEG | NEG | NEG | NEG | NEG | NEG | NEG | NEG | NEG | NEG | NEG | NEG | NEG | NEG | NEG |
| | NEG | NEG | NEG | AMB | AMB | NEG | POS | POS | POS | NEG | NEG | NEG | NEG | NEG | NEG | NEG | NEG | NEG | NEG | NEG | NEG | NEG | NEG | NEG | NEG | NEG | NEG | NEG | NEG | NEG | NEG |
| Freiburg-964-53.964-DBK0000267 | NEG | NEG | NEG | POS | POS | NEG | POS | POS | POS | NEG | NEG | NEG | NEG | NEG | NEG | NEG | NEG | NEG | NEG | NEG | NEG | NEG | NEG | NEG | NEG | NEG | NEG | NEG | NEG | NEG | NEG |
| | NEG | NEG | NEG | NEG | NEG | NEG | POS | POS | POS | NEG | NEG | NEG | NEG | NEG | NEG | NEG | NEG | NEG | NEG | NEG | NEG | NEG | NEG | NEG | NEG | NEG | NEG | NEG | NEG | NEG | NEG |
| Freiburg-899-41.899-DBK0000224 | NEG | NEG | NEG | POS | POS | NEG | POS | POS | POS | NEG | NEG | NEG | NEG | NEG | NEG | NEG | NEG | NEG | NEG | NEG | NEG | NEG | NEG | NEG | NEG | NEG | NEG | NEG | NEG | NEG | NEG |
| | NEG | NEG | NEG | NEG | NEG | NEG | POS | NEG | AMB | NEG | NEG | NEG | NEG | NEG | NEG | NEG | NEG | NEG | NEG | NEG | NEG | NEG | NEG | NEG | NEG | NEG | NEG | NEG | NEG | NEG | NEG |
| Freiburg-982-58.982-DBK0000257 | NEG | NEG | NEG | POS | POS | NEG | POS | POS | POS | NEG | NEG | NEG | NEG | NEG | NEG | NEG | NEG | NEG | NEG | NEG | NEG | NEG | NEG | NEG | NEG | NEG | NEG | NEG | NEG | NEG | NEG |
| | NEG | NEG | NEG | NEG | NEG | NEG | POS | NEG | AMB | NEG | NEG | NEG | NEG | NEG | NEG | NEG | NEG | NEG | NEG | NEG | NEG | NEG | NEG | NEG | NEG | NEG | NEG | NEG | NEG | NEG | NEG |
| Dresden 07_7035 | NEG | NEG | NEG | AMB | NEG | NEG | POS | AMB | POS | NEG | NEG | NEG | NEG | NEG | NEG | NEG | NEG | NEG | NEG | NEG | NEG | NEG | NEG | NEG | NEG | NEG | NEG | NEG | NEG | NEG | NEG |
| Dresden 07_7248 | NEG | NEG | NEG | AMB | POS | NEG | POS | POS | POS | NEG | NEG | NEG | NEG | NEG | NEG | NEG | NEG | NEG | NEG | NEG | NEG | NEG | NEG | NEG | NEG | NEG | NEG | NEG | NEG | NEG | NEG |
| Dresden 07_8702 | NEG | NEG | NEG | NEG | NEG | NEG | POS | AMB | POS | NEG | NEG | NEG | NEG | NEG | NEG | NEG | NEG | NEG | NEG | NEG | NEG | NEG | NEG | NEG | NEG | NEG | NEG | NEG | NEG | NEG | NEG |
| Dresden 07_8989 | NEG | NEG | NEG | NEG | NEG | NEG | POS | AMB | POS | NEG | NEG | NEG | NEG | NEG | NEG | NEG | NEG | NEG | NEG | NEG | NEG | NEG | NEG | NEG | NEG | NEG | NEG | NEG | NEG | NEG | NEG |
| Dresden 08_0712 | NEG | NEG | NEG | NEG | NEG | NEG | POS | POS | POS | NEG | NEG | NEG | NEG | NEG | NEG | NEG | NEG | NEG | NEG | NEG | NEG | NEG | NEG | NEG | NEG | NEG | NEG | NEG | NEG | NEG | NEG |
| **>HP-09** | NEG | NEG | NEG | NEG | NEG | NEG | POS | AMB | POS | NEG | NEG | NEG | NEG | NEG | NEG | POS | NEG | NEG | NEG | NEG | NEG | NEG | NEG | NEG | NEG | NEG | NEG | NEG | NEG | NEG | NEG |
| Freiburg-1056-79.1056-DBK0000261 | NEG | NEG | NEG | NEG | NEG | NEG | POS | POS | POS | NEG | POS | NEG | NEG | NEG | NEG | POS | NEG | NEG | NEG | NEG | NEG | NEG | NEG | NEG | NEG | NEG | NEG | NEG | NEG | NEG | NEG |
| Freiburg-658-8.658-DBK0000230 | NEG | NEG | NEG | AMB | POS | AMB | POS | POS | POS | NEG | POS | NEG | NEG | NEG | NEG | POS | NEG | NEG | NEG | NEG | NEG | NEG | NEG | NEG | NEG | NEG | NEG | NEG | NEG | NEG | NEG |
| | NEG | NEG | NEG | NEG | NEG | NEG | POS | AMB | POS | NEG | POS | NEG | NEG | NEG | NEG | POS | NEG | NEG | NEG | NEG | NEG | NEG | NEG | NEG | NEG | NEG | NEG | NEG | NEG | NEG | NEG |
| Freiburg-963-52.963-DBK0000265 | NEG | NEG | NEG | AMB | AMB | NEG | POS | POS | POS | NEG | NEG | NEG | NEG | NEG | NEG | POS | NEG | NEG | NEG | NEG | NEG | NEG | NEG | NEG | NEG | NEG | NEG | NEG | NEG | NEG | NEG |
| | NEG | NEG | NEG | NEG | NEG | NEG | POS | POS | POS | NEG | NEG | NEG | NEG | NEG | NEG | POS | NEG | NEG | NEG | NEG | NEG | NEG | NEG | NEG | NEG | NEG | NEG | NEG | NEG | NEG | NEG |
| Freiburg-988-63.988-DBK0000253 | NEG | NEG | NEG | AMB | AMB | NEG | POS | POS | POS | NEG | POS | NEG | NEG | NEG | NEG | POS | NEG | NEG | NEG | NEG | NEG | NEG | NEG | NEG | NEG | NEG | NEG | NEG | NEG | NEG | NEG |
| | NEG | NEG | NEG | NEG | NEG | NEG | POS | AMB | POS | NEG | NEG | NEG | NEG | NEG | NEG | POS | NEG | NEG | NEG | NEG | NEG | NEG | NEG | NEG | NEG | NEG | NEG | NEG | NEG | NEG | NEG |
| Freiburg_79.1057 | NEG | NEG | NEG | NEG | NEG | NEG | POS | POS | POS | NEG | AMB | NEG | NEG | NEG | NEG | POS | NEG | NEG | NEG | NEG | NEG | NEG | NEG | NEG | NEG | NEG | NEG | NEG | NEG | NEG | NEG |
| Jena_S0017.11 | NEG | NEG | NEG | NEG | NEG | NEG | POS | AMB | POS | NEG | NEG | NEG | NEG | NEG | NEG | POS | NEG | NEG | NEG | NEG | NEG | NEG | NEG | NEG | NEG | NEG | NEG | NEG | NEG | NEG | NEG |
| | NEG | NEG | NEG | NEG | NEG | NEG | POS | POS | POS | NEG | NEG | NEG | NEG | NEG | NEG | POS | NEG | NEG | NEG | NEG | NEG | NEG | NEG | NEG | NEG | NEG | NEG | NEG | NEG | NEG | NEG |
| | NEG | NEG | NEG | NEG | NEG | NEG | POS | NEG | POS | NEG | NEG | NEG | NEG | NEG | NEG | POS | NEG | NEG | NEG | NEG | AMB | NEG | NEG | NEG | NEG | NEG | NEG | NEG | NEG | NEG | NEG |
| | NEG | NEG | NEG | AMB | AMB | NEG | POS | NEG | POS | NEG | NEG | NEG | NEG | NEG | NEG | POS | NEG | NEG | NEG | NEG | AMB | NEG | NEG | NEG | NEG | NEG | NEG | NEG | NEG | NEG | NEG |
| | NEG | NEG | NEG | NEG | NEG | NEG | POS | NEG | POS | NEG | NEG | NEG | NEG | NEG | NEG | POS | NEG | NEG | NEG | NEG | NEG | NEG | NEG | NEG | NEG | NEG | NEG | NEG | NEG | NEG | NEG |
| Jena_S0015_11 | NEG | NEG | NEG | NEG | NEG | NEG | POS | AMB | POS | NEG | NEG | NEG | NEG | NEG | NEG | POS | NEG | NEG | NEG | NEG | NEG | NEG | NEG | NEG | NEG | NEG | NEG | NEG | NEG | NEG | NEG |
| | NEG | NEG | NEG | NEG | AMB | NEG | POS | POS | POS | NEG | NEG | NEG | NEG | NEG | NEG | POS | NEG | NEG | NEG | NEG | NEG | NEG | NEG | NEG | NEG | NEG | NEG | NEG | NEG | NEG | NEG |
| | NEG | NEG | NEG | NEG | AMB | NEG | POS | POS | POS | NEG | NEG | NEG | NEG | NEG | NEG | POS | NEG | NEG | NEG | NEG | NEG | NEG | NEG | NEG | NEG | NEG | NEG | NEG | NEG | NEG | NEG |
| Jena_S0012_11 | NEG | NEG | NEG | NEG | NEG | NEG | POS | NEG | POS | NEG | NEG | NEG | NEG | NEG | NEG | POS | NEG | NEG | NEG | NEG | NEG | NEG | NEG | NEG | NEG | NEG | NEG | NEG | NEG | NEG | NEG |
| | NEG | NEG | NEG | NEG | NEG | NEG | POS | NEG | POS | NEG | NEG | NEG | NEG | NEG | NEG | POS | NEG | NEG | NEG | NEG | NEG | NEG | NEG | NEG | NEG | NEG | NEG | NEG | NEG | NEG | NEG |
| Dresden 07_7363 | NEG | NEG | NEG | NEG | AMB | NEG | POS | POS | POS | NEG | POS | NEG | NEG | NEG | NEG | POS | NEG | NEG | NEG | NEG | NEG | NEG | NEG | NEG | NEG | NEG | NEG | NEG | NEG | NEG | NEG |
| Dresden 07_8174 | NEG | NEG | NEG | NEG | NEG | NEG | POS | NEG | AMB | NEG | NEG | NEG | NEG | NEG | NEG | POS | NEG | NEG | NEG | NEG | NEG | NEG | NEG | NEG | NEG | NEG | NEG | NEG | NEG | NEG | NEG |
| Dresden 07_8224 | NEG | NEG | NEG | NEG | NEG | NEG | POS | NEG | AMB | NEG | AMB | NEG | NEG | NEG | NEG | POS | NEG | NEG | NEG | NEG | NEG | NEG | NEG | NEG | NEG | NEG | NEG | NEG | NEG | NEG | NEG |
| Dresden 07_8562 | NEG | NEG | NEG | NEG | NEG | NEG | POS | AMB | POS | NEG | POS | NEG | NEG | NEG | NEG | POS | NEG | NEG | NEG | NEG | NEG | NEG | NEG | NEG | NEG | NEG | NEG | NEG | NEG | NEG | NEG |
| Dresden 08_613 | NEG | NEG | NEG | NEG | NEG | NEG | POS | AMB | POS | NEG | AMB | NEG | NEG | NEG | NEG | POS | NEG | NEG | NEG | NEG | NEG | NEG | NEG | NEG | NEG | NEG | NEG | NEG | NEG | NEG | NEG |
| Dresden 08_0422 | NEG | NEG | NEG | NEG | AMB | NEG | POS | AMB | POS | NEG | NEG | NEG | NEG | NEG | NEG | POS | NEG | NEG | NEG | NEG | NEG | NEG | NEG | NEG | NEG | NEG | NEG | NEG | NEG | NEG | NEG |
| Dresden 08_0837 | NEG | NEG | NEG | NEG | NEG | NEG | POS | POS | POS | NEG | NEG | NEG | NEG | NEG | NEG | POS | NEG | NEG | NEG | NEG | NEG | NEG | NEG | NEG | NEG | NEG | NEG | NEG | NEG | NEG | NEG |
| Dresden 08_0878 | NEG | NEG | NEG | NEG | NEG | NEG | POS | NEG | AMB | NEG | NEG | NEG | NEG | NEG | NEG | POS | NEG | NEG | NEG | NEG | NEG | NEG | NEG | NEG | NEG | NEG | NEG | NEG | NEG | NEG | NEG |
| Dresden 08_1484 | NEG | NEG | NEG | NEG | NEG | NEG | POS | POS | POS | NEG | NEG | NEG | NEG | NEG | NEG | POS | NEG | NEG | NEG | NEG | NEG | NEG | NEG | NEG | NEG | NEG | NEG | NEG | NEG | NEG | NEG |
| **>HP-10** | NEG | NEG | NEG | NEG | NEG | NEG | POS | AMB | POS | NEG | NEG | NEG | NEG | NEG | NEG | NEG | NEG | NEG | NEG | NEG | NEG | NEG | POS | POS | NEG | NEG | NEG | NEG | NEG | NEG | NEG |
| Dresden 09_664_1 | NEG | NEG | NEG | NEG | NEG | NEG | POS | AMB | POS | NEG | NEG | NEG | NEG | NEG | NEG | NEG | NEG | NEG | NEG | NEG | NEG | NEG | POS | POS | NEG | NEG | NEG | NEG | NEG | NEG | NEG |
| Dresden 09_664_2 | NEG | NEG | NEG | NEG | NEG | NEG | POS | NEG | POS | NEG | NEG | NEG | NEG | NEG | NEG | NEG | NEG | NEG | NEG | NEG | NEG | NEG | POS | POS | NEG | NEG | NEG | NEG | NEG | NEG | NEG |
| Dresden 09_711_1 | NEG | NEG | NEG | NEG | NEG | NEG | POS | NEG | POS | NEG | NEG | NEG | NEG | NEG | NEG | NEG | NEG | NEG | NEG | NEG | NEG | NEG | POS | POS | NEG | NEG | NEG | NEG | NEG | NEG | NEG |
| Dresden 09_711_2 | NEG | NEG | NEG | NEG | NEG | NEG | POS | NEG | AMB | NEG | NEG | NEG | NEG | NEG | NEG | NEG | NEG | NEG | NEG | NEG | NEG | NEG | POS | POS | NEG | NEG | NEG | NEG | NEG | NEG | NEG |
| Dresden 09_932 | NEG | NEG | NEG | NEG | AMB | NEG | POS | POS | POS | NEG | NEG | NEG | NEG | NEG | NEG | NEG | NEG | NEG | NEG | NEG | NEG | NEG | POS | POS | NEG | NEG | NEG | NEG | NEG | NEG | NEG |
| Dresden 09_939 | NEG | NEG | NEG | NEG | NEG | NEG | POS | POS | POS | NEG | NEG | NEG | NEG | NEG | NEG | NEG | NEG | NEG | NEG | NEG | NEG | NEG | POS | POS | NEG | NEG | NEG | NEG | NEG | NEG | NEG |
| Dresden 09_948 | NEG | NEG | NEG | NEG | NEG | NEG | POS | POS | POS | NEG | NEG | NEG | NEG | NEG | NEG | NEG | NEG | NEG | NEG | NEG | NEG | NEG | POS | POS | NEG | NEG | NEG | NEG | NEG | NEG | NEG |
| **>HP-11** | NEG | NEG | NEG | NEG | AMB | NEG | POS | POS | POS | NEG | AMB | NEG | NEG | NEG | NEG | NEG | NEG | NEG | NEG | NEG | NEG | NEG | POS | POS | NEG | NEG | NEG | NEG | NEG | NEG | NEG |
| C. difficile CD37 (GenBank AHJJ) in silico prediction | NEG | NEG | NEG | NEG | NEG | NEG | POS | POS | POS | NEG | POS | NEG | NEG | NEG | NEG | NEG | NEG | NEG | NEG | NEG | NEG | NEG | POS | POS | NEG | NEG | NEG | NEG | NEG | NEG | NEG |
| Freiburg-684-10.684-DBK0000240 | NEG | NEG | NEG | AMB | AMB | NEG | POS | POS | POS | NEG | POS | NEG | NEG | NEG | NEG | NEG | NEG | NEG | NEG | NEG | NEG | NEG | POS | POS | NEG | NEG | NEG | NEG | NEG | NEG | NEG |
| Freiburg-838-28.838-DBK0000278 | NEG | NEG | NEG | AMB | AMB | NEG | POS | POS | POS | NEG | NEG | NEG | NEG | NEG | NEG | NEG | NEG | NEG | NEG | NEG | NEG | NEG | POS | POS | NEG | NEG | NEG | NEG | NEG | NEG | NEG |
| | NEG | NEG | NEG | NEG | NEG | NEG | POS | AMB | POS | NEG | NEG | NEG | NEG | NEG | NEG | NEG | NEG | NEG | NEG | NEG | NEG | NEG | POS | POS | NEG | NEG | NEG | NEG | NEG | NEG | NEG |
| **>HP-12** | NEG | NEG | NEG | NEG | AMB | NEG | POS | AMB | POS | NEG | POS | NEG | NEG | NEG | NEG | NEG | NEG | NEG | NEG | NEG | NEG | NEG | NEG | NEG | NEG | NEG | NEG | NEG | NEG | NEG | NEG |
| Freiburg-1007-68.1007-DBK0000252 | NEG | NEG | NEG | NEG | POS | AMB | POS | POS | POS | NEG | POS | NEG | NEG | NEG | NEG | NEG | NEG | NEG | NEG | NEG | NEG | NEG | NEG | NEG | NEG | NEG | NEG | NEG | NEG | NEG | NEG |
| | NEG | NEG | NEG | NEG | NEG | NEG | POS | NEG | POS | NEG | NEG | NEG | NEG | NEG | NEG | NEG | NEG | NEG | NEG | NEG | NEG | NEG | NEG | NEG | NEG | NEG | NEG | NEG | NEG | NEG | NEG |
| Freiburg-983-59.983-DBK0000256 | NEG | NEG | NEG | AMB | POS | AMB | POS | POS | POS | NEG | POS | NEG | NEG | NEG | NEG | NEG | NEG | NEG | NEG | NEG | NEG | NEG | NEG | NEG | NEG | NEG | NEG | NEG | NEG | NEG | NEG |
| | NEG | NEG | NEG | NEG | NEG | NEG | POS | NEG | POS | NEG | NEG | NEG | NEG | NEG | NEG | NEG | NEG | NEG | NEG | NEG | NEG | NEG | NEG | NEG | NEG | NEG | NEG | NEG | NEG | NEG | NEG |
| **>HP-13** | NEG | NEG | NEG | NEG | AMB | NEG | POS | POS | POS | NEG | NEG | NEG | NEG | NEG | NEG | NEG | NEG | NEG | NEG | NEG | NEG | NEG | NEG | NEG | NEG | NEG | NEG | NEG | NEG | NEG | NEG |
| C. difficile 70-100-2010 (GenBank AGAC) in silico prediction | NEG | NEG | NEG | NEG | NEG | NEG | POS | POS | POS | NEG | NEG | NEG | NEG | NEG | NEG | NEG | NEG | NEG | NEG | NEG | NEG | NEG | NEG | NEG | NEG | NEG | NEG | NEG | NEG | NEG | NEG |
| Freiburg-1044-75.1044-DBK0000270 | NEG | NEG | NEG | POS | POS | NEG | POS | POS | POS | NEG | NEG | NEG | NEG | NEG | NEG | NEG | NEG | NEG | NEG | NEG | NEG | NEG | NEG | NEG | NEG | NEG | NEG | NEG | NEG | NEG | NEG |
| | NEG | NEG | NEG | NEG | NEG | NEG | POS | POS | POS | NEG | NEG | NEG | NEG | NEG | NEG | NEG | NEG | NEG | NEG | NEG | NEG | NEG | NEG | NEG | NEG | NEG | NEG | NEG | NEG | NEG | NEG |
| Freiburg-1057-80.1057-DBK0000260 | NEG | NEG | NEG | POS | POS | AMB | POS | POS | POS | NEG | NEG | NEG | NEG | NEG | NEG | NEG | NEG | NEG | NEG | NEG | NEG | NEG | NEG | NEG | NEG | NEG | NEG | NEG | NEG | NEG | NEG |
| | NEG | NEG | NEG | AMB | NEG | NEG | POS | POS | POS | NEG | NEG | NEG | NEG | NEG | NEG | NEG | NEG | NEG | NEG | NEG | NEG | NEG | NEG | NEG | NEG | NEG | NEG | NEG | NEG | NEG | NEG |
| Freiburg-710-12.710-DBK0000241 | NEG | NEG | NEG | NEG | AMB | NEG | POS | POS | POS | NEG | NEG | NEG | NEG | NEG | NEG | NEG | NEG | NEG | NEG | NEG | NEG | NEG | NEG | NEG | NEG | NEG | NEG | NEG | NEG | NEG | NEG |
| | NEG | NEG | NEG | NEG | AMB | NEG | POS | POS | POS | NEG | NEG | NEG | NEG | NEG | NEG | NEG | NEG | NEG | NEG | NEG | NEG | NEG | NEG | NEG | NEG | NEG | NEG | NEG | NEG | NEG | NEG |
| Freiburg-940-48.94-DBK0000244 | NEG | NEG | NEG | NEG | AMB | NEG | POS | POS | POS | NEG | NEG | NEG | NEG | NEG | NEG | NEG | NEG | NEG | NEG | NEG | NEG | NEG | NEG | NEG | NEG | NEG | NEG | NEG | NEG | NEG | NEG |
| | NEG | NEG | NEG | NEG | NEG | NEG | POS | POS | POS | NEG | NEG | NEG | NEG | NEG | NEG | NEG | NEG | NEG | NEG | NEG | NEG | NEG | NEG | NEG | NEG | NEG | NEG | NEG | NEG | NEG | NEG |
| Freiburg-980-56.98-DBK0000246 | NEG | NEG | NEG | POS | POS | AMB | POS | POS | POS | NEG | POS | NEG | NEG | NEG | NEG | NEG | NEG | NEG | NEG | NEG | NEG | NEG | NEG | NEG | NEG | NEG | NEG | NEG | NEG | NEG | NEG |
| | NEG | NEG | NEG | NEG | NEG | NEG | POS | AMB | POS | NEG | NEG | NEG | NEG | NEG | NEG | NEG | NEG | NEG | NEG | NEG | NEG | NEG | NEG | NEG | NEG | NEG | NEG | NEG | NEG | NEG | NEG |
| Jena_S0014_11 | NEG | NEG | NEG | NEG | NEG | NEG | POS | AMB | POS | NEG | NEG | NEG | NEG | NEG | NEG | NEG | NEG | NEG | NEG | NEG | NEG | NEG | NEG | NEG | NEG | NEG | NEG | NEG | NEG | NEG | NEG |
| | NEG | NEG | NEG | AMB | AMB | NEG | POS | POS | POS | NEG | AMB | NEG | NEG | NEG | NEG | NEG | NEG | NEG | NEG | NEG | NEG | NEG | NEG | NEG | NEG | NEG | NEG | NEG | NEG | NEG | NEG |
| | NEG | NEG | NEG | NEG | NEG | NEG | POS | AMB | POS | NEG | NEG | NEG | NEG | NEG | NEG | NEG | NEG | NEG | NEG | NEG | NEG | NEG | NEG | NEG | NEG | NEG | NEG | NEG | NEG | NEG | NEG |
| Jena_S0023_11 | NEG | NEG | NEG | NEG | NEG | NEG | POS | AMB | POS | NEG | NEG | NEG | NEG | NEG | NEG | NEG | NEG | NEG | NEG | NEG | NEG | NEG | NEG | NEG | NEG | NEG | NEG | NEG | NEG | NEG | NEG |
| | NEG | NEG | NEG | NEG | AMB | NEG | POS | AMB | POS | NEG | NEG | NEG | NEG | NEG | NEG | NEG | NEG | NEG | NEG | NEG | NEG | NEG | NEG | NEG | NEG | NEG | NEG | NEG | NEG | NEG | NEG |
| | NEG | NEG | NEG | NEG | NEG | NEG | POS | NEG | AMB | NEG | NEG | NEG | NEG | NEG | NEG | NEG | NEG | NEG | NEG | NEG | NEG | NEG | NEG | NEG | NEG | NEG | NEG | NEG | NEG | NEG | NEG |
| Dresden 07_7779 | NEG | NEG | NEG | NEG | NEG | NEG | POS | NEG | NEG | NEG | AMB | NEG | NEG | NEG | NEG | NEG | NEG | NEG | NEG | NEG | NEG | NEG | NEG | NEG | NEG | NEG | NEG | NEG | NEG | NEG | NEG |
| Dresden 07_7950 | NEG | NEG | NEG | POS | POS | NEG | POS | POS | POS | NEG | NEG | NEG | NEG | NEG | NEG | NEG | NEG | NEG | NEG | NEG | NEG | NEG | NEG | NEG | NEG | NEG | NEG | NEG | NEG | NEG | NEG |
| Dresden 07_7953 | NEG | NEG | AMB | NEG | AMB | NEG | POS | AMB | POS | NEG | POS | NEG | POS | NEG | NEG | NEG | NEG | NEG | NEG | NEG | NEG | NEG | NEG | NEG | NEG | NEG | NEG | NEG | NEG | NEG | NEG |
| Dresden 07_8015 | NEG | NEG | NEG | AMB | POS | NEG | POS | AMB | POS | NEG | NEG | NEG | NEG | NEG | NEG | NEG | NEG | NEG | NEG | NEG | NEG | NEG | NEG | NEG | NEG | NEG | NEG | NEG | NEG | NEG | NEG |
| Dresden 07_8205 | NEG | NEG | NEG | AMB | POS | NEG | POS | NEG | POS | NEG | POS | NEG | NEG | NEG | NEG | NEG | NEG | NEG | NEG | NEG | NEG | NEG | NEG | NEG | NEG | NEG | NEG | NEG | NEG | NEG | NEG |
| Dresden 07_8595 | NEG | NEG | NEG | NEG | AMB | NEG | POS | POS | POS | NEG | NEG | NEG | NEG | NEG | NEG | NEG | NEG | NEG | NEG | NEG | NEG | NEG | NEG | NEG | NEG | NEG | NEG | NEG | NEG | NEG | NEG |
| Dresden 07_8613 | NEG | NEG | NEG | NEG | NEG | NEG | POS | NEG | NEG | NEG | NEG | NEG | NEG | NEG | NEG | NEG | NEG | NEG | NEG | NEG | NEG | NEG | NEG | NEG | NEG | NEG | NEG | NEG | NEG | NEG | NEG |
| Dresden 07_8686 | NEG | NEG | NEG | AMB | POS | NEG | POS | AMB | POS | NEG | POS | NEG | NEG | NEG | NEG | NEG | NEG | NEG | NEG | NEG | NEG | NEG | NEG | NEG | NEG | NEG | NEG | NEG | NEG | NEG | NEG |
| Dresden 07_9180 | NEG | NEG | NEG | NEG | AMB | NEG | POS | POS | POS | NEG | POS | NEG | NEG | NEG | NEG | NEG | NEG | NEG | NEG | NEG | NEG | NEG | NEG | NEG | NEG | NEG | NEG | NEG | NEG | NEG | NEG |
| Dresden 08_0237 | NEG | NEG | NEG | NEG | NEG | NEG | POS | NEG | POS | NEG | AMB | NEG | NEG | NEG | NEG | NEG | NEG | NEG | NEG | NEG | NEG | NEG | NEG | NEG | NEG | NEG | NEG | NEG | NEG | NEG | NEG |
| Dresden 08_0492 | NEG | NEG | NEG | NEG | NEG | NEG | POS | AMB | POS | NEG | NEG | NEG | NEG | NEG | NEG | NEG | NEG | NEG | NEG | NEG | NEG | NEG | NEG | NEG | NEG | NEG | NEG | NEG | NEG | NEG | NEG |
| Dresden 08_0992 | NEG | NEG | NEG | NEG | NEG | NEG | POS | POS | POS | NEG | NEG | NEG | NEG | NEG | NEG | NEG | NEG | NEG | NEG | NEG | NEG | NEG | NEG | NEG | NEG | NEG | NEG | NEG | NEG | NEG | NEG |
| Dresden 08_1184 | NEG | NEG | NEG | POS | POS | AMB | POS | POS | POS | NEG | NEG | NEG | NEG | NEG | NEG | NEG | NEG | NEG | NEG | NEG | NEG | NEG | NEG | NEG | NEG | NEG | NEG | NEG | NEG | NEG | NEG |
| Dresden 08_1314 | NEG | NEG | NEG | NEG | NEG | NEG | POS | AMB | POS | NEG | NEG | NEG | NEG | NEG | NEG | NEG | NEG | NEG | NEG | NEG | NEG | NEG | NEG | NEG | NEG | NEG | NEG | NEG | NEG | NEG | NEG |
| Dresden 08_1640 | NEG | NEG | NEG | NEG | NEG | NEG | POS | NEG | POS | NEG | NEG | NEG | NEG | NEG | NEG | NEG | NEG | NEG | NEG | NEG | NEG | NEG | NEG | NEG | NEG | NEG | NEG | NEG | NEG | NEG | NEG |
| Dresden 08_1640 | NEG | NEG | NEG | NEG | NEG | NEG | POS | NEG | POS | NEG | NEG | NEG | NEG | NEG | NEG | NEG | NEG | NEG | NEG | NEG | NEG | NEG | NEG | NEG | NEG | NEG | NEG | NEG | NEG | NEG | NEG |
| Dresden 08_1758 | NEG | NEG | NEG | NEG | NEG | NEG | POS | NEG | POS | NEG | AMB | NEG | NEG | NEG | NEG | NEG | NEG | NEG | NEG | NEG | NEG | NEG | NEG | NEG | NEG | NEG | NEG | NEG | NEG | NEG | NEG |
| **>HP-14** | NEG | NEG | NEG | AMB | POS | NEG | POS | POS | POS | NEG | NEG | NEG | NEG | NEG | NEG | NEG | NEG | POS | NEG | NEG | NEG | NEG | NEG | NEG | NEG | NEG | NEG | NEG | NEG | NEG | NEG |
| Freiburg-1019-70.1019-DBK0000255 | NEG | NEG | NEG | AMB | POS | NEG | POS | POS | POS | NEG | NEG | NEG | NEG | NEG | NEG | NEG | NEG | POS | NEG | NEG | NEG | NEG | NEG | NEG | NEG | NEG | NEG | NEG | NEG | NEG | NEG |
| **>HP-15** | NEG | NEG | NEG | NEG | NEG | NEG | POS | POS | POS | NEG | NEG | NEG | NEG | NEG | NEG | NEG | NEG | POS | NEG | NEG | NEG | NEG | NEG | NEG | NEG | NEG | NEG | NEG | NEG | NEG | NEG |
| Freiburg_42.907 | NEG | NEG | NEG | NEG | NEG | NEG | POS | POS | POS | NEG | NEG | NEG | NEG | NEG | NEG | NEG | NEG | POS | NEG | NEG | NEG | NEG | NEG | NEG | NEG | NEG | NEG | NEG | NEG | NEG | NEG |
| **>HP-16** | NEG | NEG | NEG | AMB | AMB | NEG | POS | AMB | POS | NEG | NEG | NEG | NEG | NEG | NEG | NEG | NEG | NEG | NEG | NEG | NEG | NEG | NEG | NEG | NEG | NEG | NEG | NEG | NEG | NEG | NEG |
| Freiburg-978-55.978-DBK0000259 | NEG | NEG | NEG | POS | POS | AMB | POS | POS | POS | NEG | NEG | NEG | NEG | NEG | NEG | NEG | NEG | NEG | NEG | NEG | NEG | NEG | NEG | NEG | NEG | NEG | NEG | NEG | NEG | NEG | NEG |
| | NEG | NEG | NEG | NEG | NEG | NEG | POS | AMB | POS | NEG | NEG | NEG | NEG | NEG | NEG | NEG | NEG | NEG | NEG | NEG | NEG | NEG | NEG | NEG | NEG | NEG | NEG | NEG | NEG | NEG | NEG |
| Dresden 08_1810 | NEG | NEG | NEG | NEG | NEG | NEG | POS | NEG | AMB | NEG | NEG | NEG | NEG | NEG | NEG | NEG | NEG | NEG | NEG | NEG | NEG | NEG | NEG | NEG | NEG | NEG | NEG | NEG | NEG | NEG | NEG |
| **>HP-17** | NEG | NEG | NEG | AMB | AMB | NEG | POS | AMB | POS | NEG | NEG | NEG | NEG | NEG | NEG | NEG | NEG | NEG | NEG | NEG | NEG | NEG | NEG | NEG | NEG | NEG | NEG | NEG | NEG | NEG | NEG |
| Freiburg-729-15.729-DBK0000226 | NEG | NEG | NEG | NEG | NEG | NEG | POS | NEG | NEG | NEG | NEG | NEG | NEG | NEG | NEG | NEG | NEG | NEG | NEG | NEG | NEG | NEG | NEG | NEG | NEG | NEG | NEG | NEG | NEG | NEG | NEG |
| | NEG | NEG | NEG | NEG | NEG | NEG | POS | NEG | AMB | NEG | NEG | NEG | NEG | NEG | NEG | NEG | NEG | NEG | NEG | NEG | NEG | NEG | NEG | NEG | NEG | NEG | NEG | NEG | NEG | NEG | NEG |
| Freiburg-926-45.926-DBK0000236 | NEG | NEG | NEG | POS | POS | AMB | POS | POS | POS | NEG | NEG | NEG | NEG | NEG | NEG | NEG | NEG | NEG | NEG | NEG | NEG | NEG | NEG | NEG | NEG | NEG | NEG | NEG | NEG | NEG | NEG |
| | NEG | NEG | NEG | AMB | AMB | NEG | POS | POS | POS | NEG | NEG | NEG | NEG | NEG | NEG | NEG | NEG | NEG | NEG | NEG | NEG | NEG | NEG | NEG | NEG | NEG | NEG | NEG | NEG | NEG | NEG |
| **>HP-18** | NEG | NEG | NEG | AMB | AMB | NEG | POS | AMB | POS | NEG | NEG | NEG | NEG | NEG | NEG | NEG | NEG | NEG | NEG | NEG | NEG | NEG | NEG | NEG | NEG | NEG | NEG | NEG | NEG | NEG | POS |
| Freiburg-651-7.651-DBK0000229 | NEG | NEG | NEG | POS | POS | AMB | POS | POS | POS | NEG | NEG | NEG | NEG | NEG | NEG | NEG | NEG | NEG | NEG | NEG | NEG | NEG | NEG | NEG | NEG | NEG | NEG | NEG | NEG | NEG | POS |
| | NEG | NEG | NEG | NEG | NEG | NEG | POS | POS | POS | NEG | NEG | NEG | NEG | NEG | NEG | NEG | NEG | NEG | NEG | NEG | NEG | NEG | NEG | NEG | NEG | NEG | NEG | NEG | NEG | NEG | POS |
| Freiburg-725-13.725-DBK0000227 | NEG | NEG | NEG | NEG | NEG | NEG | POS | NEG | POS | NEG | NEG | NEG | NEG | NEG | NEG | NEG | NEG | NEG | NEG | NEG | NEG | NEG | NEG | NEG | NEG | NEG | NEG | NEG | NEG | NEG | POS |
| | NEG | NEG | NEG | NEG | NEG | NEG | POS | NEG | AMB | NEG | NEG | NEG | NEG | NEG | NEG | NEG | NEG | NEG | NEG | NEG | NEG | NEG | NEG | NEG | NEG | NEG | NEG | NEG | NEG | NEG | POS |
| Dresden 07_7176 | NEG | NEG | NEG | NEG | NEG | NEG | NEG | NEG | NEG | NEG | NEG | NEG | NEG | NEG | NEG | NEG | NEG | NEG | NEG | NEG | NEG | NEG | NEG | NEG | NEG | NEG | NEG | NEG | NEG | NEG | POS |
| Dresden 07_7209 | NEG | NEG | NEG | POS | POS | AMB | POS | POS | POS | NEG | NEG | NEG | NEG | NEG | NEG | NEG | NEG | NEG | NEG | NEG | NEG | NEG | NEG | NEG | NEG | NEG | NEG | NEG | NEG | NEG | POS |
| Dresden 07_8029 | NEG | NEG | NEG | NEG | AMB | NEG | POS | AMB | POS | NEG | POS | NEG | NEG | NEG | NEG | NEG | NEG | NEG | NEG | NEG | NEG | NEG | NEG | NEG | NEG | NEG | NEG | NEG | NEG | NEG | POS |
| Dresden 07_8575 | NEG | NEG | NEG | NEG | POS | NEG | POS | AMB | POS | NEG | NEG | NEG | NEG | NEG | NEG | NEG | NEG | NEG | NEG | NEG | NEG | NEG | NEG | NEG | NEG | NEG | NEG | NEG | NEG | NEG | POS |
| Dresden 08_1746 | NEG | NEG | NEG | NEG | NEG | NEG | POS | AMB | POS | NEG | NEG | NEG | NEG | NEG | NEG | NEG | NEG | NEG | NEG | NEG | NEG | NEG | NEG | NEG | NEG | NEG | NEG | NEG | NEG | NEG | POS |
| **>HP-19** | NEG | NEG | NEG | AMB | AMB | NEG | POS | POS | POS | NEG | NEG | NEG | NEG | NEG | NEG | NEG | NEG | NEG | NEG | NEG | NEG | NEG | NEG | NEG | NEG | NEG | NEG | POS | NEG | NEG | NEG |
| Freiburg-894-37.894-DBK0000289 | NEG | NEG | NEG | POS | POS | NEG | POS | POS | POS | NEG | NEG | NEG | NEG | NEG | NEG | NEG | NEG | NEG | NEG | NEG | NEG | NEG | NEG | NEG | NEG | NEG | NEG | POS | NEG | NEG | NEG |
| | NEG | NEG | NEG | NEG | NEG | NEG | POS | AMB | POS | NEG | NEG | NEG | NEG | NEG | NEG | NEG | NEG | NEG | NEG | NEG | NEG | NEG | NEG | NEG | NEG | NEG | NEG | POS | NEG | NEG | NEG |
| Freiburg-1000-67.1-DBK0000263 | NEG | NEG | NEG | POS | POS | AMB | POS | POS | POS | NEG | NEG | NEG | NEG | NEG | NEG | NEG | NEG | NEG | NEG | NEG | NEG | NEG | NEG | NEG | NEG | NEG | NEG | POS | NEG | NEG | NEG |
| | NEG | NEG | NEG | NEG | AMB | NEG | POS | POS | POS | NEG | NEG | NEG | NEG | NEG | NEG | NEG | NEG | NEG | NEG | NEG | NEG | NEG | NEG | NEG | NEG | NEG | NEG | POS | NEG | NEG | NEG |
| Dresden 07_7828 | NEG | NEG | NEG | NEG | NEG | NEG | POS | AMB | POS | NEG | NEG | NEG | NEG | NEG | NEG | NEG | NEG | NEG | NEG | NEG | NEG | NEG | NEG | NEG | NEG | NEG | NEG | POS | NEG | NEG | NEG |
| Dresden 08_1128 | NEG | NEG | NEG | NEG | NEG | NEG | POS | AMB | POS | NEG | NEG | NEG | NEG | NEG | NEG | NEG | NEG | NEG | NEG | NEG | NEG | NEG | NEG | NEG | NEG | NEG | NEG | POS | NEG | NEG | NEG |

| STRAIN / ISOLATE | putative endonuclease relaxase Tn1549-like | | | | | | putative calcium-binding adhesion protein | | ethanolamine iron-dependent alcohol dehydrogenase | | leucine Rich repeat-containing domain protein |
|---|---|---|---|---|---|---|---|---|---|---|---|
| | hp1214\|CD0425 | hp1220\|CD0425 | hp1228\|CD0425 | hp1229\|CD0425 | hp1230\|CD0425 | hp1231\|CD0425 | hp1149\|CD2797 | hp1212\|CD2797 | hp1061\|eutG | hp1062\|eutG | hp1227\|IPR007253 |
| **>HP-20** | NEG | NEG | NEG | AMB | AMB | NEG | POS | NEG | POS | NEG | NEG |
| Freiburg-963-50.963-DBK0000243 | NEG | NEG | NEG | POS | POS | NEG | POS | POS | POS | NEG | NEG |
| Freiburg-750-18.75-DBK0000232 | NEG | NEG | NEG | POS | POS | NEG | POS | AMB | POS | NEG | NEG |
| | NEG | NEG | NEG | NEG | NEG | NEG | NEG | NEG | AMB | NEG | NEG |
| Freiburg-637-4.637-DBK0000280 | NEG | NEG | NEG | POS | POS | AMB | POS | POS | POS | NEG | NEG |
| | NEG | NEG | NEG | NEG | NEG | NEG | POS | NEG | POS | NEG | NEG |
| Freiburg-856-31.856-DBK0000273 | NEG | NEG | NEG | AMB | POS | NEG | POS | NEG | POS | NEG | POS |
| | NEG | NEG | NEG | AMB | AMB | NEG | POS | NEG | POS | NEG | AMB |
| Freiburg_51.956 | NEG | NEG | NEG | NEG | NEG | NEG | POS | POS | POS | NEG | NEG |
| Dresden 07_7984 | NEG | NEG | NEG | NEG | AMB | NEG | POS | NEG | POS | NEG | NEG |
| Dresden 07_8203 | NEG | NEG | NEG | AMB | POS | AMB | POS | NEG | POS | NEG | NEG |
| Dresden 07_8398 | NEG | NEG | NEG | NEG | NEG | NEG | AMB | NEG | AMB | NEG | NEG |
| Dresden 07_8798 | NEG | NEG | NEG | NEG | AMB | NEG | POS | NEG | POS | NEG | NEG |
| **>HP-21** | NEG | NEG | NEG | POS | POS | NEG | POS | POS | POS | NEG | NEG |
| Freiburg-823-26.823-DBK0000283 | NEG | NEG | NEG | POS | POS | AMB | POS | POS | POS | NEG | NEG |
| | NEG | NEG | NEG | AMB | AMB | NEG | POS | POS | POS | NEG | NEG |
| Freiburg-952-49.952-DBK0000245 | NEG | NEG | NEG | AMB | AMB | NEG | POS | POS | POS | NEG | NEG |
| | NEG | NEG | NEG | POS | POS | AMB | POS | POS | POS | NEG | NEG |
| **>HP-22** | NEG | NEG | NEG | POS | AMB | NEG | POS | POS | POS | NEG | NEG |
| Dresden 08_0288 | NEG | NEG | NEG | POS | AMB | NEG | POS | POS | POS | NEG | NEG |
| **>HP-23** | NEG | NEG | NEG | POS | AMB | NEG | POS | POS | POS | NEG | NEG |
| Freiburg-786-21.786-DBK0000275 | NEG | NEG | NEG | POS | AMB | NEG | POS | POS | POS | NEG | NEG |
| | NEG | NEG | NEG | POS | NEG | NEG | POS | AMB | POS | NEG | NEG |
| **>HP-24** | NEG | NEG | NEG | POS | AMB | POS | POS | AMB | POS | NEG | NEG |
| C. difficile 6534 (GenBank ADEJ) in silico prediction | NEG | NEG | NEG | POS | NEG | POS | POS | POS | POS | NEG | NEG |
| C. difficile 630 (GenBank AM180355) in silico prediction | NEG | NEG | NEG | POS | NEG | POS | POS | POS | POS | NEG | NEG |
| C. difficile Strain 630 | NEG | NEG | NEG | POS | AMB | POS | POS | NEG | POS | NEG | NEG |
| | NEG | NEG | NEG | POS | POS | POS | POS | NEG | POS | NEG | NEG |
| | NEG | NEG | NEG | POS | NEG | POS | POS | NEG | POS | NEG | NEG |
| | NEG | NEG | NEG | POS | NEG | POS | POS | AMB | POS | NEG | NEG |
| | NEG | NEG | NEG | POS | AMB | POS | POS | POS | POS | NEG | NEG |
| | NEG | NEG | NEG | POS | NEG | POS | POS | NEG | AMB | NEG | NEG |
| Dresden 07_7156 | NEG | NEG | NEG | POS | NEG | AMB | POS | NEG | NEG | NEG | NEG |
| Dresden 07_7158 | NEG | NEG | NEG | POS | AMB | POS | POS | AMB | POS | NEG | NEG |
| Dresden 07_7203 | NEG | NEG | NEG | POS | AMB | POS | POS | POS | POS | NEG | NEG |
| Dresden 07_8217 | NEG | NEG | NEG | POS | NEG | POS | POS | NEG | POS | NEG | NEG |
| Dresden 07_8270 | NEG | NEG | NEG | POS | NEG | POS | POS | AMB | POS | NEG | NEG |
| Dresden 07_8749 | NEG | NEG | NEG | AMB | NEG | AMB | POS | NEG | NEG | NEG | NEG |
| Dresden 07_8788 | NEG | NEG | NEG | POS | POS | POS | POS | AMB | POS | NEG | NEG |
| Dresden 07_9245 | NEG | NEG | NEG | POS | NEG | POS | POS | AMB | POS | NEG | NEG |
| Dresden 08_0217 | NEG | NEG | NEG | POS | AMB | POS | POS | POS | POS | NEG | NEG |
| Dresden 08_0219 | NEG | NEG | NEG | POS | AMB | POS | POS | POS | POS | NEG | NEG |
| Dresden 08_0419 | NEG | NEG | NEG | POS | AMB | POS | POS | AMB | POS | NEG | NEG |
| Dresden 08_0484 | NEG | NEG | NEG | POS | NEG | POS | POS | POS | POS | NEG | NEG |
| Dresden 08_0994 | NEG | NEG | NEG | POS | AMB | POS | POS | POS | POS | NEG | NEG |
| Dresden 08_1148 | NEG | NEG | NEG | POS | NEG | POS | POS | AMB | POS | NEG | NEG |
| Dresden 08_1188 | NEG | NEG | NEG | POS | NEG | POS | POS | POS | POS | NEG | NEG |
| Dresden 08_1205 | NEG | NEG | NEG | POS | NEG | POS | POS | AMB | POS | NEG | NEG |
| Dresden 08_1541 | NEG | NEG | NEG | POS | POS | POS | POS | POS | POS | NEG | NEG |
| Dresden 06_7896 | NEG | NEG | NEG | NEG | NEG | NEG | POS | NEG | NEG | NEG | NEG |
| **>HP-25** | AMB | NEG | NEG | NEG | NEG | NEG | POS | NEG | NEG | NEG | POS |
| Freiburg-809-24.809-DBK0000287 | AMB | NEG | NEG | NEG | NEG | NEG | POS | NEG | NEG | NEG | AMB |
| | NEG | NEG | NEG | NEG | NEG | NEG | POS | NEG | NEG | NEG | AMB |
| Freiburg-990-64.99-DBK0000264 | AMB | NEG | NEG | AMB | NEG | NEG | POS | NEG | NEG | NEG | POS |
| | NEG | NEG | NEG | NEG | NEG | NEG | POS | NEG | NEG | NEG | AMB |
| Dresden 07_7952 | POS | NEG | NEG | NEG | NEG | NEG | POS | NEG | NEG | NEG | POS |
| Dresden 07_8955 | NEG | NEG | NEG | NEG | NEG | NEG | POS | NEG | NEG | NEG | POS |
| Dresden 07_8958 | NEG | NEG | NEG | NEG | NEG | NEG | POS | NEG | NEG | NEG | POS |
| Dresden 07_9027 | POS | NEG | NEG | NEG | AMB | NEG | POS | NEG | NEG | NEG | POS |
| Dresden 08_1169 | NEG | NEG | NEG | NEG | NEG | NEG | POS | NEG | NEG | NEG | NEG |
| **>HP-26** | POS | NEG | NEG | AMB | AMB | NEG | POS | POS | POS | NEG | POS |
| Freiburg-635-3.635-DBK0000297 | POS | NEG | NEG | POS | POS | AMB | POS | POS | POS | NEG | POS |
| | POS | NEG | NEG | NEG | NEG | NEG | POS | POS | POS | NEG | NEG |
| Freiburg-895-38.895-DBK0000290 | POS | NEG | NEG | POS | POS | NEG | POS | POS | POS | NEG | POS |
| | POS | NEG | NEG | NEG | NEG | NEG | POS | POS | POS | NEG | NEG |
| **>HP-27** | NEG | NEG | NEG | NEG | AMB | NEG | POS | POS | POS | NEG | POS |
| Freiburg-726-14.726-DBK0000228 | NEG | NEG | NEG | AMB | AMB | AMB | POS | POS | POS | NEG | POS |
| | NEG | NEG | NEG | NEG | NEG | NEG | POS | POS | POS | NEG | POS |
| Freiburg-837-27.837-DBK0000285 | NEG | NEG | NEG | AMB | AMB | NEG | POS | POS | POS | NEG | POS |
| | NEG | NEG | NEG | NEG | NEG | NEG | POS | POS | POS | NEG | NEG |
| **>HP-28** | NEG | NEG | NEG | NEG | NEG | NEG | NEG | POS | POS | NEG | POS |
| C. difficile 6407 (GenBank ADEH) in silico prediction | NEG | NEG | NEG | NEG | NEG | NEG | NEG | POS | POS | NEG | POS |
| **>HP-29** | NEG | NEG | NEG | AMB | POS | NEG | POS | POS | POS | NEG | AMB |
| Freiburg-852-29.852-DBK0000279 | NEG | NEG | NEG | AMB | POS | NEG | POS | POS | POS | NEG | POS |
| | NEG | NEG | NEG | AMB | AMB | NEG | POS | AMB | POS | NEG | NEG |
| Freiburg-858-32.858-DBK0000222 | NEG | NEG | NEG | POS | POS | NEG | POS | POS | POS | NEG | POS |
| **>HP-30** | NEG | NEG | NEG | AMB | AMB | NEG | POS | POS | POS | NEG | AMB |
| Freiburg-1047-76.1047-DBK0000269 | NEG | NEG | NEG | AMB | POS | NEG | POS | POS | POS | NEG | POS |
| | NEG | NEG | NEG | NEG | NEG | NEG | POS | POS | POS | NEG | NEG |
| Freiburg-999-66.999-DBK0000262 | NEG | NEG | NEG | AMB | POS | NEG | POS | POS | POS | NEG | POS |
| | NEG | NEG | NEG | NEG | AMB | NEG | POS | POS | POS | NEG | AMB |
| Freiburg-896-39.896-DBK0000291 | NEG | NEG | NEG | POS | POS | NEG | POS | POS | POS | NEG | POS |
| | NEG | NEG | NEG | NEG | NEG | NEG | POS | POS | POS | NEG | NEG |
| Freiburg-871-33.871-DBK0000272 | NEG | NEG | NEG | NEG | AMB | NEG | POS | POS | POS | NEG | AMB |
| | NEG | NEG | NEG | NEG | NEG | NEG | POS | POS | POS | NEG | AMB |
| Dresden 08_1596 | NEG | NEG | NEG | NEG | NEG | NEG | POS | AMB | POS | NEG | NEG |

| STRAIN / ISOLATE | splA Surface layer protein A | | | | | | | | | | | | | | | | | | | |
|---|---|---|---|---|---|---|---|---|---|---|---|---|---|---|---|---|---|---|---|---|
| | hp1150\|slpA | hp1151\|slpA | hp1153\|slpA | hp1154\|slpA | hp1155\|slpA | hp1156\|slpA | hp1158\|slpA | hp1164\|slpA | hp1166\|slpA | hp1167\|slpA | hp1168\|slpA | hp1169\|slpA | hp1170\|slpA | hp1171\|slpA | hp1173\|slpA | hp1174\|slpA | hp1175\|slpA | hp1176\|slpA | hp1177\|slpA | hp1178\|slpA |
| **>HP-20** | NEG | NEG | NEG | NEG | NEG | POS | NEG | NEG | NEG | NEG | NEG | NEG | NEG | NEG | NEG | NEG | NEG | NEG | NEG | NEG |
| Freiburg-963-50.963-DBK0000243 | NEG | NEG | NEG | NEG | NEG | POS | NEG | NEG | NEG | NEG | NEG | NEG | NEG | NEG | NEG | NEG | NEG | NEG | NEG | NEG |
| Freiburg-750-18.75-DBK0000232 | NEG | NEG | NEG | NEG | NEG | POS | NEG | NEG | NEG | NEG | NEG | NEG | NEG | NEG | NEG | NEG | NEG | NEG | NEG | NEG |
| | NEG | NEG | NEG | NEG | NEG | POS | NEG | NEG | NEG | NEG | NEG | NEG | NEG | NEG | NEG | NEG | NEG | NEG | NEG | NEG |
| Freiburg-637-4.637-DBK0000280 | NEG | NEG | NEG | NEG | NEG | POS | NEG | NEG | NEG | NEG | NEG | NEG | NEG | NEG | NEG | NEG | NEG | NEG | NEG | NEG |
| | NEG | NEG | NEG | NEG | NEG | POS | NEG | NEG | NEG | NEG | NEG | NEG | NEG | NEG | NEG | NEG | NEG | NEG | NEG | NEG |
| Freiburg-856-31.856-DBK0000273 | NEG | NEG | NEG | NEG | NEG | POS | NEG | NEG | NEG | NEG | NEG | NEG | NEG | NEG | NEG | NEG | NEG | NEG | NEG | NEG |
| | NEG | NEG | NEG | NEG | NEG | POS | NEG | NEG | NEG | NEG | NEG | NEG | NEG | NEG | NEG | NEG | NEG | NEG | NEG | NEG |
| Freiburg_51.956 | NEG | NEG | NEG | NEG | NEG | POS | NEG | NEG | NEG | NEG | NEG | NEG | NEG | NEG | NEG | NEG | NEG | NEG | NEG | NEG |
| Dresden 07_7984 | NEG | NEG | NEG | NEG | NEG | POS | NEG | NEG | NEG | NEG | NEG | NEG | NEG | NEG | NEG | NEG | NEG | NEG | NEG | NEG |
| Dresden 07_8203 | NEG | NEG | NEG | NEG | NEG | POS | NEG | NEG | NEG | NEG | NEG | NEG | NEG | NEG | NEG | NEG | NEG | NEG | NEG | NEG |
| Dresden 07_8398 | NEG | NEG | NEG | NEG | NEG | POS | NEG | NEG | NEG | NEG | NEG | NEG | NEG | NEG | NEG | NEG | NEG | NEG | NEG | NEG |
| Dresden 07_8798 | NEG | NEG | NEG | NEG | NEG | POS | NEG | NEG | NEG | NEG | NEG | NEG | NEG | NEG | NEG | NEG | NEG | NEG | NEG | NEG |
| **>HP-21** | NEG | NEG | NEG | NEG | NEG | NEG | NEG | NEG | NEG | NEG | NEG | NEG | NEG | NEG | NEG | NEG | NEG | NEG | NEG | NEG |
| Freiburg-823-26.823-DBK0000283 | NEG | NEG | NEG | NEG | NEG | NEG | NEG | NEG | NEG | NEG | NEG | NEG | NEG | NEG | NEG | NEG | NEG | NEG | NEG | NEG |
| | NEG | NEG | NEG | NEG | NEG | NEG | NEG | NEG | NEG | NEG | NEG | NEG | NEG | NEG | NEG | NEG | NEG | NEG | NEG | NEG |
| Freiburg-952-49.952-DBK0000245 | NEG | NEG | NEG | NEG | NEG | NEG | NEG | NEG | NEG | NEG | NEG | NEG | NEG | NEG | NEG | NEG | NEG | NEG | NEG | NEG |
| | NEG | NEG | NEG | NEG | NEG | NEG | NEG | NEG | NEG | NEG | NEG | NEG | NEG | NEG | NEG | NEG | NEG | NEG | NEG | NEG |
| **>HP-22** | NEG | NEG | NEG | NEG | NEG | NEG | NEG | NEG | NEG | NEG | NEG | NEG | NEG | NEG | NEG | NEG | NEG | NEG | NEG | NEG |
| Dresden 08_0288 | NEG | NEG | NEG | NEG | NEG | NEG | NEG | NEG | NEG | NEG | NEG | NEG | NEG | NEG | NEG | NEG | NEG | NEG | NEG | NEG |
| **>HP-23** | NEG | NEG | NEG | NEG | NEG | NEG | NEG | NEG | NEG | NEG | NEG | NEG | NEG | NEG | NEG | NEG | NEG | NEG | NEG | NEG |
| Freiburg-786-21.786-DBK0000275 | NEG | NEG | NEG | NEG | NEG | NEG | NEG | NEG | NEG | NEG | NEG | NEG | NEG | NEG | NEG | NEG | NEG | NEG | NEG | NEG |
| | NEG | NEG | NEG | NEG | NEG | NEG | NEG | NEG | NEG | NEG | NEG | NEG | NEG | NEG | NEG | NEG | NEG | NEG | NEG | NEG |
| **>HP-24** | NEG | NEG | NEG | NEG | NEG | NEG | NEG | NEG | POS | NEG | NEG | NEG | NEG | NEG | NEG | NEG | NEG | NEG | NEG | NEG |
| C. difficile 6534 (GenBank ADEJ) in silico prediction | NEG | NEG | NEG | NEG | NEG | NEG | NEG | NEG | POS | NEG | NEG | NEG | NEG | NEG | NEG | NEG | NEG | NEG | NEG | NEG |
| C. difficile 630 (GenBank AM180355) in silico prediction | NEG | NEG | NEG | NEG | NEG | NEG | NEG | NEG | POS | NEG | NEG | NEG | NEG | NEG | NEG | NEG | NEG | NEG | NEG | NEG |
| C. difficile Strain 630 | NEG | NEG | NEG | NEG | NEG | NEG | NEG | NEG | POS | AMB | NEG | NEG | NEG | NEG | NEG | NEG | NEG | NEG | NEG | NEG |
| | NEG | NEG | NEG | NEG | NEG | NEG | NEG | NEG | POS | POS | NEG | NEG | NEG | NEG | NEG | NEG | NEG | NEG | NEG | NEG |
| | NEG | NEG | NEG | NEG | NEG | NEG | NEG | NEG | POS | NEG | NEG | NEG | NEG | NEG | NEG | NEG | NEG | NEG | NEG | NEG |
| | NEG | NEG | NEG | NEG | NEG | NEG | NEG | NEG | POS | NEG | NEG | NEG | NEG | NEG | NEG | NEG | NEG | NEG | NEG | NEG |
| | NEG | NEG | NEG | NEG | NEG | NEG | NEG | NEG | POS | NEG | NEG | NEG | NEG | NEG | NEG | NEG | NEG | NEG | NEG | NEG |
| | NEG | NEG | NEG | NEG | NEG | NEG | NEG | NEG | POS | NEG | NEG | NEG | NEG | NEG | NEG | NEG | NEG | NEG | NEG | NEG |
| Dresden 07_7156 | NEG | NEG | NEG | NEG | NEG | NEG | NEG | NEG | POS | NEG | NEG | NEG | NEG | NEG | NEG | NEG | NEG | NEG | NEG | NEG |
| Dresden 07_7158 | NEG | NEG | NEG | NEG | NEG | NEG | NEG | NEG | POS | NEG | NEG | NEG | NEG | NEG | NEG | NEG | NEG | NEG | NEG | NEG |
| Dresden 07_7203 | NEG | NEG | NEG | NEG | NEG | NEG | NEG | NEG | POS | NEG | NEG | NEG | NEG | NEG | NEG | NEG | NEG | NEG | NEG | NEG |
| Dresden 07_8217 | NEG | NEG | NEG | NEG | NEG | NEG | NEG | NEG | POS | NEG | NEG | NEG | NEG | NEG | NEG | NEG | NEG | NEG | NEG | NEG |
| Dresden 07_8270 | NEG | NEG | NEG | NEG | NEG | NEG | NEG | NEG | POS | NEG | NEG | NEG | NEG | NEG | NEG | NEG | NEG | NEG | NEG | NEG |
| Dresden 07_8749 | NEG | NEG | NEG | NEG | NEG | NEG | NEG | NEG | POS | NEG | NEG | NEG | NEG | NEG | NEG | NEG | NEG | NEG | NEG | NEG |
| Dresden 07_8788 | NEG | NEG | NEG | NEG | NEG | NEG | NEG | NEG | POS | NEG | NEG | NEG | NEG | NEG | NEG | NEG | NEG | NEG | NEG | NEG |
| Dresden 07_9245 | NEG | NEG | NEG | NEG | NEG | NEG | NEG | NEG | POS | NEG | NEG | NEG | NEG | NEG | NEG | NEG | NEG | NEG | NEG | NEG |
| Dresden 08_0217 | NEG | NEG | NEG | NEG | NEG | NEG | NEG | NEG | POS | NEG | NEG | NEG | NEG | NEG | NEG | NEG | NEG | NEG | NEG | NEG |
| Dresden 08_0219 | NEG | NEG | NEG | NEG | NEG | NEG | NEG | NEG | POS | NEG | NEG | NEG | NEG | NEG | NEG | NEG | NEG | NEG | NEG | NEG |
| Dresden 08_0419 | NEG | NEG | NEG | NEG | NEG | NEG | NEG | NEG | POS | NEG | NEG | NEG | NEG | NEG | NEG | NEG | NEG | NEG | NEG | NEG |
| Dresden 08_0484 | NEG | NEG | NEG | NEG | NEG | NEG | NEG | NEG | POS | NEG | NEG | NEG | NEG | NEG | NEG | NEG | NEG | NEG | NEG | NEG |
| Dresden 08_0994 | NEG | NEG | NEG | NEG | NEG | NEG | NEG | NEG | POS | NEG | NEG | NEG | NEG | NEG | NEG | NEG | NEG | NEG | NEG | NEG |
| Dresden 08_1148 | NEG | NEG | NEG | NEG | NEG | NEG | NEG | NEG | POS | NEG | NEG | NEG | NEG | NEG | NEG | NEG | NEG | NEG | NEG | NEG |
| Dresden 08_1188 | NEG | NEG | NEG | NEG | NEG | NEG | NEG | NEG | POS | NEG | NEG | NEG | NEG | NEG | NEG | NEG | NEG | NEG | NEG | NEG |
| Dresden 08_1205 | NEG | NEG | NEG | NEG | NEG | NEG | NEG | NEG | POS | NEG | NEG | NEG | NEG | NEG | NEG | NEG | NEG | NEG | NEG | NEG |
| Dresden 08_1541 | NEG | NEG | NEG | NEG | NEG | NEG | NEG | NEG | POS | NEG | NEG | NEG | NEG | NEG | NEG | NEG | NEG | NEG | NEG | NEG |
| Dresden 06_7896 | NEG | NEG | NEG | NEG | NEG | NEG | NEG | NEG | POS | NEG | NEG | NEG | NEG | NEG | NEG | NEG | NEG | NEG | NEG | NEG |
| **>HP-25** | NEG | NEG | NEG | NEG | NEG | NEG | NEG | NEG | NEG | NEG | NEG | NEG | NEG | NEG | POS | NEG | NEG | NEG | NEG | NEG |
| Freiburg-809-24.809-DBK0000287 | NEG | NEG | NEG | NEG | NEG | NEG | NEG | NEG | NEG | NEG | NEG | NEG | NEG | NEG | POS | NEG | NEG | NEG | NEG | NEG |
| | NEG | NEG | NEG | NEG | NEG | NEG | NEG | NEG | NEG | NEG | NEG | NEG | NEG | NEG | POS | NEG | NEG | NEG | NEG | NEG |
| Freiburg-990-64.99-DBK0000264 | NEG | NEG | NEG | NEG | NEG | NEG | NEG | NEG | NEG | NEG | NEG | NEG | NEG | NEG | POS | NEG | NEG | NEG | NEG | NEG |
| | NEG | NEG | NEG | NEG | NEG | NEG | NEG | NEG | NEG | NEG | NEG | NEG | NEG | NEG | POS | NEG | NEG | NEG | NEG | NEG |
| Dresden 07_7952 | NEG | NEG | NEG | NEG | NEG | NEG | NEG | NEG | NEG | NEG | NEG | NEG | NEG | NEG | POS | NEG | NEG | NEG | NEG | NEG |
| Dresden 07_8955 | NEG | NEG | NEG | NEG | NEG | NEG | NEG | NEG | NEG | NEG | NEG | NEG | NEG | NEG | POS | NEG | NEG | NEG | NEG | NEG |
| Dresden 07_8958 | NEG | NEG | NEG | NEG | NEG | NEG | NEG | NEG | NEG | NEG | NEG | NEG | NEG | NEG | POS | NEG | NEG | NEG | NEG | NEG |
| Dresden 07_9027 | NEG | NEG | NEG | NEG | NEG | NEG | NEG | NEG | NEG | NEG | NEG | NEG | NEG | NEG | POS | NEG | NEG | NEG | NEG | NEG |
| Dresden 08_1169 | NEG | NEG | NEG | NEG | NEG | NEG | NEG | NEG | NEG | NEG | NEG | NEG | NEG | NEG | POS | NEG | NEG | NEG | NEG | NEG |
| **>HP-26** | NEG | NEG | NEG | NEG | NEG | NEG | NEG | NEG | NEG | NEG | NEG | NEG | NEG | NEG | NEG | NEG | NEG | NEG | NEG | NEG |
| Freiburg-635-3.635-DBK0000297 | NEG | NEG | NEG | NEG | NEG | NEG | NEG | NEG | NEG | NEG | NEG | NEG | NEG | NEG | NEG | NEG | NEG | NEG | NEG | NEG |
| | NEG | NEG | NEG | NEG | NEG | NEG | NEG | NEG | NEG | NEG | NEG | NEG | NEG | NEG | NEG | NEG | NEG | NEG | NEG | NEG |
| Freiburg-895-38.895-DBK0000290 | NEG | NEG | NEG | NEG | NEG | NEG | NEG | NEG | NEG | NEG | NEG | NEG | NEG | NEG | NEG | NEG | NEG | NEG | NEG | NEG |
| | NEG | NEG | NEG | NEG | NEG | NEG | NEG | NEG | NEG | NEG | NEG | NEG | NEG | NEG | NEG | NEG | NEG | NEG | NEG | NEG |
| **>HP-27** | NEG | NEG | NEG | POS | NEG | NEG | NEG | NEG | NEG | NEG | NEG | NEG | NEG | NEG | NEG | NEG | NEG | NEG | NEG | NEG |
| Freiburg-726-14.726-DBK0000228 | NEG | NEG | NEG | POS | NEG | NEG | NEG | NEG | NEG | NEG | NEG | NEG | NEG | NEG | NEG | NEG | NEG | NEG | NEG | NEG |
| | NEG | NEG | NEG | POS | NEG | NEG | NEG | NEG | NEG | NEG | NEG | NEG | NEG | NEG | NEG | NEG | NEG | NEG | NEG | NEG |
| Freiburg-837-27.837-DBK0000285 | NEG | NEG | NEG | POS | NEG | NEG | NEG | NEG | NEG | NEG | NEG | NEG | NEG | NEG | NEG | NEG | NEG | NEG | NEG | NEG |
| | NEG | NEG | NEG | POS | NEG | NEG | NEG | NEG | NEG | NEG | NEG | NEG | NEG | NEG | NEG | NEG | NEG | NEG | NEG | NEG |
| **>HP-28** | NEG | NEG | NEG | NEG | NEG | NEG | NEG | NEG | NEG | POS | POS | NEG | NEG | NEG | NEG | NEG | NEG | NEG | NEG | NEG |
| C. difficile 6407 (GenBank ADEH) in silico prediction | NEG | NEG | NEG | NEG | NEG | NEG | NEG | NEG | NEG | POS | POS | NEG | NEG | NEG | NEG | NEG | NEG | NEG | NEG | NEG |
| **>HP-29** | NEG | NEG | NEG | NEG | NEG | NEG | NEG | NEG | NEG | POS | POS | NEG | NEG | NEG | NEG | NEG | NEG | NEG | NEG | NEG |
| Freiburg-852-29.852-DBK0000279 | NEG | NEG | NEG | NEG | NEG | NEG | NEG | NEG | NEG | POS | POS | NEG | NEG | NEG | NEG | NEG | NEG | NEG | NEG | NEG |
| | NEG | NEG | NEG | NEG | NEG | NEG | NEG | NEG | NEG | POS | POS | NEG | NEG | NEG | NEG | NEG | NEG | NEG | NEG | NEG |
| Freiburg-858-32.858-DBK0000222 | NEG | NEG | NEG | NEG | NEG | NEG | NEG | NEG | NEG | POS | POS | NEG | NEG | NEG | NEG | NEG | NEG | NEG | NEG | NEG |
| **>HP-30** | NEG | NEG | NEG | NEG | NEG | NEG | NEG | AMB | NEG | NEG | NEG | NEG | NEG | NEG | NEG | NEG | NEG | NEG | NEG | NEG |
| Freiburg-1047-76.1047-DBK0000269 | NEG | NEG | NEG | NEG | NEG | NEG | NEG | AMB | NEG | NEG | NEG | NEG | NEG | NEG | NEG | NEG | NEG | NEG | NEG | NEG |
| | NEG | NEG | NEG | NEG | NEG | NEG | NEG | NEG | NEG | NEG | NEG | NEG | NEG | NEG | NEG | NEG | NEG | NEG | NEG | NEG |
| Freiburg-999-66.999-DBK0000262 | NEG | NEG | NEG | NEG | NEG | NEG | NEG | AMB | NEG | NEG | NEG | NEG | NEG | NEG | NEG | NEG | NEG | NEG | NEG | NEG |
| | NEG | NEG | NEG | NEG | NEG | NEG | NEG | NEG | NEG | NEG | NEG | NEG | NEG | NEG | NEG | NEG | NEG | NEG | NEG | NEG |
| Freiburg-896-39.896-DBK0000291 | NEG | NEG | NEG | NEG | NEG | NEG | NEG | AMB | NEG | NEG | NEG | NEG | NEG | NEG | NEG | NEG | NEG | NEG | NEG | NEG |
| | NEG | NEG | NEG | NEG | NEG | NEG | NEG | NEG | NEG | NEG | NEG | NEG | NEG | NEG | NEG | NEG | NEG | NEG | NEG | NEG |
| Freiburg-871-33.871-DBK0000272 | NEG | NEG | NEG | NEG | NEG | NEG | NEG | POS | NEG | NEG | NEG | NEG | NEG | NEG | NEG | NEG | NEG | NEG | NEG | NEG |
| | NEG | NEG | NEG | NEG | NEG | NEG | NEG | AMB | NEG | NEG | NEG | NEG | NEG | NEG | NEG | NEG | NEG | NEG | NEG | NEG |
| Dresden 08_1596 | NEG | NEG | NEG | NEG | NEG | NEG | NEG | NEG | NEG | NEG | NEG | NEG | NEG | NEG | NEG | NEG | NEG | NEG | NEG | NEG |

| STRAIN / ISOLATE | putative endonuclease relaxase Tn1549-like | | | | | | putative calcium-binding adhesion protein | | ethanolamine iron-dependent alcohol dehydrogenase | | leucine Rich repeat-containing domain protein | splA Surface layer protein A | | | | | | | | | | | | | | | | | | | |
|---|---|---|---|---|---|---|---|---|---|---|---|---|---|---|---|---|---|---|---|---|---|---|---|---|---|---|---|---|---|---|---|
| | hp1214\|CD0425 | hp1220\|CD0425 | hp1228\|CD0425 | hp1229\|CD0425 | hp1230\|CD0425 | hp1231\|CD0425 | hp1149\|CD2797 | hp1212\|CD2797 | hp1061\|eutG | hp1062\|eutG | hp1227\|IPR007253 | hp1150\|slpA | hp1151\|slpA | hp1153\|slpA | hp1154\|slpA | hp1155\|slpA | hp1156\|slpA | hp1158\|slpA | hp1164\|slpA | hp1166\|slpA | hp1167\|slpA | hp1168\|slpA | hp1169\|slpA | hp1170\|slpA | hp1171\|slpA | hp1173\|slpA | hp1174\|slpA | hp1175\|slpA | hp1176\|slpA | hp1177\|slpA | hp1178\|slpA |
| **CLADE 2 (CC1 /Ribo 27 Complex)** | | | | | | | | | | | | | | | | | | | | | | | | | | | | | | | |
| **>HP-31** | NEG | NEG | NEG | NEG | NEG | NEG | POS | POS | POS | NEG | POS | POS | NEG | POS | NEG | NEG | NEG | NEG | NEG | NEG | NEG | NEG | NEG | NEG | NEG | NEG | NEG | NEG | NEG | NEG | NEG |
| C. difficile BI1 (GenBank FN668941) in silico prediction | NEG | NEG | NEG | NEG | NEG | NEG | POS | POS | POS | NEG | POS | POS | NEG | POS | NEG | NEG | NEG | NEG | NEG | NEG | NEG | NEG | NEG | NEG | NEG | NEG | NEG | NEG | NEG | NEG | NEG |
| C. difficile QCD-97b34 (GenBank ABHF) in silico prediction | NEG | NEG | NEG | NEG | NEG | NEG | POS | POS | POS | NEG | POS | POS | NEG | POS | NEG | NEG | NEG | NEG | NEG | NEG | NEG | NEG | NEG | NEG | NEG | NEG | NEG | NEG | NEG | NEG | NEG |
| C. difficile QCD-76w55 (GenBank ABHE) in silico prediction | NEG | NEG | NEG | NEG | NEG | NEG | POS | POS | POS | NEG | POS | POS | NEG | POS | NEG | NEG | NEG | NEG | NEG | NEG | NEG | NEG | NEG | NEG | NEG | NEG | NEG | NEG | NEG | NEG | NEG |
| C. difficile CIP107932 (GenBank ABKK) in silico prediction | NEG | NEG | NEG | NEG | NEG | NEG | POS | POS | POS | NEG | POS | POS | NEG | POS | NEG | NEG | NEG | NEG | NEG | NEG | NEG | NEG | NEG | NEG | NEG | NEG | NEG | NEG | NEG | NEG | NEG |
| C. difficile CD196 (GenBank FN538970) in silico prediction | NEG | NEG | NEG | NEG | NEG | NEG | POS | POS | POS | NEG | POS | POS | NEG | POS | NEG | NEG | NEG | NEG | NEG | NEG | NEG | NEG | NEG | NEG | NEG | NEG | NEG | NEG | NEG | NEG | NEG |
| C. difficile Strain CD196 | NEG | NEG | NEG | NEG | NEG | NEG | POS | NEG | POS | NEG | NEG | POS | NEG | POS | NEG | NEG | NEG | NEG | NEG | NEG | NEG | NEG | NEG | NEG | NEG | NEG | NEG | NEG | NEG | NEG | NEG |
| | NEG | NEG | NEG | NEG | NEG | NEG | POS | AMB | POS | NEG | AMB | POS | NEG | POS | NEG | NEG | NEG | NEG | NEG | NEG | NEG | NEG | NEG | NEG | NEG | NEG | NEG | NEG | NEG | NEG | NEG |
| | NEG | NEG | NEG | AMB | AMB | NEG | POS | NEG | POS | NEG | AMB | POS | NEG | POS | NEG | NEG | NEG | NEG | NEG | NEG | NEG | NEG | NEG | NEG | NEG | NEG | NEG | NEG | NEG | NEG | NEG |
| | NEG | NEG | NEG | NEG | NEG | NEG | POS | NEG | POS | NEG | AMB | POS | NEG | POS | NEG | NEG | NEG | NEG | NEG | NEG | NEG | NEG | NEG | NEG | NEG | NEG | NEG | NEG | NEG | NEG | NEG |
| | NEG | NEG | NEG | NEG | NEG | NEG | POS | POS | POS | NEG | AMB | POS | NEG | POS | NEG | NEG | NEG | NEG | NEG | NEG | NEG | NEG | NEG | NEG | NEG | NEG | NEG | NEG | NEG | NEG | NEG |
| Dresden 07-7829 | NEG | NEG | NEG | NEG | NEG | NEG | POS | POS | POS | NEG | POS | POS | NEG | POS | NEG | NEG | NEG | NEG | NEG | NEG | NEG | NEG | NEG | NEG | NEG | NEG | NEG | NEG | NEG | NEG | NEG |
| Dresden 07-8176 | NEG | NEG | NEG | NEG | NEG | NEG | POS | POS | POS | NEG | POS | POS | NEG | POS | NEG | NEG | NEG | NEG | NEG | NEG | NEG | NEG | NEG | NEG | NEG | NEG | NEG | NEG | NEG | NEG | NEG |
| **>HP-32** | POS | NEG | NEG | NEG | NEG | NEG | POS | POS | POS | NEG | POS | POS | NEG | POS | NEG | NEG | NEG | NEG | NEG | NEG | NEG | NEG | NEG | NEG | NEG | NEG | NEG | NEG | NEG | NEG | NEG |
| C. difficile 2007855 (GenBank FN665654) in silico prediction | POS | NEG | NEG | NEG | NEG | NEG | POS | POS | POS | NEG | POS | POS | NEG | POS | NEG | NEG | NEG | NEG | NEG | NEG | NEG | NEG | NEG | NEG | NEG | NEG | NEG | NEG | NEG | NEG | NEG |
| C. difficile QCD-32g58 (GenBank AAML) in silico prediction | POS | NEG | NEG | NEG | NEG | NEG | POS | POS | POS | NEG | POS | POS | NEG | POS | NEG | NEG | NEG | NEG | NEG | NEG | NEG | NEG | NEG | NEG | NEG | NEG | NEG | NEG | NEG | NEG | NEG |
| C. difficile QCD-37x79 (GenBank ABHG) in silico prediction | POS | NEG | NEG | NEG | NEG | NEG | POS | POS | POS | NEG | POS | POS | NEG | POS | NEG | NEG | NEG | NEG | NEG | NEG | NEG | NEG | NEG | NEG | NEG | NEG | NEG | NEG | NEG | NEG | NEG |
| C. difficile QCD-66c26 (GenBank ABFD) in silico prediction | POS | NEG | NEG | NEG | NEG | NEG | POS | POS | POS | NEG | POS | POS | NEG | POS | NEG | NEG | NEG | NEG | NEG | NEG | NEG | NEG | NEG | NEG | NEG | NEG | NEG | NEG | NEG | NEG | NEG |
| C. difficile R20291 (GenBank FN545816) in silico prediction | POS | NEG | NEG | NEG | NEG | NEG | POS | POS | POS | NEG | POS | POS | NEG | POS | NEG | NEG | NEG | NEG | NEG | NEG | NEG | NEG | NEG | NEG | NEG | NEG | NEG | NEG | NEG | NEG | NEG |
| C. difficile Strain R20291 | POS | NEG | NEG | NEG | NEG | NEG | POS | NEG | POS | NEG | NEG | POS | NEG | POS | NEG | NEG | NEG | NEG | NEG | NEG | NEG | NEG | NEG | NEG | NEG | NEG | NEG | NEG | NEG | NEG | NEG |
| | POS | NEG | NEG | AMB | AMB | NEG | POS | NEG | POS | NEG | AMB | POS | NEG | POS | NEG | NEG | NEG | NEG | NEG | NEG | NEG | NEG | NEG | NEG | NEG | NEG | NEG | NEG | NEG | NEG | NEG |
| | POS | NEG | NEG | NEG | NEG | NEG | POS | NEG | POS | NEG | AMB | POS | NEG | POS | NEG | NEG | NEG | NEG | NEG | NEG | NEG | NEG | NEG | NEG | NEG | NEG | NEG | NEG | NEG | NEG | NEG |
| | POS | NEG | NEG | NEG | NEG | NEG | POS | POS | POS | NEG | AMB | POS | NEG | POS | NEG | NEG | NEG | NEG | NEG | NEG | NEG | NEG | NEG | NEG | NEG | NEG | NEG | NEG | NEG | NEG | NEG |
| **CLADE 3 (CC5 /Ribo 23 Complex)** | | | | | | | | | | | | | | | | | | | | | | | | | | | | | | | |
| **>HP-33** | NEG | NEG | NEG | NEG | NEG | NEG | POS | NEG | POS | NEG | NEG | NEG | NEG | NEG | NEG | NEG | POS | NEG | NEG | NEG | NEG | NEG | NEG | NEG | NEG | NEG | NEG | NEG | NEG | NEG | NEG |
| Freiburg872-34.872-DBK0000295 | NEG | NEG | NEG | NEG | NEG | NEG | POS | AMB | POS | NEG | NEG | NEG | NEG | NEG | NEG | NEG | POS | NEG | NEG | NEG | NEG | NEG | NEG | NEG | NEG | NEG | NEG | NEG | NEG | NEG | NEG |
| | NEG | NEG | NEG | NEG | NEG | NEG | POS | NEG | POS | NEG | NEG | NEG | NEG | NEG | NEG | NEG | POS | NEG | NEG | NEG | NEG | NEG | NEG | NEG | NEG | NEG | NEG | NEG | NEG | NEG | NEG |
| Freiburg-773-19.773-DBK0000274 | NEG | NEG | NEG | NEG | NEG | NEG | POS | NEG | POS | NEG | NEG | NEG | NEG | NEG | NEG | NEG | POS | NEG | NEG | NEG | NEG | NEG | NEG | NEG | NEG | NEG | NEG | NEG | NEG | NEG | NEG |
| | NEG | NEG | NEG | NEG | NEG | NEG | POS | NEG | POS | NEG | NEG | NEG | NEG | NEG | NEG | NEG | POS | NEG | NEG | NEG | NEG | NEG | NEG | NEG | NEG | NEG | NEG | NEG | NEG | NEG | NEG |
| **>HP-34** | NEG | NEG | NEG | NEG | NEG | NEG | AMB | NEG | POS | NEG | NEG | NEG | NEG | NEG | NEG | NEG | POS | NEG | NEG | NEG | NEG | NEG | NEG | NEG | NEG | NEG | NEG | NEG | NEG | NEG | NEG |
| Jena_0018/11 | NEG | NEG | NEG | NEG | NEG | NEG | AMB | NEG | POS | NEG | NEG | NEG | NEG | NEG | NEG | NEG | POS | NEG | NEG | NEG | NEG | NEG | NEG | NEG | NEG | NEG | NEG | NEG | NEG | NEG | NEG |
| **CLADE 4 (CC37/Ribo 17 Complex)** | | | | | | | | | | | | | | | | | | | | | | | | | | | | | | | |
| **>HP-35** | NEG | NEG | NEG | POS | AMB | NEG | POS | POS | POS | NEG | NEG | NEG | NEG | NEG | NEG | NEG | NEG | NEG | NEG | NEG | NEG | NEG | NEG | NEG | NEG | NEG | NEG | NEG | NEG | NEG | NEG |
| C. difficile 002-P50-2011 (GenBank AGAA) in silico prediction | NEG | NEG | NEG | POS | NEG | NEG | POS | POS | POS | NEG | NEG | NEG | NEG | NEG | NEG | NEG | NEG | NEG | NEG | NEG | NEG | NEG | NEG | NEG | NEG | NEG | NEG | NEG | NEG | NEG | NEG |
| C. difficile 050-P50-2011 (GenBank AGAB) in silico prediction | NEG | NEG | NEG | POS | NEG | NEG | POS | POS | POS | NEG | POS | NEG | NEG | NEG | NEG | NEG | NEG | NEG | NEG | NEG | NEG | NEG | NEG | NEG | NEG | NEG | NEG | NEG | NEG | NEG | NEG |
| C. difficile M68 (GenBank FN668375) in silico prediction | NEG | NEG | NEG | POS | NEG | NEG | POS | POS | POS | NEG | POS | NEG | NEG | NEG | NEG | NEG | NEG | NEG | NEG | NEG | NEG | NEG | NEG | NEG | NEG | NEG | NEG | NEG | NEG | NEG | NEG |
| C. difficile CF5 (GenBank FN665652) in silico prediction | NEG | NEG | NEG | POS | NEG | NEG | POS | POS | POS | NEG | NEG | NEG | NEG | NEG | NEG | NEG | NEG | NEG | NEG | NEG | NEG | NEG | NEG | NEG | NEG | NEG | NEG | NEG | NEG | NEG | NEG |
| C. difficile Strain CF5 | NEG | NEG | NEG | POS | AMB | NEG | POS | NEG | POS | NEG | NEG | NEG | NEG | NEG | NEG | NEG | NEG | NEG | NEG | NEG | NEG | NEG | NEG | NEG | NEG | NEG | NEG | NEG | NEG | NEG | NEG |
| | NEG | NEG | NEG | POS | POS | NEG | POS | NEG | POS | NEG | NEG | NEG | NEG | NEG | NEG | NEG | NEG | NEG | NEG | NEG | NEG | NEG | NEG | NEG | NEG | NEG | NEG | NEG | NEG | NEG | NEG |
| | NEG | NEG | NEG | POS | NEG | NEG | POS | NEG | POS | NEG | NEG | NEG | NEG | NEG | NEG | NEG | NEG | NEG | NEG | NEG | NEG | NEG | NEG | NEG | NEG | NEG | NEG | NEG | NEG | NEG | NEG |
| | NEG | NEG | NEG | POS | NEG | NEG | POS | AMB | POS | NEG | NEG | NEG | NEG | NEG | NEG | NEG | NEG | NEG | NEG | NEG | NEG | NEG | NEG | NEG | NEG | NEG | NEG | NEG | NEG | NEG | NEG |
| | NEG | NEG | NEG | POS | AMB | NEG | POS | POS | POS | NEG | NEG | NEG | NEG | NEG | NEG | NEG | NEG | NEG | NEG | NEG | NEG | NEG | NEG | NEG | NEG | NEG | NEG | NEG | NEG | NEG | NEG |
| | NEG | NEG | NEG | POS | NEG | NEG | POS | POS | POS | NEG | NEG | NEG | NEG | NEG | NEG | NEG | NEG | NEG | NEG | NEG | NEG | NEG | NEG | NEG | NEG | NEG | NEG | NEG | NEG | NEG | NEG |
| Freiburg-736-16.736-DBK0000233 | NEG | NEG | NEG | POS | POS | AMB | POS | POS | POS | NEG | NEG | NEG | NEG | NEG | NEG | NEG | NEG | NEG | NEG | NEG | NEG | NEG | NEG | NEG | NEG | NEG | NEG | NEG | NEG | NEG | NEG |
| | NEG | NEG | NEG | POS | NEG | NEG | POS | POS | POS | NEG | NEG | NEG | NEG | NEG | NEG | NEG | NEG | NEG | NEG | NEG | NEG | NEG | NEG | NEG | NEG | NEG | NEG | NEG | NEG | NEG | NEG |
| **>HP-36** | NEG | NEG | NEG | NEG | AMB | NEG | POS | POS | POS | NEG | NEG | NEG | NEG | NEG | NEG | NEG | NEG | NEG | NEG | NEG | NEG | NEG | NEG | NEG | NEG | NEG | NEG | NEG | NEG | NEG | NEG |
| Freiburg-642-5.642-DBK0000281 | NEG | NEG | NEG | AMB | POS | AMB | POS | POS | POS | NEG | NEG | NEG | NEG | NEG | NEG | NEG | NEG | NEG | NEG | NEG | NEG | NEG | NEG | NEG | NEG | NEG | NEG | NEG | NEG | NEG | NEG |
| | NEG | NEG | NEG | NEG | NEG | NEG | POS | POS | POS | NEG | NEG | NEG | NEG | NEG | NEG | NEG | NEG | NEG | NEG | NEG | NEG | NEG | NEG | NEG | NEG | NEG | NEG | NEG | NEG | NEG | NEG |
| **CLADE 5 (CC11 /Ribo 78 Complex)** | | | | | | | | | | | | | | | | | | | | | | | | | | | | | | | |
| **>HP-37** | NEG | AMB | NEG | NEG | NEG | NEG | NEG | NEG | NEG | POS | NEG | NEG | NEG | NEG | NEG | NEG | NEG | NEG | NEG | NEG | NEG | NEG | NEG | NEG | NEG | NEG | NEG | NEG | NEG | POS | NEG |
| C. difficile NAP08 (GenBank ADNX) in silico prediction | NEG | NEG | NEG | NEG | NEG | NEG | NEG | NEG | NEG | POS | NEG | NEG | NEG | NEG | NEG | NEG | NEG | NEG | NEG | NEG | NEG | NEG | NEG | NEG | NEG | NEG | NEG | NEG | NEG | POS | NEG |
| C. difficile NAP07 (GenBank ADVM) in silico prediction | NEG | NEG | NEG | NEG | NEG | NEG | NEG | NEG | NEG | POS | NEG | NEG | NEG | NEG | NEG | NEG | NEG | NEG | NEG | NEG | NEG | NEG | NEG | NEG | NEG | NEG | NEG | NEG | NEG | POS | NEG |
| C. difficile M120 (GenBank FN665653) in silico prediction | NEG | POS | NEG | NEG | NEG | NEG | NEG | NEG | NEG | POS | NEG | NEG | NEG | NEG | NEG | NEG | NEG | NEG | NEG | NEG | NEG | NEG | NEG | NEG | NEG | NEG | NEG | NEG | NEG | POS | NEG |
| C. difficile Strain M120 | NEG | POS | NEG | NEG | NEG | NEG | NEG | NEG | NEG | POS | NEG | NEG | NEG | NEG | NEG | NEG | NEG | NEG | NEG | NEG | NEG | NEG | NEG | NEG | NEG | NEG | NEG | NEG | NEG | POS | NEG |
| | NEG | POS | NEG | AMB | AMB | NEG | NEG | NEG | NEG | POS | NEG | NEG | NEG | NEG | NEG | NEG | NEG | NEG | NEG | NEG | NEG | NEG | NEG | NEG | NEG | NEG | NEG | NEG | NEG | POS | NEG |
| | NEG | POS | NEG | NEG | NEG | NEG | NEG | NEG | NEG | POS | NEG | NEG | NEG | NEG | NEG | NEG | NEG | NEG | NEG | NEG | NEG | NEG | NEG | NEG | NEG | NEG | NEG | NEG | NEG | POS | NEG |
| | NEG | POS | NEG | NEG | NEG | NEG | NEG | NEG | NEG | POS | NEG | NEG | NEG | NEG | NEG | NEG | NEG | NEG | NEG | NEG | NEG | NEG | NEG | NEG | NEG | NEG | NEG | NEG | NEG | POS | NEG |
| | NEG | POS | NEG | NEG | NEG | NEG | NEG | NEG | NEG | POS | NEG | NEG | NEG | NEG | NEG | NEG | NEG | NEG | NEG | NEG | NEG | NEG | NEG | NEG | NEG | NEG | NEG | NEG | NEG | POS | NEG |
| Freiburg_69.1017 | NEG | NEG | NEG | NEG | NEG | NEG | NEG | NEG | NEG | AMB | NEG | NEG | NEG | NEG | NEG | NEG | NEG | NEG | NEG | NEG | NEG | NEG | POS | NEG | NEG | NEG | NEG | NEG | NEG | POS | NEG |
| Freiburg-1051-77.1051-DBK0000293 | NEG | NEG | NEG | NEG | AMB | AMB | NEG | NEG | NEG | POS | NEG | NEG | NEG | NEG | NEG | NEG | NEG | NEG | NEG | NEG | NEG | NEG | NEG | NEG | NEG | NEG | NEG | NEG | NEG | POS | NEG |
| | NEG | NEG | NEG | NEG | NEG | NEG | NEG | NEG | NEG | POS | NEG | NEG | NEG | NEG | NEG | NEG | NEG | NEG | NEG | NEG | NEG | NEG | NEG | NEG | NEG | NEG | NEG | NEG | NEG | POS | NEG |
| Freiburg-649-6.649-DBK0000235 | NEG | NEG | NEG | AMB | POS | AMB | NEG | NEG | NEG | POS | NEG | NEG | NEG | NEG | NEG | NEG | NEG | NEG | NEG | NEG | NEG | NEG | NEG | NEG | NEG | NEG | NEG | NEG | NEG | POS | NEG |
| | NEG | NEG | NEG | NEG | NEG | NEG | NEG | NEG | NEG | POS | NEG | NEG | NEG | NEG | NEG | NEG | NEG | NEG | NEG | NEG | NEG | NEG | NEG | NEG | NEG | NEG | NEG | NEG | NEG | POS | NEG |
| Freiburg-700-11.7-DBK0000242 | NEG | NEG | NEG | NEG | NEG | NEG | NEG | NEG | NEG | POS | NEG | NEG | NEG | NEG | NEG | NEG | NEG | NEG | NEG | NEG | NEG | NEG | NEG | NEG | NEG | NEG | NEG | NEG | NEG | POS | NEG |
| | NEG | NEG | NEG | NEG | NEG | NEG | NEG | NEG | NEG | POS | NEG | NEG | NEG | NEG | NEG | NEG | NEG | NEG | NEG | NEG | NEG | NEG | NEG | NEG | NEG | NEG | NEG | NEG | NEG | POS | NEG |
| Freiburg-855-30.855-DBK0000277 | NEG | NEG | NEG | NEG | NEG | NEG | NEG | NEG | NEG | POS | NEG | NEG | NEG | NEG | NEG | NEG | NEG | NEG | NEG | NEG | NEG | NEG | NEG | NEG | NEG | NEG | NEG | NEG | NEG | POS | NEG |
| | NEG | NEG | NEG | NEG | NEG | NEG | NEG | NEG | NEG | POS | NEG | NEG | NEG | NEG | NEG | NEG | NEG | NEG | NEG | NEG | NEG | NEG | NEG | NEG | NEG | NEG | NEG | NEG | NEG | POS | NEG |
| Dresden 08_1353 | NEG | NEG | NEG | NEG | NEG | NEG | NEG | NEG | NEG | POS | NEG | NEG | NEG | NEG | NEG | NEG | NEG | NEG | NEG | NEG | NEG | NEG | NEG | NEG | NEG | NEG | NEG | NEG | NEG | POS | NEG |
| Dresden 08_574 | NEG | NEG | NEG | AMB | AMB | AMB | NEG | NEG | NEG | POS | NEG | NEG | NEG | NEG | NEG | NEG | NEG | NEG | NEG | NEG | NEG | NEG | NEG | NEG | NEG | NEG | NEG | NEG | NEG | POS | NEG |
| Dresden 09_2650 | NEG | NEG | NEG | NEG | NEG | NEG | NEG | NEG | NEG | POS | NEG | NEG | NEG | NEG | NEG | NEG | NEG | NEG | NEG | NEG | NEG | NEG | NEG | NEG | NEG | NEG | NEG | NEG | NEG | POS | NEG |
| **>HP-38** | NEG | NEG | NEG | NEG | POS | NEG | NEG | NEG | NEG | POS | NEG | NEG | NEG | NEG | NEG | NEG | NEG | NEG | NEG | NEG | NEG | NEG | NEG | NEG | NEG | NEG | NEG | NEG | NEG | POS | NEG |
| C. difficile 6466 (GenBank ADDE) in silico prediction | NEG | NEG | NEG | NEG | POS | NEG | NEG | NEG | NEG | POS | NEG | NEG | NEG | NEG | NEG | NEG | NEG | NEG | NEG | NEG | NEG | NEG | NEG | NEG | NEG | NEG | NEG | NEG | NEG | POS | NEG |
| **>HP-39** | NEG | NEG | AMB | NEG | NEG | NEG | NEG | NEG | NEG | NEG | NEG | NEG | NEG | NEG | NEG | NEG | NEG | NEG | NEG | NEG | NEG | NEG | POS | POS | NEG | NEG | NEG | NEG | NEG | NEG | NEG |
| C. difficile QCD-23m63 (GenBank ABKL) in silico prediction | NEG | NEG | POS | NEG | NEG | NEG | NEG | NEG | NEG | NEG | NEG | NEG | NEG | NEG | NEG | NEG | NEG | NEG | NEG | NEG | NEG | NEG | POS | POS | NEG | NEG | NEG | NEG | NEG | NEG | NEG |
| Freiburg-956-51.956-DBK0000266 | NEG | NEG | NEG | NEG | NEG | NEG | NEG | NEG | NEG | NEG | NEG | NEG | NEG | NEG | NEG | NEG | NEG | NEG | NEG | NEG | NEG | NEG | POS | POS | NEG | NEG | NEG | NEG | NEG | NEG | NEG |
| Freiburg_50.963 | NEG | NEG | NEG | NEG | NEG | NEG | NEG | NEG | NEG | NEG | NEG | NEG | NEG | NEG | NEG | NEG | NEG | NEG | NEG | NEG | NEG | NEG | POS | POS | NEG | NEG | NEG | NEG | NEG | NEG | NEG |
| **"CLADE 6"** | | | | | | | | | | | | | | | | | | | | | | | | | | | | | | | |
| **>HP-40** | NEG | NEG | NEG | NEG | NEG | NEG | POS | POS | POS | NEG | NEG | NEG | NEG | NEG | NEG | NEG | NEG | NEG | POS | NEG | NEG | NEG | NEG | NEG | NEG | NEG | NEG | NEG | NEG | NEG | NEG |
| C. difficile 6503 (GenBank ADEI) in silico prediction | NEG | NEG | NEG | NEG | NEG | NEG | POS | POS | POS | NEG | NEG | NEG | NEG | NEG | NEG | NEG | NEG | NEG | POS | NEG | NEG | NEG | NEG | NEG | NEG | NEG | NEG | NEG | NEG | NEG | NEG |

| STRAIN / ISOLATE | splA Surface layer protein A, cont. | | | | | | | | | | | | | | | | | | | | | | | | | | | | | | | | |
|---|---|---|---|---|---|---|---|---|---|---|---|---|---|---|---|---|---|---|---|---|---|---|---|---|---|---|---|---|---|---|---|---|---|
| | hp1180\|slpA | hp1182\|slpA | hp1183\|slpA | hp1184\|slpA | hp1186\|slpA | hp1188\|slpA | hp1190\|slpA | hp1191\|slpA | hp1192\|slpA | hp1193\|slpA | hp1195\|slpA | hp1197\|slpA | hp1198\|slpA | hp1199\|slpA | hp1200\|slpA | hp1201\|slpA | hp1202\|slpA | hp1203\|slpA | hp1204\|slpA | hp1205\|slpA | hp1206\|slpA | hp1208\|slpA | hp1209\|slpA | hp1210\|slpA | hp1211\|slpA | hp1232\|slpA | hp1233\|slpA | hp1234\|slpA | hp1236\|slpA | hp1237\|slpA | hp1239\|slpA | hp1242\|slpA | hp1243\|slpA | hp1249\|slpA |
| **CLADE 1** | | | | | | | | | | | | | | | | | | | | | | | | | | | | | | | | | | |
| **>HP-01** | NEG | NEG | NEG | NEG | NEG | NEG | POS | NEG | NEG | NEG | NEG | NEG | NEG | NEG | NEG | NEG | NEG | NEG | NEG | NEG | NEG | NEG | NEG | NEG | NEG | NEG | NEG | NEG | NEG | NEG | NEG | NEG | NEG | NEG |
| C. difficile QCD-63q42 (GenBank ABHD) in silico prediction | NEG | NEG | NEG | NEG | NEG | NEG | POS | NEG | NEG | NEG | NEG | NEG | NEG | NEG | NEG | NEG | NEG | NEG | NEG | NEG | NEG | NEG | NEG | NEG | NEG | NEG | NEG | NEG | NEG | NEG | NEG | NEG | NEG | NEG |
| C. difficile BI9 (GenBank FN668944) in silico prediction | NEG | NEG | NEG | NEG | NEG | NEG | POS | NEG | NEG | NEG | NEG | NEG | NEG | NEG | NEG | NEG | NEG | NEG | NEG | NEG | NEG | NEG | NEG | NEG | NEG | NEG | NEG | NEG | NEG | NEG | NEG | NEG | NEG | NEG |
| C. difficile BI-9 | NEG | NEG | NEG | NEG | NEG | NEG | POS | NEG | NEG | NEG | NEG | NEG | NEG | NEG | NEG | NEG | NEG | NEG | NEG | NEG | NEG | NEG | NEG | NEG | NEG | NEG | NEG | NEG | NEG | NEG | NEG | NEG | NEG | NEG |
| | NEG | NEG | NEG | NEG | NEG | NEG | POS | NEG | NEG | NEG | NEG | NEG | NEG | NEG | NEG | NEG | NEG | NEG | NEG | NEG | NEG | NEG | NEG | NEG | NEG | NEG | NEG | NEG | NEG | NEG | NEG | NEG | NEG | NEG |
| | NEG | NEG | NEG | NEG | NEG | NEG | POS | NEG | NEG | NEG | NEG | AMB | NEG | NEG | NEG | NEG | NEG | NEG | NEG | NEG | NEG | NEG | NEG | NEG | NEG | NEG | NEG | NEG | NEG | NEG | NEG | NEG | NEG | NEG |
| | NEG | NEG | NEG | NEG | NEG | NEG | POS | NEG | NEG | NEG | NEG | NEG | NEG | NEG | NEG | NEG | NEG | NEG | NEG | NEG | NEG | NEG | NEG | NEG | NEG | NEG | NEG | NEG | NEG | NEG | NEG | NEG | NEG | NEG |
| | NEG | NEG | NEG | NEG | NEG | NEG | POS | NEG | NEG | NEG | NEG | NEG | NEG | NEG | NEG | NEG | NEG | NEG | NEG | NEG | NEG | NEG | NEG | NEG | NEG | NEG | NEG | NEG | NEG | NEG | NEG | NEG | NEG | NEG |
| Freiburg-1040-73.104-DBK0000249 | NEG | NEG | NEG | NEG | NEG | NEG | POS | NEG | NEG | NEG | NEG | NEG | NEG | NEG | NEG | NEG | NEG | NEG | NEG | NEG | NEG | NEG | NEG | NEG | NEG | NEG | NEG | NEG | NEG | NEG | NEG | NEG | NEG | NEG |
| | NEG | NEG | NEG | NEG | NEG | NEG | POS | NEG | NEG | NEG | NEG | NEG | NEG | NEG | NEG | NEG | NEG | NEG | NEG | NEG | NEG | NEG | NEG | NEG | NEG | NEG | NEG | NEG | NEG | NEG | NEG | NEG | NEG | NEG |
| Freiburg-1052-78.1052-DBK0000294 | NEG | NEG | NEG | NEG | NEG | NEG | POS | NEG | NEG | NEG | NEG | NEG | NEG | NEG | NEG | NEG | NEG | NEG | NEG | NEG | NEG | NEG | NEG | NEG | NEG | NEG | NEG | NEG | NEG | NEG | NEG | NEG | NEG | NEG |
| | NEG | NEG | NEG | NEG | NEG | NEG | POS | NEG | NEG | NEG | NEG | NEG | NEG | NEG | NEG | NEG | NEG | NEG | NEG | NEG | NEG | NEG | NEG | NEG | NEG | NEG | NEG | NEG | NEG | NEG | NEG | NEG | NEG | NEG |
| Freiburg-605-1.605-DBK0000223 | NEG | NEG | NEG | NEG | NEG | NEG | POS | NEG | NEG | NEG | NEG | NEG | NEG | NEG | NEG | NEG | NEG | NEG | NEG | NEG | NEG | NEG | NEG | NEG | NEG | NEG | NEG | NEG | NEG | NEG | NEG | NEG | NEG | NEG |
| Freiburg-787-22.787-DBK0000288 | NEG | NEG | NEG | NEG | NEG | NEG | POS | NEG | NEG | NEG | NEG | NEG | NEG | NEG | NEG | NEG | NEG | NEG | NEG | NEG | NEG | NEG | NEG | NEG | NEG | NEG | NEG | NEG | NEG | NEG | NEG | NEG | NEG | NEG |
| | NEG | NEG | NEG | NEG | NEG | NEG | POS | NEG | NEG | NEG | NEG | NEG | NEG | NEG | NEG | NEG | NEG | NEG | NEG | NEG | NEG | NEG | NEG | NEG | NEG | NEG | NEG | NEG | NEG | NEG | NEG | NEG | NEG | NEG |
| Freiburg-897-40.897-DBK0000225 | NEG | NEG | NEG | NEG | NEG | NEG | POS | NEG | NEG | NEG | NEG | NEG | NEG | NEG | NEG | NEG | NEG | NEG | NEG | NEG | NEG | NEG | NEG | NEG | NEG | NEG | NEG | NEG | NEG | NEG | NEG | NEG | NEG | NEG |
| | NEG | NEG | NEG | NEG | NEG | NEG | POS | NEG | NEG | NEG | NEG | NEG | NEG | NEG | NEG | NEG | NEG | NEG | NEG | NEG | NEG | NEG | NEG | NEG | NEG | NEG | NEG | NEG | NEG | NEG | NEG | NEG | NEG | NEG |
| Dresden 06_7735 | NEG | NEG | NEG | NEG | NEG | NEG | POS | NEG | NEG | NEG | NEG | NEG | NEG | NEG | NEG | NEG | NEG | NEG | NEG | NEG | NEG | NEG | NEG | NEG | NEG | NEG | NEG | NEG | NEG | NEG | NEG | NEG | NEG | NEG |
| Dresden 06_7736 | NEG | NEG | NEG | NEG | NEG | NEG | POS | NEG | NEG | NEG | NEG | NEG | NEG | NEG | NEG | NEG | NEG | NEG | NEG | NEG | NEG | NEG | NEG | NEG | NEG | NEG | NEG | NEG | NEG | NEG | NEG | NEG | NEG | NEG |
| Dresden 07_6943 | NEG | NEG | NEG | NEG | NEG | NEG | POS | NEG | NEG | NEG | NEG | NEG | NEG | NEG | NEG | NEG | NEG | NEG | NEG | NEG | NEG | NEG | NEG | NEG | NEG | NEG | NEG | NEG | NEG | NEG | NEG | NEG | NEG | NEG |
| Dresden 07_7200 | NEG | NEG | NEG | NEG | NEG | NEG | POS | NEG | NEG | NEG | NEG | NEG | NEG | NEG | NEG | NEG | NEG | NEG | NEG | NEG | NEG | NEG | NEG | NEG | NEG | NEG | NEG | NEG | NEG | NEG | NEG | NEG | NEG | NEG |
| Dresden 07_7269 | NEG | NEG | NEG | NEG | NEG | NEG | POS | NEG | NEG | NEG | NEG | NEG | NEG | NEG | NEG | NEG | NEG | NEG | NEG | NEG | NEG | NEG | NEG | NEG | NEG | NEG | NEG | NEG | NEG | NEG | NEG | NEG | NEG | NEG |
| Dresden 07_7678 | NEG | NEG | NEG | NEG | NEG | NEG | POS | NEG | NEG | NEG | NEG | NEG | NEG | NEG | NEG | NEG | NEG | NEG | NEG | NEG | NEG | NEG | NEG | NEG | NEG | NEG | NEG | NEG | NEG | NEG | NEG | NEG | NEG | NEG |
| Dresden 07_7773 | NEG | NEG | NEG | NEG | NEG | NEG | POS | NEG | NEG | NEG | NEG | NEG | NEG | NEG | NEG | NEG | NEG | NEG | NEG | NEG | NEG | NEG | NEG | NEG | NEG | NEG | NEG | NEG | NEG | NEG | NEG | NEG | NEG | NEG |
| Dresden 07_7832 | NEG | NEG | NEG | NEG | NEG | NEG | POS | NEG | NEG | NEG | NEG | NEG | NEG | NEG | NEG | NEG | NEG | NEG | NEG | NEG | NEG | NEG | NEG | NEG | NEG | NEG | NEG | NEG | NEG | NEG | NEG | NEG | NEG | NEG |
| Dresden 07_7967 | NEG | NEG | NEG | NEG | NEG | NEG | POS | NEG | NEG | NEG | NEG | NEG | NEG | NEG | NEG | NEG | NEG | NEG | NEG | NEG | NEG | NEG | NEG | NEG | NEG | NEG | NEG | NEG | NEG | NEG | NEG | NEG | NEG | NEG |
| Dresden 07_8099 | NEG | NEG | NEG | NEG | NEG | NEG | POS | NEG | NEG | NEG | NEG | NEG | NEG | NEG | NEG | NEG | NEG | NEG | NEG | NEG | NEG | NEG | NEG | NEG | NEG | NEG | NEG | NEG | NEG | NEG | NEG | NEG | NEG | NEG |
| Dresden 07_8100 | NEG | NEG | NEG | NEG | NEG | NEG | POS | NEG | NEG | NEG | NEG | NEG | NEG | NEG | NEG | NEG | NEG | NEG | NEG | NEG | NEG | NEG | NEG | NEG | NEG | NEG | NEG | NEG | NEG | NEG | NEG | NEG | NEG | NEG |
| Dresden 07_8170 | NEG | NEG | NEG | NEG | NEG | NEG | POS | NEG | NEG | NEG | NEG | NEG | NEG | NEG | NEG | NEG | NEG | NEG | NEG | NEG | NEG | NEG | NEG | NEG | NEG | NEG | NEG | NEG | NEG | NEG | NEG | NEG | NEG | NEG |
| Dresden 08_0544 | NEG | NEG | NEG | NEG | NEG | NEG | POS | NEG | NEG | NEG | NEG | NEG | NEG | NEG | NEG | NEG | NEG | NEG | NEG | NEG | NEG | NEG | NEG | NEG | NEG | NEG | NEG | NEG | NEG | NEG | NEG | NEG | NEG | NEG |
| Dresden 08_0550 | NEG | NEG | NEG | NEG | NEG | NEG | POS | NEG | NEG | NEG | NEG | NEG | NEG | NEG | NEG | NEG | NEG | NEG | NEG | NEG | NEG | NEG | NEG | NEG | NEG | NEG | NEG | NEG | NEG | NEG | NEG | NEG | NEG | NEG |
| Dresden 08_0827 | NEG | AMB | NEG | NEG | NEG | NEG | POS | NEG | NEG | NEG | NEG | NEG | NEG | NEG | NEG | NEG | NEG | NEG | NEG | NEG | NEG | NEG | NEG | NEG | NEG | NEG | NEG | NEG | NEG | NEG | NEG | NEG | NEG | NEG |
| Dresden 08_0902 | NEG | NEG | NEG | NEG | NEG | NEG | POS | NEG | NEG | NEG | NEG | NEG | NEG | NEG | NEG | NEG | NEG | NEG | NEG | NEG | NEG | NEG | NEG | NEG | NEG | NEG | NEG | NEG | NEG | NEG | NEG | NEG | NEG | NEG |
| Dresden 08_0955 | NEG | NEG | NEG | NEG | NEG | NEG | POS | NEG | NEG | NEG | NEG | NEG | NEG | NEG | NEG | NEG | NEG | NEG | NEG | NEG | NEG | NEG | NEG | NEG | NEG | NEG | NEG | NEG | NEG | NEG | NEG | NEG | NEG | NEG |
| Dresden 08_0958 | NEG | NEG | NEG | NEG | NEG | NEG | POS | NEG | NEG | NEG | NEG | NEG | NEG | NEG | NEG | NEG | NEG | NEG | NEG | NEG | NEG | NEG | NEG | NEG | NEG | NEG | NEG | NEG | NEG | NEG | NEG | NEG | NEG | NEG |
| Dresden 08_1073 | NEG | NEG | NEG | NEG | NEG | NEG | POS | NEG | NEG | NEG | NEG | NEG | NEG | NEG | NEG | NEG | NEG | NEG | NEG | NEG | NEG | NEG | NEG | NEG | NEG | NEG | NEG | NEG | NEG | NEG | NEG | NEG | NEG | NEG |
| Dresden 08_1074 | NEG | NEG | NEG | NEG | NEG | NEG | POS | NEG | NEG | NEG | NEG | NEG | NEG | NEG | NEG | NEG | NEG | NEG | NEG | NEG | NEG | NEG | NEG | NEG | NEG | NEG | NEG | NEG | NEG | NEG | NEG | NEG | NEG | NEG |
| Dresden 08_1206 | NEG | NEG | NEG | NEG | NEG | NEG | POS | NEG | NEG | NEG | NEG | NEG | NEG | NEG | NEG | NEG | NEG | NEG | NEG | NEG | NEG | NEG | NEG | NEG | NEG | NEG | NEG | NEG | NEG | NEG | NEG | NEG | NEG | NEG |
| Dresden 08_1216 | NEG | NEG | NEG | NEG | NEG | NEG | AMB | NEG | NEG | NEG | NEG | NEG | NEG | NEG | NEG | NEG | NEG | NEG | NEG | NEG | NEG | NEG | NEG | NEG | NEG | NEG | NEG | NEG | NEG | NEG | NEG | NEG | NEG | NEG |
| Dresden 08_1305 | NEG | AMB | NEG | NEG | NEG | NEG | POS | NEG | NEG | NEG | NEG | NEG | NEG | NEG | NEG | NEG | NEG | NEG | NEG | NEG | NEG | NEG | NEG | NEG | NEG | NEG | NEG | NEG | NEG | NEG | NEG | NEG | NEG | NEG |
| Dresden 07_7679 | NEG | NEG | NEG | NEG | NEG | NEG | POS | NEG | NEG | NEG | NEG | NEG | NEG | NEG | NEG | NEG | NEG | NEG | NEG | NEG | NEG | NEG | NEG | NEG | NEG | NEG | NEG | NEG | NEG | NEG | NEG | NEG | NEG | NEG |
| Dresden 07_7683 | NEG | NEG | NEG | NEG | NEG | NEG | POS | NEG | NEG | NEG | NEG | NEG | NEG | NEG | NEG | NEG | NEG | NEG | NEG | NEG | NEG | NEG | NEG | NEG | NEG | NEG | NEG | NEG | NEG | NEG | NEG | NEG | NEG | NEG |
| Dresden 07_7980 | NEG | NEG | NEG | NEG | NEG | NEG | POS | NEG | NEG | NEG | NEG | NEG | NEG | NEG | NEG | NEG | NEG | NEG | NEG | NEG | NEG | NEG | NEG | NEG | NEG | NEG | NEG | NEG | NEG | NEG | NEG | NEG | NEG | NEG |
| Dresden 07_8022 | NEG | NEG | NEG | NEG | NEG | NEG | POS | NEG | NEG | NEG | NEG | NEG | NEG | NEG | NEG | NEG | NEG | NEG | NEG | NEG | NEG | NEG | NEG | NEG | NEG | NEG | NEG | NEG | NEG | NEG | NEG | NEG | NEG | NEG |
| Dresden 07_8291 | NEG | NEG | NEG | NEG | NEG | NEG | POS | NEG | NEG | NEG | NEG | NEG | NEG | NEG | NEG | NEG | NEG | NEG | NEG | NEG | NEG | NEG | NEG | NEG | NEG | NEG | NEG | NEG | NEG | NEG | NEG | NEG | NEG | NEG |
| Dresden 07_8314 | NEG | NEG | NEG | NEG | NEG | NEG | POS | NEG | NEG | NEG | NEG | NEG | NEG | NEG | NEG | NEG | NEG | NEG | NEG | NEG | NEG | NEG | NEG | NEG | NEG | NEG | NEG | NEG | NEG | NEG | NEG | NEG | NEG | NEG |
| Dresden 07_8315 | NEG | NEG | NEG | NEG | NEG | NEG | POS | NEG | NEG | NEG | NEG | NEG | NEG | NEG | NEG | NEG | NEG | NEG | NEG | NEG | NEG | NEG | NEG | NEG | NEG | NEG | NEG | NEG | NEG | NEG | NEG | NEG | NEG | NEG |
| Dresden 07_8323 | NEG | NEG | NEG | NEG | NEG | NEG | POS | NEG | NEG | NEG | NEG | NEG | NEG | NEG | NEG | NEG | NEG | NEG | NEG | NEG | NEG | NEG | NEG | NEG | NEG | NEG | NEG | NEG | NEG | NEG | NEG | NEG | NEG | NEG |
| Dresden 07_8356 | NEG | NEG | NEG | NEG | NEG | NEG | POS | NEG | NEG | NEG | NEG | NEG | NEG | NEG | NEG | NEG | NEG | NEG | NEG | NEG | NEG | NEG | NEG | NEG | NEG | NEG | NEG | NEG | NEG | NEG | NEG | NEG | NEG | NEG |
| Dresden 07_8557 | NEG | NEG | NEG | NEG | NEG | NEG | POS | NEG | NEG | NEG | NEG | NEG | NEG | NEG | NEG | NEG | NEG | NEG | NEG | NEG | NEG | NEG | NEG | NEG | NEG | NEG | NEG | NEG | NEG | NEG | NEG | NEG | NEG | NEG |
| Dresden 07_8736 | NEG | NEG | NEG | NEG | NEG | NEG | POS | NEG | NEG | NEG | NEG | NEG | NEG | NEG | NEG | NEG | NEG | NEG | NEG | NEG | NEG | NEG | NEG | NEG | NEG | NEG | NEG | NEG | NEG | NEG | NEG | NEG | NEG | NEG |
| Dresden 07_8945 | NEG | NEG | NEG | NEG | NEG | NEG | POS | NEG | NEG | NEG | NEG | NEG | NEG | NEG | NEG | NEG | NEG | NEG | NEG | NEG | NEG | NEG | NEG | NEG | NEG | NEG | NEG | NEG | NEG | NEG | NEG | NEG | NEG | NEG |
| Dresden 07_9138 | NEG | NEG | NEG | NEG | NEG | NEG | POS | NEG | NEG | NEG | NEG | NEG | NEG | NEG | NEG | NEG | NEG | NEG | NEG | NEG | NEG | NEG | NEG | NEG | NEG | NEG | NEG | NEG | NEG | NEG | NEG | NEG | NEG | NEG |
| Dresden 07_9175 | NEG | NEG | NEG | NEG | NEG | NEG | POS | NEG | NEG | NEG | NEG | NEG | NEG | NEG | NEG | NEG | NEG | NEG | NEG | NEG | NEG | NEG | NEG | NEG | NEG | NEG | NEG | NEG | NEG | NEG | NEG | NEG | NEG | NEG |
| Dresden 07_9234 | NEG | NEG | NEG | NEG | NEG | NEG | POS | NEG | NEG | NEG | NEG | NEG | NEG | NEG | NEG | NEG | NEG | NEG | NEG | NEG | NEG | NEG | NEG | NEG | NEG | NEG | NEG | NEG | NEG | NEG | NEG | NEG | NEG | NEG |
| Dresden 07_9255 | NEG | NEG | NEG | NEG | NEG | NEG | POS | NEG | NEG | NEG | NEG | NEG | NEG | NEG | NEG | NEG | NEG | NEG | NEG | NEG | NEG | NEG | NEG | NEG | NEG | NEG | NEG | NEG | NEG | NEG | NEG | NEG | NEG | NEG |
| Dresden 07_9309 | NEG | NEG | NEG | NEG | NEG | NEG | POS | NEG | NEG | NEG | NEG | NEG | NEG | NEG | NEG | NEG | NEG | NEG | NEG | NEG | NEG | NEG | NEG | NEG | NEG | NEG | NEG | NEG | NEG | NEG | NEG | NEG | NEG | NEG |
| Dresden 07_9330 | NEG | NEG | NEG | NEG | NEG | NEG | POS | NEG | NEG | NEG | NEG | NEG | NEG | NEG | NEG | NEG | NEG | NEG | NEG | NEG | NEG | NEG | NEG | NEG | NEG | NEG | NEG | NEG | NEG | NEG | NEG | NEG | NEG | NEG |
| Dresden 07_9399 | NEG | NEG | NEG | NEG | NEG | NEG | POS | NEG | NEG | NEG | NEG | NEG | NEG | NEG | NEG | NEG | NEG | NEG | NEG | NEG | NEG | NEG | NEG | NEG | NEG | NEG | NEG | NEG | NEG | NEG | NEG | NEG | NEG | NEG |
| Dresden 08_0188 | NEG | NEG | NEG | NEG | NEG | NEG | POS | NEG | NEG | NEG | NEG | NEG | NEG | NEG | NEG | NEG | NEG | NEG | NEG | NEG | NEG | NEG | NEG | NEG | NEG | NEG | NEG | NEG | NEG | NEG | NEG | NEG | NEG | NEG |
| Dresden 08_0190 | NEG | NEG | NEG | NEG | NEG | NEG | POS | NEG | NEG | NEG | NEG | NEG | NEG | NEG | NEG | NEG | NEG | NEG | NEG | NEG | NEG | NEG | NEG | NEG | NEG | NEG | NEG | NEG | NEG | NEG | NEG | NEG | NEG | NEG |
| Dresden 08_0220 | NEG | NEG | NEG | NEG | NEG | NEG | POS | NEG | NEG | NEG | NEG | NEG | NEG | NEG | NEG | NEG | NEG | NEG | NEG | NEG | NEG | NEG | NEG | NEG | NEG | NEG | NEG | NEG | NEG | NEG | NEG | NEG | NEG | NEG |
| Dresden 08_0326 | NEG | NEG | NEG | NEG | NEG | NEG | POS | NEG | NEG | NEG | NEG | NEG | NEG | NEG | NEG | NEG | NEG | NEG | NEG | NEG | NEG | NEG | NEG | NEG | NEG | NEG | NEG | NEG | NEG | NEG | NEG | NEG | NEG | NEG |
| Dresden 08_0327 | NEG | NEG | NEG | NEG | NEG | NEG | POS | NEG | NEG | NEG | NEG | NEG | NEG | NEG | NEG | NEG | NEG | NEG | NEG | NEG | NEG | NEG | NEG | NEG | NEG | NEG | NEG | NEG | NEG | NEG | NEG | NEG | NEG | NEG |
| Dresden 08_0648 | NEG | NEG | NEG | NEG | NEG | NEG | POS | NEG | NEG | NEG | NEG | NEG | NEG | NEG | NEG | NEG | NEG | NEG | NEG | NEG | NEG | NEG | NEG | NEG | NEG | NEG | NEG | NEG | NEG | NEG | NEG | NEG | NEG | NEG |
| Dresden 08_0751 | NEG | NEG | NEG | NEG | NEG | NEG | POS | NEG | NEG | NEG | NEG | NEG | NEG | NEG | NEG | NEG | NEG | NEG | NEG | NEG | NEG | NEG | NEG | NEG | NEG | NEG | NEG | NEG | NEG | NEG | NEG | NEG | NEG | NEG |
| Dresden 08_0769 | NEG | NEG | NEG | NEG | NEG | NEG | POS | NEG | NEG | NEG | NEG | NEG | NEG | NEG | NEG | NEG | NEG | NEG | NEG | NEG | NEG | NEG | NEG | NEG | NEG | NEG | NEG | NEG | NEG | NEG | NEG | NEG | NEG | NEG |
| Dresden 08_0770 | NEG | NEG | NEG | NEG | NEG | NEG | POS | NEG | NEG | NEG | NEG | NEG | NEG | NEG | NEG | NEG | NEG | NEG | NEG | NEG | NEG | NEG | NEG | NEG | NEG | NEG | NEG | NEG | NEG | NEG | NEG | NEG | NEG | NEG |
| Dresden 08_0771 | NEG | NEG | NEG | NEG | NEG | NEG | POS | NEG | NEG | NEG | NEG | NEG | NEG | NEG | NEG | NEG | NEG | NEG | NEG | NEG | NEG | NEG | NEG | NEG | NEG | NEG | NEG | NEG | NEG | NEG | NEG | NEG | NEG | NEG |
| Dresden 08_1308 | NEG | NEG | NEG | NEG | NEG | NEG | POS | NEG | NEG | NEG | NEG | NEG | NEG | NEG | NEG | NEG | NEG | NEG | NEG | NEG | NEG | NEG | NEG | NEG | NEG | NEG | NEG | NEG | NEG | NEG | NEG | NEG | NEG | NEG |
| Dresden 08_1311 | NEG | NEG | NEG | NEG | NEG | NEG | POS | NEG | NEG | NEG | NEG | NEG | NEG | NEG | NEG | NEG | NEG | NEG | NEG | NEG | NEG | NEG | NEG | NEG | NEG | NEG | NEG | NEG | NEG | NEG | NEG | NEG | NEG | NEG |
| Dresden 08_1316 | NEG | NEG | NEG | NEG | NEG | NEG | POS | NEG | NEG | NEG | NEG | NEG | NEG | NEG | NEG | NEG | NEG | NEG | NEG | NEG | NEG | NEG | NEG | NEG | NEG | NEG | NEG | NEG | NEG | NEG | NEG | NEG | NEG | NEG |
| Dresden 08_1542 | NEG | AMB | NEG | NEG | NEG | NEG | POS | NEG | NEG | NEG | NEG | NEG | NEG | NEG | NEG | NEG | NEG | NEG | NEG | NEG | NEG | NEG | NEG | NEG | NEG | NEG | NEG | NEG | NEG | NEG | NEG | NEG | NEG | NEG |
| Dresden 08_1544 | NEG | NEG | NEG | NEG | NEG | NEG | POS | NEG | NEG | NEG | NEG | NEG | NEG | NEG | NEG | NEG | NEG | NEG | NEG | NEG | NEG | NEG | NEG | NEG | NEG | NEG | NEG | NEG | NEG | NEG | NEG | NEG | NEG | NEG |
| Dresden 08_1581 | NEG | NEG | NEG | NEG | NEG | NEG | POS | NEG | NEG | NEG | NEG | NEG | NEG | NEG | NEG | NEG | NEG | NEG | NEG | NEG | NEG | NEG | NEG | NEG | NEG | NEG | NEG | NEG | NEG | NEG | NEG | NEG | NEG | NEG |
| Dresden 08_1826 | NEG | AMB | NEG | NEG | NEG | NEG | AMB | NEG | NEG | NEG | NEG | NEG | NEG | NEG | NEG | NEG | NEG | NEG | NEG | NEG | NEG | NEG | NEG | NEG | NEG | NEG | NEG | NEG | NEG | NEG | NEG | NEG | NEG | NEG |
| Dresden 08_1817 | NEG | NEG | NEG | NEG | NEG | NEG | POS | NEG | NEG | NEG | NEG | NEG | NEG | NEG | NEG | NEG | NEG | NEG | NEG | NEG | NEG | NEG | NEG | NEG | NEG | NEG | NEG | NEG | NEG | NEG | NEG | NEG | NEG | NEG |
| Dresden 08_1791 | NEG | NEG | NEG | NEG | NEG | NEG | POS | NEG | NEG | NEG | NEG | NEG | NEG | NEG | NEG | NEG | NEG | NEG | NEG | NEG | NEG | NEG | NEG | NEG | NEG | NEG | NEG | NEG | NEG | NEG | NEG | NEG | NEG | NEG |
| Dresden 08_1643 | NEG | NEG | NEG | NEG | NEG | NEG | POS | NEG | NEG | NEG | NEG | NEG | NEG | NEG | NEG | NEG | NEG | NEG | NEG | NEG | NEG | NEG | NEG | NEG | NEG | NEG | NEG | NEG | NEG | NEG | NEG | NEG | NEG | NEG |
| Dresden 08_1764 | NEG | NEG | NEG | NEG | NEG | NEG | POS | NEG | NEG | NEG | NEG | NEG | NEG | NEG | NEG | NEG | NEG | NEG | NEG | NEG | NEG | NEG | NEG | NEG | NEG | NEG | NEG | NEG | NEG | NEG | NEG | NEG | NEG | NEG |
| Dresden 08_1748 | NEG | NEG | NEG | NEG | NEG | NEG | POS | NEG | NEG | NEG | NEG | NEG | NEG | NEG | NEG | NEG | NEG | NEG | NEG | NEG | NEG | NEG | NEG | NEG | NEG | NEG | NEG | NEG | NEG | NEG | NEG | NEG | NEG | NEG |
| Dresden 08_1736 | NEG | NEG | NEG | NEG | NEG | NEG | POS | NEG | NEG | NEG | NEG | NEG | NEG | NEG | NEG | NEG | NEG | NEG | NEG | NEG | NEG | NEG | NEG | NEG | NEG | NEG | NEG | NEG | NEG | NEG | NEG | NEG | NEG | NEG |
| Dresden 08_1582 | NEG | NEG | NEG | NEG | NEG | NEG | POS | NEG | NEG | NEG | NEG | NEG | NEG | NEG | NEG | NEG | NEG | NEG | NEG | NEG | NEG | NEG | NEG | NEG | NEG | NEG | NEG | NEG | NEG | NEG | NEG | NEG | NEG | NEG |
| **>HP-02** | NEG | NEG | NEG | NEG | NEG | NEG | POS | NEG | NEG | NEG | NEG | NEG | NEG | NEG | NEG | NEG | NEG | NEG | NEG | NEG | NEG | NEG | NEG | NEG | NEG | NEG | NEG | NEG | NEG | NEG | NEG | NEG | NEG | NEG |
| C. difficile ATCC43255 (GenBank ABKJ) in silico prediction | NEG | NEG | NEG | NEG | NEG | NEG | POS | NEG | NEG | NEG | NEG | NEG | NEG | NEG | NEG | NEG | NEG | NEG | NEG | NEG | NEG | NEG | NEG | NEG | NEG | NEG | NEG | NEG | NEG | NEG | NEG | NEG | NEG | NEG |
| Freiburg-885-36.885-DBK0000296 | NEG | NEG | NEG | NEG | NEG | NEG | POS | NEG | NEG | NEG | NEG | NEG | NEG | NEG | NEG | NEG | NEG | NEG | NEG | NEG | NEG | NEG | NEG | NEG | NEG | NEG | NEG | NEG | NEG | NEG | NEG | NEG | NEG | NEG |
| Freiburg_37.894-II | NEG | NEG | NEG | NEG | NEG | NEG | POS | NEG | NEG | NEG | NEG | NEG | NEG | NEG | NEG | NEG | NEG | NEG | NEG | NEG | NEG | NEG | NEG | NEG | NEG | NEG | NEG | NEG | NEG | NEG | NEG | NEG | NEG | NEG |
| Freiburg_981-57.981-DBK0000258 | NEG | NEG | NEG | NEG | NEG | NEG | POS | NEG | NEG | NEG | NEG | NEG | NEG | NEG | NEG | NEG | NEG | NEG | NEG | NEG | NEG | NEG | NEG | NEG | NEG | NEG | NEG | NEG | NEG | NEG | NEG | NEG | NEG | NEG |
| | NEG | NEG | NEG | NEG | NEG | NEG | POS | NEG | NEG | NEG | NEG | NEG | NEG | NEG | NEG | NEG | NEG | NEG | NEG | NEG | NEG | NEG | NEG | NEG | NEG | NEG | NEG | NEG | NEG | NEG | NEG | NEG | NEG | NEG |
| Jena_S0011_11 | NEG | AMB | NEG | NEG | NEG | NEG | POS | NEG | NEG | NEG | NEG | NEG | NEG | NEG | NEG | NEG | NEG | NEG | NEG | NEG | NEG | NEG | NEG | NEG | NEG | AMB | AMB | NEG | NEG | NEG | NEG | NEG | NEG | NEG |
| | NEG | NEG | NEG | NEG | NEG | NEG | POS | NEG | NEG | NEG | NEG | NEG | NEG | NEG | NEG | NEG | NEG | NEG | NEG | NEG | NEG | NEG | NEG | NEG | NEG | NEG | NEG | NEG | NEG | NEG | NEG | NEG | NEG | NEG |
| Dresden 08_1352 | NEG | NEG | NEG | NEG | NEG | NEG | POS | NEG | NEG | NEG | NEG | NEG | NEG | NEG | NEG | NEG | NEG | NEG | NEG | NEG | NEG | NEG | NEG | NEG | NEG | NEG | NEG | NEG | NEG | NEG | NEG | NEG | NEG | NEG |
| **>HP-03** | NEG | NEG | NEG | NEG | NEG | NEG | NEG | NEG | NEG | NEG | NEG | NEG | NEG | NEG | NEG | NEG | NEG | NEG | NEG | NEG | NEG | NEG | NEG | NEG | NEG | NEG | NEG | NEG | NEG | NEG | NEG | NEG | NEG | NEG |
| Freiburg-938-47.938-DBK0000237 | NEG | NEG | NEG | NEG | NEG | NEG | NEG | NEG | NEG | NEG | NEG | NEG | NEG | NEG | NEG | NEG | NEG | NEG | NEG | NEG | NEG | NEG | NEG | NEG | NEG | NEG | NEG | NEG | NEG | NEG | NEG | NEG | NEG | NEG |
| | NEG | NEG | NEG | NEG | NEG | NEG | NEG | NEG | NEG | NEG | NEG | NEG | NEG | NEG | NEG | NEG | NEG | NEG | NEG | NEG | NEG | NEG | NEG | NEG | NEG | NEG | NEG | NEG | NEG | NEG | NEG | NEG | NEG | NEG |
| Freiburg-811-25.811-DBK0000284 | NEG | NEG | POS | NEG | NEG | NEG | NEG | NEG | NEG | NEG | NEG | NEG | NEG | NEG | NEG | NEG | NEG | NEG | NEG | NEG | NEG | NEG | NEG | NEG | NEG | NEG | NEG | NEG | NEG | NEG | NEG | NEG | NEG | NEG |
| | NEG | NEG | NEG | NEG | NEG | NEG | NEG | NEG | NEG | NEG | NEG | NEG | NEG | NEG | NEG | NEG | NEG | NEG | NEG | NEG | NEG | NEG | NEG | NEG | NEG | NEG | NEG | NEG | NEG | NEG | NEG | NEG | NEG | NEG |
| Freiburg-987-62.987-DBK0000251 | NEG | NEG | NEG | NEG | NEG | NEG | NEG | NEG | NEG | NEG | NEG | NEG | NEG | NEG | NEG | NEG | NEG | NEG | NEG | NEG | NEG | NEG | NEG | NEG | NEG | NEG | NEG | NEG | NEG | NEG | NEG | NEG | NEG | NEG |
| | NEG | NEG | NEG | NEG | NEG | NEG | NEG | NEG | NEG | NEG | NEG | NEG | NEG | NEG | NEG | NEG | NEG | NEG | NEG | NEG | NEG | NEG | NEG | NEG | NEG | NEG | NEG | NEG | NEG | NEG | NEG | NEG | NEG | NEG |
| Freiburg-972-54.972-DBK0000247 | NEG | NEG | NEG | NEG | NEG | NEG | NEG | NEG | NEG | NEG | NEG | NEG | NEG | NEG | NEG | NEG | NEG | NEG | NEG | NEG | NEG | NEG | NEG | NEG | NEG | NEG | NEG | NEG | NEG | NEG | NEG | NEG | NEG | NEG |
| | NEG | NEG | NEG | NEG | NEG | NEG | NEG | NEG | NEG | NEG | NEG | NEG | NEG | NEG | NEG | NEG | NEG | NEG | NEG | NEG | NEG | NEG | NEG | NEG | NEG | NEG | NEG | NEG | NEG | NEG | NEG | NEG | NEG | NEG |
| **>HP-04** | NEG | NEG | NEG | NEG | NEG | NEG | NEG | NEG | NEG | NEG | NEG | NEG | POS | NEG | NEG | NEG | NEG | NEG | NEG | NEG | NEG | NEG | NEG | NEG | NEG | NEG | NEG | NEG | NEG | NEG | NEG | NEG | NEG | NEG |
| Freiburg-984-60.984-DBK0000250 | NEG | NEG | NEG | NEG | NEG | NEG | NEG | NEG | NEG | NEG | NEG | NEG | POS | NEG | NEG | NEG | NEG | NEG | NEG | NEG | NEG | NEG | NEG | NEG | NEG | NEG | NEG | NEG | NEG | NEG | NEG | NEG | NEG | NEG |
| Freiburg-1023-72.1023-DBK0000271 | NEG | NEG | NEG | NEG | NEG | NEG | NEG | NEG | NEG | NEG | NEG | NEG | POS | NEG | NEG | NEG | NEG | NEG | NEG | NEG | NEG | NEG | NEG | NEG | NEG | NEG | NEG | NEG | NEG | NEG | NEG | NEG | NEG | NEG |
| | NEG | NEG | NEG | NEG | NEG | NEG | NEG | NEG | NEG | NEG | NEG | NEG | POS | NEG | NEG | NEG | NEG | NEG | NEG | NEG | NEG | NEG | NEG | NEG | NEG | NEG | NEG | NEG | NEG | NEG | NEG | NEG | NEG | NEG |
| Freiburg-1043-74.1043-DBK0000268 | NEG | NEG | NEG | NEG | NEG | NEG | NEG | NEG | NEG | NEG | NEG | AMB | POS | NEG | NEG | NEG | NEG | NEG | NEG | NEG | NEG | NEG | NEG | NEG | NEG | NEG | NEG | NEG | NEG | NEG | NEG | NEG | NEG | NEG |
| Freiburg-633-2.663-DBK0000292 | NEG | NEG | NEG | NEG | NEG | NEG | NEG | NEG | NEG | NEG | NEG | NEG | POS | NEG | NEG | NEG | NEG | NEG | NEG | NEG | NEG | NEG | NEG | NEG | NEG | NEG | NEG | NEG | NEG | NEG | NEG | NEG | NEG | NEG |
| | NEG | NEG | NEG | NEG | NEG | NEG | NEG | NEG | NEG | NEG | NEG | NEG | POS | NEG | NEG | NEG | NEG | NEG | NEG | NEG | NEG | NEG | NEG | NEG | NEG | NEG | NEG | NEG | NEG | NEG | NEG | NEG | NEG | NEG |
| Freiburg-782-20.782-DBK0000276 | NEG | NEG | NEG | NEG | NEG | NEG | NEG | NEG | NEG | NEG | NEG | NEG | POS | NEG | NEG | NEG | NEG | NEG | NEG | NEG | NEG | NEG | NEG | NEG | NEG | NEG | NEG | NEG | NEG | NEG | NEG | NEG | NEG | NEG |
| | NEG | NEG | NEG | NEG | NEG | NEG | NEG | NEG | NEG | NEG | NEG | NEG | AMB | NEG | NEG | NEG | NEG | NEG | NEG | NEG | NEG | NEG | NEG | NEG | NEG | NEG | NEG | NEG | NEG | NEG | NEG | NEG | NEG | NEG |
| **>HP-05** | NEG | NEG | NEG | NEG | NEG | NEG | NEG | NEG | NEG | NEG | NEG | NEG | NEG | POS | POS | NEG | NEG | NEG | NEG | NEG | NEG | NEG | NEG | NEG | NEG | NEG | NEG | NEG | NEG | NEG | NEG | NEG | NEG | NEG |
| Freiburg-740-17.74-DBK0000234 | NEG | NEG | NEG | NEG | NEG | NEG | NEG | NEG | NEG | NEG | NEG | NEG | NEG | POS | POS | NEG | NEG | NEG | NEG | NEG | NEG | NEG | NEG | NEG | NEG | NEG | NEG | NEG | NEG | NEG | NEG | NEG | NEG | NEG |
| | NEG | NEG | NEG | NEG | NEG | NEG | NEG | NEG | NEG | NEG | NEG | NEG | NEG | POS | POS | NEG | NEG | NEG | NEG | NEG | NEG | NEG | NEG | NEG | NEG | NEG | NEG | NEG | NEG | NEG | NEG | NEG | NEG | NEG |
| Freiburg-793-23.793-DBK0000286 | NEG | NEG | NEG | NEG | NEG | NEG | NEG | NEG | NEG | NEG | NEG | NEG | NEG | POS | POS | NEG | NEG | NEG | NEG | NEG | NEG | NEG | NEG | NEG | NEG | NEG | NEG | NEG | NEG | NEG | NEG | NEG | NEG | NEG |
| | NEG | NEG | NEG | NEG | NEG | NEG | NEG | NEG | NEG | NEG | NEG | NEG | NEG | POS | POS | NEG | NEG | NEG | NEG | NEG | NEG | NEG | NEG | NEG | NEG | NEG | NEG | NEG | NEG | NEG | NEG | NEG | NEG | NEG |
| **>HP-06** | NEG | NEG | NEG | NEG | NEG | NEG | NEG | NEG | NEG | NEG | NEG | NEG | NEG | NEG | NEG | NEG | NEG | NEG | NEG | NEG | NEG | NEG | NEG | NEG | NEG | NEG | NEG | NEG | NEG | NEG | NEG | NEG | NEG | NEG |
| Freiburg-874-35.874-DBK0000282 | NEG | NEG | NEG | NEG | NEG | NEG | NEG | NEG | NEG | NEG | NEG | NEG | NEG | NEG | NEG | NEG | NEG | NEG | NEG | NEG | NEG | NEG | NEG | NEG | NEG | NEG | NEG | NEG | NEG | NEG | NEG | NEG | NEG | NEG |
| | NEG | NEG | NEG | NEG | NEG | NEG | NEG | NEG | NEG | NEG | NEG | NEG | NEG | NEG | NEG | NEG | NEG | NEG | NEG | NEG | NEG | NEG | NEG | NEG | NEG | NEG | NEG | NEG | NEG | NEG | NEG | NEG | NEG | NEG |

| STRAIN / ISOLATE | splA Surface layer protein A, cont. | | | | | | | | | | | | | | | | | | | | | | | | | | | | | | | | |
|---|---|---|---|---|---|---|---|---|---|---|---|---|---|---|---|---|---|---|---|---|---|---|---|---|---|---|---|---|---|---|---|---|---|
| | hp1180\|slpA | hp1182\|slpA | hp1183\|slpA | hp1184\|slpA | hp1186\|slpA | hp1188\|slpA | hp1190\|slpA | hp1191\|slpA | hp1192\|slpA | hp1193\|slpA | hp1195\|slpA | hp1197\|slpA | hp1198\|slpA | hp1199\|slpA | hp1200\|slpA | hp1201\|slpA | hp1202\|slpA | hp1203\|slpA | hp1204\|slpA | hp1205\|slpA | hp1206\|slpA | hp1208\|slpA | hp1209\|slpA | hp1210\|slpA | hp1211\|slpA | hp1232\|slpA | hp1233\|slpA | hp1234\|slpA | hp1236\|slpA | hp1237\|slpA | hp1239\|slpA | hp1242\|slpA | hp1243\|slpA | hp1249\|slpA |
| >HP-07 | NEG | NEG | NEG | NEG | NEG | NEG | NEG | NEG | NEG | NEG | NEG | NEG | NEG | NEG | NEG | NEG | NEG | NEG | NEG | NEG | NEG | NEG | NEG | NEG | NEG | NEG | NEG | NEG | NEG | NEG | NEG | NEG | NEG | NEG |
| Freiburg-923-44.923-DBK0000238 | NEG | AMB | NEG | NEG | NEG | NEG | NEG | NEG | NEG | NEG | NEG | NEG | NEG | NEG | NEG | NEG | NEG | NEG | NEG | NEG | NEG | NEG | NEG | NEG | NEG | NEG | NEG | NEG | NEG | NEG | NEG | NEG | NEG | NEG |
| | NEG | NEG | NEG | NEG | NEG | NEG | NEG | NEG | NEG | NEG | NEG | NEG | NEG | NEG | NEG | NEG | NEG | NEG | NEG | NEG | NEG | NEG | NEG | NEG | NEG | NEG | NEG | NEG | NEG | NEG | NEG | NEG | NEG | NEG |
| >HP-08 | NEG | NEG | NEG | NEG | NEG | NEG | NEG | NEG | NEG | NEG | NEG | NEG | NEG | NEG | NEG | NEG | NEG | NEG | NEG | NEG | NEG | NEG | NEG | NEG | NEG | POS | NEG | NEG | NEG | NEG | POS | NEG | NEG | NEG |
| Freiburg-1021-71.1021-DBK0000248 | NEG | NEG | NEG | NEG | NEG | NEG | NEG | NEG | NEG | NEG | NEG | NEG | NEG | NEG | NEG | NEG | NEG | NEG | NEG | NEG | NEG | NEG | NEG | NEG | NEG | POS | NEG | NEG | NEG | NEG | POS | NEG | NEG | NEG |
| | NEG | NEG | NEG | NEG | NEG | NEG | NEG | NEG | NEG | NEG | NEG | NEG | NEG | NEG | NEG | NEG | NEG | NEG | NEG | NEG | NEG | NEG | NEG | NEG | NEG | POS | NEG | NEG | NEG | NEG | POS | NEG | NEG | NEG |
| Freiburg-669-9.669-DBK0000231 | NEG | NEG | NEG | NEG | NEG | NEG | NEG | NEG | NEG | NEG | NEG | NEG | NEG | NEG | NEG | NEG | NEG | NEG | NEG | NEG | NEG | NEG | NEG | NEG | NEG | POS | NEG | NEG | NEG | NEG | POS | NEG | NEG | NEG |
| | NEG | NEG | NEG | NEG | NEG | NEG | NEG | NEG | NEG | NEG | NEG | NEG | NEG | NEG | NEG | NEG | NEG | NEG | NEG | NEG | NEG | NEG | NEG | NEG | NEG | POS | NEG | NEG | NEG | NEG | POS | NEG | NEG | NEG |
| Freiburg-964-53.964-DBK0000267 | NEG | NEG | NEG | NEG | NEG | NEG | NEG | NEG | NEG | NEG | NEG | NEG | NEG | NEG | NEG | NEG | NEG | NEG | NEG | NEG | NEG | NEG | NEG | NEG | NEG | POS | NEG | NEG | NEG | NEG | POS | NEG | NEG | NEG |
| | NEG | NEG | NEG | NEG | NEG | NEG | NEG | NEG | NEG | NEG | NEG | NEG | NEG | NEG | NEG | NEG | NEG | NEG | NEG | NEG | NEG | NEG | NEG | NEG | NEG | POS | NEG | NEG | NEG | NEG | POS | NEG | NEG | NEG |
| Freiburg-899-41.899-DBK0000224 | NEG | NEG | NEG | NEG | NEG | NEG | NEG | NEG | NEG | NEG | NEG | NEG | NEG | NEG | NEG | NEG | NEG | NEG | NEG | NEG | NEG | NEG | NEG | NEG | NEG | POS | NEG | NEG | NEG | NEG | POS | NEG | NEG | NEG |
| | NEG | NEG | NEG | NEG | NEG | NEG | NEG | NEG | NEG | NEG | NEG | NEG | NEG | NEG | NEG | NEG | NEG | NEG | NEG | NEG | NEG | NEG | NEG | NEG | NEG | POS | NEG | NEG | NEG | NEG | POS | NEG | NEG | NEG |
| Freiburg-982-58.982-DBK0000257 | NEG | NEG | NEG | NEG | NEG | NEG | NEG | NEG | NEG | NEG | NEG | NEG | NEG | NEG | NEG | NEG | NEG | NEG | NEG | NEG | NEG | NEG | NEG | NEG | NEG | POS | NEG | NEG | NEG | NEG | POS | NEG | NEG | NEG |
| | NEG | NEG | NEG | NEG | NEG | NEG | NEG | NEG | NEG | NEG | NEG | NEG | NEG | NEG | NEG | NEG | NEG | NEG | NEG | NEG | NEG | NEG | NEG | NEG | NEG | POS | NEG | NEG | NEG | NEG | AMB | NEG | NEG | NEG |
| Dresden 07_7035 | NEG | NEG | NEG | NEG | NEG | NEG | NEG | NEG | NEG | NEG | NEG | NEG | NEG | NEG | NEG | NEG | NEG | NEG | NEG | NEG | NEG | NEG | NEG | NEG | NEG | POS | NEG | NEG | NEG | NEG | POS | NEG | NEG | NEG |
| Dresden 07_7248 | NEG | NEG | NEG | NEG | NEG | NEG | NEG | NEG | NEG | NEG | NEG | NEG | NEG | NEG | NEG | NEG | NEG | NEG | NEG | NEG | NEG | NEG | NEG | NEG | NEG | POS | NEG | NEG | NEG | NEG | POS | NEG | NEG | NEG |
| Dresden 07_8702 | NEG | NEG | NEG | NEG | NEG | NEG | NEG | NEG | NEG | NEG | NEG | NEG | NEG | NEG | NEG | NEG | NEG | NEG | NEG | NEG | NEG | NEG | NEG | NEG | NEG | POS | NEG | NEG | NEG | NEG | POS | NEG | NEG | NEG |
| Dresden 07_8989 | NEG | NEG | NEG | NEG | NEG | NEG | NEG | NEG | NEG | NEG | NEG | NEG | NEG | NEG | NEG | NEG | NEG | NEG | NEG | NEG | NEG | NEG | NEG | NEG | NEG | POS | NEG | NEG | NEG | NEG | POS | NEG | NEG | NEG |
| Dresden 08_0712 | NEG | NEG | NEG | NEG | NEG | NEG | NEG | NEG | NEG | NEG | NEG | NEG | NEG | NEG | NEG | NEG | NEG | NEG | NEG | NEG | NEG | NEG | NEG | NEG | NEG | POS | NEG | NEG | NEG | NEG | POS | NEG | NEG | NEG |
| >HP-09 | NEG | NEG | NEG | NEG | NEG | NEG | NEG | POS | NEG | NEG | NEG | NEG | NEG | NEG | NEG | NEG | NEG | NEG | NEG | NEG | NEG | NEG | NEG | NEG | NEG | NEG | NEG | NEG | NEG | NEG | NEG | NEG | NEG | NEG |
| Freiburg-1056-79.1056-DBK0000261 | NEG | NEG | NEG | NEG | NEG | NEG | NEG | POS | NEG | NEG | NEG | NEG | NEG | NEG | NEG | NEG | NEG | NEG | NEG | NEG | NEG | NEG | NEG | NEG | NEG | NEG | NEG | NEG | NEG | NEG | NEG | NEG | NEG | NEG |
| Freiburg-658-8.658-DBK0000230 | NEG | NEG | NEG | NEG | NEG | NEG | NEG | POS | NEG | NEG | NEG | AMB | NEG | NEG | NEG | NEG | NEG | NEG | NEG | NEG | NEG | NEG | NEG | NEG | NEG | NEG | NEG | NEG | NEG | NEG | NEG | NEG | NEG | NEG |
| | NEG | NEG | NEG | NEG | NEG | NEG | NEG | POS | NEG | NEG | NEG | NEG | NEG | NEG | NEG | NEG | NEG | NEG | NEG | NEG | NEG | NEG | NEG | NEG | NEG | NEG | NEG | NEG | NEG | NEG | NEG | NEG | NEG | NEG |
| Freiburg-963-52.963-DBK0000265 | NEG | NEG | NEG | NEG | NEG | NEG | NEG | POS | NEG | NEG | NEG | NEG | NEG | NEG | NEG | NEG | NEG | NEG | NEG | NEG | NEG | NEG | NEG | NEG | NEG | NEG | NEG | NEG | NEG | NEG | NEG | NEG | NEG | NEG |
| | NEG | NEG | NEG | NEG | NEG | NEG | NEG | POS | NEG | NEG | NEG | NEG | NEG | NEG | NEG | NEG | NEG | NEG | NEG | NEG | NEG | NEG | NEG | NEG | NEG | NEG | NEG | NEG | NEG | NEG | NEG | NEG | NEG | NEG |
| Freiburg-988-63.988-DBK0000253 | NEG | NEG | NEG | NEG | NEG | NEG | NEG | POS | NEG | NEG | NEG | NEG | NEG | NEG | NEG | NEG | NEG | NEG | NEG | NEG | NEG | NEG | NEG | NEG | NEG | NEG | NEG | NEG | NEG | NEG | NEG | NEG | NEG | NEG |
| | NEG | NEG | NEG | NEG | NEG | NEG | NEG | AMB | NEG | NEG | NEG | NEG | NEG | NEG | NEG | NEG | NEG | NEG | NEG | NEG | NEG | NEG | NEG | NEG | NEG | NEG | NEG | NEG | NEG | NEG | NEG | NEG | NEG | NEG |
| Freiburg_79.1057 | NEG | NEG | NEG | NEG | NEG | NEG | NEG | POS | NEG | NEG | NEG | NEG | NEG | NEG | NEG | NEG | NEG | NEG | NEG | NEG | NEG | NEG | NEG | NEG | NEG | NEG | NEG | NEG | NEG | NEG | NEG | NEG | NEG | NEG |
| Jena_S0017.11 | NEG | NEG | NEG | NEG | NEG | NEG | NEG | AMB | NEG | NEG | NEG | NEG | NEG | NEG | NEG | NEG | NEG | NEG | NEG | NEG | NEG | NEG | NEG | NEG | NEG | NEG | NEG | NEG | NEG | NEG | NEG | NEG | NEG | NEG |
| | NEG | NEG | NEG | NEG | NEG | NEG | NEG | POS | NEG | NEG | NEG | NEG | NEG | NEG | NEG | NEG | NEG | NEG | NEG | NEG | NEG | NEG | NEG | NEG | NEG | NEG | NEG | NEG | NEG | NEG | NEG | NEG | NEG | NEG |
| | NEG | NEG | NEG | NEG | NEG | NEG | NEG | NEG | NEG | NEG | NEG | NEG | NEG | NEG | NEG | NEG | NEG | NEG | NEG | NEG | NEG | NEG | NEG | NEG | NEG | NEG | NEG | NEG | NEG | NEG | NEG | NEG | NEG | NEG |
| | NEG | NEG | NEG | NEG | NEG | NEG | NEG | NEG | NEG | NEG | NEG | AMB | NEG | NEG | NEG | NEG | NEG | NEG | NEG | NEG | NEG | NEG | NEG | NEG | NEG | NEG | NEG | NEG | NEG | NEG | NEG | NEG | NEG | NEG |
| | NEG | NEG | NEG | NEG | NEG | NEG | NEG | POS | NEG | NEG | NEG | NEG | NEG | NEG | NEG | NEG | NEG | NEG | NEG | NEG | NEG | NEG | NEG | NEG | NEG | NEG | NEG | NEG | NEG | NEG | NEG | NEG | NEG | NEG |
| Jena_S0015_11 | NEG | NEG | NEG | NEG | NEG | NEG | NEG | NEG | NEG | NEG | NEG | NEG | NEG | NEG | NEG | NEG | NEG | NEG | NEG | NEG | NEG | NEG | NEG | NEG | NEG | NEG | NEG | NEG | NEG | NEG | NEG | NEG | NEG | NEG |
| | NEG | NEG | NEG | NEG | NEG | NEG | NEG | NEG | NEG | NEG | NEG | NEG | NEG | NEG | NEG | NEG | NEG | NEG | NEG | NEG | NEG | NEG | NEG | NEG | NEG | NEG | NEG | NEG | NEG | NEG | NEG | NEG | NEG | NEG |
| | NEG | NEG | NEG | NEG | NEG | NEG | NEG | NEG | NEG | NEG | NEG | NEG | NEG | NEG | NEG | NEG | NEG | NEG | NEG | NEG | NEG | NEG | NEG | NEG | NEG | NEG | NEG | NEG | NEG | NEG | NEG | NEG | NEG | NEG |
| Jena_S0012_11 | NEG | NEG | NEG | AMB | NEG | NEG | NEG | AMB | NEG | NEG | NEG | NEG | NEG | NEG | NEG | NEG | NEG | NEG | NEG | NEG | NEG | NEG | NEG | NEG | NEG | NEG | NEG | NEG | NEG | NEG | NEG | NEG | NEG | NEG |
| | NEG | NEG | NEG | NEG | NEG | NEG | NEG | AMB | NEG | NEG | NEG | NEG | NEG | NEG | NEG | NEG | NEG | NEG | NEG | NEG | NEG | NEG | NEG | NEG | NEG | NEG | NEG | NEG | NEG | NEG | NEG | NEG | NEG | NEG |
| Dresden 07_7363 | NEG | NEG | NEG | NEG | NEG | NEG | NEG | POS | NEG | NEG | NEG | NEG | NEG | NEG | NEG | NEG | NEG | NEG | NEG | NEG | NEG | NEG | NEG | NEG | NEG | NEG | NEG | NEG | NEG | NEG | NEG | NEG | NEG | NEG |
| Dresden 07_8174 | NEG | NEG | NEG | NEG | NEG | NEG | NEG | AMB | NEG | NEG | NEG | NEG | NEG | NEG | NEG | NEG | NEG | NEG | NEG | NEG | NEG | NEG | NEG | NEG | NEG | NEG | NEG | NEG | NEG | NEG | NEG | NEG | NEG | NEG |
| Dresden 07_8224 | NEG | NEG | NEG | NEG | NEG | NEG | NEG | AMB | NEG | NEG | NEG | NEG | NEG | NEG | NEG | NEG | NEG | NEG | NEG | NEG | NEG | NEG | NEG | NEG | NEG | NEG | NEG | NEG | NEG | NEG | NEG | NEG | NEG | NEG |
| Dresden 07_8562 | NEG | NEG | NEG | NEG | NEG | NEG | NEG | POS | NEG | NEG | NEG | NEG | NEG | NEG | NEG | NEG | NEG | NEG | NEG | NEG | NEG | NEG | NEG | NEG | NEG | NEG | NEG | NEG | NEG | NEG | NEG | NEG | NEG | NEG |
| Dresden 08_613 | NEG | NEG | NEG | NEG | NEG | NEG | AMB | POS | NEG | NEG | NEG | NEG | NEG | NEG | NEG | NEG | NEG | NEG | NEG | NEG | NEG | NEG | NEG | NEG | NEG | NEG | NEG | NEG | NEG | NEG | NEG | NEG | NEG | NEG |
| Dresden 08_0422 | NEG | NEG | NEG | NEG | NEG | NEG | NEG | NEG | NEG | NEG | NEG | NEG | NEG | NEG | NEG | NEG | NEG | NEG | NEG | NEG | NEG | NEG | NEG | NEG | NEG | NEG | NEG | NEG | NEG | NEG | NEG | NEG | NEG | NEG |
| Dresden 08_0837 | NEG | NEG | NEG | NEG | NEG | NEG | NEG | AMB | NEG | NEG | NEG | NEG | NEG | NEG | NEG | NEG | NEG | NEG | NEG | NEG | NEG | NEG | NEG | NEG | NEG | NEG | NEG | NEG | NEG | NEG | NEG | NEG | NEG | NEG |
| Dresden 08_0878 | NEG | NEG | NEG | NEG | NEG | NEG | NEG | NEG | NEG | NEG | NEG | NEG | NEG | NEG | NEG | NEG | NEG | NEG | NEG | NEG | NEG | NEG | NEG | NEG | NEG | NEG | NEG | NEG | NEG | NEG | NEG | NEG | NEG | NEG |
| Dresden 08_1484 | NEG | NEG | NEG | NEG | NEG | NEG | NEG | POS | NEG | NEG | NEG | NEG | NEG | NEG | NEG | NEG | NEG | NEG | NEG | NEG | NEG | NEG | NEG | NEG | NEG | NEG | NEG | NEG | NEG | NEG | NEG | NEG | NEG | NEG |
| >HP-10 | NEG | NEG | NEG | NEG | NEG | NEG | NEG | NEG | NEG | NEG | NEG | NEG | NEG | NEG | NEG | NEG | NEG | NEG | NEG | NEG | NEG | NEG | NEG | NEG | NEG | NEG | NEG | NEG | NEG | NEG | NEG | NEG | NEG | NEG |
| Dresden 09_664_1 | NEG | NEG | NEG | NEG | NEG | NEG | NEG | NEG | NEG | NEG | NEG | NEG | NEG | NEG | NEG | NEG | NEG | NEG | NEG | NEG | NEG | NEG | NEG | NEG | NEG | NEG | NEG | NEG | NEG | NEG | NEG | NEG | NEG | NEG |
| Dresden 09_664_2 | NEG | NEG | NEG | NEG | NEG | NEG | NEG | NEG | NEG | NEG | NEG | NEG | NEG | NEG | NEG | NEG | NEG | NEG | NEG | NEG | NEG | NEG | NEG | NEG | NEG | NEG | NEG | NEG | NEG | NEG | NEG | NEG | NEG | NEG |
| Dresden 09_711_1 | NEG | NEG | NEG | NEG | NEG | NEG | NEG | NEG | NEG | NEG | NEG | NEG | NEG | NEG | NEG | NEG | NEG | NEG | NEG | NEG | NEG | NEG | NEG | NEG | NEG | NEG | NEG | NEG | NEG | NEG | NEG | NEG | NEG | NEG |
| Dresden 09_711_2 | NEG | NEG | NEG | NEG | NEG | NEG | NEG | NEG | NEG | NEG | NEG | NEG | NEG | NEG | NEG | NEG | NEG | NEG | NEG | NEG | NEG | NEG | NEG | NEG | NEG | NEG | NEG | NEG | NEG | NEG | NEG | NEG | NEG | NEG |
| Dresden 09_932 | NEG | NEG | NEG | NEG | NEG | NEG | NEG | NEG | NEG | NEG | NEG | NEG | NEG | NEG | NEG | NEG | NEG | NEG | NEG | NEG | NEG | NEG | NEG | NEG | NEG | NEG | NEG | NEG | NEG | NEG | NEG | NEG | NEG | NEG |
| Dresden 09_939 | NEG | NEG | NEG | NEG | NEG | NEG | NEG | NEG | NEG | NEG | NEG | NEG | NEG | NEG | NEG | NEG | NEG | NEG | NEG | NEG | NEG | NEG | NEG | NEG | NEG | NEG | NEG | NEG | NEG | NEG | NEG | NEG | NEG | NEG |
| Dresden 09_948 | NEG | NEG | NEG | NEG | NEG | NEG | NEG | NEG | NEG | NEG | NEG | NEG | NEG | NEG | NEG | NEG | NEG | NEG | NEG | NEG | NEG | NEG | NEG | NEG | NEG | NEG | NEG | NEG | NEG | NEG | NEG | NEG | NEG | NEG |
| >HP-11 | NEG | NEG | NEG | NEG | NEG | NEG | NEG | NEG | NEG | NEG | NEG | NEG | NEG | NEG | NEG | NEG | NEG | NEG | NEG | NEG | NEG | NEG | NEG | NEG | NEG | NEG | NEG | NEG | NEG | NEG | NEG | NEG | NEG | NEG |
| C. difficile CD37 (GenBank AHJJ) in silico prediction | NEG | NEG | NEG | NEG | NEG | NEG | NEG | NEG | NEG | NEG | NEG | NEG | NEG | NEG | NEG | NEG | NEG | NEG | NEG | NEG | NEG | NEG | NEG | NEG | NEG | NEG | NEG | NEG | NEG | NEG | NEG | NEG | NEG | NEG |
| Freiburg-684-10.684-DBK0000240 | NEG | NEG | NEG | NEG | NEG | NEG | NEG | NEG | NEG | NEG | NEG | NEG | NEG | NEG | NEG | NEG | NEG | NEG | NEG | NEG | NEG | NEG | NEG | NEG | NEG | NEG | NEG | NEG | NEG | NEG | NEG | NEG | NEG | NEG |
| Freiburg-838-28.838-DBK0000278 | NEG | NEG | NEG | NEG | NEG | NEG | NEG | NEG | NEG | NEG | NEG | NEG | NEG | NEG | NEG | NEG | NEG | NEG | NEG | NEG | NEG | NEG | NEG | NEG | NEG | NEG | NEG | NEG | NEG | NEG | NEG | NEG | NEG | NEG |
| | NEG | AMB | NEG | NEG | NEG | NEG | NEG | NEG | NEG | NEG | NEG | NEG | NEG | NEG | NEG | NEG | NEG | NEG | NEG | NEG | NEG | NEG | NEG | NEG | NEG | NEG | NEG | NEG | NEG | NEG | NEG | NEG | NEG | NEG |
| >HP-12 | NEG | NEG | NEG | NEG | NEG | NEG | NEG | NEG | NEG | POS | NEG | NEG | NEG | NEG | NEG | NEG | POS | NEG | NEG | NEG | NEG | NEG | NEG | NEG | NEG | NEG | NEG | NEG | NEG | NEG | NEG | NEG | NEG | NEG |
| Freiburg-1007-68.1007-DBK0000252 | NEG | NEG | NEG | NEG | NEG | NEG | NEG | NEG | NEG | POS | NEG | NEG | NEG | NEG | NEG | NEG | POS | NEG | NEG | NEG | NEG | NEG | NEG | NEG | NEG | NEG | NEG | NEG | NEG | NEG | NEG | NEG | NEG | NEG |
| | NEG | NEG | NEG | NEG | NEG | NEG | NEG | NEG | NEG | AMB | NEG | NEG | NEG | NEG | NEG | NEG | POS | NEG | NEG | NEG | NEG | NEG | NEG | NEG | NEG | NEG | NEG | NEG | NEG | NEG | NEG | NEG | NEG | NEG |
| Freiburg-983-59.983-DBK0000256 | NEG | NEG | NEG | NEG | NEG | NEG | NEG | NEG | NEG | POS | NEG | NEG | NEG | NEG | NEG | NEG | POS | NEG | NEG | NEG | NEG | NEG | NEG | NEG | NEG | NEG | NEG | NEG | NEG | NEG | NEG | NEG | NEG | NEG |
| | NEG | NEG | NEG | NEG | NEG | NEG | NEG | NEG | NEG | POS | NEG | NEG | NEG | NEG | NEG | NEG | POS | NEG | NEG | NEG | NEG | NEG | NEG | NEG | NEG | NEG | NEG | NEG | NEG | NEG | NEG | NEG | NEG | NEG |
| >HP-13 | NEG | NEG | NEG | NEG | NEG | NEG | NEG | NEG | NEG | NEG | NEG | NEG | NEG | NEG | NEG | NEG | NEG | NEG | NEG | NEG | NEG | NEG | POS | POS | NEG | NEG | NEG | NEG | NEG | NEG | NEG | NEG | NEG | NEG |
| C. difficile 70-100-2010 (GenBank AGAC) in silico prediction | NEG | NEG | NEG | NEG | NEG | NEG | NEG | NEG | NEG | NEG | NEG | NEG | NEG | NEG | NEG | NEG | NEG | NEG | NEG | NEG | NEG | NEG | POS | POS | NEG | NEG | NEG | NEG | NEG | NEG | NEG | NEG | NEG | NEG |
| Freiburg-1044-75.1044-DBK0000270 | NEG | NEG | NEG | NEG | NEG | NEG | NEG | NEG | NEG | NEG | NEG | NEG | NEG | NEG | NEG | NEG | NEG | NEG | NEG | NEG | NEG | NEG | POS | POS | NEG | NEG | NEG | NEG | NEG | NEG | NEG | NEG | NEG | NEG |
| | NEG | NEG | NEG | NEG | NEG | NEG | NEG | NEG | NEG | NEG | NEG | NEG | NEG | NEG | NEG | NEG | NEG | NEG | NEG | NEG | NEG | NEG | POS | POS | NEG | NEG | NEG | NEG | NEG | NEG | NEG | NEG | NEG | NEG |
| Freiburg-1057-80.1057-DBK0000260 | NEG | POS | NEG | NEG | NEG | NEG | NEG | NEG | NEG | NEG | NEG | NEG | NEG | NEG | NEG | NEG | NEG | NEG | NEG | NEG | NEG | NEG | POS | POS | NEG | NEG | NEG | NEG | NEG | NEG | NEG | NEG | NEG | NEG |
| | NEG | NEG | NEG | NEG | NEG | NEG | NEG | NEG | NEG | NEG | NEG | NEG | NEG | NEG | NEG | NEG | NEG | NEG | NEG | NEG | NEG | NEG | POS | POS | NEG | NEG | NEG | NEG | NEG | NEG | NEG | NEG | NEG | NEG |
| Freiburg-710-12.710-DBK0000241 | NEG | NEG | NEG | NEG | NEG | NEG | NEG | NEG | NEG | NEG | NEG | NEG | NEG | NEG | NEG | NEG | NEG | NEG | NEG | NEG | NEG | NEG | POS | POS | NEG | NEG | NEG | NEG | NEG | NEG | NEG | NEG | NEG | NEG |
| | NEG | NEG | NEG | NEG | NEG | NEG | NEG | NEG | NEG | NEG | NEG | NEG | NEG | NEG | NEG | NEG | NEG | NEG | NEG | NEG | NEG | NEG | POS | POS | NEG | NEG | NEG | NEG | NEG | NEG | NEG | NEG | NEG | NEG |
| Freiburg-940-48.94-DBK0000244 | NEG | NEG | NEG | NEG | NEG | NEG | NEG | NEG | NEG | NEG | NEG | NEG | NEG | NEG | NEG | NEG | NEG | NEG | NEG | NEG | NEG | NEG | POS | POS | NEG | NEG | NEG | NEG | NEG | NEG | NEG | NEG | NEG | NEG |
| | NEG | NEG | NEG | NEG | NEG | NEG | NEG | NEG | NEG | NEG | NEG | NEG | NEG | NEG | NEG | NEG | NEG | NEG | NEG | NEG | NEG | NEG | POS | POS | NEG | NEG | NEG | NEG | NEG | NEG | NEG | NEG | NEG | NEG |
| Freiburg-980-56.98-DBK0000246 | NEG | NEG | NEG | NEG | NEG | NEG | NEG | NEG | NEG | NEG | NEG | NEG | NEG | NEG | NEG | NEG | NEG | NEG | NEG | NEG | NEG | NEG | POS | POS | NEG | NEG | NEG | NEG | NEG | NEG | NEG | NEG | NEG | NEG |
| | NEG | NEG | NEG | NEG | NEG | NEG | NEG | NEG | NEG | NEG | NEG | NEG | NEG | NEG | NEG | NEG | NEG | NEG | NEG | NEG | NEG | NEG | POS | POS | NEG | NEG | NEG | NEG | NEG | NEG | NEG | NEG | NEG | NEG |
| Jena_S0014_11 | NEG | NEG | NEG | NEG | NEG | NEG | NEG | NEG | NEG | NEG | NEG | NEG | NEG | NEG | NEG | NEG | NEG | NEG | NEG | NEG | NEG | NEG | POS | POS | NEG | NEG | NEG | NEG | NEG | NEG | NEG | NEG | NEG | NEG |
| | NEG | NEG | NEG | NEG | NEG | NEG | NEG | NEG | NEG | NEG | NEG | NEG | NEG | NEG | NEG | NEG | NEG | NEG | NEG | NEG | NEG | NEG | POS | POS | NEG | NEG | NEG | NEG | NEG | NEG | NEG | NEG | NEG | NEG |
| | NEG | NEG | NEG | NEG | NEG | NEG | NEG | NEG | NEG | NEG | NEG | NEG | NEG | NEG | NEG | NEG | NEG | NEG | NEG | NEG | NEG | NEG | POS | POS | NEG | NEG | NEG | NEG | NEG | NEG | NEG | NEG | NEG | NEG |
| Jena_S0023_11 | NEG | NEG | NEG | NEG | NEG | NEG | NEG | NEG | NEG | NEG | NEG | NEG | NEG | NEG | NEG | NEG | NEG | NEG | NEG | NEG | NEG | NEG | POS | POS | NEG | NEG | NEG | NEG | NEG | NEG | NEG | NEG | NEG | NEG |
| | NEG | NEG | NEG | NEG | NEG | NEG | NEG | NEG | NEG | NEG | NEG | NEG | NEG | NEG | NEG | NEG | NEG | NEG | NEG | NEG | NEG | NEG | POS | POS | NEG | NEG | NEG | NEG | NEG | NEG | NEG | NEG | NEG | NEG |
| | NEG | NEG | NEG | NEG | NEG | NEG | NEG | NEG | NEG | NEG | NEG | NEG | NEG | NEG | NEG | NEG | NEG | NEG | NEG | NEG | NEG | NEG | POS | AMB | NEG | NEG | NEG | NEG | NEG | NEG | NEG | NEG | NEG | NEG |
| Dresden 07_7779 | NEG | NEG | NEG | NEG | NEG | NEG | NEG | NEG | NEG | NEG | NEG | NEG | NEG | NEG | NEG | NEG | NEG | NEG | NEG | NEG | NEG | NEG | POS | POS | NEG | NEG | NEG | NEG | NEG | NEG | NEG | NEG | NEG | NEG |
| Dresden 07_7950 | NEG | NEG | NEG | NEG | NEG | NEG | NEG | NEG | NEG | NEG | NEG | NEG | NEG | NEG | NEG | NEG | NEG | NEG | NEG | NEG | NEG | NEG | POS | POS | NEG | NEG | NEG | NEG | NEG | NEG | NEG | NEG | NEG | NEG |
| Dresden 07_7953 | NEG | NEG | NEG | NEG | NEG | NEG | AMB | NEG | NEG | NEG | NEG | NEG | NEG | NEG | NEG | NEG | NEG | NEG | NEG | NEG | NEG | NEG | POS | POS | NEG | NEG | NEG | NEG | NEG | NEG | NEG | NEG | NEG | NEG |
| Dresden 07_8015 | NEG | NEG | NEG | NEG | NEG | NEG | NEG | NEG | NEG | NEG | NEG | NEG | NEG | NEG | NEG | NEG | NEG | NEG | NEG | NEG | NEG | NEG | POS | POS | NEG | NEG | NEG | NEG | NEG | NEG | NEG | NEG | NEG | NEG |
| Dresden 07_8205 | NEG | NEG | NEG | NEG | NEG | NEG | NEG | NEG | NEG | NEG | NEG | NEG | NEG | NEG | NEG | NEG | NEG | NEG | NEG | NEG | NEG | NEG | POS | POS | NEG | NEG | NEG | NEG | NEG | NEG | NEG | NEG | NEG | NEG |
| Dresden 07_8595 | NEG | NEG | NEG | NEG | NEG | NEG | NEG | NEG | NEG | NEG | NEG | NEG | NEG | NEG | NEG | NEG | NEG | NEG | NEG | NEG | NEG | NEG | POS | POS | NEG | NEG | NEG | NEG | NEG | NEG | NEG | NEG | NEG | NEG |
| Dresden 07_8613 | NEG | NEG | NEG | NEG | NEG | NEG | NEG | NEG | NEG | NEG | NEG | NEG | NEG | NEG | NEG | NEG | NEG | NEG | NEG | NEG | NEG | NEG | POS | POS | NEG | NEG | NEG | NEG | NEG | NEG | NEG | NEG | NEG | NEG |
| Dresden 07_8686 | NEG | NEG | NEG | NEG | NEG | NEG | NEG | NEG | NEG | NEG | NEG | NEG | NEG | NEG | NEG | NEG | NEG | NEG | NEG | NEG | NEG | NEG | POS | POS | NEG | NEG | NEG | NEG | NEG | NEG | NEG | NEG | NEG | NEG |
| Dresden 07_9180 | NEG | NEG | NEG | NEG | NEG | NEG | NEG | NEG | NEG | NEG | NEG | NEG | NEG | NEG | NEG | NEG | NEG | NEG | NEG | NEG | NEG | NEG | POS | POS | NEG | NEG | NEG | NEG | NEG | NEG | NEG | NEG | NEG | NEG |
| Dresden 08_0237 | NEG | NEG | NEG | NEG | NEG | NEG | NEG | NEG | NEG | NEG | NEG | NEG | NEG | NEG | NEG | NEG | NEG | NEG | NEG | NEG | NEG | NEG | POS | POS | NEG | NEG | NEG | NEG | NEG | NEG | NEG | NEG | NEG | NEG |
| Dresden 08_0492 | NEG | NEG | NEG | NEG | NEG | NEG | NEG | NEG | NEG | NEG | NEG | NEG | NEG | NEG | NEG | NEG | NEG | NEG | NEG | NEG | NEG | NEG | POS | AMB | NEG | NEG | NEG | NEG | NEG | NEG | NEG | NEG | NEG | NEG |
| Dresden 08_0992 | NEG | NEG | NEG | NEG | NEG | NEG | NEG | NEG | NEG | NEG | NEG | NEG | NEG | NEG | NEG | NEG | NEG | NEG | NEG | NEG | NEG | NEG | POS | AMB | NEG | NEG | NEG | NEG | NEG | NEG | NEG | NEG | NEG | NEG |
| Dresden 08_1184 | NEG | AMB | NEG | NEG | NEG | NEG | NEG | NEG | NEG | NEG | NEG | NEG | NEG | NEG | NEG | NEG | NEG | NEG | NEG | NEG | NEG | NEG | POS | POS | NEG | NEG | NEG | NEG | NEG | NEG | NEG | NEG | NEG | NEG |
| Dresden 08_1314 | NEG | NEG | NEG | NEG | NEG | NEG | NEG | NEG | NEG | NEG | NEG | NEG | NEG | NEG | NEG | NEG | NEG | NEG | NEG | NEG | NEG | NEG | POS | POS | NEG | NEG | NEG | NEG | NEG | NEG | NEG | NEG | NEG | NEG |
| Dresden 08_1640 | NEG | NEG | NEG | NEG | NEG | NEG | NEG | NEG | NEG | NEG | NEG | NEG | NEG | NEG | NEG | NEG | NEG | NEG | NEG | NEG | NEG | NEG | POS | POS | NEG | NEG | NEG | NEG | NEG | NEG | NEG | NEG | NEG | NEG |
| Dresden 08_1640 | NEG | NEG | NEG | NEG | NEG | NEG | NEG | NEG | NEG | NEG | NEG | NEG | NEG | NEG | NEG | NEG | NEG | NEG | NEG | NEG | NEG | NEG | POS | POS | NEG | NEG | NEG | NEG | NEG | NEG | NEG | NEG | NEG | NEG |
| Dresden 08_1758 | NEG | NEG | NEG | NEG | NEG | NEG | NEG | NEG | NEG | NEG | NEG | NEG | NEG | NEG | NEG | NEG | NEG | NEG | NEG | NEG | NEG | NEG | POS | POS | NEG | NEG | NEG | NEG | NEG | NEG | NEG | NEG | NEG | NEG |
| >HP-14 | NEG | NEG | POS | NEG | NEG | NEG | NEG | NEG | NEG | NEG | NEG | NEG | NEG | NEG | NEG | NEG | NEG | NEG | NEG | NEG | NEG | NEG | NEG | NEG | NEG | NEG | NEG | NEG | NEG | NEG | NEG | NEG | NEG | NEG |
| Freiburg-1019-70.1019-DBK0000255 | NEG | NEG | POS | NEG | NEG | NEG | NEG | NEG | NEG | NEG | NEG | NEG | NEG | NEG | NEG | NEG | NEG | NEG | NEG | NEG | NEG | NEG | NEG | NEG | NEG | NEG | NEG | NEG | NEG | NEG | NEG | NEG | NEG | NEG |
| >HP-15 | NEG | NEG | POS | NEG | NEG | NEG | NEG | NEG | NEG | NEG | NEG | NEG | NEG | NEG | NEG | NEG | NEG | NEG | NEG | NEG | NEG | NEG | NEG | NEG | NEG | NEG | NEG | NEG | NEG | NEG | NEG | NEG | NEG | NEG |
| Freiburg_42.907 | NEG | NEG | POS | NEG | NEG | NEG | NEG | NEG | NEG | NEG | NEG | NEG | NEG | NEG | NEG | NEG | NEG | NEG | NEG | NEG | NEG | NEG | NEG | NEG | NEG | NEG | NEG | NEG | NEG | NEG | NEG | NEG | NEG | NEG |
| >HP-16 | NEG | NEG | NEG | NEG | NEG | POS | NEG | NEG | NEG | NEG | NEG | NEG | NEG | NEG | NEG | NEG | NEG | NEG | NEG | NEG | NEG | NEG | NEG | NEG | NEG | NEG | NEG | NEG | NEG | NEG | NEG | NEG | NEG | NEG |
| Freiburg-978-55.978-DBK0000259 | NEG | NEG | NEG | NEG | NEG | POS | NEG | NEG | NEG | NEG | NEG | NEG | NEG | NEG | NEG | NEG | NEG | NEG | NEG | NEG | NEG | NEG | NEG | NEG | NEG | NEG | NEG | NEG | NEG | NEG | NEG | NEG | NEG | NEG |
| | NEG | NEG | NEG | NEG | NEG | POS | NEG | NEG | NEG | NEG | NEG | NEG | NEG | NEG | NEG | NEG | NEG | NEG | NEG | NEG | NEG | NEG | NEG | NEG | NEG | NEG | NEG | NEG | NEG | NEG | NEG | NEG | NEG | NEG |
| Dresden 08_1810 | NEG | NEG | NEG | NEG | NEG | POS | NEG | NEG | NEG | NEG | NEG | NEG | NEG | NEG | NEG | NEG | NEG | NEG | NEG | NEG | NEG | NEG | NEG | NEG | NEG | NEG | NEG | NEG | NEG | NEG | NEG | NEG | NEG | NEG |
| >HP-17 | NEG | NEG | NEG | NEG | NEG | NEG | NEG | NEG | NEG | NEG | NEG | NEG | NEG | NEG | NEG | NEG | NEG | NEG | NEG | POS | POS | NEG | NEG | NEG | NEG | NEG | NEG | NEG | NEG | NEG | NEG | NEG | NEG | NEG |
| Freiburg-729-15.729-DBK0000226 | NEG | NEG | NEG | NEG | NEG | NEG | NEG | NEG | NEG | NEG | NEG | NEG | NEG | NEG | NEG | NEG | NEG | NEG | NEG | AMB | NEG | NEG | NEG | NEG | NEG | NEG | NEG | NEG | NEG | NEG | NEG | NEG | NEG | NEG |
| | NEG | NEG | NEG | NEG | NEG | NEG | NEG | NEG | NEG | NEG | NEG | NEG | NEG | NEG | NEG | NEG | NEG | NEG | NEG | AMB | POS | NEG | NEG | NEG | NEG | NEG | NEG | NEG | NEG | NEG | NEG | NEG | NEG | NEG |
| Freiburg-926-45.926-DBK0000236 | NEG | NEG | NEG | NEG | NEG | NEG | NEG | NEG | NEG | NEG | NEG | NEG | NEG | NEG | NEG | NEG | NEG | NEG | NEG | POS | POS | NEG | NEG | NEG | NEG | NEG | NEG | NEG | NEG | NEG | NEG | NEG | NEG | NEG |
| | NEG | NEG | NEG | NEG | NEG | NEG | NEG | NEG | NEG | NEG | NEG | NEG | NEG | NEG | NEG | NEG | NEG | NEG | NEG | POS | POS | NEG | NEG | NEG | NEG | NEG | NEG | NEG | NEG | NEG | NEG | NEG | NEG | NEG |
| >HP-18 | NEG | NEG | NEG | NEG | NEG | NEG | NEG | NEG | NEG | NEG | NEG | NEG | NEG | NEG | NEG | NEG | NEG | NEG | POS | NEG | NEG | NEG | NEG | NEG | NEG | NEG | NEG | NEG | NEG | NEG | NEG | NEG | NEG | NEG |
| Freiburg-651-7.651-DBK0000229 | NEG | NEG | NEG | NEG | NEG | NEG | NEG | NEG | NEG | NEG | NEG | NEG | NEG | NEG | NEG | NEG | NEG | NEG | POS | NEG | NEG | NEG | NEG | NEG | NEG | NEG | NEG | NEG | NEG | NEG | NEG | NEG | NEG | NEG |
| | NEG | NEG | NEG | NEG | NEG | NEG | NEG | NEG | NEG | NEG | NEG | NEG | NEG | NEG | NEG | NEG | NEG | NEG | POS | NEG | NEG | NEG | NEG | NEG | NEG | NEG | NEG | NEG | NEG | NEG | NEG | NEG | NEG | NEG |
| Freiburg-725-13.725-DBK0000227 | NEG | NEG | NEG | NEG | NEG | NEG | NEG | NEG | NEG | NEG | NEG | NEG | NEG | NEG | NEG | NEG | NEG | NEG | AMB | NEG | NEG | NEG | NEG | NEG | NEG | NEG | NEG | NEG | NEG | NEG | NEG | NEG | NEG | NEG |
| | NEG | NEG | NEG | NEG | NEG | NEG | NEG | NEG | NEG | NEG | NEG | NEG | NEG | NEG | NEG | NEG | NEG | NEG | POS | NEG | NEG | NEG | NEG | NEG | NEG | NEG | NEG | NEG | NEG | NEG | NEG | NEG | NEG | NEG |
| Dresden 07_7176 | NEG | NEG | NEG | NEG | NEG | NEG | NEG | NEG | NEG | NEG | NEG | NEG | NEG | NEG | NEG | NEG | NEG | NEG | NEG | NEG | NEG | NEG | NEG | NEG | NEG | NEG | NEG | NEG | NEG | NEG | NEG | NEG | NEG | NEG |
| Dresden 07_7209 | NEG | AMB | NEG | NEG | NEG | NEG | NEG | NEG | NEG | NEG | NEG | NEG | NEG | NEG | NEG | NEG | NEG | NEG | POS | NEG | NEG | NEG | NEG | NEG | NEG | NEG | NEG | NEG | NEG | NEG | NEG | NEG | NEG | NEG |
| Dresden 07_8029 | NEG | NEG | NEG | NEG | NEG | NEG | NEG | NEG | NEG | NEG | NEG | NEG | NEG | NEG | NEG | NEG | NEG | NEG | POS | NEG | NEG | NEG | NEG | NEG | NEG | NEG | NEG | NEG | NEG | NEG | NEG | NEG | NEG | NEG |
| Dresden 07_8575 | NEG | NEG | NEG | NEG | NEG | NEG | NEG | NEG | NEG | NEG | NEG | NEG | NEG | NEG | NEG | NEG | NEG | NEG | POS | NEG | NEG | NEG | NEG | NEG | NEG | NEG | NEG | NEG | NEG | NEG | NEG | NEG | NEG | NEG |
| Dresden 08_1746 | NEG | NEG | NEG | NEG | NEG | NEG | NEG | NEG | NEG | NEG | NEG | NEG | NEG | NEG | NEG | NEG | NEG | NEG | POS | NEG | NEG | NEG | NEG | NEG | NEG | NEG | NEG | NEG | NEG | NEG | NEG | NEG | NEG | NEG |
| >HP-19 | NEG | NEG | NEG | NEG | NEG | NEG | NEG | NEG | NEG | NEG | NEG | NEG | NEG | NEG | NEG | NEG | NEG | NEG | NEG | NEG | NEG | NEG | NEG | NEG | NEG | NEG | NEG | NEG | NEG | NEG | NEG | NEG | NEG | NEG |
| Freiburg-894-37.894-DBK0000289 | NEG | NEG | NEG | NEG | NEG | NEG | NEG | NEG | NEG | NEG | NEG | NEG | NEG | NEG | NEG | NEG | NEG | NEG | NEG | NEG | NEG | NEG | NEG | NEG | NEG | NEG | NEG | NEG | NEG | NEG | NEG | NEG | NEG | NEG |
| | NEG | NEG | NEG | NEG | NEG | NEG | NEG | NEG | NEG | NEG | NEG | NEG | NEG | NEG | NEG | NEG | NEG | NEG | NEG | NEG | NEG | NEG | NEG | NEG | NEG | NEG | NEG | NEG | NEG | NEG | NEG | NEG | NEG | NEG |
| Freiburg-1000-67.1-DBK0000263 | NEG | NEG | NEG | NEG | NEG | NEG | NEG | NEG | NEG | NEG | NEG | NEG | NEG | NEG | NEG | NEG | NEG | NEG | NEG | NEG | NEG | NEG | NEG | NEG | NEG | NEG | NEG | NEG | NEG | NEG | NEG | NEG | NEG | NEG |
| | NEG | NEG | NEG | NEG | NEG | NEG | NEG | NEG | NEG | NEG | NEG | NEG | NEG | NEG | NEG | NEG | NEG | NEG | NEG | NEG | NEG | NEG | NEG | NEG | NEG | NEG | NEG | NEG | NEG | NEG | NEG | NEG | NEG | NEG |
| Dresden 07_7828 | NEG | NEG | NEG | NEG | NEG | NEG | NEG | NEG | NEG | NEG | NEG | NEG | NEG | NEG | NEG | NEG | NEG | NEG | NEG | NEG | NEG | NEG | NEG | NEG | NEG | NEG | NEG | NEG | NEG | NEG | NEG | NEG | NEG | NEG |
| Dresden 08_1128 | NEG | NEG | NEG | NEG | NEG | NEG | NEG | NEG | NEG | NEG | NEG | NEG | NEG | NEG | NEG | NEG | NEG | NEG | NEG | NEG | NEG | NEG | NEG | NEG | NEG | NEG | NEG | NEG | NEG | NEG | NEG | NEG | NEG | NEG |

| STRAIN / ISOLATE | splA Surface layer protein A, cont. | | | | | | | | | | | | | | | | | | | | | | | | | | | | | | | | | |
|---|---|---|---|---|---|---|---|---|---|---|---|---|---|---|---|---|---|---|---|---|---|---|---|---|---|---|---|---|---|---|---|---|---|---|
| | hp1180\|slpA | hp1182\|slpA | hp1183\|slpA | hp1184\|slpA | hp1186\|slpA | hp1188\|slpA | hp1190\|slpA | hp1191\|slpA | hp1192\|slpA | hp1193\|slpA | hp1195\|slpA | hp1197\|slpA | hp1198\|slpA | hp1199\|slpA | hp1200\|slpA | hp1201\|slpA | hp1202\|slpA | hp1203\|slpA | hp1204\|slpA | hp1205\|slpA | hp1206\|slpA | hp1208\|slpA | hp1209\|slpA | hp1210\|slpA | hp1211\|slpA | hp1232\|slpA | hp1233\|slpA | hp1234\|slpA | hp1236\|slpA | hp1237\|slpA | hp1239\|slpA | hp1242\|slpA | hp1243\|slpA | hp1249\|slpA |
| >HP-20 | NEG | NEG | NEG | NEG | NEG | NEG | NEG | NEG | NEG | NEG | NEG | NEG | NEG | NEG | NEG | NEG | NEG | POS | NEG | NEG | NEG | NEG | NEG | NEG | NEG | NEG | NEG | NEG | NEG | NEG | NEG | NEG | NEG | NEG |
| Freiburg-963-50.963-DBK0000243 | NEG | NEG | NEG | NEG | NEG | NEG | NEG | NEG | NEG | NEG | NEG | NEG | NEG | NEG | NEG | NEG | NEG | POS | NEG | NEG | NEG | NEG | NEG | NEG | NEG | NEG | NEG | NEG | NEG | NEG | NEG | NEG | NEG | NEG |
| Freiburg-750-18.75-DBK0000232 | NEG | NEG | NEG | NEG | NEG | NEG | NEG | NEG | NEG | NEG | NEG | NEG | NEG | NEG | NEG | NEG | NEG | POS | NEG | NEG | NEG | NEG | NEG | NEG | NEG | NEG | NEG | NEG | NEG | NEG | NEG | NEG | NEG | NEG |
| | NEG | NEG | NEG | NEG | NEG | NEG | NEG | NEG | NEG | NEG | NEG | NEG | NEG | NEG | NEG | NEG | NEG | NEG | NEG | NEG | NEG | NEG | NEG | NEG | NEG | NEG | NEG | NEG | NEG | NEG | NEG | NEG | NEG | NEG |
| Freiburg-637-4.637-DBK0000280 | NEG | NEG | NEG | NEG | NEG | NEG | NEG | NEG | NEG | NEG | NEG | NEG | NEG | NEG | NEG | NEG | NEG | POS | NEG | NEG | NEG | NEG | NEG | NEG | NEG | NEG | NEG | NEG | NEG | NEG | NEG | NEG | NEG | NEG |
| | NEG | NEG | NEG | NEG | NEG | NEG | NEG | NEG | NEG | NEG | NEG | NEG | NEG | NEG | NEG | NEG | NEG | POS | NEG | NEG | NEG | NEG | NEG | NEG | NEG | NEG | NEG | NEG | NEG | NEG | NEG | NEG | NEG | NEG |
| Freiburg-856-31.856-DBK0000273 | NEG | NEG | NEG | NEG | NEG | NEG | NEG | NEG | NEG | NEG | NEG | NEG | NEG | NEG | NEG | NEG | NEG | POS | NEG | NEG | NEG | NEG | NEG | NEG | NEG | NEG | NEG | NEG | NEG | NEG | NEG | NEG | NEG | NEG |
| | NEG | AMB | NEG | NEG | NEG | NEG | NEG | NEG | NEG | NEG | NEG | NEG | NEG | NEG | NEG | NEG | NEG | POS | NEG | NEG | NEG | NEG | NEG | NEG | NEG | NEG | NEG | NEG | NEG | NEG | NEG | NEG | NEG | NEG |
| Freiburg_51.956 | NEG | NEG | NEG | NEG | NEG | NEG | NEG | NEG | NEG | NEG | NEG | NEG | NEG | NEG | NEG | NEG | NEG | POS | NEG | NEG | NEG | NEG | NEG | NEG | NEG | NEG | NEG | NEG | NEG | NEG | NEG | NEG | NEG | NEG |
| Dresden 07_7984 | NEG | NEG | NEG | NEG | NEG | NEG | NEG | NEG | NEG | NEG | NEG | NEG | NEG | NEG | NEG | NEG | NEG | AMB | NEG | NEG | NEG | NEG | NEG | NEG | NEG | NEG | NEG | NEG | NEG | NEG | NEG | NEG | NEG | NEG |
| Dresden 07_8203 | NEG | NEG | NEG | NEG | NEG | NEG | NEG | NEG | NEG | NEG | NEG | NEG | NEG | NEG | NEG | NEG | NEG | AMB | NEG | NEG | NEG | NEG | NEG | NEG | NEG | NEG | NEG | NEG | NEG | NEG | NEG | NEG | NEG | NEG |
| Dresden 07_8398 | NEG | NEG | NEG | NEG | NEG | NEG | NEG | NEG | NEG | NEG | NEG | NEG | NEG | NEG | NEG | NEG | NEG | AMB | NEG | NEG | NEG | NEG | NEG | NEG | NEG | NEG | NEG | NEG | NEG | NEG | NEG | NEG | NEG | NEG |
| Dresden 07_8798 | NEG | NEG | NEG | NEG | NEG | NEG | NEG | NEG | NEG | NEG | NEG | NEG | NEG | NEG | NEG | NEG | NEG | AMB | NEG | NEG | NEG | NEG | NEG | NEG | NEG | NEG | NEG | NEG | NEG | NEG | NEG | NEG | NEG | NEG |
| >HP-21 | NEG | NEG | NEG | NEG | NEG | NEG | NEG | NEG | NEG | NEG | NEG | NEG | NEG | NEG | NEG | NEG | NEG | NEG | NEG | NEG | NEG | NEG | NEG | NEG | NEG | POS | NEG | NEG | NEG | NEG | POS | NEG | NEG | NEG |
| Freiburg-823-26.823-DBK0000283 | NEG | NEG | NEG | NEG | NEG | NEG | NEG | NEG | NEG | NEG | NEG | NEG | NEG | NEG | NEG | NEG | NEG | NEG | NEG | NEG | NEG | NEG | NEG | NEG | NEG | POS | NEG | NEG | NEG | NEG | POS | NEG | NEG | NEG |
| | NEG | NEG | NEG | NEG | NEG | NEG | NEG | NEG | NEG | NEG | NEG | NEG | NEG | NEG | NEG | NEG | NEG | NEG | NEG | NEG | NEG | NEG | NEG | NEG | NEG | POS | NEG | NEG | NEG | NEG | POS | NEG | NEG | NEG |
| Freiburg-952-49.952-DBK0000245 | NEG | NEG | NEG | NEG | NEG | NEG | NEG | NEG | NEG | NEG | NEG | NEG | NEG | NEG | NEG | NEG | NEG | NEG | NEG | NEG | NEG | NEG | NEG | NEG | NEG | POS | NEG | NEG | NEG | NEG | POS | NEG | NEG | NEG |
| | NEG | NEG | NEG | NEG | NEG | NEG | NEG | NEG | NEG | NEG | NEG | NEG | NEG | NEG | NEG | NEG | NEG | NEG | NEG | NEG | NEG | NEG | NEG | NEG | NEG | POS | NEG | NEG | NEG | NEG | POS | NEG | NEG | NEG |
| >HP-22 | NEG | NEG | NEG | NEG | NEG | NEG | NEG | NEG | NEG | NEG | NEG | NEG | NEG | NEG | NEG | NEG | NEG | NEG | NEG | NEG | NEG | NEG | NEG | NEG | NEG | POS | NEG | NEG | NEG | NEG | POS | NEG | NEG | NEG |
| Dresden 08_0288 | NEG | NEG | NEG | NEG | NEG | NEG | NEG | NEG | NEG | NEG | NEG | NEG | NEG | NEG | NEG | NEG | NEG | NEG | NEG | NEG | NEG | NEG | NEG | NEG | NEG | POS | NEG | NEG | NEG | NEG | POS | NEG | NEG | NEG |
| >HP-23 | NEG | NEG | NEG | NEG | NEG | NEG | NEG | NEG | NEG | NEG | NEG | NEG | NEG | NEG | NEG | NEG | NEG | NEG | NEG | NEG | NEG | NEG | NEG | NEG | NEG | POS | NEG | NEG | NEG | NEG | POS | NEG | NEG | NEG |
| Freiburg-786-21.786-DBK0000275 | NEG | NEG | NEG | NEG | NEG | NEG | NEG | NEG | NEG | NEG | NEG | NEG | NEG | NEG | NEG | NEG | NEG | NEG | NEG | NEG | NEG | NEG | NEG | NEG | NEG | POS | NEG | NEG | NEG | NEG | POS | NEG | NEG | NEG |
| | NEG | NEG | NEG | NEG | NEG | NEG | NEG | NEG | NEG | NEG | NEG | NEG | NEG | NEG | NEG | NEG | NEG | NEG | NEG | NEG | NEG | NEG | NEG | NEG | NEG | POS | NEG | NEG | NEG | NEG | POS | NEG | NEG | NEG |
| >HP-24 | NEG | NEG | NEG | NEG | NEG | NEG | NEG | NEG | NEG | NEG | NEG | NEG | POS | NEG | NEG | NEG | NEG | NEG | NEG | NEG | NEG | NEG | NEG | NEG | NEG | NEG | NEG | NEG | NEG | NEG | NEG | NEG | NEG | NEG |
| C. difficile 6534 (GenBank ADEJ) in silico prediction | NEG | NEG | NEG | NEG | NEG | NEG | NEG | NEG | NEG | NEG | NEG | NEG | POS | NEG | NEG | NEG | NEG | NEG | NEG | NEG | NEG | NEG | NEG | NEG | NEG | NEG | NEG | NEG | NEG | NEG | NEG | NEG | NEG | NEG |
| C. difficile 630 (GenBank AM180355) in silico prediction | NEG | NEG | NEG | NEG | NEG | NEG | NEG | NEG | NEG | NEG | NEG | NEG | POS | NEG | NEG | NEG | NEG | NEG | NEG | NEG | NEG | NEG | NEG | NEG | NEG | NEG | NEG | NEG | NEG | NEG | NEG | NEG | NEG | NEG |
| C. difficile Strain 630 | NEG | NEG | NEG | NEG | NEG | NEG | NEG | NEG | NEG | NEG | NEG | NEG | AMB | NEG | NEG | NEG | NEG | NEG | NEG | NEG | NEG | NEG | NEG | NEG | NEG | NEG | NEG | NEG | NEG | NEG | NEG | NEG | NEG | NEG |
| | NEG | NEG | NEG | NEG | NEG | NEG | NEG | NEG | NEG | NEG | NEG | AMB | AMB | NEG | NEG | NEG | NEG | NEG | NEG | NEG | NEG | NEG | NEG | NEG | NEG | NEG | NEG | NEG | NEG | NEG | NEG | NEG | NEG | NEG |
| | NEG | NEG | NEG | NEG | NEG | NEG | NEG | NEG | NEG | NEG | NEG | NEG | POS | NEG | NEG | NEG | NEG | NEG | NEG | NEG | NEG | NEG | NEG | NEG | NEG | NEG | NEG | NEG | NEG | NEG | NEG | NEG | NEG | NEG |
| | NEG | NEG | NEG | NEG | NEG | NEG | NEG | NEG | NEG | NEG | NEG | NEG | AMB | NEG | NEG | NEG | NEG | NEG | NEG | NEG | NEG | NEG | NEG | NEG | NEG | NEG | NEG | NEG | NEG | NEG | NEG | NEG | NEG | NEG |
| | NEG | NEG | NEG | NEG | NEG | NEG | NEG | NEG | NEG | NEG | NEG | NEG | POS | NEG | NEG | NEG | NEG | NEG | NEG | NEG | NEG | NEG | NEG | NEG | NEG | NEG | NEG | NEG | NEG | NEG | NEG | NEG | NEG | NEG |
| | NEG | NEG | NEG | NEG | NEG | NEG | NEG | NEG | NEG | NEG | NEG | NEG | NEG | NEG | NEG | NEG | NEG | NEG | NEG | NEG | NEG | NEG | NEG | NEG | NEG | NEG | NEG | NEG | NEG | NEG | NEG | NEG | NEG | NEG |
| Dresden 07_7156 | NEG | NEG | NEG | NEG | NEG | NEG | NEG | NEG | NEG | NEG | NEG | NEG | NEG | NEG | NEG | NEG | NEG | NEG | NEG | NEG | NEG | NEG | NEG | NEG | NEG | NEG | NEG | NEG | NEG | NEG | NEG | NEG | NEG | NEG |
| Dresden 07_7158 | NEG | NEG | NEG | NEG | NEG | NEG | NEG | NEG | NEG | NEG | NEG | NEG | POS | NEG | NEG | NEG | NEG | NEG | NEG | NEG | NEG | NEG | NEG | NEG | NEG | NEG | NEG | NEG | NEG | NEG | NEG | NEG | NEG | NEG |
| Dresden 07_7203 | NEG | NEG | NEG | NEG | NEG | NEG | NEG | NEG | NEG | NEG | NEG | NEG | POS | NEG | NEG | NEG | NEG | NEG | NEG | NEG | NEG | NEG | NEG | NEG | NEG | NEG | NEG | NEG | NEG | NEG | NEG | NEG | NEG | NEG |
| Dresden 07_8217 | NEG | NEG | NEG | NEG | NEG | NEG | NEG | NEG | NEG | NEG | NEG | NEG | POS | NEG | NEG | NEG | NEG | NEG | NEG | NEG | NEG | NEG | NEG | NEG | NEG | NEG | NEG | NEG | NEG | NEG | NEG | NEG | NEG | NEG |
| Dresden 07_8270 | NEG | NEG | NEG | NEG | NEG | NEG | NEG | NEG | NEG | NEG | NEG | NEG | POS | NEG | NEG | NEG | NEG | NEG | NEG | NEG | NEG | NEG | NEG | NEG | NEG | NEG | NEG | NEG | NEG | NEG | NEG | NEG | NEG | NEG |
| Dresden 07_8749 | NEG | NEG | NEG | NEG | NEG | NEG | NEG | NEG | NEG | NEG | NEG | NEG | NEG | NEG | NEG | NEG | NEG | NEG | NEG | NEG | NEG | NEG | NEG | NEG | NEG | NEG | NEG | NEG | NEG | NEG | NEG | NEG | NEG | NEG |
| Dresden 07_8788 | NEG | NEG | NEG | NEG | NEG | NEG | NEG | NEG | NEG | NEG | NEG | NEG | POS | NEG | NEG | NEG | NEG | NEG | NEG | NEG | NEG | NEG | NEG | NEG | NEG | NEG | NEG | NEG | NEG | NEG | NEG | NEG | NEG | NEG |
| Dresden 07_9245 | NEG | NEG | NEG | NEG | NEG | NEG | NEG | NEG | NEG | NEG | NEG | NEG | POS | NEG | NEG | NEG | NEG | NEG | NEG | NEG | NEG | NEG | NEG | NEG | NEG | NEG | NEG | NEG | NEG | NEG | NEG | NEG | NEG | NEG |
| Dresden 08_0217 | NEG | NEG | NEG | NEG | NEG | NEG | NEG | NEG | NEG | NEG | NEG | NEG | POS | NEG | NEG | NEG | NEG | NEG | NEG | NEG | NEG | NEG | NEG | NEG | NEG | NEG | NEG | NEG | NEG | NEG | NEG | NEG | NEG | NEG |
| Dresden 08_0219 | NEG | NEG | NEG | NEG | NEG | NEG | NEG | NEG | NEG | NEG | NEG | NEG | POS | NEG | NEG | NEG | NEG | NEG | NEG | NEG | NEG | NEG | NEG | NEG | NEG | NEG | NEG | NEG | NEG | NEG | NEG | NEG | NEG | NEG |
| Dresden 08_0419 | NEG | NEG | NEG | NEG | NEG | NEG | NEG | NEG | NEG | NEG | NEG | NEG | POS | NEG | NEG | NEG | NEG | NEG | NEG | NEG | NEG | NEG | NEG | NEG | NEG | NEG | NEG | NEG | NEG | NEG | NEG | NEG | NEG | NEG |
| Dresden 08_0484 | NEG | NEG | NEG | NEG | NEG | NEG | NEG | NEG | NEG | NEG | NEG | NEG | POS | NEG | NEG | NEG | NEG | NEG | NEG | NEG | NEG | NEG | NEG | NEG | NEG | NEG | NEG | NEG | NEG | NEG | NEG | NEG | NEG | NEG |
| Dresden 08_0994 | NEG | NEG | NEG | NEG | NEG | NEG | NEG | NEG | NEG | NEG | NEG | NEG | POS | NEG | NEG | NEG | NEG | NEG | NEG | NEG | NEG | NEG | NEG | NEG | NEG | NEG | NEG | NEG | NEG | NEG | NEG | NEG | NEG | NEG |
| Dresden 08_1148 | NEG | NEG | NEG | NEG | NEG | NEG | NEG | NEG | NEG | NEG | NEG | NEG | AMB | NEG | NEG | NEG | NEG | NEG | NEG | NEG | NEG | NEG | NEG | NEG | NEG | NEG | NEG | NEG | NEG | NEG | NEG | NEG | NEG | NEG |
| Dresden 08_1188 | NEG | NEG | NEG | NEG | NEG | NEG | NEG | NEG | NEG | NEG | NEG | NEG | POS | NEG | NEG | NEG | NEG | NEG | NEG | NEG | NEG | NEG | NEG | NEG | NEG | NEG | NEG | NEG | NEG | NEG | NEG | NEG | NEG | NEG |
| Dresden 08_1205 | NEG | NEG | NEG | NEG | NEG | NEG | NEG | NEG | NEG | NEG | NEG | NEG | NEG | NEG | NEG | NEG | NEG | NEG | NEG | NEG | NEG | NEG | NEG | NEG | NEG | NEG | NEG | NEG | NEG | NEG | NEG | NEG | NEG | NEG |
| Dresden 08_1541 | NEG | NEG | NEG | NEG | NEG | NEG | AMB | NEG | NEG | NEG | NEG | NEG | POS | NEG | NEG | NEG | NEG | NEG | NEG | NEG | NEG | NEG | NEG | NEG | NEG | NEG | NEG | NEG | NEG | NEG | NEG | NEG | NEG | NEG |
| Dresden 06_7896 | NEG | NEG | NEG | NEG | NEG | NEG | NEG | NEG | NEG | NEG | NEG | NEG | AMB | NEG | NEG | NEG | NEG | NEG | NEG | NEG | NEG | NEG | NEG | NEG | NEG | NEG | NEG | NEG | NEG | NEG | NEG | NEG | NEG | NEG |
| >HP-25 | NEG | NEG | NEG | NEG | NEG | NEG | NEG | NEG | NEG | NEG | NEG | NEG | NEG | NEG | NEG | NEG | NEG | NEG | NEG | NEG | NEG | NEG | NEG | NEG | NEG | NEG | NEG | NEG | NEG | NEG | NEG | NEG | POS | NEG |
| Freiburg-809-24.809-DBK0000287 | NEG | NEG | NEG | NEG | NEG | NEG | NEG | NEG | NEG | NEG | NEG | NEG | NEG | NEG | NEG | NEG | NEG | NEG | NEG | NEG | NEG | NEG | NEG | NEG | NEG | NEG | NEG | NEG | NEG | NEG | NEG | NEG | POS | NEG |
| | NEG | NEG | NEG | NEG | NEG | NEG | NEG | NEG | NEG | NEG | NEG | NEG | NEG | NEG | NEG | NEG | NEG | NEG | NEG | NEG | NEG | NEG | NEG | NEG | NEG | NEG | NEG | NEG | NEG | NEG | NEG | NEG | POS | NEG |
| Freiburg-990-64.99-DBK0000264 | NEG | NEG | NEG | NEG | NEG | NEG | NEG | NEG | NEG | NEG | NEG | NEG | NEG | NEG | NEG | NEG | NEG | NEG | NEG | NEG | NEG | NEG | NEG | NEG | NEG | NEG | NEG | NEG | NEG | NEG | NEG | NEG | POS | NEG |
| | NEG | NEG | NEG | NEG | NEG | NEG | NEG | NEG | NEG | NEG | NEG | NEG | NEG | NEG | NEG | NEG | NEG | NEG | NEG | NEG | NEG | NEG | NEG | NEG | NEG | NEG | NEG | NEG | NEG | NEG | NEG | NEG | POS | NEG |
| Dresden 07_7952 | NEG | NEG | NEG | NEG | NEG | NEG | NEG | NEG | NEG | NEG | NEG | NEG | NEG | NEG | NEG | NEG | NEG | NEG | NEG | NEG | NEG | NEG | NEG | NEG | NEG | NEG | NEG | NEG | NEG | NEG | NEG | NEG | POS | NEG |
| Dresden 07_8955 | NEG | NEG | NEG | NEG | NEG | NEG | NEG | NEG | NEG | NEG | NEG | NEG | NEG | NEG | NEG | NEG | NEG | NEG | NEG | NEG | NEG | NEG | NEG | NEG | NEG | NEG | NEG | NEG | NEG | NEG | NEG | NEG | POS | NEG |
| Dresden 07_8958 | NEG | NEG | NEG | NEG | NEG | NEG | NEG | NEG | NEG | NEG | NEG | NEG | NEG | NEG | NEG | NEG | NEG | NEG | NEG | NEG | NEG | NEG | NEG | NEG | NEG | NEG | NEG | NEG | NEG | NEG | NEG | NEG | POS | NEG |
| Dresden 07_9027 | NEG | NEG | NEG | NEG | NEG | NEG | NEG | NEG | NEG | NEG | NEG | NEG | NEG | NEG | NEG | NEG | NEG | NEG | NEG | NEG | NEG | NEG | NEG | NEG | NEG | NEG | NEG | NEG | NEG | NEG | NEG | NEG | POS | NEG |
| Dresden 08_1169 | NEG | NEG | NEG | NEG | NEG | NEG | NEG | NEG | NEG | NEG | NEG | NEG | NEG | NEG | NEG | NEG | NEG | NEG | NEG | NEG | NEG | NEG | NEG | NEG | NEG | NEG | NEG | NEG | NEG | NEG | NEG | NEG | NEG | NEG |
| >HP-26 | NEG | NEG | NEG | NEG | POS | NEG | NEG | NEG | NEG | NEG | NEG | NEG | NEG | NEG | NEG | POS | NEG | NEG | NEG | NEG | NEG | NEG | NEG | NEG | NEG | NEG | NEG | NEG | NEG | NEG | NEG | NEG | NEG | NEG |
| Freiburg-635-3.635-DBK0000297 | NEG | NEG | NEG | NEG | POS | NEG | NEG | NEG | NEG | NEG | NEG | NEG | NEG | NEG | NEG | POS | NEG | NEG | NEG | NEG | NEG | NEG | NEG | NEG | NEG | NEG | NEG | NEG | NEG | NEG | NEG | NEG | NEG | NEG |
| | NEG | NEG | NEG | NEG | POS | NEG | NEG | NEG | NEG | NEG | NEG | NEG | NEG | NEG | NEG | POS | NEG | NEG | NEG | NEG | NEG | NEG | NEG | NEG | NEG | NEG | NEG | NEG | NEG | NEG | NEG | NEG | NEG | NEG |
| Freiburg-895-38.895-DBK0000290 | NEG | NEG | NEG | NEG | POS | NEG | NEG | NEG | NEG | NEG | NEG | NEG | NEG | NEG | NEG | POS | NEG | NEG | NEG | NEG | NEG | NEG | NEG | NEG | NEG | NEG | NEG | NEG | NEG | NEG | NEG | NEG | NEG | NEG |
| | NEG | NEG | NEG | NEG | POS | NEG | NEG | NEG | NEG | NEG | NEG | NEG | NEG | NEG | NEG | POS | NEG | NEG | NEG | NEG | NEG | NEG | NEG | NEG | NEG | NEG | NEG | NEG | NEG | NEG | NEG | NEG | NEG | NEG |
| >HP-27 | NEG | NEG | NEG | NEG | NEG | NEG | NEG | NEG | POS | NEG | NEG | NEG | NEG | NEG | NEG | NEG | NEG | NEG | NEG | NEG | NEG | NEG | NEG | NEG | NEG | NEG | NEG | NEG | NEG | NEG | NEG | NEG | NEG | NEG |
| Freiburg-726-14.726-DBK0000228 | NEG | NEG | NEG | NEG | NEG | NEG | NEG | NEG | POS | NEG | NEG | NEG | NEG | NEG | NEG | NEG | NEG | NEG | NEG | NEG | NEG | NEG | NEG | NEG | NEG | NEG | NEG | NEG | NEG | NEG | NEG | NEG | NEG | NEG |
| | NEG | NEG | NEG | NEG | NEG | NEG | NEG | NEG | POS | NEG | NEG | NEG | NEG | NEG | NEG | NEG | NEG | NEG | NEG | NEG | NEG | NEG | NEG | NEG | NEG | NEG | NEG | NEG | NEG | NEG | NEG | NEG | NEG | NEG |
| Freiburg-837-27.837-DBK0000285 | NEG | NEG | NEG | NEG | NEG | NEG | NEG | NEG | POS | NEG | NEG | NEG | NEG | NEG | NEG | NEG | NEG | NEG | NEG | NEG | NEG | NEG | NEG | NEG | NEG | NEG | NEG | NEG | NEG | NEG | NEG | NEG | NEG | NEG |
| | NEG | NEG | NEG | NEG | NEG | NEG | NEG | NEG | POS | NEG | NEG | NEG | NEG | NEG | NEG | NEG | NEG | NEG | NEG | NEG | NEG | NEG | NEG | NEG | NEG | NEG | NEG | NEG | NEG | NEG | NEG | NEG | NEG | NEG |
| >HP-28 | NEG | NEG | NEG | NEG | NEG | NEG | NEG | NEG | NEG | NEG | NEG | NEG | NEG | NEG | NEG | NEG | NEG | NEG | NEG | NEG | NEG | NEG | NEG | NEG | NEG | NEG | NEG | NEG | NEG | NEG | NEG | NEG | NEG | NEG |
| C. difficile 6407 (GenBank ADEH) in silico prediction | NEG | NEG | NEG | NEG | NEG | NEG | NEG | NEG | NEG | NEG | NEG | NEG | NEG | NEG | NEG | NEG | NEG | NEG | NEG | NEG | NEG | NEG | NEG | NEG | NEG | NEG | NEG | NEG | NEG | NEG | NEG | NEG | NEG | NEG |
| >HP-29 | NEG | NEG | NEG | NEG | NEG | NEG | NEG | NEG | NEG | NEG | NEG | NEG | NEG | NEG | NEG | NEG | NEG | NEG | NEG | NEG | NEG | NEG | NEG | NEG | NEG | NEG | NEG | NEG | NEG | NEG | NEG | NEG | NEG | NEG |
| Freiburg-852-29.852-DBK0000279 | NEG | NEG | NEG | NEG | NEG | NEG | NEG | NEG | NEG | NEG | NEG | NEG | NEG | NEG | NEG | NEG | NEG | NEG | NEG | NEG | NEG | NEG | NEG | NEG | NEG | NEG | NEG | NEG | NEG | NEG | NEG | NEG | NEG | NEG |
| | NEG | NEG | NEG | NEG | NEG | NEG | NEG | NEG | NEG | NEG | NEG | NEG | NEG | NEG | NEG | NEG | NEG | NEG | NEG | NEG | NEG | NEG | NEG | NEG | NEG | NEG | NEG | NEG | NEG | NEG | NEG | NEG | NEG | NEG |
| Freiburg-858-32.858-DBK0000222 | NEG | NEG | NEG | NEG | NEG | NEG | NEG | NEG | NEG | NEG | NEG | NEG | NEG | NEG | NEG | NEG | NEG | NEG | NEG | NEG | NEG | NEG | NEG | NEG | NEG | NEG | NEG | NEG | NEG | NEG | NEG | NEG | NEG | NEG |
| >HP-30 | NEG | NEG | NEG | NEG | NEG | NEG | NEG | NEG | NEG | NEG | NEG | NEG | NEG | NEG | NEG | NEG | NEG | NEG | NEG | NEG | NEG | NEG | NEG | NEG | NEG | NEG | NEG | NEG | NEG | NEG | NEG | NEG | NEG | NEG |
| Freiburg-1047-76.1047-DBK0000269 | NEG | NEG | NEG | NEG | NEG | NEG | NEG | NEG | NEG | NEG | NEG | NEG | NEG | NEG | NEG | NEG | NEG | NEG | NEG | NEG | NEG | NEG | NEG | NEG | NEG | NEG | NEG | NEG | NEG | NEG | NEG | NEG | NEG | NEG |
| | NEG | NEG | NEG | NEG | NEG | NEG | NEG | NEG | NEG | NEG | NEG | NEG | NEG | NEG | NEG | NEG | NEG | NEG | NEG | NEG | NEG | NEG | NEG | NEG | NEG | NEG | NEG | NEG | NEG | NEG | NEG | NEG | NEG | NEG |
| Freiburg-999-66.999-DBK0000262 | NEG | NEG | NEG | NEG | NEG | NEG | NEG | NEG | NEG | NEG | NEG | NEG | NEG | NEG | NEG | NEG | NEG | NEG | NEG | NEG | NEG | NEG | NEG | NEG | NEG | NEG | NEG | NEG | NEG | NEG | NEG | NEG | NEG | NEG |
| | NEG | NEG | NEG | NEG | NEG | NEG | NEG | NEG | NEG | NEG | NEG | NEG | NEG | NEG | NEG | NEG | NEG | NEG | NEG | NEG | NEG | NEG | NEG | NEG | NEG | NEG | NEG | NEG | NEG | NEG | NEG | NEG | NEG | NEG |
| Freiburg-896-39.896-DBK0000291 | NEG | NEG | NEG | NEG | NEG | NEG | NEG | NEG | NEG | NEG | NEG | NEG | NEG | NEG | NEG | NEG | NEG | NEG | NEG | NEG | NEG | NEG | NEG | NEG | NEG | NEG | NEG | NEG | NEG | NEG | NEG | NEG | NEG | NEG |
| | NEG | NEG | NEG | NEG | NEG | NEG | NEG | NEG | NEG | NEG | NEG | NEG | NEG | NEG | NEG | NEG | NEG | NEG | NEG | NEG | NEG | NEG | NEG | NEG | NEG | NEG | NEG | NEG | NEG | NEG | NEG | NEG | NEG | NEG |
| Freiburg-871-33.871-DBK0000272 | NEG | NEG | NEG | NEG | NEG | NEG | NEG | NEG | NEG | NEG | NEG | NEG | NEG | NEG | NEG | NEG | NEG | NEG | NEG | NEG | NEG | NEG | NEG | NEG | NEG | NEG | NEG | NEG | NEG | NEG | NEG | NEG | NEG | NEG |
| | NEG | NEG | NEG | NEG | NEG | NEG | NEG | NEG | NEG | NEG | NEG | NEG | NEG | NEG | NEG | NEG | NEG | NEG | NEG | NEG | NEG | NEG | NEG | NEG | NEG | NEG | NEG | NEG | NEG | NEG | NEG | NEG | NEG | NEG |
| Dresden 08_1596 | NEG | NEG | NEG | NEG | NEG | NEG | NEG | NEG | NEG | NEG | NEG | NEG | NEG | NEG | NEG | NEG | NEG | NEG | NEG | NEG | NEG | NEG | NEG | NEG | NEG | NEG | NEG | NEG | NEG | NEG | NEG | NEG | NEG | NEG |

| STRAIN / ISOLATE | splA Surface layer protein A, cont. | | | | | | | | | | | | | | | | | | | | | | | | | | | | | | | | | |
|---|---|---|---|---|---|---|---|---|---|---|---|---|---|---|---|---|---|---|---|---|---|---|---|---|---|---|---|---|---|---|---|---|---|---|
| | hp1180\|slpA | hp1182\|slpA | hp1183\|slpA | hp1184\|slpA | hp1186\|slpA | hp1188\|slpA | hp1190\|slpA | hp1191\|slpA | hp1192\|slpA | hp1193\|slpA | hp1195\|slpA | hp1197\|slpA | hp1198\|slpA | hp1199\|slpA | hp1200\|slpA | hp1201\|slpA | hp1202\|slpA | hp1203\|slpA | hp1204\|slpA | hp1205\|slpA | hp1206\|slpA | hp1208\|slpA | hp1209\|slpA | hp1210\|slpA | hp1211\|slpA | hp1232\|slpA | hp1233\|slpA | hp1234\|slpA | hp1236\|slpA | hp1237\|slpA | hp1239\|slpA | hp1242\|slpA | hp1243\|slpA | hp1249\|slpA |
| CLADE 2 (CC1 /Ribo 27 Complex) | | | | | | | | | | | | | | | | | | | | | | | | | | | | | | | | | | |
| >HP-31 | NEG | NEG | NEG | NEG | NEG | NEG | NEG | NEG | NEG | NEG | NEG | NEG | NEG | NEG | NEG | NEG | NEG | NEG | NEG | NEG | NEG | NEG | NEG | NEG | NEG | NEG | NEG | NEG | NEG | NEG | NEG | NEG | NEG | NEG |
| C. difficile BI1 (GenBank FN668941) in silico prediction | NEG | NEG | NEG | NEG | NEG | NEG | NEG | NEG | NEG | NEG | NEG | NEG | NEG | NEG | NEG | NEG | NEG | NEG | NEG | NEG | NEG | NEG | NEG | NEG | NEG | NEG | NEG | NEG | NEG | NEG | NEG | NEG | NEG | NEG |
| C. difficile QCD-97b34 (GenBank ABHF) in silico prediction | NEG | NEG | NEG | NEG | NEG | NEG | NEG | NEG | NEG | NEG | NEG | NEG | NEG | NEG | NEG | NEG | NEG | NEG | NEG | NEG | NEG | NEG | NEG | NEG | NEG | NEG | NEG | NEG | NEG | NEG | NEG | NEG | NEG | NEG |
| C. difficile QCD-76w55 (GenBank ABHE) in silico prediction | NEG | NEG | NEG | NEG | NEG | NEG | NEG | NEG | NEG | NEG | NEG | NEG | NEG | NEG | NEG | NEG | NEG | NEG | NEG | NEG | NEG | NEG | NEG | NEG | NEG | NEG | NEG | NEG | NEG | NEG | NEG | NEG | NEG | NEG |
| C. difficile CIP107932 (GenBank ABKK) in silico prediction | NEG | NEG | NEG | NEG | NEG | NEG | NEG | NEG | NEG | NEG | NEG | NEG | NEG | NEG | NEG | NEG | NEG | NEG | NEG | NEG | NEG | NEG | NEG | NEG | NEG | NEG | NEG | NEG | NEG | NEG | NEG | NEG | NEG | NEG |
| C. difficile CD196 (GenBank FN538970) in silico prediction | NEG | NEG | NEG | NEG | NEG | NEG | NEG | NEG | NEG | NEG | NEG | NEG | NEG | NEG | NEG | NEG | NEG | NEG | NEG | NEG | NEG | NEG | NEG | NEG | NEG | NEG | NEG | NEG | NEG | NEG | NEG | NEG | NEG | NEG |
| C. difficile Strain CD196 | NEG | NEG | NEG | NEG | NEG | NEG | NEG | NEG | NEG | NEG | NEG | NEG | NEG | NEG | NEG | NEG | NEG | NEG | NEG | NEG | NEG | NEG | NEG | NEG | NEG | NEG | NEG | NEG | NEG | NEG | NEG | NEG | NEG | NEG |
| | NEG | NEG | NEG | NEG | NEG | NEG | NEG | NEG | NEG | NEG | NEG | NEG | NEG | NEG | NEG | NEG | NEG | NEG | NEG | NEG | NEG | NEG | NEG | NEG | NEG | NEG | NEG | NEG | NEG | NEG | NEG | NEG | NEG | NEG |
| | NEG | NEG | NEG | NEG | NEG | NEG | NEG | NEG | NEG | NEG | NEG | AMB | NEG | NEG | NEG | NEG | NEG | NEG | NEG | NEG | NEG | NEG | NEG | NEG | NEG | NEG | NEG | NEG | NEG | NEG | NEG | NEG | NEG | NEG |
| | NEG | NEG | NEG | NEG | NEG | NEG | NEG | NEG | NEG | NEG | NEG | NEG | NEG | NEG | NEG | NEG | NEG | NEG | NEG | NEG | NEG | NEG | NEG | NEG | NEG | NEG | NEG | NEG | NEG | NEG | NEG | NEG | NEG | NEG |
| | NEG | NEG | NEG | NEG | NEG | NEG | NEG | NEG | NEG | NEG | NEG | NEG | NEG | NEG | NEG | NEG | NEG | NEG | NEG | NEG | NEG | NEG | NEG | NEG | NEG | NEG | NEG | NEG | NEG | NEG | NEG | NEG | NEG | NEG |
| Dresden 07-7829 | NEG | NEG | NEG | NEG | NEG | NEG | NEG | NEG | NEG | NEG | NEG | NEG | NEG | NEG | NEG | NEG | NEG | NEG | NEG | NEG | NEG | NEG | NEG | NEG | NEG | NEG | NEG | NEG | NEG | NEG | NEG | NEG | NEG | NEG |
| Dresden 07-8176 | NEG | NEG | NEG | NEG | NEG | NEG | NEG | NEG | NEG | NEG | NEG | NEG | NEG | NEG | NEG | NEG | NEG | NEG | NEG | NEG | NEG | NEG | NEG | NEG | NEG | NEG | NEG | NEG | NEG | NEG | NEG | NEG | NEG | NEG |
| >HP-32 | NEG | NEG | NEG | NEG | NEG | NEG | NEG | NEG | NEG | NEG | NEG | NEG | NEG | NEG | NEG | NEG | NEG | NEG | NEG | NEG | NEG | NEG | NEG | NEG | NEG | NEG | NEG | NEG | NEG | NEG | NEG | NEG | NEG | NEG |
| C. difficile 2007855 (GenBank FN665654) in silico prediction | NEG | NEG | NEG | NEG | NEG | NEG | NEG | NEG | NEG | NEG | NEG | NEG | NEG | NEG | NEG | NEG | NEG | NEG | NEG | NEG | NEG | NEG | NEG | NEG | NEG | NEG | NEG | NEG | NEG | NEG | NEG | NEG | NEG | NEG |
| C. difficile QCD-32g58 (GenBank AAML) in silico prediction | NEG | NEG | NEG | NEG | NEG | NEG | NEG | NEG | NEG | NEG | NEG | NEG | NEG | NEG | NEG | NEG | NEG | NEG | NEG | NEG | NEG | NEG | NEG | NEG | NEG | NEG | NEG | NEG | NEG | NEG | NEG | NEG | NEG | NEG |
| C. difficile QCD-37x79 (GenBank ABHG) in silico prediction | NEG | NEG | NEG | NEG | NEG | NEG | NEG | NEG | NEG | NEG | NEG | NEG | NEG | NEG | NEG | NEG | NEG | NEG | NEG | NEG | NEG | NEG | NEG | NEG | NEG | NEG | NEG | NEG | NEG | NEG | NEG | NEG | NEG | NEG |
| C. difficile QCD-66c26 (GenBank ABFD) in silico prediction | NEG | NEG | NEG | NEG | NEG | NEG | NEG | NEG | NEG | NEG | NEG | NEG | NEG | NEG | NEG | NEG | NEG | NEG | NEG | NEG | NEG | NEG | NEG | NEG | NEG | NEG | NEG | NEG | NEG | NEG | NEG | NEG | NEG | NEG |
| C. difficile R20291 (GenBank FN545816) in silico prediction | NEG | NEG | NEG | NEG | NEG | NEG | NEG | NEG | NEG | NEG | NEG | NEG | NEG | NEG | NEG | NEG | NEG | NEG | NEG | NEG | NEG | NEG | NEG | NEG | NEG | NEG | NEG | NEG | NEG | NEG | NEG | NEG | NEG | NEG |
| C. difficile Strain R20291 | NEG | NEG | NEG | NEG | NEG | NEG | NEG | NEG | NEG | NEG | NEG | NEG | NEG | NEG | NEG | NEG | NEG | NEG | NEG | NEG | NEG | NEG | NEG | NEG | NEG | NEG | NEG | NEG | NEG | NEG | NEG | NEG | NEG | NEG |
| | NEG | NEG | NEG | NEG | NEG | NEG | NEG | NEG | NEG | NEG | NEG | AMB | NEG | NEG | NEG | NEG | NEG | NEG | NEG | NEG | NEG | NEG | NEG | NEG | NEG | NEG | NEG | NEG | NEG | NEG | NEG | NEG | NEG | NEG |
| | NEG | NEG | NEG | NEG | NEG | NEG | NEG | NEG | NEG | NEG | NEG | NEG | NEG | NEG | NEG | NEG | NEG | NEG | NEG | NEG | NEG | NEG | NEG | NEG | NEG | NEG | NEG | NEG | NEG | NEG | NEG | NEG | NEG | NEG |
| | NEG | NEG | NEG | NEG | NEG | NEG | NEG | NEG | NEG | NEG | NEG | NEG | NEG | NEG | NEG | NEG | NEG | NEG | NEG | NEG | NEG | NEG | NEG | NEG | NEG | NEG | NEG | NEG | NEG | NEG | NEG | NEG | NEG | NEG |
| CLADE 3 (CC5 /Ribo 23 Complex) | | | | | | | | | | | | | | | | | | | | | | | | | | | | | | | | | | |
| >HP-33 | NEG | NEG | NEG | NEG | NEG | NEG | NEG | NEG | NEG | NEG | NEG | NEG | NEG | NEG | NEG | NEG | NEG | POS | NEG | NEG | NEG | NEG | NEG | NEG | NEG | NEG | NEG | NEG | NEG | NEG | NEG | NEG | NEG | NEG |
| Freiburg872-34.872-DBK0000295 | NEG | NEG | NEG | NEG | NEG | NEG | NEG | NEG | NEG | NEG | NEG | NEG | NEG | NEG | NEG | NEG | NEG | POS | NEG | NEG | NEG | NEG | NEG | NEG | NEG | NEG | NEG | NEG | NEG | NEG | NEG | NEG | NEG | NEG |
| | NEG | NEG | NEG | NEG | NEG | NEG | NEG | NEG | NEG | NEG | NEG | NEG | NEG | NEG | NEG | NEG | NEG | POS | NEG | NEG | NEG | NEG | NEG | NEG | NEG | NEG | NEG | NEG | NEG | NEG | NEG | NEG | NEG | NEG |
| Freiburg-773-19.773-DBK0000274 | NEG | NEG | NEG | NEG | NEG | NEG | NEG | NEG | NEG | NEG | NEG | NEG | NEG | NEG | NEG | NEG | NEG | POS | NEG | NEG | NEG | NEG | NEG | NEG | NEG | NEG | NEG | NEG | NEG | NEG | NEG | NEG | NEG | NEG |
| | NEG | NEG | NEG | NEG | NEG | NEG | NEG | NEG | NEG | NEG | NEG | NEG | NEG | NEG | NEG | NEG | NEG | POS | NEG | NEG | NEG | NEG | NEG | NEG | NEG | NEG | NEG | NEG | NEG | NEG | NEG | NEG | NEG | NEG |
| >HP-34 | NEG | NEG | NEG | NEG | NEG | NEG | NEG | NEG | NEG | NEG | NEG | NEG | NEG | NEG | NEG | NEG | NEG | NEG | NEG | NEG | NEG | NEG | NEG | NEG | NEG | NEG | NEG | NEG | NEG | NEG | NEG | NEG | NEG | NEG |
| Jena_0018/11 | NEG | NEG | NEG | NEG | NEG | NEG | NEG | NEG | NEG | NEG | NEG | NEG | NEG | NEG | NEG | NEG | NEG | NEG | NEG | NEG | NEG | NEG | NEG | NEG | NEG | NEG | NEG | NEG | NEG | NEG | NEG | NEG | NEG | NEG |
| CLADE 4 (CC37/Ribo 17 Complex) | | | | | | | | | | | | | | | | | | | | | | | | | | | | | | | | | | |
| >HP-35 | NEG | NEG | NEG | NEG | NEG | NEG | NEG | NEG | NEG | NEG | NEG | NEG | NEG | NEG | NEG | NEG | NEG | NEG | NEG | NEG | NEG | NEG | NEG | NEG | NEG | NEG | NEG | POS | NEG | NEG | NEG | NEG | NEG | POS |
| C. difficile 002-P50-2011 (GenBank AGAA) in silico prediction | NEG | NEG | NEG | NEG | NEG | NEG | NEG | NEG | NEG | NEG | NEG | NEG | NEG | NEG | NEG | NEG | NEG | NEG | NEG | NEG | NEG | NEG | NEG | NEG | NEG | NEG | NEG | POS | NEG | NEG | NEG | NEG | NEG | POS |
| C. difficile 050-P50-2011 (GenBank AGAB) in silico prediction | NEG | NEG | NEG | NEG | NEG | NEG | NEG | NEG | NEG | NEG | NEG | NEG | NEG | NEG | NEG | NEG | NEG | NEG | NEG | NEG | NEG | NEG | NEG | NEG | NEG | NEG | NEG | POS | NEG | NEG | NEG | NEG | NEG | POS |
| C. difficile M68 (GenBank FN668375) in silico prediction | NEG | NEG | NEG | NEG | NEG | NEG | NEG | NEG | NEG | NEG | NEG | NEG | NEG | NEG | NEG | NEG | NEG | NEG | NEG | NEG | NEG | NEG | NEG | NEG | NEG | NEG | NEG | POS | NEG | NEG | NEG | NEG | NEG | POS |
| C. difficile CF5 (GenBank FN665652) in silico prediction | NEG | NEG | NEG | NEG | NEG | NEG | NEG | NEG | NEG | NEG | NEG | NEG | NEG | NEG | NEG | NEG | NEG | NEG | NEG | NEG | NEG | NEG | NEG | NEG | NEG | NEG | NEG | POS | NEG | NEG | NEG | NEG | NEG | POS |
| C. difficile Strain CF5 | NEG | NEG | NEG | NEG | NEG | NEG | NEG | NEG | NEG | NEG | NEG | NEG | NEG | NEG | NEG | NEG | NEG | NEG | NEG | NEG | NEG | NEG | NEG | NEG | NEG | NEG | NEG | POS | NEG | NEG | NEG | NEG | NEG | AMB |
| | NEG | NEG | NEG | NEG | NEG | NEG | NEG | NEG | NEG | NEG | NEG | NEG | NEG | NEG | NEG | NEG | NEG | NEG | NEG | NEG | NEG | NEG | NEG | NEG | NEG | NEG | NEG | POS | NEG | NEG | NEG | NEG | NEG | POS |
| | NEG | NEG | NEG | NEG | NEG | NEG | NEG | NEG | NEG | NEG | NEG | NEG | NEG | NEG | NEG | NEG | NEG | NEG | NEG | NEG | NEG | NEG | NEG | NEG | NEG | NEG | NEG | POS | NEG | NEG | NEG | NEG | NEG | POS |
| | NEG | NEG | NEG | NEG | NEG | NEG | NEG | NEG | NEG | NEG | NEG | NEG | NEG | NEG | NEG | NEG | NEG | NEG | NEG | NEG | NEG | NEG | NEG | NEG | NEG | NEG | NEG | POS | NEG | NEG | NEG | NEG | NEG | AMB |
| | NEG | NEG | NEG | NEG | NEG | NEG | NEG | NEG | NEG | NEG | NEG | NEG | NEG | NEG | NEG | NEG | NEG | NEG | NEG | NEG | NEG | NEG | NEG | NEG | NEG | NEG | NEG | POS | NEG | NEG | NEG | NEG | NEG | POS |
| | NEG | NEG | NEG | NEG | NEG | NEG | NEG | NEG | NEG | NEG | NEG | NEG | NEG | NEG | NEG | NEG | NEG | NEG | NEG | NEG | NEG | NEG | NEG | NEG | NEG | NEG | NEG | POS | NEG | NEG | NEG | NEG | NEG | POS |
| Freiburg-736-16.736-DBK0000233 | NEG | NEG | NEG | NEG | NEG | NEG | NEG | NEG | NEG | NEG | NEG | NEG | NEG | NEG | NEG | NEG | NEG | NEG | NEG | NEG | NEG | NEG | NEG | NEG | NEG | NEG | NEG | POS | NEG | NEG | NEG | NEG | NEG | POS |
| | NEG | NEG | NEG | NEG | NEG | NEG | NEG | NEG | NEG | NEG | NEG | NEG | NEG | NEG | NEG | NEG | NEG | NEG | NEG | NEG | NEG | NEG | NEG | NEG | NEG | NEG | NEG | POS | NEG | NEG | NEG | NEG | NEG | POS |
| >HP-36 | NEG | NEG | NEG | NEG | NEG | POS | NEG | NEG | NEG | NEG | NEG | NEG | NEG | NEG | NEG | NEG | NEG | NEG | NEG | NEG | NEG | NEG | NEG | NEG | NEG | NEG | NEG | NEG | NEG | NEG | NEG | NEG | NEG | NEG |
| Freiburg-642-5.642-DBK0000281 | NEG | NEG | NEG | NEG | NEG | POS | NEG | NEG | NEG | NEG | NEG | NEG | NEG | NEG | NEG | NEG | NEG | NEG | NEG | NEG | NEG | NEG | NEG | NEG | NEG | NEG | NEG | NEG | NEG | NEG | NEG | NEG | NEG | NEG |
| | NEG | NEG | NEG | NEG | NEG | POS | NEG | NEG | NEG | NEG | NEG | NEG | NEG | NEG | NEG | NEG | NEG | NEG | NEG | NEG | NEG | NEG | NEG | NEG | NEG | NEG | NEG | NEG | NEG | NEG | NEG | NEG | NEG | NEG |
| CLADE 5 (CC11 /Ribo 78 Complex) | | | | | | | | | | | | | | | | | | | | | | | | | | | | | | | | | | |
| >HP-37 | NEG | NEG | NEG | NEG | NEG | NEG | NEG | NEG | NEG | NEG | NEG | NEG | NEG | NEG | NEG | NEG | NEG | NEG | NEG | NEG | NEG | NEG | NEG | NEG | NEG | NEG | POS | NEG | NEG | NEG | NEG | NEG | NEG | NEG |
| C. difficile NAP08 (GenBank ADNX) in silico prediction | NEG | NEG | NEG | NEG | NEG | NEG | NEG | NEG | NEG | NEG | NEG | NEG | NEG | NEG | NEG | NEG | NEG | NEG | NEG | NEG | NEG | NEG | NEG | NEG | NEG | NEG | POS | NEG | NEG | NEG | NEG | NEG | NEG | NEG |
| C. difficile NAP07 (GenBank ADVM) in silico prediction | NEG | NEG | NEG | NEG | NEG | NEG | NEG | NEG | NEG | NEG | NEG | NEG | NEG | NEG | NEG | NEG | NEG | NEG | NEG | NEG | NEG | NEG | NEG | NEG | NEG | NEG | POS | NEG | NEG | NEG | NEG | NEG | NEG | NEG |
| C. difficile M120 (GenBank FN665653) in silico prediction | NEG | NEG | NEG | NEG | NEG | NEG | NEG | NEG | NEG | NEG | NEG | NEG | NEG | NEG | NEG | NEG | NEG | NEG | NEG | NEG | NEG | NEG | NEG | NEG | NEG | NEG | POS | NEG | NEG | NEG | NEG | NEG | NEG | NEG |
| C. difficile Strain M120 | NEG | NEG | NEG | NEG | NEG | NEG | NEG | NEG | NEG | NEG | NEG | NEG | NEG | NEG | NEG | NEG | NEG | NEG | NEG | NEG | NEG | NEG | NEG | NEG | NEG | NEG | AMB | NEG | NEG | NEG | NEG | NEG | NEG | NEG |
| | NEG | NEG | NEG | NEG | NEG | NEG | NEG | NEG | NEG | NEG | NEG | AMB | NEG | NEG | NEG | NEG | NEG | NEG | NEG | NEG | NEG | NEG | NEG | NEG | NEG | NEG | POS | NEG | NEG | NEG | NEG | NEG | NEG | NEG |
| | NEG | NEG | NEG | NEG | NEG | NEG | NEG | NEG | NEG | NEG | NEG | NEG | NEG | NEG | NEG | NEG | NEG | NEG | NEG | NEG | NEG | NEG | NEG | NEG | NEG | NEG | POS | NEG | NEG | NEG | NEG | NEG | NEG | NEG |
| | NEG | NEG | NEG | NEG | NEG | NEG | NEG | NEG | NEG | NEG | NEG | NEG | NEG | NEG | NEG | NEG | NEG | NEG | NEG | NEG | NEG | NEG | NEG | NEG | NEG | NEG | AMB | NEG | NEG | NEG | NEG | NEG | NEG | NEG |
| | NEG | NEG | NEG | NEG | NEG | NEG | NEG | NEG | NEG | NEG | NEG | NEG | NEG | NEG | NEG | NEG | NEG | NEG | NEG | NEG | NEG | NEG | NEG | NEG | NEG | NEG | POS | NEG | NEG | NEG | NEG | NEG | NEG | NEG |
| Freiburg_69.1017 | NEG | NEG | NEG | NEG | NEG | NEG | NEG | NEG | NEG | NEG | NEG | NEG | NEG | NEG | NEG | NEG | NEG | NEG | NEG | NEG | NEG | NEG | NEG | NEG | NEG | NEG | NEG | NEG | NEG | NEG | NEG | NEG | NEG | NEG |
| Freiburg-1051-77.1051-DBK0000293 | NEG | NEG | NEG | NEG | NEG | NEG | NEG | NEG | NEG | NEG | NEG | NEG | NEG | NEG | NEG | NEG | NEG | NEG | NEG | NEG | NEG | NEG | NEG | NEG | NEG | NEG | POS | NEG | NEG | NEG | NEG | NEG | NEG | NEG |
| | NEG | NEG | NEG | NEG | NEG | NEG | NEG | NEG | NEG | NEG | NEG | NEG | NEG | NEG | NEG | NEG | NEG | NEG | NEG | NEG | NEG | NEG | NEG | NEG | NEG | NEG | POS | NEG | NEG | NEG | NEG | NEG | NEG | NEG |
| Freiburg-649-6.649-DBK0000235 | NEG | NEG | NEG | NEG | NEG | NEG | NEG | NEG | NEG | NEG | NEG | NEG | NEG | NEG | NEG | NEG | NEG | NEG | NEG | NEG | NEG | NEG | NEG | NEG | NEG | NEG | POS | NEG | NEG | NEG | NEG | NEG | NEG | NEG |
| | NEG | NEG | NEG | NEG | NEG | NEG | NEG | NEG | NEG | NEG | NEG | NEG | NEG | NEG | NEG | NEG | NEG | NEG | NEG | NEG | NEG | NEG | NEG | NEG | NEG | NEG | POS | NEG | NEG | NEG | NEG | NEG | NEG | NEG |
| Freiburg-700-11.7-DBK0000242 | NEG | NEG | NEG | NEG | NEG | NEG | NEG | NEG | NEG | NEG | NEG | NEG | NEG | NEG | NEG | NEG | NEG | NEG | NEG | NEG | NEG | NEG | NEG | NEG | NEG | NEG | POS | NEG | NEG | NEG | NEG | NEG | NEG | NEG |
| | NEG | NEG | NEG | NEG | NEG | NEG | NEG | NEG | NEG | NEG | NEG | NEG | NEG | NEG | NEG | NEG | NEG | NEG | NEG | NEG | NEG | NEG | NEG | NEG | NEG | NEG | POS | NEG | NEG | NEG | NEG | NEG | NEG | NEG |
| Freiburg-855-30.855-DBK0000277 | NEG | NEG | NEG | NEG | NEG | NEG | NEG | NEG | NEG | NEG | NEG | NEG | NEG | NEG | NEG | NEG | NEG | NEG | NEG | NEG | NEG | NEG | NEG | NEG | NEG | NEG | POS | NEG | NEG | NEG | NEG | NEG | NEG | NEG |
| | NEG | NEG | NEG | NEG | NEG | NEG | NEG | NEG | NEG | NEG | NEG | NEG | NEG | NEG | NEG | NEG | NEG | NEG | NEG | NEG | NEG | NEG | NEG | NEG | NEG | NEG | POS | NEG | NEG | NEG | NEG | NEG | NEG | NEG |
| Dresden 08_1353 | NEG | NEG | NEG | NEG | NEG | NEG | NEG | NEG | NEG | NEG | NEG | NEG | NEG | NEG | NEG | NEG | NEG | NEG | NEG | NEG | NEG | NEG | NEG | NEG | NEG | NEG | POS | NEG | NEG | NEG | NEG | NEG | NEG | NEG |
| Dresden 08_574 | NEG | NEG | NEG | NEG | NEG | NEG | NEG | NEG | NEG | NEG | NEG | NEG | NEG | NEG | NEG | NEG | NEG | NEG | NEG | NEG | NEG | NEG | NEG | NEG | NEG | NEG | POS | NEG | NEG | NEG | NEG | NEG | NEG | NEG |
| Dresden 09_2650 | NEG | NEG | NEG | NEG | NEG | NEG | NEG | NEG | NEG | NEG | NEG | NEG | NEG | NEG | NEG | NEG | NEG | NEG | NEG | NEG | NEG | NEG | NEG | NEG | NEG | NEG | POS | NEG | NEG | NEG | NEG | NEG | NEG | NEG |
| >HP-38 | NEG | NEG | NEG | NEG | NEG | NEG | NEG | NEG | NEG | NEG | NEG | NEG | NEG | NEG | NEG | NEG | NEG | NEG | NEG | NEG | NEG | NEG | NEG | NEG | NEG | NEG | POS | NEG | NEG | NEG | NEG | NEG | NEG | NEG |
| C. difficile 6466 (GenBank ADDE) in silico prediction | NEG | NEG | NEG | NEG | NEG | NEG | NEG | NEG | NEG | NEG | NEG | NEG | NEG | NEG | NEG | NEG | NEG | NEG | NEG | NEG | NEG | NEG | NEG | NEG | NEG | NEG | POS | NEG | NEG | NEG | NEG | NEG | NEG | NEG |
| >HP-39 | NEG | NEG | NEG | NEG | NEG | NEG | NEG | NEG | NEG | NEG | NEG | NEG | NEG | NEG | NEG | NEG | NEG | NEG | NEG | NEG | NEG | NEG | NEG | NEG | NEG | NEG | NEG | NEG | NEG | NEG | NEG | NEG | NEG | NEG |
| C. difficile QCD-23m63 (GenBank ABKL) in silico prediction | NEG | NEG | NEG | NEG | NEG | NEG | NEG | NEG | NEG | NEG | NEG | NEG | NEG | NEG | NEG | NEG | NEG | NEG | NEG | NEG | NEG | NEG | NEG | NEG | NEG | NEG | NEG | NEG | NEG | NEG | NEG | NEG | NEG | NEG |
| Freiburg-956-51.956-DBK0000266 | NEG | NEG | NEG | NEG | NEG | NEG | NEG | NEG | NEG | NEG | NEG | NEG | NEG | NEG | NEG | NEG | NEG | NEG | NEG | NEG | NEG | NEG | NEG | NEG | NEG | NEG | NEG | NEG | NEG | NEG | NEG | NEG | NEG | NEG |
| Freiburg_50.963 | NEG | NEG | NEG | NEG | NEG | NEG | NEG | NEG | NEG | NEG | NEG | NEG | NEG | NEG | NEG | NEG | NEG | NEG | NEG | NEG | NEG | NEG | NEG | NEG | NEG | NEG | NEG | NEG | NEG | NEG | NEG | NEG | NEG | NEG |
| "CLADE 6" | | | | | | | | | | | | | | | | | | | | | | | | | | | | | | | | | | |
| >HP-40 | NEG | NEG | NEG | NEG | NEG | NEG | NEG | NEG | NEG | NEG | NEG | POS | NEG | NEG | NEG | NEG | NEG | NEG | NEG | NEG | NEG | NEG | NEG | NEG | NEG | NEG | NEG | NEG | NEG | NEG | NEG | NEG | NEG | NEG |
| C. difficile 6503 (GenBank ADEI) in silico prediction | NEG | NEG | NEG | NEG | NEG | NEG | NEG | NEG | NEG | NEG | NEG | POS | NEG | NEG | NEG | NEG | NEG | NEG | NEG | NEG | NEG | NEG | NEG | NEG | NEG | NEG | NEG | NEG | NEG | NEG | NEG | NEG | NEG | NEG |
